# Buchwald–Hartwig Amination of Aryl Halides with Heterocyclic Amines in the Synthesis of Highly Fluorescent Benzodifuran-Based Star-Shaped Organic Semiconductors

**DOI:** 10.1021/acs.joc.1c01583

**Published:** 2021-12-03

**Authors:** Mariusz J. Bosiak, Alicja A. Zielińska, Piotr Trzaska, Dariusz Kędziera, Jörg Adams

**Affiliations:** † Department of Organic Chemistry, Faculty of Chemistry, Nicolaus Copernicus University in Toruń, 7 Gagarin Street, 87-100 Toruń, Poland; ‡ Doctoral School of Exact and Natural Sciences “Academia Scientiarum Thoruniensis”, Nicolaus Copernicus University in Toruń, 5 Grudziądzka Street, 87-100 Toruń, Poland; § Department of Chemistry of Materials Adsorption and Catalysis, Faculty of Chemistry, Nicolaus Copernicus University in Toruń, 7 Gagarin Street, 87-100 Toruń, Poland; ∥ Institute of Physical Chemistry, Clausthal University of Technology, Arnold-Sommerfeld-Str. 4, 38678 Clausthal-Zellerfeld, Germany; ⊥ Noctiluca SA, 7/41B Gagarina Street, 87-100 Toruń, Poland

## Abstract

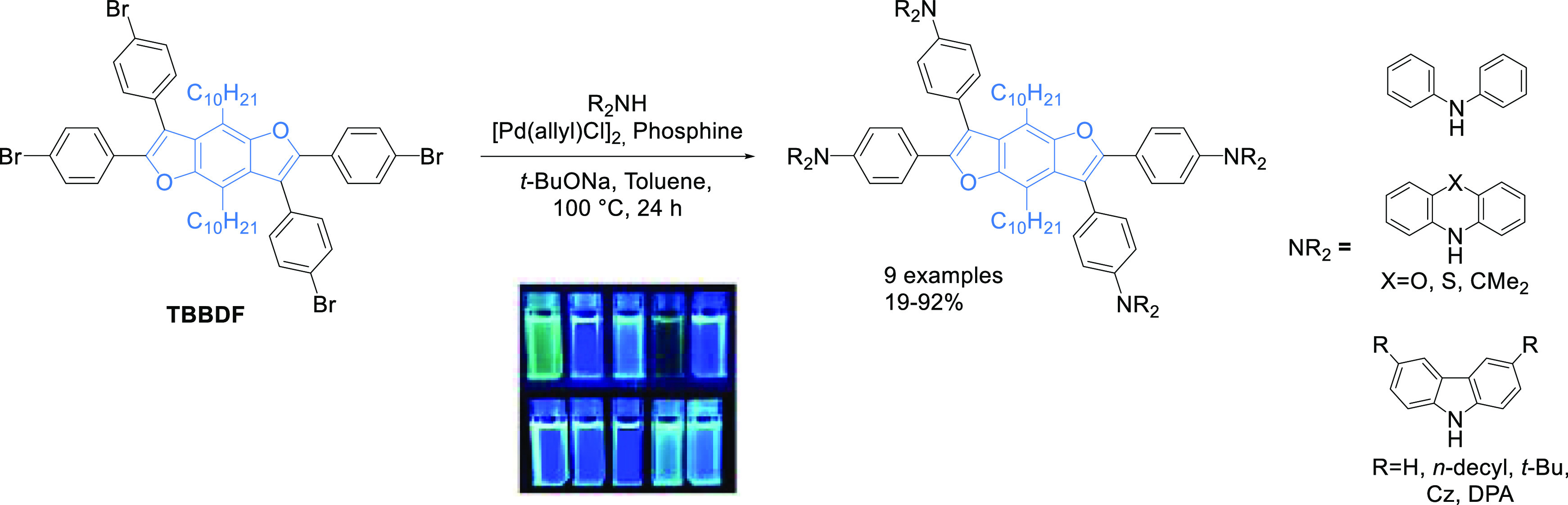

The study of palladium-catalyzed amination of bromobenzene with aromatic and heterocyclic amines, widely used in the synthesis of organic semiconductors, was performed. The best conditions for the coupling of aryl bromides with carbazole, diphenylamine, phenoxazine, phenothiazine, 9,9-dimethyl-9,10-dihydroacridine, and their derivatives have been developed. Based on the results, nine new star-shaped organic semiconductors, exhibiting up to 100% fluorescent quantum yield in the 400–550 nm range, have been synthesized in good yields. The TDDFT calculations of the absorption spectra revealed a good correlation with experimental results and slight solvatochromic effects with a change in the polarity of the solvent.

## Introduction

Benzodifurans (**BDFs**), due to their p-type organic semiconductor properties, excellent light absorption and emission capability, and high hole mobility, are a group of compounds with a great potential application as luminescent and electroluminescent materials.^1−4^ In addition, they are much less studied compared to benzodithiophenes widely used in optoelectronics. The appropriate molecular design of the **BDF**-based semiconductors allows for their application in many fields, including molecular switches and electrical regulators,^5−7^ high-affinity fluorescent probes,^8^ potential therapeutic agents,^9^ dye-sensitized solar cell sensitizers,^10−13^ polymer materials in polymer solar cells,^14−22^ organic solar cells,^23,24^ organic thin-film transistor materials,^25,26^ and different layers in organic light-emitting diodes (LEDs).^27−34^

A huge number of organic semiconductors used in optoelectronics as electron transport layers (**ETL**) and electron injection layers (**EIL**),^35,36^ hole transport layers (**HTL**) and hole injection layers (**HIL**),^37,38^ hosts for phosphorescent and thermally activated delayed fluorescent (TADF) materials,^39−43^ and TADF materials themselves^44−51^ contain aromatic or heterocyclic amines such as carbazole (**Cz**), diphenylamine (**DPA**), phenoxazine (**PXZ**), phenothiazine (**PTZ**), and 9,9-dimethyl-9,10-dihydroacridine (**DMAC**). Although in the literature one can find numerous examples of coupling of the above-mentioned amines and their derivatives,^52−54^ the comprehensive study of their palladium-catalyzed coupling with aryl halides has not yet been performed.

Herein, the study of the Buchwald–Hartwig amination of aryl bromides with the amines mentioned above, leading to novel star-shaped **BDF** derivatives, along with their density functional theory (DFT) and spectral characteristics, is described.

## Results and Discussion

**Scheme 1:**
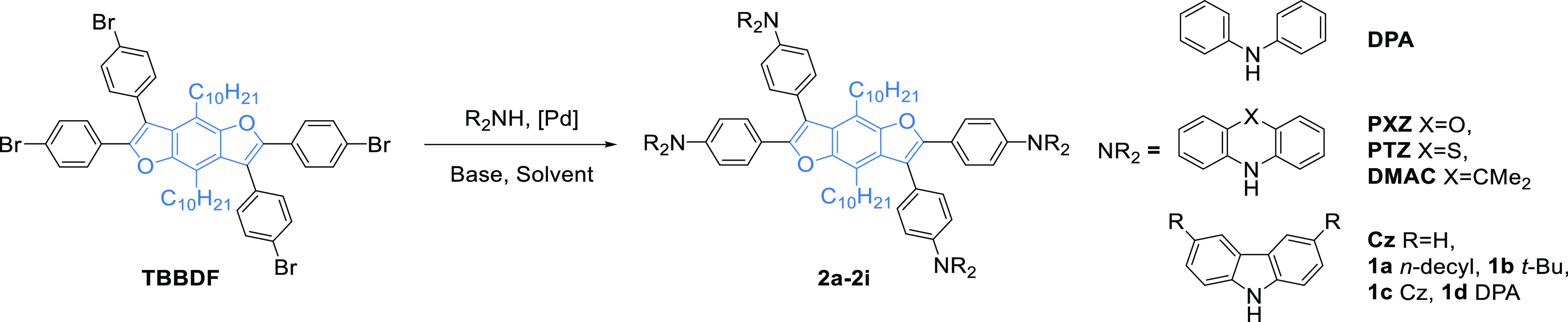
Buchwald–Hartwig Coupling Leading to Star-Shaped **BDFs**

The benzodifuran core (**TBBDF**), containing four *para*-bromophenylene groups and long alkyl chains in positions 4 and 8 of the **BD**F core, to improve the solubility of the desired compounds, was synthesized according to the procedure described earlier by our team^55^ and directed to the Buchwald–Hartwig amination with **Cz**, **DPA**, **PXZ**, **PTZ**, **DMAC**, and their derivatives (Scheme 1).

### Optimization of the Reaction Conditions

For the efficient synthesis of the expanded star-shaped systems by the Buchwald–Hartwig amination, the coupling conditions for each secondary amine with less-demanding bromobenzene were developed. The screening tests included selecting the palladium precatalyst, phosphine ligand, solvent, and base for the reaction.

### Catalysts

To identify the best catalytic system, commercially available palladium catalysts and phosphine ligands were tested (Table 1). It was found that Pd(dppf)Cl_2_, Pd(PPh_3_)_2_Cl_2_, and Pd(PPh_3_)_2_(OAc)_2_ (entries 19–21) were ineffective in **Cz** coupling, but they gave average results for **DPA** and **DMAC** and good conversion levels for **PXZ** and **PTZ**. For [Pd]/phosphine catalytic systems, we decided to use [Pd(allyl)Cl]_2_ dimer as a palladium source, although the Pd_2_(dba)_3_ precatalyst gave comparable results (entries 6 and 7) when sodium *tert*-butanolate in toluene and XPhos as a ligand were used. It was found that phosphines containing electron-donating groups on the biphenyl moiety gave poor conversion rates in the **Cz** and **DPA** coupling and the average for the other tested amines (entries 16 and 17). The best results for **Cz** coupling were obtained using TrixiePhos and *t*-BuBrettPhos (97%, entries 12 and 14, respectively). For **DPA**, it was [*t*-Bu_3_PH]BF_4_, XPhos, RuPhos, and SPhos (96%, entries 2, 6, 9, and 10). **PXZ** was found to be very easily coupled with bromobenzene, and results of >99% were obtained for eight ligands (entries 4–10, 12, and 13). The best conversion rates for **PTZ** coupling were obtained using DavePhos and XPhos (99%, entries 4 and 6) and for **DMAC**—*t*-BuXPhos (98%) and XPhos (96%) (entries 8 and 6). The most universal ligands were revealed to be XPhos and TrixiePhos, giving conversion rates above 90% for all tested amines.

**Table 1:**
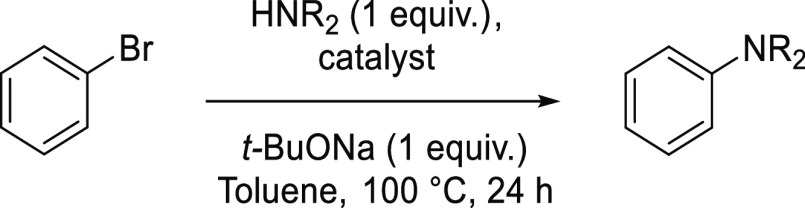
Coupling of Bromobenzene with Secondary Aryl Amines in the Presence of Commercially Available Phosphines and Palladium Catalysts

		conversion [%]^d^				
entry	conditions^a^	**Cz**	**DPA**	**PXZ**	**PTZ**	**DMAC**
1	PPh_3_	0	75	91	90	53
2	[(*t*-Bu)_3_PH]BF_4_	13	**96**	97	95	92
3	JohnPhos	89	89	95	95	90
4	DavePhos	85	94	**>99**	**99**	87
5	CyJohnPhos	86	85	**>99**	92	93
6	XPhos	92	**96**	**>99**	**99**	96
7	XPhos^b^	89	93	**>99**	**99**	88
8	*t*-BuXPhos	94	87	**>99**	95	**98**
9	RuPhos	29	**96**	**>99**	90	89
10	SPhos	57	**96**	**>99**	97	78
11	XantPhos	42	93	95	88	88
12	TrixiePhos	**97**	91	**>99**	97	94
13	*t*-BuDavePhos	88	92	**>99**	96	84
14	*t*-BuBrettPhos	97	36	94	78	63
15	P(*o*-tolyl)_3_	0	89	99	80	81
16	Me_4_*t*-BuXPhos	27	26	95	57	59
17	Me_3_(OMe)*t*-BuXPhos	17	43	85	78	72
18	(*R*/*S*)-BINAP	4	84	86	1	19
19	Pd(dppf)Cl_2_^c^	2	63	89	82	58
20	Pd(PPh_3_)_2_Cl_2_^c^	0	71	95	89	65
21	Pd(PPh_3_)_2_(OAc)_2_^c^	2	38	82	87	43

a [Pd(allyl)Cl]_2_ (1 mol %) and phosphine ligand (4 mol %).

b Pd_2_(dba)_3_ instead of [Pd(allyl)Cl]_2_.

c Catalyst (2 mol %).

d GC–MS, average of two runs.

### Solvent Screening

Using predetermined [Pd(allyl)Cl]_2_/ligand systems for each examined amine, the screening of solvent (Table 2) and base type (Table 3) was carried out. It was found that toluene was the best choice for all tested systems, allowing us to obtain over 95% conversion rates. Satisfactory good results were also obtained when 1,4-dioxane was used.

**Table 2:**
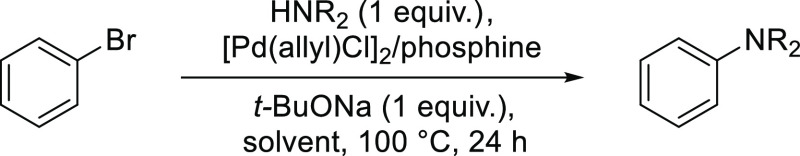
Solvent Screening for the Coupling of Bromobenzene with Secondary Amines

	conversion [%]^a^				
solvent	**Cz**^b^	**DPA**^c^	**PXZ**^c^	**PTZ**^c^	**DMAC**^d^
toluene	**97**	**96**	**>99**	**99**	**98**
1,4-dioxane	87	91	**>99**	98	93
THF	83	67	83	84	72
DMF	28	3	60	19	35
DMSO	2	11	19	12	6

a GC–MS, average of two runs.

b [Pd(allyl)Cl]_2_ (1 mol %) and TrixiePhos (4 mol %).

c [Pd(allyl)Cl]_2_ (1 mol %) and XPhos (4 mol %).

d [Pd(allyl)Cl]_2_ (1 mol %) and *t*-BuXPhos (4 mol %).

**Table 3:**
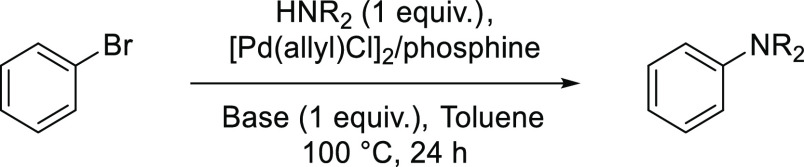
Base Screening for the Coupling of Bromobenzene with Secondary Amines

	conversion [%]^a^				
base	**Cz**^b^	**DPA**^c^	**PXZ**^c^	**PTZ**^c^	**DMAC**^d^
*t*-BuONa	97	**96**	**>99**	**99**	**98**
*t*-BuOLi	**98**	83	89	89	93
K_2_CO_3_	82	35	86	55	64
K_3_PO_4_	42	29	85	54	38
MeMgCl	95	92	90	93	89
Cs_2_CO_3_	96	93	**>99**	77	88
KOH	77	67	87	79	82

a GC–MS, average of two runs.

b [Pd(allyl)Cl]_2_ (1 mol %) and TrixiePhos (4 mol %).

c [Pd(allyl)Cl]_2_ (1 mol %) and XPhos (4 mol %).

d [Pd(allyl)Cl]_2_ (1 mol %) and *t*-BuXPhos (4 mol %).

### Base Screening

The best base for the reaction with **Cz** proved to be *t*-BuOLi (98%), but almost equal yields were obtained for *t*-BuONa and Cs_2_CO_3_ (97 and 96%, respectively). *t*-BuONa seems to be the most universal, although *t*-BuOLi and Cs_2_CO_3_ also gave satisfactory results in individual cases (Table 3). Good results were also obtained using methylmagnesium chloride as a base. Weaker inorganic bases (K_2_CO_3_, K_3_PO_4_, and KOH) gave good results only for coupling with **PXZ**, but the amination with this amine is relatively easy.

### General Conditions

It was found that the Buchwald–Hartwig amination of bromobenzene with secondary amines is most preferably carried out in an environment of relatively non-polar solvents, although the base choice seems to be more complex; however, one can conclude that strong organic bases and Cs_2_CO_3_ will work well in this reaction. The best reaction systems for palladium-catalyzed coupling of bromobenzene with **Cz** proved to be TrixiePhos/*t*-BuOLi/toluene, with **DPA**, **PXZ**, and **PTX**—XPhos/*t*-BuONa/toluene, and with **DMAC**—*t*-BuXPhos/*t*-BuONa/toluene.

### Star-Shaped **BDF** Synthesis

**Scheme 2:**
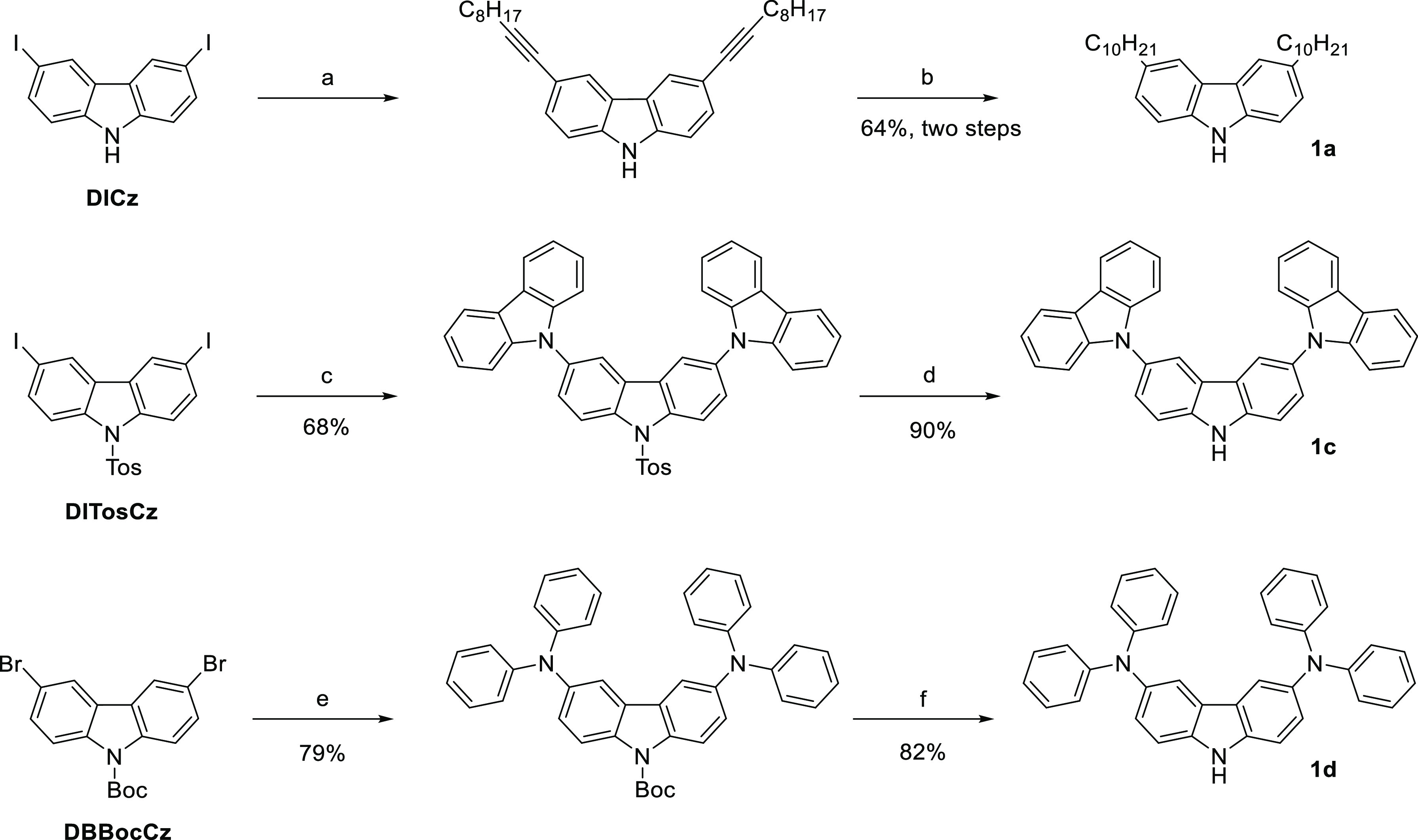
Synthesis of **Cz** Derivatives Substituted in Positions 3 and 6 Conditions: ^a^Pd(dppf)Cl_2_ (1.35 mol %), CuI (2.7 mol %), *i*-Pr_2_NH (4 equiv), 1-decyne (2.5 equiv), toluene, 70 °C, and 3 h; ^b^Pd/C (10% w/w), hydrogen, 1 atm, ethyl acetate, 50 °C, and 12 h; ^c^[Pd(allyl)Cl]_2_ (1 mol %), *t*-BuXPhos (4 mol %), **Cz** (2.1 equiv), *t*-BuOLi (2.1 equiv), 1,4-dioxane, 100 °C, and 24 h; ^d^KOH (12 equiv), THF, DMSO, water, reflux, and 18 h; ^e^Pd_2_(dba)_3_ (2 mol %), XPhos (8 mol %), **DPA** (2.08 equiv), *t*-BuONa (2.08 equiv), toluene, 100 °C, and 24 h; and ^f^DCM, trifluoroacetic acid (10 equiv), RT, and 2.5 h.

The developed conditions were applied to the synthesis of the fluorescent **BDF** star-shaped compounds. Amine **1b** was commercially available (Table 4), while the more complex amines (**1a**, **1c**, and **1d**) were synthesized according to the procedures presented in Scheme 2.

**Table 4:**
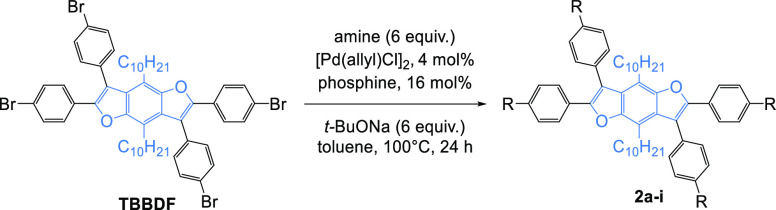

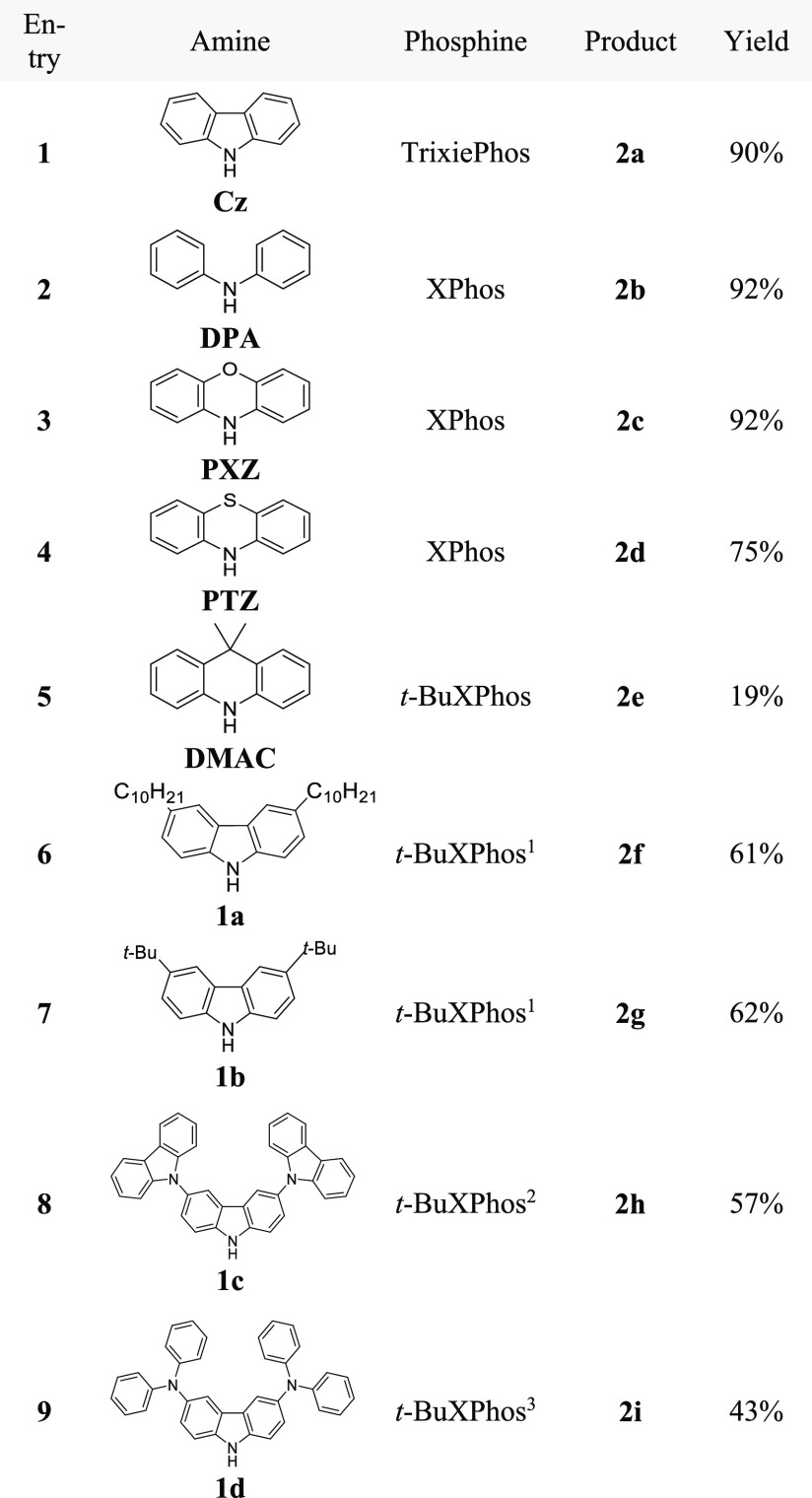
Palladium-Catalyzed Coupling Reaction of **TBBDF** with Amines

a [Pd(allyl)Cl]_2_ (8 mol %) and *t*-BuXPhos (32 mol %).

b Pd(allyl)Cl]_2_ (8 mol %), *t*-BuXPhos (32 mol %), and base *t*-BuOLi instead of *t*-BuONa.

c [Pd(allyl)Cl]_2_ (8 mol %), *t*-BuXPhos (32 mol %), and 170 °C in a sealed tube.

For **1a** synthesis, the Sonogashira reaction of **DICz** and 1-decyne, followed by hydrogen–Pd/C reduction, was performed. Amine **1c** was obtained by the Buchwald–Hartwig reaction between **DITosCz** and **Cz**, followed by basic hydrolysis of the tosyl group. Although the Ullmann synthesis of **1c** is described in the literature, our method allowed us to obtain the product in 68% yield using milder conditions (100 °C, 24 h vs 166 °C, and 48 h).^56^ It was found that due to the better reactivity of aryl iodides compared to bromides, the same product yield was obtained after catalyst loading reduction to 0.5 mol % of [Pd(allyl)Cl]_2_ and 2 mol % of *t*-BuXPhos per one iodine atom.

Compound **1d** was synthesized analogously to **1c**, but the use of the *tert*-butylcarboxycarbonate (**Boc**) protecting group was necessary since the reaction of **DITosCz** and standard [Pd(allyl)Cl]_2_/XPhos or Pd_2_(dba)_3_/[*t*-Bu_3_PH]BF_4_ catalytic systems led to the deiodination of **DITosCz**, resulting in a complex mixture of byproducts. The synthesis of **1d** from **DBBocCz** was also described in the literature;^57^ however, we found that temperature reduction from the literature 220 to 100 °C did not affect the yield of the reaction, which in both cases was 79%, but eliminates difficult to remove byproducts formed in the higher temperature.

Based on the developed amination conditions, it was found that **TBBDF** coupling with **Cz**, **DPA**, **PXZ**, and **PTZ** (Table 4, entries 1–4) undergoes smoothly, yielding desired products in good yields (61–92%), and only for **DMAC**, the yield was significantly lower (19%, entry 5). Since **TBBDF** contains four bromophenylene moieties, the total catalyst loading was 4 mol % of [Pd(allyl)Cl]_2_ and 16 mol % of phosphine ligand. Larger loadings (8 and 32 mol %, respectively) were necessary to achieve satisfactory yields of **2f–2i** (entries 6–9). Also, the ligand of choice for the more sterically demanding amines proved to be *t*-BuXPhos and *t*-BuONa as a base, and only for **2h** (entry 8), *t*-BuOLi gave better results. Additionally, the increase of amine and base load to 1.5 equiv per one bromophenylene moiety allowed us to obtain slightly better results. Even in these conditions, the low yield was obtained for **DMAC** coupling due to the significant amounts of partially substituted and debrominated byproducts, and in this particular case, other catalytic systems should be considered. Another approach that worked well for coupling **1d** with **TBBDF** was using a pressure vessel and temperature above the boiling point of toluene (entry 9). Thus, it was possible to increase the yield of **2i** synthesis from 29 to 43%.

### Suzuki Reaction

**Scheme 3:**
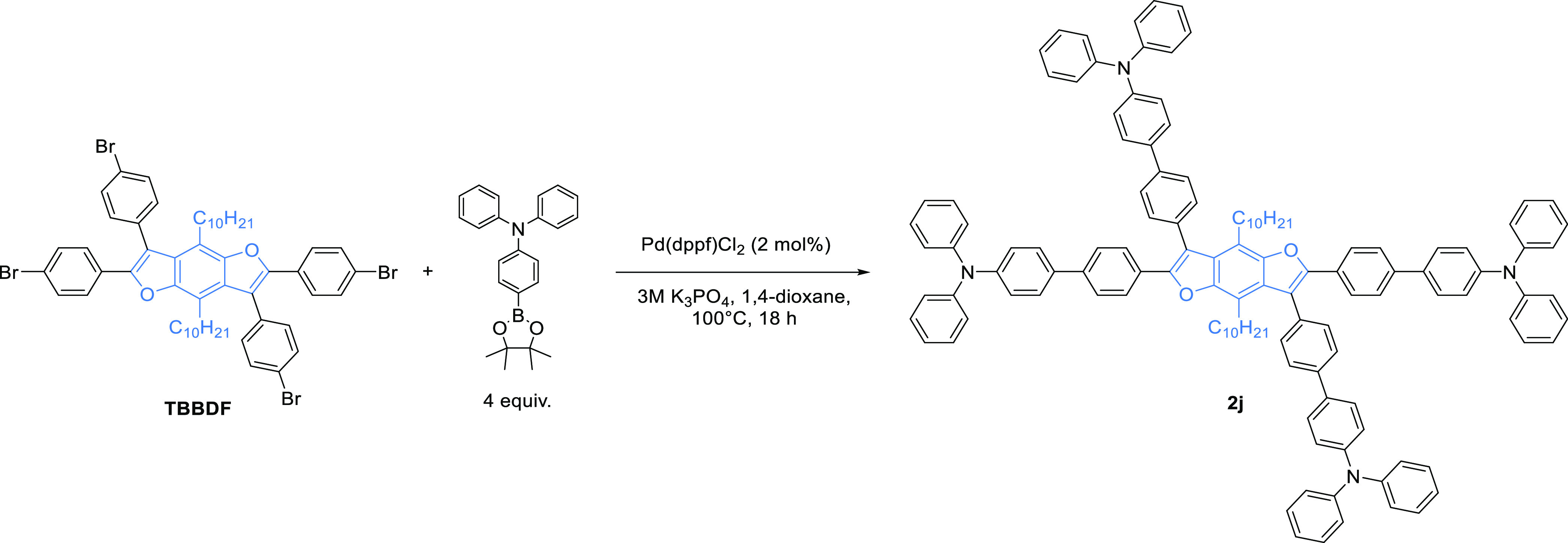
Suzuki Synthesis of **2j**

To estimate the effect of the elongation of the conjugated system of phenylene rings on the spectral properties of star-shaped **BDF**, the **2i**, an analogue of **2b**, was prepared using the Suzuki reaction (Scheme 3).

Commercially available 4-(diphenylamino)phenylboronic acid pinacol ester was reacted with **TBBDF** using [Pd(dppf)Cl]_2_, 3 M aqueous K_3_PO_4_, and 1,4-dioxane to obtain the target compound in a high 90% yield.

### Photophysical Properties

**Figure 1:**
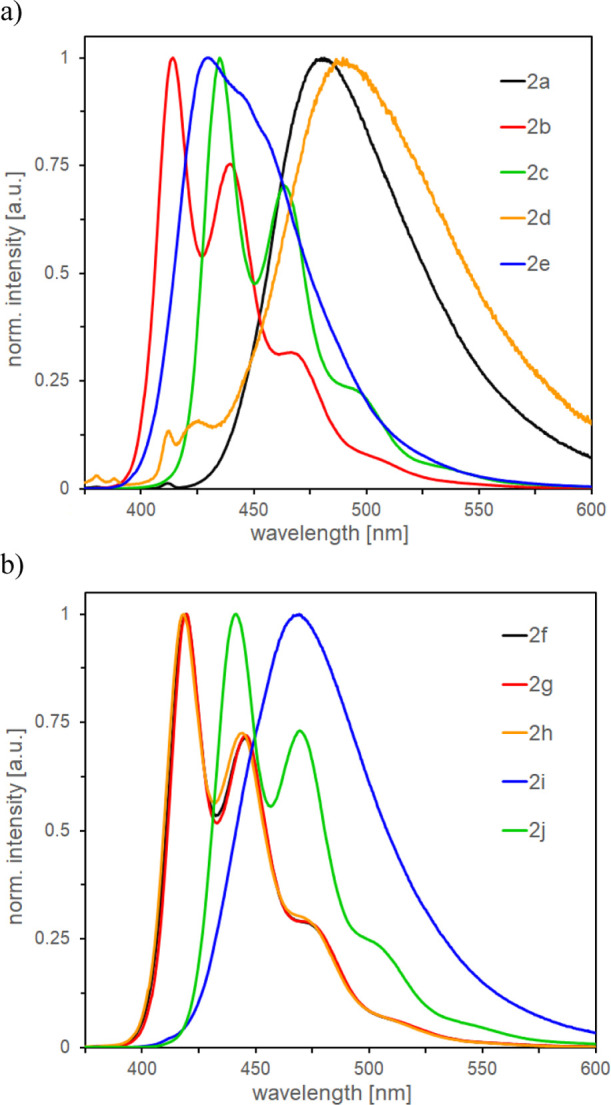
Normalized emission spectra of (a) **2a–2e** and (b) **2f–2j** in toluene. Excitation wavelength λ_ex_ = 366 nm. The concentration is set to an absorbance value between 0.06 and 0.11 to avoid the inner filter effect. The spectra are normalized to the maximal intensity.

**Figure 2:**
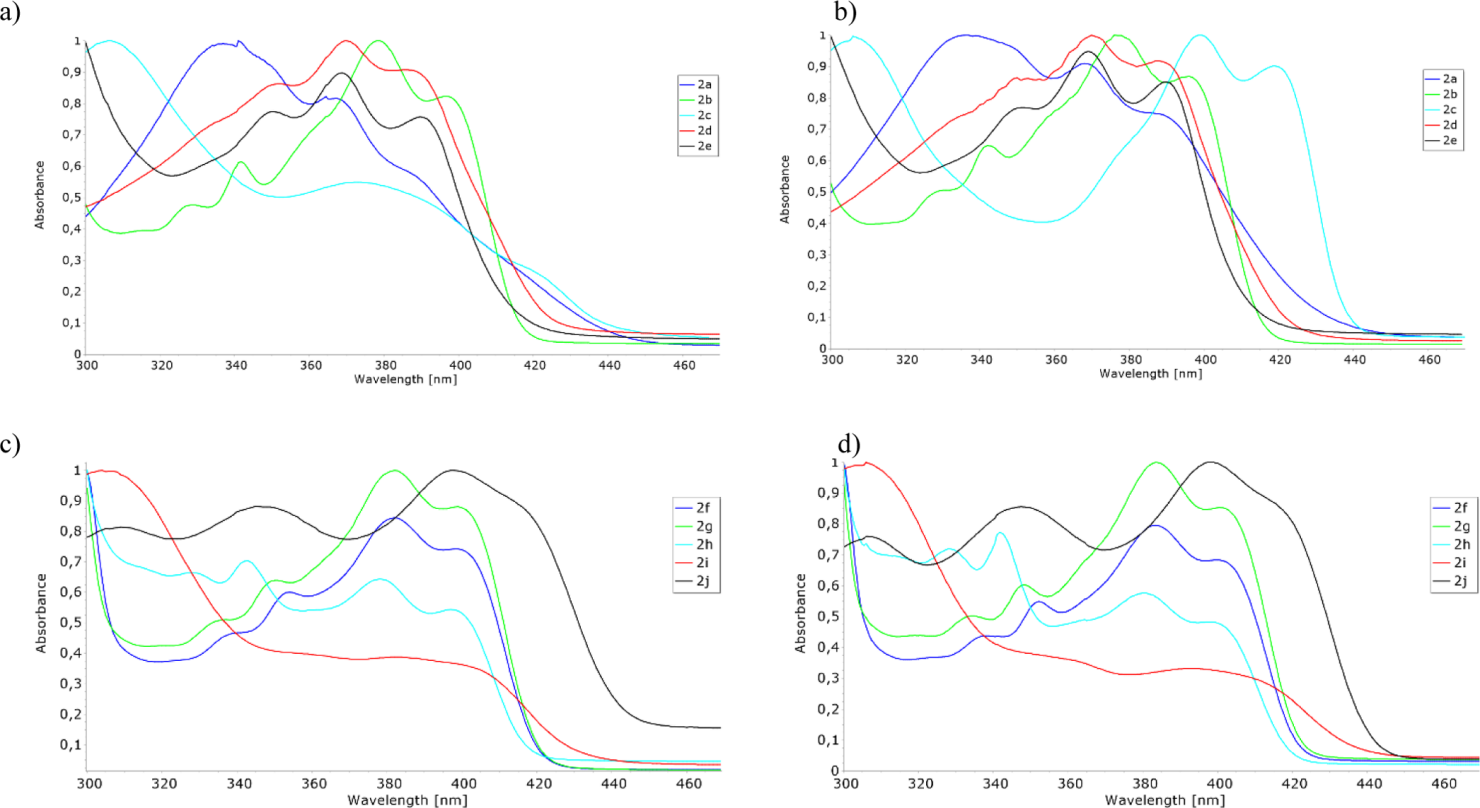
Normalized absorption spectra of (a) **2a–2e** in toluene, (b) **2a–2e** in chloroform, (c) **2f–2j** in toluene, and (d) **2f–2j** in chloroform.

The star-shaped **BDF** derivatives were analyzed using ultraviolet–visible (UV–vis) (Supporting Information) and photoluminescence spectroscopies. Most of the obtained compounds revealed strong fluorescence both in solution and in solid states. Compounds **2b** and **2f–2h** revealed the highest, ∼100% quantum yield (QY) in toluene and chloroform. In addition, all these compounds have almost identical emission spectra profiles and maxima in a very narrow 418.4–419.8 nm range. In toluene, **2a**, **2d**, **2e**, and **2i** (Figure 1) had broad emission with one maximum, while **2b**, **2c**, **2f–2h**, and **2j** had two or three maxima. The latter compounds show structured emission spectra and exhibit no or marginal solvatochromic effect (chloroform vs toluene). Moreover, Stoke’s shift values for these emitters are rather small, 36–43 nm. These observations are typical of molecules with no or very little change in geometry after excitation. On the other hand, compounds **2a**, **2d–e**, and **2i** show structureless emission spectra and exhibit substantial solvatochromism; the bathochromic effect was observed by changing the solvent from toluene to chloroform (Table 5). Stoke’s shifts are much larger (80, 118, 61, and 76 nm for **2a**, **2d**, **2e**, and **2i**, respectively), which are typical for molecules whose excited-state geometry differ substantially from the geometry of the ground state. The elongation of the conjugated phenylene ring system (**2j** vs **2b**) slightly shifted the emission maximum toward longer wavelengths and decreased QY by 15% but did not affect the emission spectrum profile. On analyzing the UV–vis spectra of **2a–2j** in toluene and chloroform, it can be seen that they are rather insensitive to solvent change (Figure 2). We found it interesting, bearing in mind some bathochromic shifts in emission spectra, so we decided to support these observations with computational chemistry methods.

**Table 5:** Physical Properties of **BDFs 2a–2j** in Toluene and Chloroform Solutions

	toluene solution									chloroform solution					
comp.	λ_abs_ (nm)	λ_em_ (nm)	Stoke’s shift (nm)	QY_F_^a^	τ_F_ (ns)	HOMO^b^ (eV)	LUMO^b^ (eV)	*E*_g_^b^ (eV)	*E*_g_^c^ (eV)	λ_abs_ (nm)	toluene to chloroform difference in λ_em_ (nm)	QY_F_^a^	τ_F_ (ns)	HOMO^b^ (eV)	LUMO^b^ (eV)
**2a**	334	414	80	0.36	4.32	–5.37	–1.67	3.10	3.01	336	16 nm bat.^d^	0.25	0.09	–5.38	–1.68
	367									368					
**2b**	379	418	39	1.01	0.87	–4.88	–1.39	2.90	2.86	377	no	1.04	0.84	–4.93	–1.44
	397														
**2c**	399	437	38	0.83	0.91	–5.04	–1.68	2.79	3.01	360	marginal	0.75	0.82	–5.07	–1.67
	419									373					
**2d**	370	488	118	0.06	1.93	–5.22	–1.53	3.12	2.97	370	26 nm bat.	0.04	0.24	–5.24	–1.55
**2e**	369	430	61	0.77	3.11	–5.23	–1.60	3.11	3.04	369	29 nm bat.	0.65	5.11	–5.27	–1.61
**2f**	383	420	37	0.92	0.82	–5.27	–1.61	3.07	2.97	382	marginal	1.09	0.86	–5.29	–1.63
**2g**	384	420	36	0.99	0.82	–5.24	–1.60	3.05	2.98	382	marginal	1.07	0.82	–5.27	–1.62
**2h**	342	419	77	0.95	1.24	–5.40	–1.89	3.03	2.99	343	none	1.07	2.29	–5.41	–1.83
	380									378					
**2i**	393	470	77	0.72	2.58	–4.86	–1.59	2.83	2.88	393	26 nm bat.	0.46	4.76	–4.90	–1.59
**2j**	398	441	43	0.85	0.72	–5.03	–1.57	2.90	2.85	398	marginal	0.94	0.73	–5.08	–1.64

a Values above 1 can occur due to the statistical error of ±0.1 for the QY.

b Calculated at the PBE0/6-31G* level of theory with GD3 empirical dispersion.

c Estimated from the UV–vis spectrum onset.

d bat. = bathochromic.

### Computational Results

Due to the structural flexibility of investigated systems, the first step was identifying the most representative rotamers for which absorption spectra should be calculated. This task was performed with the help of the CREST software,^58,59^ which provides an automated scheme for finding rotamers based on the semiempirical tight-binding GFN2-xTB method^60^ coupled to meta-dynamics simulations.^59^

**Figure 3:**
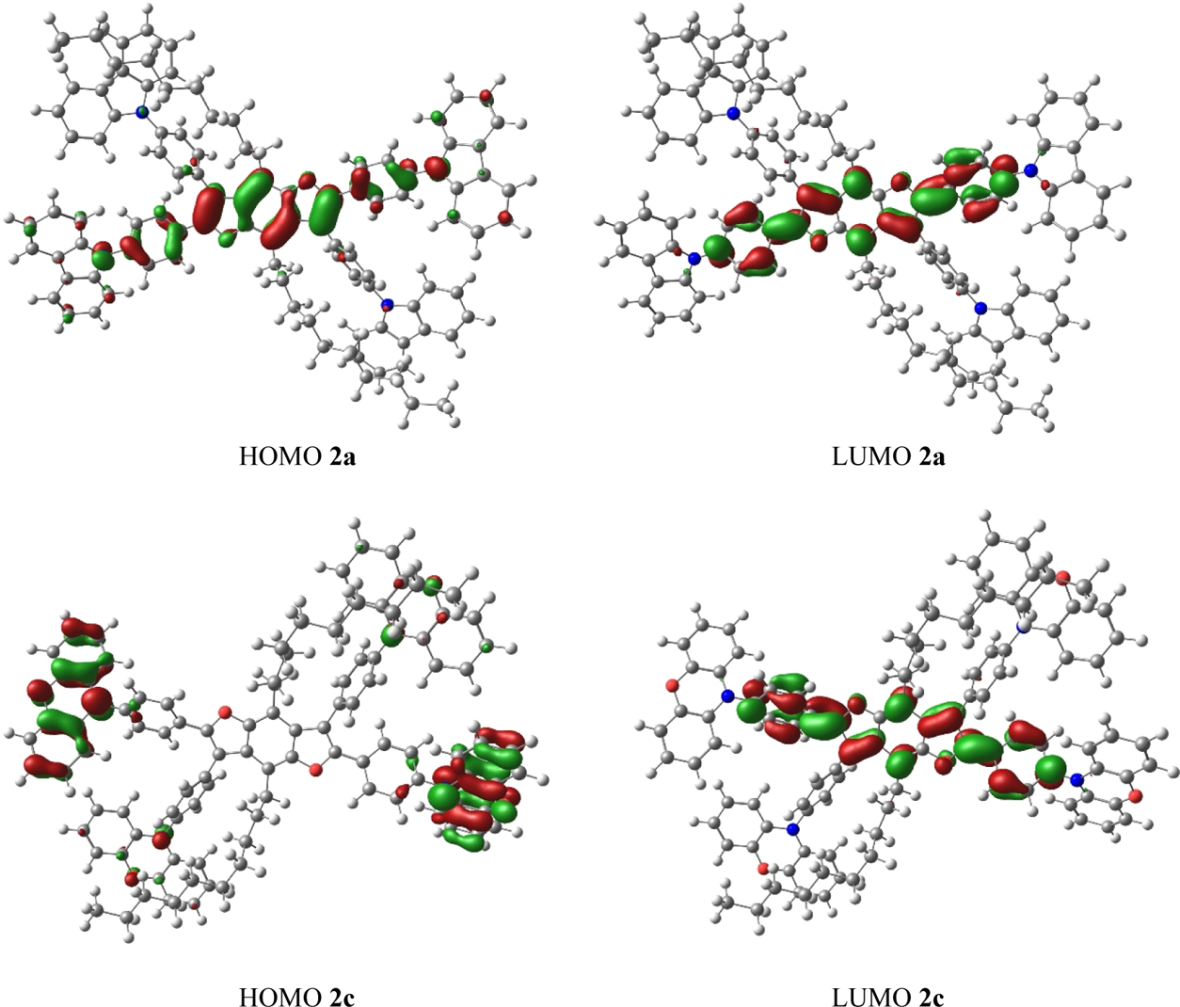
HOMO and LUMO orbitals of **2a** and **2c** calculated within the PBE0/6-31G* level of theory with GD3 empirical dispersion.

At first, in the case of every investigated system, hydrocarbon chains were replaced with methyl groups. Then, CREST calculations were performed, and as a result, the sets of rotamers were obtained. The rotamers with the lowest energy were taken to further calculations: they were enlarged by missing hydrocarbon groups and optimized within the PBE0/6-31G(*) approach with and without Grimme’s GD3 empirical dispersion correction^60^ provided by the Gaussian 19 package. Then, for every investigated system, the UV absorption spectra were recorded using several solvents: chloroform, dichloromethane (DCM), dimethyl sulfoxide (DMSO), tetrahydrofuran (THF), and toluene. Solvents were mimicked by the polarizable continuum model, and the gas-phase geometry of the system was used. All bands for all investigated systems were found to be the π–π*-type transitions. For all systems, the lowest unoccupied molecular orbital (LUMO) orbitals lie on the **BDF** chain (Figures 3 and S23–S32). However, the position of the highest occupied molecular orbital (HOMO) orbitals let us divide investigated molecules into two groups. The first one consists of **2a–b**, **2d**, **2f–g**, and **2j**, where the HOMO orbitals lie on the **BDF** core, and the second is **2c**, **2e**, and **2h–i**, where the HOMO orbitals occupy the outer part of the molecule. One could expect that manifested charge transfer in the second group will exhibit a more significant impact of the solvent on absorption or emission spectra. In Table 6, we compare S0 → S1 excitation wavelengths for different solvents (with toluene as a reference). Indeed, for the second group of molecules, the changes are much more significant. Additionally, the Δδ index,^62^ which is the overall difference of root-mean-square deviation of electron and hole distributions, was calculated in MultiWFn,^63^ for S0 → S1 excitation of the given system in toluene. The Δδ parameter allowed us to quantify the CT for the analyzed star systems. For **2c**, **2e**, and **2h–i**, the absolute value of Δδ (4.46, 4.75, and 6.00, respectively) is much larger than for other systems. It indicates that for the star-shaped benzodifurans and similar systems, Δδ can be a good index for the charge transfer estimation. To provide a simple interpretation of excitation, the natural transition orbitals^61^ have been obtained for the S0 → S1 excitation (the corresponding data are presented in the Supporting Information).

**Table 6:** Increase of the Wavelength [nm] for the First Absorption Line Regarding Toluene

	Δλ^a^				
comp.	THF	chloroform	DCM	DMSO	Δδ
**2a**	2.27	1.43	2.20	2.86	0.95
**2b**	–0.35	–0.37	–0.73	–1.39	1.20
**2c**	9.05	6.23	9.85	13.64	4.46
**2d**	1.82	1.07	1.73	2.37	0.62
**2e**	5.00	3.43	5.49	7.71	4.75
**2f**	2.37	1.52	2.37	3.15	1.63
**2g**	2.29	1.44	2.25	3.01	1.68
**2h**	6.11	4.31	6.42	8.35	3.65
**2i**	8.21	5.53	9.00	13.09	6.00
**2j**	–0.14	–0.23	–0.45	–0.86	1.89

a Δλ = λ_abs,toluene_ – λ_abs,solvent_; calculated at the PBE0/6-31G* level of theory with GD3 empirical dispersion at the geometry for the gas phase. Δδ index calculated for toluene.

## Conclusions

In conclusion, the condition development for palladium-catalyzed amination of bromobenzene with **Cz**, **DPA**, **PXZ**, **PTX**, and **DMAC** was performed. The best catalytic system for palladium-catalyzed coupling with **Cz** proved to be TrixiePhos/*t*-BuOLi/toluene, with **DPA**, **PXZ**, and **PTX**—XPhos/*t*-BuONa/toluene, and with **DMAC**—*t*-BuXPhos/*t*-BuONa/toluene. Based on these results, the Buchwald–Hartwig reaction between the benzodifuran core (**TBBDF**) and the amines mentioned above, and their more extended derivatives, led to desired star-shaped benzodifurans in good to excellent yields. Most of the synthesized compounds revealed an exceptionally good fluorescence in the 400–550 nm range with a QY of up to 100%. TDDFT calculations of absorption spectra showed a small solvatochromic effect with a change in the polarity of the solvent.

## Experimental Section

### General Experimental Methods

Experiments with air- and moisture-sensitive materials were carried under an argon atmosphere. Glassware was oven-dried for several hours, assembled hot, and cooled in a stream of argon. Silica gel 60, Merck 230–400 mesh, was used for preparative column flash chromatography. Analytical thin-layer chromatography (TLC) was performed using Merck TLC silica gel 60 F254 0.2 mm plates. Allylpalladium(II) chloride dimer, other palladium catalysts, phosphines, 4-(diphenylamino)phenylboronic acid pinacol ester, and other commercially available reagents were from Sigma-Aldrich, Merck, or Fluorochem and were used without further purification. 2,3,6,7-Tetrakis(4-bromophenyl)-4,8-didecylbenzo[1,2-*b*:4,5-*b*′]difuran (**TBBDF**),^55^*tert*-butyl-3,6-dibromo-9*H*-carbazole-9-carboxylate (**DBBocCz**),^57^ 3,6-di-*tert*-butyl-9*H*-carbazole,^64^ 3,6-diiodo-9-tosyl-9*H*-carbazole (**DITosCz**),^65^ and 3,6-diiodo-9*H*-carbazole (**DICz**)^65^ were synthesized according to a literature procedure. Solvents were purchased from Avantor, VWR, and Sigma-Aldrich. 1,4-Dioxane, toluene, and THF were distilled from sodium benzophenone ketyl before use. DCM, chloroform, DMSO, acetone, methanol, diethyl ether, and ethyl acetate were dried with molecular sieves and used without further purification. ^1^H and ^13^C NMR spectra were recorded on a Bruker Avance III 400 MHz or Bruker Avance III 700 MHz instrument at ambient temperature. Chemical shifts are reported in parts per million (δ scale), and coupling constants (*J* values) are listed in hertz. Structural assignments were made with additional information from gCOSY, gHSQC, and gHMBC experiments. GC–MS analyses were performed on a Shimadzu GCMS-TQ8040 system, and detector response was calibrated on substrate and product standards. IR spectra were recorded on a PerkinElmer Spectrum Two FT-IR Spectrometer. The melting points were determined with a Büchi SMP 32 and Barnstead Thermolyne Mel-Temp II apparatus in open capillaries and are uncorrected. Elemental analyses were performed using an Elementar Analysensysteme GmbH Vario MACRO CHNanalyzer.

### Spectroscopic Measurements

Toluene and chloroform (spectrometric grade from Merck) were employed as solvents for absorption and fluorescence measurements. UV–vis absorption spectra were recorded on a PerkinElmer UV–vis Lambda 25 spectrometer in a 1 cm quartz cell compared to solvent blank. Emission spectra were obtained on a JASCO FP-8500 spectrometer. The QY was determined using 9,10-diphenylanthracene in toluene (θ_ref_ = 0.95) at λ_ex_ = 366 nm. The concentration of **9,10-diphenylanthracene** and the analyzed substances were set so that the absorbance at 366 nm was low enough to avoid the inner filter effect. The fluorescence lifetime was determined using a time-correlated single-photon counting setup with a Maestro spectrum analyzer (EG&G Ortec, Oak Ridge, USA) and a pulsed LED (376 nm, PicoQuant GmbH, Berlin, Germany) with a pulse width of fewer than 1.5 ns (full width at half-maximum). All fluorescence light above 406 nm was detected using a low-pass filter. The decay traces were analyzed assuming a single exponential decay function.

### Amination Screening

In a pressure vial closed with a septum, an amine (1.62 mmol) and a base (1.62 mmol) were mixed in a dry solvent (4 mL) and stirred for 5 min under argon. In a separate vial, [Pd(allyl)Cl]_2_ (0.016 mmol; 5.9 mg; 1 mol %) and phosphine ligand (0.065 mmol; 4 mol %) in a dry solvent (1.5 mL) were stirred under argon for 5 min. Bromobenzene (1.62 mmol) and the catalyst mixture were added to the pressure vial, the septum was replaced by a screw cap, the vial was immersed in an oil bath, and the mixture was stirred at 100 °C for 24 h. After this time, the reaction mixture was cooled to room temperature and analyzed by GC–MS.

### Synthesis of **BDF** Derivatives

#### 9,9′,9″,9‴-((4,8-Didecylbenzo[1,2-*b*:4,5-*b*′]difuran-2,3,6,7-tetrayl)tetrakis(benzene-4,1-diyl))tetrakis(9*H*-carbazole) (**2a**)

In a 10 mL vial, [Pd(allyl)Cl]_2_ (0.006 mmol; 2.2 mg; 4 mol %) and TrixiePhos (0.024 mmol; 9.6 mg; 16 mol %) were mixed in dry, degassed toluene (3 mL) and stirred for 10 min in an inert gas atmosphere. In a Schlenk flask, 9*H*-carbazole (0.9 mmol; 150 mg) was dissolved in dry, degassed toluene (10 mL), *t*-BuONa (0.9 mmol; 86 mg) was added, and the mixture was stirred for 5 min under argon. 2,3,6,7-Tetrakis(4-bromophenyl)-4,8-didecylbenzo[1,2-*b*:4,5-*b*′]difuran (**TBBDF**) (0.15 mmol; 158 mg) was added, followed by the catalyst mixture, and the flask was immersed in an oil bath preheated to 100 °C and stirred at this temperature for 24 h. It was cooled to room temperature and poured into MeOH (40 mL). The precipitate was filtered off; washed with H_2_O (5 mL), MeOH (2 × 5 mL), and Et_2_O (2 mL); and dried to afford 191 mg of light beige solid (90%). Although, NMR analysis revealed high purity of the desired product, flash chromatography, AcOEt/PE 2:8, was performed, but no changes in purity and product mass were observed. ^1^H NMR (CDCl_3_, 700 MHz): δ [ppm] 8.20 (d, *J* = 7.7 Hz, 4H, 4CH_Ar_), 8.15 (d, *J* = 7.8 Hz, 4H, 4CH_Ar_), 7.91–7.85 (m, 8H, 8CH_Ar_), 7.84–7.80 (m, 4H, 4CH_Ar_), 7.61–7.55 (m, 8H, 8CH_Ar_), 7.51–7.46 (m, 8H, 8CH_Ar_), 7.42 (t, *J* = 7.5 Hz, 4H, 4CH_Ar_), 7.34 (t, *J* = 7.4 Hz, 4H, 4CH_Ar_), 7.31–7.29 (t, *J* = 7.5 Hz, 4H, 4CH_Ar_), 3.00–2.94 (m, 4H, 2CH_2_), 1.73–1.69 (m, 4H, 2CH_2_), 1.34–1.10 (m, 28H, 14CH_2_), 0.79 (t, *J* = 7.2 Hz, 6H, 2CH_3_). ^13^C{^1^H} NMR (100 MHz, CDCl_3_): δ [ppm] 150.0 (2C_Ar_), 140.8 (4C_Ar_), 140.6 (4C_Ar_), 137.8 (2C_Ar_), 137.4 (4C_Ar_), 133.8 (2C_Ar_), 132.4 (4CH_Ar_), 129.9 (2C_Ar_), 127.7 (4CH_Ar_), 127.6 (4CH_Ar_), 126.9 (4CH_Ar_), 126.1 (4CH_Ar_), 126.0 (4CH_Ar_), 123.6 (2C_Ar_), 123.6 (2C_Ar_), 120.5 (4CH_Ar_), 120.4 (4CH_Ar_), 120.2 (4CH_Ar_), 120.2 (4CH_Ar_), 118.4 (2C_Ar_), 116.4 (8C_Ar_), 109.8 (4CH_Ar_), 109.7 (4CH_Ar_), 31.8 (2CH_2_), 31.2 (2CH_2_), 30.3 (2CH_2_), 29.7 (2CH_2_), 29.7 (2CH_2_), 29.6 (2CH_2_), 29.4 (2CH_2_), 25.9 (2CH_2_), 22.6 (2CH_2_), 14.0 (2CH_3_). IR ν_max_ cm^–1^: 1451 (s), 1228 (s), 747 (s). mp: 309–310 °C. Anal. Calcd for C_102_H_90_N_4_O_2_: C, 87.27; H, 6.46; N, 3.99; O, 2.28. Found: C, 87.60; H, 6.42; N, 4.01; O, 2.32.

#### 4,4′,4″,4‴-(4,8-Didecylbenzo[1,2-*b*:4,5-*b*′]difuran-2,3,6,7-tetrayl)tetrakis(*N*,*N*-diphenylaniline) (**2b**)

This compound was prepared according to the procedure for **2a** using XPhos (0.024 mmol; 11.5 mg; 16 mol %) and diphenylamine (0.9 mmol; 152 mg) to obtain 196 mg of light green solid (92%). ^1^H NMR (CDCl_3_, 700 MHz): δ [ppm] 7.49 (d, *J* = 8.8 Hz, 4H, 4CH_Ar_), 7.38 (d, *J* = 8.5 Hz, 4H, 4CH_Ar_), 7.32–7.27 (m, 16H, 16CH_Ar_), 7.22–7.18 (m, 12H, 12CH_Ar_), 7.15 (d, *J* = 8.4 Hz, 8H, 8CH_Ar_), 7.10–7.05 (m, 8H, 8CH_Ar_), 7.00 (d, *J* = 8.9 Hz, 4H, 4CH_Ar_), 2.82–2.75 (m, 4H, 2CH_2_), 1.56–1.52 (m, 4H, 2CH_2_), 1.29–1.19 (m, 28H, 14CH_2_), 0.86 (t, *J* = 7.2 Hz, 6H, 2CH_3_). ^13^C{^1^H} NMR (175 MHz, CDCl_3_): δ [ppm] 149.6 (2C_Ar_), 147.8 (6C_Ar_), 147.5 (6C_Ar_), 147.3 (4C_Ar_), 131.7 (4CH_Ar_), 129.3 (16C_Ar_), 127.0 (4CH_Ar_), 125.9 (2C_Ar_), 125.2 (2C_Ar_), 124.8 (8C_Ar_), 124.6 (8CH_Ar_), 123.6 (4CH_Ar_), 123.3 (4CH_Ar_), 123.0 (4CH_Ar_), 122.6 (4CH_Ar_), 115.6 (4C_Ar_), 31.9 (2CH_2_), 30.9 (2CH_2_), 30.1 (2CH_2_), 29.7 (2CH_2_), 29.6 (2CH_2_), 29.5 (2CH_2_), 29.4 (2CH_2_), 25.6 (2CH_2_), 22.6 (2CH_2_), 14.0 (2CH_3_). IR ν_max_ cm^–1^: 1460 (s), 740 (s). mp: 211–212 °C. Anal. Calcd for C_102_H_98_N_4_O_2_: C, 86.77; H, 7.00; N, 3.97; O, 2.27. Found: C, 86.34; H, 7.04; N, 3.99; O, 2.17.

#### 10,10′,10″,10‴-((4,8-Didecylbenzo[1,2-*b*:4,5-*b*′]difuran-2,3,6,7-tetrayl)tetrakis(benzene-4,1-diyl))tetrakis(10*H*-phenoxazine) (**2c**)

This compound was prepared according to the procedure for **2a** using XPhos (0.024 mmol; 11.5 mg; 16 mol %) and 10*H*-phenoxazine (0.9 mmol; 165 mg) to obtain 196 mg of light green solid (92%). ^1^H NMR (CDCl_3_, 700 MHz): δ [ppm] 7.86–7.82 (m, 4H, 4CH_Ar_), 7.80–7.75 (m, 4H, 4CH_Ar_), 7.60–7.55 (m, 4H, 4CH_Ar_), 7.31–7.27 (m, 4H, 4CH_Ar_), 6.76–6.57 (m, 24H, 24CH_Ar_), 6.11–6.06 (m, 4H, 4CH_Ar_), 6.01–5.95 (m, 4H, 4CH_Ar_), 2.88–2.83 (m, 4H, 2CH_2_), 1.66–1.59 (m, 4H, 2CH_2_), 1.33–1.16 (m, 28H, 14CH_2_), 0.82 (t, *J* = 7.2 Hz, 6H, 2CH_3_). ^13^C{^1^H} NMR (100 MHz, CDCl_3_): δ [ppm] 150.0 (2C_Ar_), 144.1 (2C_Ar_), 144.0 (2C_Ar_), 139.1 (2C_Ar_), 138.7 (2C_Ar_), 135.2 (2C_Ar_), 134.2 (2C_Ar_), 134.1 (4C_Ar_), 133.5 (4CH_Ar_), 131.8 (4CH_Ar_), 131.0 (2C_Ar_), 130.9 (4CH_Ar_), 128.6 (4CH_Ar_), 126.1 (4C_Ar_), 123.3 (4CH_Ar_), 123.3 (4CH_Ar_), 121.7 (4CH_Ar_), 121.5(4CH_Ar_), 118.6 (2C_Ar_), 116.4 (8C_Ar_), 115.7 (4CH_Ar_), 115.6 (4CH_Ar_), 113.2 (4CH_Ar_), 113.1 (4CH_Ar_), 32.0 (2CH_2_), 31.0 (2CH_2_), 30.0 (2CH_2_), 29.7 (2CH_2_), 29.6 (2CH_2_), 29.5 (2CH_2_), 29.4 (2CH_2_), 25.8 (2CH_2_), 22.7 (2CH_2_), 14.1 (2CH_3_). IR ν_max_ cm^–1^: 1485 (s), 1269 (s), 739 (s). mp: 307–308 °C. Anal. Calcd for C_102_H_90_N_4_O_6_: C, 83.46; H, 6.18; N, 3.82; O, 6.54. Found: C, 83.34; H, 6.13; N, 3.79; O, 6.58.

#### 10,10′,10″,10‴-((4,8-Didecylbenzo[1,2-*b*:4,5-*b*′]difuran-2,3,6,7-tetrayl)tetrakis(benzene-4,1-diyl))tetrakis(10*H*-phenothiazine) (**2d**)

This compound was prepared according to the procedure for **2a** using XPhos (0.024 mmol; 11.5 mg; 16 mol %) and 10*H*-phenothiazine (0.9 mmol; 179 mg) to obtain 166 mg of light green solid (75%). ^1^H NMR (CDCl_3_, 700 MHz): δ [ppm] 7.83 (d, *J* = 8.3 Hz, 4H, 4CH_Ar_), 7.74 (d, *J* = 8.6 Hz, 4H, 4CH_Ar_), 7.60 (d, *J* = 8.3 Hz, 4H, 4CH_Ar_), 7.30–7.27 (m, 4H, 4CH_Ar_), 7.13 (dd, *J* = 7.5, 1.6 Hz, 8H, 8CH_Ar_), 6.98–6.94 (m, 8H, 8CH_Ar_), 6.92–6.89 (m, 8H, 8CH_Ar_), 6.52–6.48 (m, 8H, 8CH_Ar_), 2.92–2.88 (m, 4H, 2CH_2_), 1.67–1.62 (m, 4H, 2CH_2_), 1.32–1.20 (m, 28H, 14CH_2_), 0.86 (t, *J* = 7.2 Hz, 6H, 2CH_3_). ^13^C{^1^H} NMR (175 MHz, CDCl_3_): δ [ppm] 150.2 (2C_Ar_), 149.9 (2C_Ar_), 143.9 (4C_Ar_), 143.6 (4C_Ar_), 141.7 (2C_Ar_), 141.5 (2C_Ar_), 134.4 (2C_Ar_), 133.1 (4CH_Ar_), 130.5 (4CH_Ar_), 129.5 (2C_Ar_), 128.2 (4CH_Ar_), 127.9 (4CH_Ar_), 127.2 (4CH_Ar_), 127.1 (4CH_Ar_), 126.9 (8CH_Ar_), 126.0 (2C_Ar_), 123.2 (4CH_Ar_), 123.1 (4C_Ar_), 122.9 (4CH_Ar_), 121.8 (4C_Ar_), 118.2 (2C_Ar_), 118.1 (4CH_Ar_), 116.9 (4CH_Ar_), 116.2 (2C_Ar_), 31.8 (2CH_2_), 31.0 (2CH_2_), 29.9 (2CH_2_), 29.6 (2CH_2_), 29.5 (4CH_2_), 29.3 (2CH_2_), 25.8 (2CH_2_), 22.6 (2CH_2_), 14.0 (2CH_3_). IR ν_max_ cm^–1^: 1466 (s), 1229 (s), 723 (s). mp: 307–308 °C. Anal. Calcd for C_102_H_90_N_4_O_2_S_4_: C, 79.96; H, 5.92; N, 3.66; O, 2.09; S, 8.37. Found: C, 80.39; H, 5.97; N, 3.53; O, 2.12; S, 8.35.

#### 10,10′,10″,10‴-((4,8-Didecylbenzo[1,2-*b*:4,5-*b*′]difuran-2,3,6,7-tetrayl)tetrakis(benzene-4,1-diyl))tetrakis(9,9-dimethyl-9,10-dihydroacridine) (**2e**)

This compound was prepared according to the procedure for **2a** using *t*-BuXPhos (0.024 mmol; 10.2 mg; 16 mol %) and **DMAC** (0.9 mmol; 188 mg). After 24 h, the reaction mixture was cooled to RT and transferred into a separatory funnel. DCM (20 mL) and water (50 mL) were added, and the layers were separated. The aqueous layer was extracted with DCM (3 × 20 mL). Combined organic layers were dried over anhydrous magnesium sulfate and concentrated. Purification was done by flash chromatography using DCM/PE (1:1) as an eluent to obtain 61 mg of light green solid (19%). ^1^H NMR (CDCl_3_, 700 MHz): δ [ppm] 7.95–7.90 (m, 8H, 8CH_Ar_), 7.61 (d, *J* = 8.3 Hz, 4H, 4CH_Ar_), 7.53–7.49 (m, 8H, 8CH_Ar_), 7.34 (d, *J* = 8.6 Hz, 4H, 4CH_Ar_), 7.05–6.95 (m, 16H, 16CH_Ar_), 6.51 (dd, *J* = 8.1, 1.1 Hz, 4H, 4CH_Ar_), 6.38 (dd, *J* = 8.2, 1.12 Hz, 4CH_Ar_), 3.00–2.98 (m, 4H, 2CH_2_), 1.75 (s, 12H, 4CH_3_), 1.73 (s, 12H, 4CH_3_), 1.37–1.16 (m, 32H, 16CH_2_), 0.82 (t, *J* = 7.2 Hz, 6H, 2CH_3_). ^13^C{^1^H} NMR (175 MHz, CDCl_3_): δ [ppm] 150.2 (2C_Ar_), 150.1 (2C_Ar_), 144.4 (2C_Ar_), 141.1 (2C_Ar_), 140.8 (8C_Ar_), 135.0 (2C_Ar_), 133.4 (4CH_Ar_), 132.3 (4CH_Ar_), 131.3 (4CH_Ar_), 130.8 (2C_Ar_), 130.4 (4C_Ar_), 130.3 (4C_Ar_), 128.5 (4CH_Ar_), 126.4 (4CH_Ar_), 126.4 (4CH_Ar_), 126.2 (2C_Ar_), 125.3 (4CH_Ar_), 125.2 (4CH_Ar_), 120.8 (4CH_Ar_), 120.8 (4CH_Ar_), 118.8 (2C_Ar_), 116.4 (2C_Ar_), 114.1 (4CH_Ar_), 114.0 (4CH_Ar_), 36.1 (2C), 36.0 (2C), 31.8 (2CH_2_), 31.1 (8CH_3_), 31.0 (2CH_2_), 29.9 (2CH_2_), 29.6 (2CH_2_), 29.6 (2CH_2_), 29.5 (2CH_2_), 29.3 (2CH_2_), 25.8 (2CH_2_), 22.6 (2CH_2_), 14.0 (2CH_3_). IR ν_max_ cm^–1^: 1466 (s), 1268 (s), 742 (s). mp: 285–288 °C. Anal. Calcd for C_114_H_114_N_4_O_2_: C, 87.09; H, 7.31; N, 3.56; O, 2.04. Found: C, 87.00; H, 7.27; N, 3.58; O, 2.09.

#### 3,6-Di(dec-1-yn-1-yl)-9*H*-carbazole

In a round-bottom flask, 3,6-diiodocarbazole (**DICz**) (20 mmol; 8.38 g), Pd(dppf)Cl_2_ (0.27 mmol; 0.198 g), and CuI (0.54 mmol; 0.103 g) were dissolved in dry, degassed toluene (50 mL). Then, *i*-Pr_2_NH (80 mmol; 11.2 mL) was added, and after 5 min, 1-decyne (50 mmol; 6.91 g; 9 mL) was added dropwise. The flask was immersed in an oil bath at 70 °C for 3 h. The reaction mixture was then cooled to RT, and water (200 mL) was added. The solution was extracted with ethyl acetate (3 × 60 mL) and then washed with citric acid, NaHCO_3_, water, and brine. The extract was dried over anhydrous MgSO_4_ and concentrated to afford 9.77 g of the product that was taken into the next step without purification. ^1^H NMR (CDCl_3_, 700 MHz): δ [ppm] 8.11 (s, 2H, 2CH_Ar_), 8.08 (s, 1H, NH), 7.48 (dd, *J* = 8.3, 1.6 Hz, 2H, 2CH_Ar_), 7.32 (d, *J* = 8.3 Hz, 2H, 2CH_Ar_), 2.48 (t, *J* = 7.2 Hz, 4H, 2CH_2_), 1.70–1.64 (m, 4H, 2CH_2_), 1.54–1.49 (m, 4H, 2CH_2_), 1.41–1.29 (m, 16H, 8CH_2_), 0.93 (t, *J* = 7.0 Hz, 6H, 2CH_3_). ^13^C{^1^H} NMR (175 MHz, CDCl_3_): δ [ppm] 139.0 (2C_Ar_), 129.8 (2CH_Ar_), 123.8 (2CH_Ar_), 123.0 (2C_Ar_), 115.5 (2C_Ar_), 110.5 (2CH_Ar_), 88.4 (2C_alk_), 81.3 (2C_alk_), 31.9 (2CH_2_), 29.2 (2CH_2_), 29.2 (2CH_2_), 29.0 (4CH_2_), 22.6 (2CH_2_), 19.5 (2CH_2_), 14.0 (2CH_3_). IR ν_max_ cm^–1^: 1469 (s), 1254 (s), 714 (m). mp: 75–76 °C. Anal. Calcd for C_32_H_41_N: C, 87.41; H, 9.40; N, 3.19. Found: C, 87.87; H, 9.41; N, 3.14.

#### 3,6-Didecyl-9*H*-carbazole (**1a**)

In a round-bottom flask, 3,6-di(dec-1-yn-1-yl)-9*H*-carbazole (20.0 mmol; 8.80 g) was dissolved in degassed ethyl acetate (100 mL) under nitrogen at RT. Then, Pd/C (10% w/w; 1.20 g) was added and stirred at 50 °C under an atmosphere of hydrogen. After 12 h, the mixture was cooled to RT and filtered over Celite, washed with ethyl acetate, and evaporated to dryness on a rotary evaporator. The crude product was purified by flash chromatography using EA/PE (1:9) as an eluent. The product was obtained as a light beige solid, 5.60 g (64%). ^1^H NMR (CDCl_3_, 700 MHz): δ [ppm] 7.88 (s, 2H, 2CH_Ar_), 7.86 (s, 1H, NH), 7.33 (d, *J* = 8.2 Hz, 2H, 2CH_Ar_), 7.24 (dd, *J* = 8.2, 1.6 Hz, 2H, 2CH_Ar_), 2.80 (t, *J* = 7.8 Hz, 4H, 2CH_2_), 1.77–1.69 (m, 4H, 2CH_2_), 1.43–1.25 (m, 28H, 9CH_2_), 0.91 (t, *J* = 7.1 Hz, 6H, 2CH_3_). ^13^C{^1^H} NMR (175 MHz, CDCl_3_): δ [ppm] 138.3 (2C_Ar_), 133.9 (2C_Ar_), 126.4 (2CH_Ar_), 123.5 (2C_Ar_), 119.5 (2CH_Ar_), 110.2 (2CH_Ar_), 36.1(2CH_2_), 32.2 (2CH_2_), 31.9 (2CH_2_), 29.6 (4CH_2_), 29.4 (2CH_2_), 29.3 (2CH_2_), 22.7 (2CH_2_), 14.1 (2CH_3_). IR ν_max_ cm^–1^: 1248 (m), 723 (m). mp: 62–63 °C. Anal. Calcd for C_32_H_49_N: C, 85.84; H, 11.03; N, 3.13. Found: C, 85.43; H, 11.12; N, 3.01.

#### 9,9′,9″,9‴-((4,8-Didecylbenzo[1,2-*b*:4,5-*b*′]difuran-2,3,6,7-tetrayl)tetrakis(benzene-4,1-diyl))tetrakis(3,6-didecyl-9*H*-carbazole) (**2f**)

This compound was prepared according to the procedure for **2a** except **TBBDF** (0.25 mmol; 264 mg), 3,6-didecylcarbazole (1.5 mmol; 672 mg), [Pd(allyl)Cl]_2_ (0.02 mmol; 7.3 mg; 8 mol %), *t*-BuXPhos (0.08 mmol; 29.3 mg; 32 mol %), and *t*-BuONa (1.5 mmol; 144 mg). Product as a light green solid, 386 mg (61%). ^1^H NMR (CDCl_3_, 700 MHz): δ [ppm] 7.98 (d, *J* = 1.1 Hz, 4H, 4CH_Ar_), 7.93 (d, *J* = 1.2 Hz, 4H, 4CH_Ar_), 7.88–7.81 (m, 12H, 12CH_Ar_), 7.59 (d, *J* = 8.6 Hz, 4H, 4CH_Ar_), 7.49 (d, *J* = 8.3 Hz, 4H, 4CH_Ar_), 7.42 (d, *J* = 8.3 Hz, 4H, 4CH_Ar_), 7.30 (dd, *J* = 8.4, 1.5 Hz, 4H, 4CH_Ar_), 7.24 (dd, *J* = 8.4, 1.6 Hz, 4H, 4CH_Ar_), 3.01–2.94 (m, 4H, 2CH_2_), 2.86–2.79 (m, 16H, 8CH_2_), 1.79–1.70 (m, 20H, 10CH_2_), 1.46–1.14 (s, 140H, 70CH_2_), 0.92–0.89 (td, *J* = 7.1, 1.8 Hz, 24H, 8CH_3_), 0.82 (t, *J* = 7.2 Hz, 6H, 2CH_3_). ^13^C{^1^H} NMR (175 MHz, CDCl_3_): δ [ppm] 150.4 (2C_Ar_), 150.1 (2C_Ar_), 139.5 (4C_Ar_), 139.3 (4C_Ar_), 138.3 (2C_Ar_), 138.0 (2C_Ar_), 134.8 (4C_Ar_), 134.7 (4C_Ar_), 133.4 (2C_Ar_), 132.3 (4CH_Ar_), 129.5 (2C_Ar_), 127.7 (4CH_Ar_), 127.3 (4CH_Ar_), 126.6 (4CH_Ar_), 126.6 (4CH_Ar_), 126.5 (4CH_Ar_), 126.1 (2C_Ar_), 123.8 (4CH_Ar_), 123.7 (4C_Ar_), 119.7 (4CH_Ar_), 119.6 (4CH_Ar_), 118.3 (2CH_Ar_), 116.2 (2CH_Ar_), 109.5 (4CH_Ar_), 109.3 (4CH_Ar_), 34.8 (4CH_2_), 36.0 (4CH_2_), 32.3 (4CH_2_), 32.2 (4CH_2_), 31.9 (8CH_2_), 31.8 (2CH_2_), 31.2 (2CH_2_), 30.3 (2CH_2_), 29.7 (2CH_2_), 29.7 (2CH_2_), 29.6 (8CH_2_), 29.6 (10CH_2_), 29.6 (4CH_2_), 29.6 (4CH_2_), 29.4 (6CH_2_), 29.4 (4CH_2_), 29.3 (10CH_2_), 22.6 (8CH_2_), 22.6 (2CH_2_), 14.0 (8CH_3_), 14.0 (2CH_3_). IR ν_max_ cm^–1^: 1462 (s), 1232.10 (m). mp: 216–217 °C. Anal. Calcd for C_182_H_250_N_4_O_2_: C, 86.54; H, 9.98; N, 2.22; O, 1.27. Found: C, 86.21; H, 9.86; N, 2.27; O, 1.34.

#### 9,9′,9″,9‴-((4,8-Didecylbenzo[1,2-*b*:4,5-*b*′]difuran-2,3,6,7-tetrayl)tetrakis([1,1′-biphenyl]-4′,4-diyl))tetrakis(3,6-di-*tert*-butyl-9*H*-carbazole) (**2g**)

This compound was prepared according to the procedure for **2a** except **TBBDF** (0.25 mmol; 264 mg), 3,6-di-*tert*-butyl-9*H*-carbazole (1.5 mmol; 418 mg), [Pd(allyl)Cl]_2_ (0.02 mmol; 7.3 mg; 8 mol %), *t*-BuXPhos (0.08 mmol; 29.3 mg; 32 mol %), and *t*-BuONa (1.5 mmol; 144 mg). After 24 h, the reaction mixture was cooled to RT and transferred into a separatory funnel. DCM (20 mL) and water (50 mL) were added, and the layers were separated. An aqueous layer was extracted with DCM (3 × 20 mL). The combined organic layers were dried over anhydrous magnesium sulfate and concentrated. The crude product was stirred in MeOH (50 mL) at 65 °C for 0.5 h, and the solid phase was filtered off. Then, the brown solid was stirred in ethyl acetate (50 mL) at 60 °C for 1 h, and the solid phase was filtered off and dried to obtain the product as a beige light solid, 288 mg (62%). ^1^H NMR (CDCl_3_, 700 MHz): δ [ppm] 8.21 (d, *J* = 1.8 Hz, 4H, 4CH_Ar_), 8.17 (d, *J* = 1.9 Hz, 4H, 4CH_Ar_), 7.89–7.87 (m, 4H, 4CH_Ar_), 7.85–7.81 (m, 8H, 8CH_Ar_), 7.60 (d, *J* = 8.6 Hz, 4H, 4CH_Ar_), 7.57–7.55 (m, 4H, 4CH_Ar_), 7.53–7.49 (m, 8H, 8CH_Ar_), 7.47–7.45 (m, 4H, 4CH_Ar_), 3.02–2.97 (m, 4H, 2CH_2_), 1.74–1.68 (m, 4H, 2CH_2_), 1.52 (s, 36H, 12CH_3_), 1.50 (s, 36H, 12CH_3_), 1.27–1.16 (m, 28H, 14CH_2_), 0.82 (t, *J* = 7.2 Hz, 6H, 2CH_3_). ^13^C{^1^H} NMR (175 MHz, CDCl_3_): δ [ppm] 150.5 (2C_Ar_), 150.1 (2C_Ar_), 143.2 (8C_Ar_), 139.2 (8C_Ar_), 139.0 (8C_Ar_), 138.3 (2C_Ar_), 138.0 (2C_Ar_), 133.5 (2C_Ar_), 132.3 (4CH_Ar_), 129.5 (2C_Ar_), 127.7 (4CH_Ar_), 127.2 (4CH_Ar_), 126.4 (4CH_Ar_), 126.1 (2C_Ar_), 123.8 (4CH_Ar_), 123.7 (4CH_Ar_), 123.6 (2C_Ar_), 118.4 (2C_Ar_), 116.3 (4CH_Ar_), 116.2 (4CH_Ar_), 109.4 (4CH_Ar_), 109.2 (4CH_Ar_), 34.8 (4C), 34.7 (4C), 32.0 (12CH_3_), 32.0 (12CH_3_), 31.9 (2CH_2_), 31.2 (2CH_2_), 30.3(2CH_2_), 29.8 (2CH_2_), 29.7 (2CH_2_), 29.6 (2CH_2_), 29.4 (2CH_2_), 25.9 (2CH_2_), 22.6 (2CH_2_), 14.0 (2CH_3_). IR ν_max_ cm^–1^: 1473 (s). mp: >400 °C. Anal. Calcd for C_134_H_154_N_4_O_2_: C, 86.87; H, 8.38; N, 3.02; O, 1.73. Found: C, 86.68; H, 8.45; N, 2.99; O, 1.76.

#### 9′-Tosyl-9′*H*-9,3’:6′,9″-tercarbazole^56^

In a 10 mL vial, [Pd(allyl)Cl]_2_ (0.2 mmol; 73.2 mg; 1 mol %) and *t*-BuXPhos (0.8 mmol; 340 mg; 4 mol %) were mixed in dry, degassed 1,4-dioxane (5 mL) and stirred for 5 min in an inert gas atmosphere. In a round-bottom flask, 9*H*-carbazole (42 mmol; 7.014 g) was dissolved in dry, degassed 1,4-dioxane (130 mL), *t*-BuOLi (1 M in THF, 42 mmol; 42 mL) was added, and the mixture was stirred for 5 min under argon. 3,6-Diiodo-9-tosyl-9*H*-carbazole (**DITosCz**) (20 mmol; 11.460 g) was added, followed by the catalyst mixture, and the flask was immersed in an oil bath preheated to 100 °C and stirred at this temperature for 24 h. It was cooled to room temperature and poured into MeOH (250 mL). The precipitate was filtered off; washed with H_2_O (25 mL), MeOH (2 × 50 mL), and Et_2_O (20 mL); and dried to afford 8.925 g of light beige solid (68%). However, NMR analysis revealed high purity of the desired product; flash chromatography, DCM/PE 1:1, was performed, but no changes in purity and product mass were observed. ^1^H NMR (CDCl_3_, 700 MHz): δ [ppm] 8.64 (d, *J* = 8.9 Hz, 2H, 2CH_Ar_), 8.17 (d, *J* = 7.6 Hz, 4H, 4CH_Ar_), 8.12 (d, *J* = 2.1 Hz, 2H, 2CH_Ar_), 7.97 (d, *J* = 8.4 Hz, 2H, 2CH_Ar_), 7.76 (dd, *J* = 8.9, 2.1 Hz, 2H, 2CH_Ar_), 7.38–7.35 (m, 8H, 8CH_Ar_), 7.33–7.27 (m, 6H, 6CH_Ar_), 2.40 (s, 3H, CH_3_). ^13^C{^1^H} NMR (175 MHz, CDCl_3_): δ [ppm] 145.5 (C_Ar_), 141.3 (4C_Ar_), 137.8 (2C_Ar_), 135.2 (C_Ar_), 134.1 (2C_Ar_), 130.1 (2CH_Ar_), 127.2 (2CH_Ar_), 127.1 (2C_Ar_), 126.8 (2CH_Ar_), 126.0 (4CH_Ar_), 123.5 (4C_Ar_), 120.4 (4CH_Ar_), 120.1 (4CH_Ar_), 119.1 (2CH_Ar_), 116.4 (2CH_Ar_), 109.5 (4CH_Ar_), 21.6 (CH_3_). IR ν_max_ cm^–1^: 1452 (s), 1228 (s), 748 (s). mp: 306–311 °C.

#### 9′*H*-9,3′:6′,9″-Tercarbazole (**1c**)^56^

In a round-bottom flask, 9′-tosyl-9′*H*-9.3′:6′,9″-tercarbazole (13.5 mmol; 8.925 g) was dissolved in the mixture of THF (30 mL), DMSO (15 mL), and water (5 mL) and stirred for 10 min. Then, KOH (164 mmol; 9.20 g) was added. The reaction was stirred for 18 h under reflux. After this time, the reaction was cooled to room temperature, and H_2_O (15 mL) was added, followed by neutralization with aqueous HCl (2 M). The resulting precipitate was filtered off; then washed with H_2_O (50 mL), MeOH (2 × 100 mL), and Et_2_O (50 mL); and dried to obtain the product as a gray solid (6.039 g, 90%). ^1^H NMR (CDCl_3_, 700 MHz): δ [ppm] 8.57 (s, 1H, NH), 8.20 (d, *J* = 1.8 Hz, 2H, 2CH_Ar_), 8.16 (d, *J* = 7.8 Hz, 4H, 4CH_Ar_), 7.71 (d, *J* = 8.7 Hz, 2H, 2CH_Ar_), 7.62 (dd, *J* = 8.9, 2.1 Hz, 2H, 2CH_Ar_), 7.41–7.36 (m, 8H, 8CH_Ar_), 7.29–7.26 (m, 4H, 4CH_Ar_). ^13^C{^1^H} NMR (175 MHz, CDCl_3_): δ [ppm] 141.9 (4C_Ar_), 139.3 (2CH_Ar_), 130.1 (2C_Ar_), 126.1 (2CH_Ar_), 125.9 (4CH_Ar_), 124.2 (2C_Ar_), 123.2 (4C_Ar_), 120.3 (4CH_Ar_), 119.8 (2CH_Ar_), 119.7 (4CH_Ar_), 112.0 (2CH_Ar_), 109.7 (4CH_Ar_). IR ν_max_ cm^–1^: 1452 (s), 1232 (s), 748 (s). mp: 330–333 °C.

#### 9′,9‴′,9‴‴′,9‴‴‴′-((4,8-Didecylbenzo[1,2-*b*:4,5-*b*′]difuran-2,3,6,7-tetrayl)tetrakis(benzene-4,1-diyl))tetrakis(9′*H*-9,3′:6′,9″-tercarbazole) (**2h**)

This compound was prepared according to the procedure for **2a** except **TBBDF** (0.15 mmol; 158 mg), tercarbazole (0.9 mmol; 447 mg), [Pd(allyl)Cl]_2_ (0.012 mmol; 4.5 mg; 8 mol %), *t*-BuXPhos (0.048 mmol; 20.4 mg; 32 mol %), and *t*-BuOLi (1 M, 0.9 mmol; 0.9 mL). After 72 h, the reaction mixture was cooled to RT and transferred into a separatory funnel. Chloroform (20 mL) and water (50 mL) were added, and the layers were separated. The aqueous layer was extracted with chloroform (3 × 20 mL). Combined organic layers were dried over anhydrous magnesium sulfate and concentrated. The crude product was stirred in chloroform (5 mL), and then MeOH (20 mL) was added. The precipitate formed was filtered off and dried under vacuum. Purification by flash, DCM/PE 1:1, afforded 233 mg (57%) of yellow powder. ^1^H NMR (CDCl_3_, 700 MHz): δ [ppm] 8.39–8.37 (m, 4H, 4CH_Ar_), 8.31–8.29 (m, 4H, 4CH_A_r), 8.21–8.17 (m, 16H, 16CH_Ar_), 8.12–8.06 (m, 12H, 12CH_Ar_), 7.91–7.87 (m, 4H, 4CH_Ar_), 7.84–7.82 (m, 4H, 4CH_Ar_), 7.77–7.73 (m, 8H, 8CH_Ar_), 7.66–7.63 (m, 4H, 4CH_Ar_), 7.46–7.42 (m, 18H, 18CH_Ar_), 7.40–7.38 (m, 14H, 14CH_Ar_), 7.33–7.28 (m, 16H, 16CH_Ar_), 3.13–3.04 (m, 4H, 2CH_2_), 1.87–1.76 (m, 4H, 2CH_2_), 1.35–1.08 (m, 28H, 14CH_2_), 0.72 (t, *J* = 7.2 Hz, 6H, 2CH_3_). ^13^C{^1^H} NMR (175 MHz, CDCl_3_): δ [ppm] 150.3 (2C_Ar_), 150.2 (2C_Ar_), 141.8 (8C_Ar_), 141.8 (8C_Ar_), 140.5 (4C_Ar_), 140.5 (4C_Ar_), 137.5 (2C_Ar_), 137.0 (2C_Ar_), 134.7 (2C_Ar_), 132.8 (4CH_Ar_), 131.0 (4C_Ar_), 130.8 (4C_Ar_), 130.7 (2C_Ar_), 128.1 (4CH_Ar_), 127.8 (4CH_Ar_), 127.2 (4CH_Ar_), 126.5 (4CH_Ar_), 126.3 (4CH_Ar_), 125.9 (8CH_Ar_), 125.9 (8CH_Ar_), 125.9 (4CH_Ar_), 124.4 (4C_Ar_), 124.3 (4C_Ar_), 123.3 (8C_Ar_), 123.3 (8C_Ar_), 123.2 (2C_Ar_), 120.4 (8CH_Ar_), 120.3 (8CH_Ar_), 120.3 (4CH_Ar_), 120.0 (4CH_Ar_), 119.9 (4CH_Ar_), 119.8 (8CH_Ar_), 119.8 (8CH_Ar_), 118.6 (2C_Ar_), 116.6 (2C_Ar_), 111.3 (4CH_Ar_), 111.1 (4CH_Ar_), 109.6 (8CH_Ar_), 31.8 (2CH_3_), 31.2 (2CH_2_), 30.4 (2CH_2_), 29.7 (2CH_2_), 29.7 (2CH_2_), 29.6 (2CH_2_), 29.3 (2CH_2_), 26.0 (2CH_2_), 22.5 (2CH_2_), 13.9 (2CH_3_). IR ν_max_ cm^–1^: 1454 (s), 1226 (s), 744 (s). mp: 276–281 °C. Anal. Calcd for C_198_H_146_N_12_O_2_: C, 87.26; H, 5.40; N, 6.17; O, 1.17. Found: C, 87.61; H, 5.43; N, 6.27; O, 1.14.

#### *tert*-Butyl 3,6-Bis(diphenylamino)-9*H*-carbazole-9-carboxylate^57^

In a 10 mL vial, Pd_2_(dba)_3_ (0.048 mmol; 44 mg; 2 mol %) and XPhos (0.192 mmol; 91.6 mg; 8 mol %) were mixed in dry, degassed toluene (5 mL) and stirred for 5 min in an inert gas atmosphere. In a round-bottom flask, diphenylamine (5 mmol; 0.845 g) was dissolved in dry, degassed toluene (10 mL), *t*-BuONa (5 mmol; 0.480 mL) was added, and the mixture was stirred for 5 min under argon. *tert*-Butyl-3,6-dibromo-9*H*-carbazole-9-carboxylate (2.4 mmol; 1.02 g) was added, followed by the catalyst mixture. The flask was immersed in an oil bath preheated to 100 °C and stirred at this temperature for 24 h. Then, the reaction mixture was cooled to RT and transferred into a separatory funnel. DCM (10 mL) and water (20 mL) were added, and the layers were separated. The aqueous layer was extracted with DCM (3 × 20 mL). Combined organic layers were dried over anhydrous magnesium sulfate and concentrated. The crude product was stirred in MeOH (50 mL) at 65 °C for 0.5 h, and the solid phase was filtered off and dried to obtain the product as a yellow solid, 1.14 g (79%). ^1^H NMR (DMSO-*d*_6_, 700 MHz): δ [ppm] 8.19 (d, *J* = 8.9 Hz, 2H, 2CH_Ar_), 7.83 (d, *J* = 2.5 Hz, 2H, 2CH_Ar_), 7.26–7.21 (m, 10H, 10CH_Ar_), 6.98–6.94 (m, 12H, 12CH_Ar_), 1.69 (s, 9H, 3CH_3_). ^13^C{^1^H} NMR (175 MHz, DMSO-*d*_6_): δ [ppm] 150.6 (C), 148.1 (2C_Ar_), 143.3 (C_Ar_), 135.4 (C_Ar_), 129.8 (8CH_Ar_), 129.6 (4CH_Ar_), 126.6 (2CH_Ar_), 126.6 (2C_Ar_), 123.1 (8CH_Ar_), 122.7 (4CH_Ar_), 118.0 (2CH_Ar_), 117.6 (2CH_Ar_), 84.7 (C), 28.3 (3CH_3_). IR ν_max_ cm^–1^: 1478 (s), 1214 (s), 749 (s). mp: 131–132 °C.

#### *N*^3^,*N*^3^,*N*^6^,*N*^6^-Tetraphenyl-9*H*-carbazole-3,6-diamine (**1d**)^57^

*tert*-Butyl-3,6-bis(diphenylamino)-9*H*-carbazole-9-carboxylate (1.66 mmol; 1 g) was dissolved in DCM (10 mL). Trifluoroacetic acid (16.6 mmol; 1.9 g) was added dropwise to the mixture and stirred at RT for 2.5 h. Then, DCM (50 mL) was added to the reaction mixture, and the organic layer was washed with saturated aqueous NaHCO_3_ solution and dried over MgSO_4_. The solvent was evaporated to afford 0.681 g of a green solid (82%). ^1^H NMR (DMSO-*d*_6_, 700 MHz): δ [ppm] 11.37 (s, 1H, NH), 7.88 (d, *J* = 2.2 Hz, 2H, 2CH_Ar_), 7.50 (d, *J* = 8.9 Hz, 2H, 2CH_Ar_), 7.23–7.19 (m, 8H, 8CH_Ar_), 7.17 (dd, *J* = 8.6, 2.1 Hz, 2H, 2CH_Ar_), 6.96–6.93 (m, 8H, 8CH_Ar_), 6.90 (t, *J* = 7.4 Hz, 4H, 4CH_Ar_). ^13^C{^1^H} NMR (175 MHz, DMSO-*d*_6_): δ [ppm] 148.7 (4C_Ar_), 138.9 (2C_Ar_), 138.5 (2C_Ar_), 129.6 (8CH_Ar_), 126.5 (2CH_Ar_), 123.9 (2C_Ar_), 122.3 (8CH_Ar_), 121.8 (4CH_Ar_), 119.8 (2CH_Ar_), 112.8 (2CH_Ar_). IR ν_max_ cm^–1^: 1476 (s), 1230 (s), 748 (s). mp: 246–248 °C.

#### 9,9′,9″,9‴-((4,8-Didecylbenzo[1,2-*b*:4,5-*b*′]difuran-2,3,6,7-tetrayl)tetrakis(benzene-4,1-diyl))tetrakis(*N*^3^,*N*^3^,*N*^6^,*N*^6^-tetraphenyl-9*H*-carbazole-3,6-diamine) (**2i**)

This compound was prepared according to the procedure for **2h** except *N*^3^,*N*^3^,*N*^6^,*N*^6^-tetraphenyl-9*H*-carbazole-3,6-diamine (0.9 mmol, 451 mg) and *t*-BuONa (0.9 mmol, 86 mg), and the reaction was performed at 170 °C in a sealed tube. The reaction was cooled to room temperature and poured into MeOH (50 mL). The precipitated solid was filtered on a glass Büchner funnel, washed with MeOH (30 mL), and air-dried. Purification by flash chromatography, DCM/PE 2:3, afforded 193 mg (43%) of yellow powder. ^1^H NMR (CDCl_3_, 700 MHz): δ [ppm] 7.91–7.83 (m, 16H, 16CH_Ar_), 7.79–7.75 (m, 4H, 4CH_Ar_), 7.61–7.57 (m, 4H, 4CH_Ar_), 7.52–7.49 (m, 4H, 4CH_A_r), 7.41–7.38 (m, 4H, 4CH_Ar_), 7.31–7.29 (m, 4H, 4CH_Ar_), 7.26–7.20 (m, 36H, 36CH_Ar_), 7.14–7.06 (m, 32H, 32CH_Ar_), 6.99–6.93 (m, 16H, 16CH_Ar_), 2.96–2.91 (m, 4H, 2CH_2_), 1.68–1.63 (m, 4H, 2CH_2_), 1.29–1.14 (m, 28H, 14CH_2_), 0.78 (t, *J* = 7.2 Hz, 6H, 2CH_3_). ^13^C{^1^H} NMR (175 MHz, CDCl_3_): δ [ppm] 150.2 (2C_Ar_), 150.0 (2C_Ar_), 148.5 (8C_Ar_), 148.5 (8C_Ar_), 140.9 (4C_Ar_), 140.8 (4C_Ar_), 138.2 (4C_Ar_), 138.1 (4C_Ar_), 137.7 (2C_Ar_), 137.3 (2C_Ar_), 133.7 (2C_Ar_), 132.4 (4CH_Ar_), 129.8 (2C_Ar_), 129.1 (16CH_Ar_), 129.1 (16CH_Ar_), 127.7 (4CH_Ar_), 127.3 (4CH_Ar_), 126.6 (4CH_Ar_), 126.1 (2C_Ar_), 126.0 (4CH_Ar_), 125.9 (4CH_Ar_), 124.4 (4C_Ar_), 124.3 (4C_Ar_), 122.8 (16CH_Ar_), 122.8 (16CH_Ar_), 121.8 (8CH_Ar_), 121.7 (8CH_Ar_), 118.7 (4CH_Ar_), 118.6 (4CH_Ar_), 118.3 (2C_Ar_), 116.3 (2C_Ar_), 110.9 (4CH_Ar_), 110.7 (4CH_Ar_), 31.8 (2CH_2_), 31.2 (2CH_2_), 30.2 (2CH_2_), 29.6 (2CH_2_), 29.6 (2CH_2_), 29.5 (2CH_2_), 29.2 (2CH_2_), 25.9 (2CH_2_), 22.6 (2CH_2_), 14.1 (2CH_3_). IR ν_max_ cm^–1^: 1486 (s), 1224 (m), 749 (s). mp: 156–157 °C. Anal. Calcd for C_198_H_162_N_12_O_2_: C, 86.75; H, 5.96; N, 6.13; O, 1.17. Found: C, 86.44; H, 6.04; N, 6.16; O, 1.11.

#### 4′,4‴,4‴″,4‴‴′-(4,8-Didecylbenzo[1,2-*b*:4,5-*b*′]difuran-2,3,6,7-tetrayl)tetrakis(*N*,*N*-diphenyl-[1,1′-biphenyl]-4-amine) (**2j**)

In a Schlenk tube, **TBBDF** (0.5 mmol; 527 mg), 4-(diphenylamino)phenylboronic acid pinacol ester (2.5 mmol; 927 mg), and Pd(dppf)Cl_2_·DCM (0.01 mmol; 8.17 mg; 2 mol %) were mixed in degassed 1,4-dioxane (5 mL) and stirred under argon for 5 min. 3 M K_3_PO_4_ (1 mL) was added, and the mixture was immersed in an oil bath preheated to 100 °C and stirred overnight. It was cooled to RT and poured into MeOH (20 mL), and the precipitate formed was filtered off and dried under vacuum. Purification by flash chromatography, DCM/PE 1:1, afforded 773 mg (90%) of green powder. ^1^H NMR (CDCl_3_, 700 MHz): δ [ppm] 7.75 (d, *J* = 8.2 Hz, 4H, 4CH_Ar_), 7.64 (d, *J* = 8.6 Hz, 4H, 4CH_Ar_), 7.59 (t, *J* = 8.7 Hz, 8H, 8CH_Ar_), 7.47 (d, *J* = 8.6 Hz, 4H, 4CH_Ar_), 7.44 (d, *J* = 8.7 Hz, 4H, 4CH_Ar_), 7.31–7.27 (m, 8H, 8CH_Ar_), 7.27–7.23 (m, 8H, 8CH_Ar_), 7.21 (d, *J* = 8.5 Hz, 4H, 4CH_Ar_), 7.17 (dd, *J* = 8.6, 1.2 Hz, 8H, 8CH_Ar_), 7.12 (dd, *J* = 8.7, 0.8 Hz, 8H, 8CH_Ar_), 7.10 (d, *J* = 8.6 Hz, 4H, 4CH_Ar_), 7.06 (tt, *J* = 7.4, 1.0 Hz, 4H, 4CH_Ar_), 7.03 (tt, *J* = 7.9, 1.0 Hz, 4H, 4CH_Ar_), 2.81–2.72 (m, 4H, 2CH_2_), 1.52–1.47 (m, 4H, 2CH_2_), 1.27–1.10 (m, 28H, 14CH_2_), 0.82 (t, *J* = 7.2 Hz, 6H, 2CH_3_). ^13^C{^1^H} NMR (175 MHz, CDCl_3_): δ [ppm] 149.9 (2C_Ar_), 147.7 (8C_Ar_), 147.6 (4C_Ar_), 147.5 (2C_Ar_), 147.4 (2C_Ar_), 140.0 (2C_Ar_), 134.6 (2C_Ar_), 134.2 (2C_Ar_), 131.3 (4CH_Ar_), 131.2 (2C_Ar_), 129.3 (8CH_Ar_), 129.3 (8CH_Ar_), 127.7 (4CH_Ar_), 127.4 (4CH_Ar_), 126.9 (4CH_Ar_), 126.7 (4CH_Ar_), 126.3 (4CH_Ar_), 126.0 (2C_Ar_), 124.5 (8C_Ar_), 124.5 (8CH_Ar_), 124.0 (4CH_Ar_), 123.7 (4CH_Ar_), 123.0 (8CH_Ar_), 118.4 (2C_Ar_), 118.4 (2C_Ar_), 116.1 (2C_Ar_), 31.9 (2CH_2_), 31.0 (2CH_2_), 29.9(2CH_2_), 29.6 (2CH_2_), 29.6 (2CH_2_), 29.4 (4CH_2_), 25.6 (2CH_2_), 22.7 (2CH_2_), 14.1 (2CH_3_). IR ν_max_ cm^–1^: 1486 (s), 1274 (m), 749 (m). mp: 261–262 °C. Anal. Calcd for C_126_H_114_N_4_O_2_: C, 88.18; H, 6.70; N, 3.26; O, 1.86. Found: C, 87.94; H, 6.67; N, 3.23; O, 1.90.

## Supporting Information Available

Experimental details, compound characterization data, crystallographic data, and NMR spectra (PDF)
