# Supplementary material for: Buchwald–Hartwig Amination of Aryl Halides with Heterocyclic Amines in the Synthesis of Highly Fluorescent Benzodifuran-Based Star-Shaped Organic Semiconductors
Source: J Org Chem. 2021 Dec 3;86(24):17594–605. doi: 10.1021/acs.joc.1c01583 (PMC8689646; doi:10.1021/acs.joc.1c01583)
Supplement: Supplementary file 1 — jo1c01583_si_001.pdf [file jo1c01583_si_001.pdf]

Supporting Information

Buchwald-Hartwig Amination of Aryl Halides with Heterocyclic Amines in the Synthesis of Highly Fluorescent Benzodifuran Based Star-Shaped Organic Semiconductors

Mariusz J. Bosiak*[a,e], Alicja A. Zielińska[b,e], Piotr Trzaska[a,e], Dariusz Kędziera[c], Jörg Adams[d]

[a] Department of Organic Chemistry, Faculty of Chemistry, Nicolaus Copernicus University in Toruń, 7 Gagarin Street, 87-100 Toruń, Poland

[b] Doctoral School of Exact and Natural Sciences "Academia Scientiarum Thoruniensis", Nicolaus Copernicus University in Toruń, 5 Grudziądzka Street, 87-100 Toruń, Poland

[c] Department of Chemistry of Materials Adsorption and Catalysis, Faculty of Chemistry, Nicolaus Copernicus University in Toruń, 7 Gagarin Street, 87-100 Toruń, Poland

[d] Institute of Physical Chemistry, Clausthal University of Technology, Arnold-Sommerfeld-Str. 4, 38678 Clausthal-Zellerfeld, Germany

[e] Noctiluca SA, 7/41B Gagarina Street, 87-100 Toruń, Poland

E-mail address: bosiu@umk.pl

Table of Contents

1. Photoluminescence of the TBBDF series 2

2. UV-Vis spectra 3

3. Computation data 13

Table S1. Summary of the geometry optimization step. PBE0/6-31G* level of theory with GD3 empirical dispersion, gas phase. 13

Table S2. HOMO-LUMO energies for different gas phase, toluene and chloroform. calculated at the PBE0/6-31G* level of theory with GD3 empirical dispersion, at the geometry for gas phase. Energies in Hartree 13

Table S3. The most intense absorption lines calculated for different solvents at the PBE0/6-31G* level of theory with GD3 empirical dispersion, at the geometry for gas phase. Oscillator strengths, denoted as *f*. 14

4. **Table S4.** $S_0 \rightarrow S_1$ excitation analysis in terms of HOMO-LUMO orbitals. 15

5. **Table S5.** $S_0 \rightarrow S_1$ excitation analysis in terms of Natural Transition Orbitals. 16

6. HOMO and LUMO orbitals calculated within PBE0/6-31G* level of theory with GD3 empirical dispersion 17

7. Natural Transition Orbitals for 2a-2j derived for $S_0 \rightarrow S_1$ excitation. 22

8. 1H NMR and 13C{1H} NMR spectra 28

9. Geometries of the optimized structures: PBE0/6-31G* level of theory with GD3 empirical dispersion, gas phase 44

1. Photoluminescence of the TBBDF series

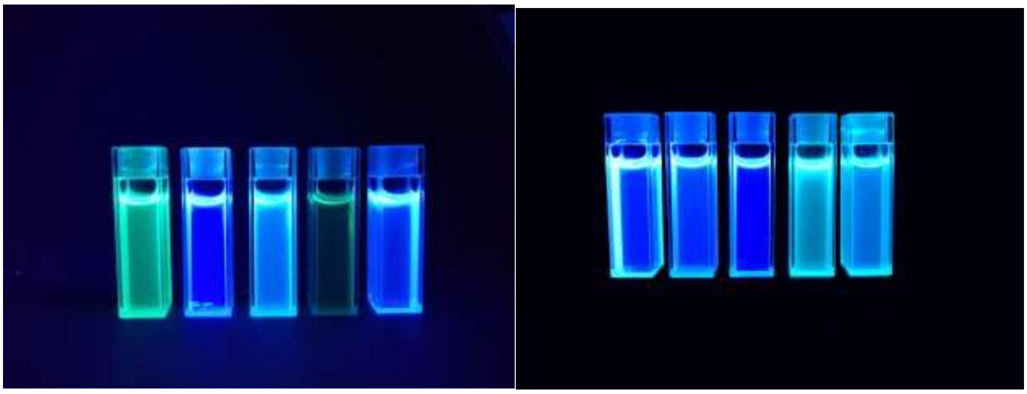

Fig. S1. Photoluminescence of the **BDF** star-shaped organic semiconductors series **2a-2j** in toluene; irradiation at 365 nm.

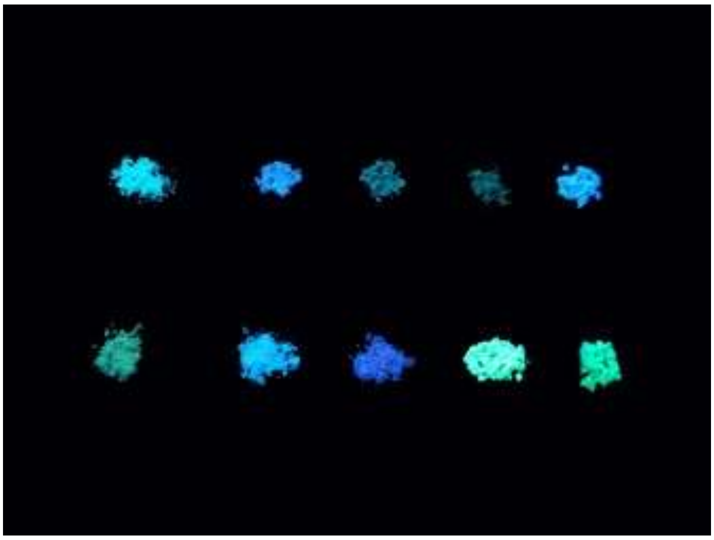

Fig. S2. Photoluminescence of the **BDF** star-shaped organic semiconductors series **2a-2j** in solid state; irradiation at 365 nm.

2. UV-Vis spectra

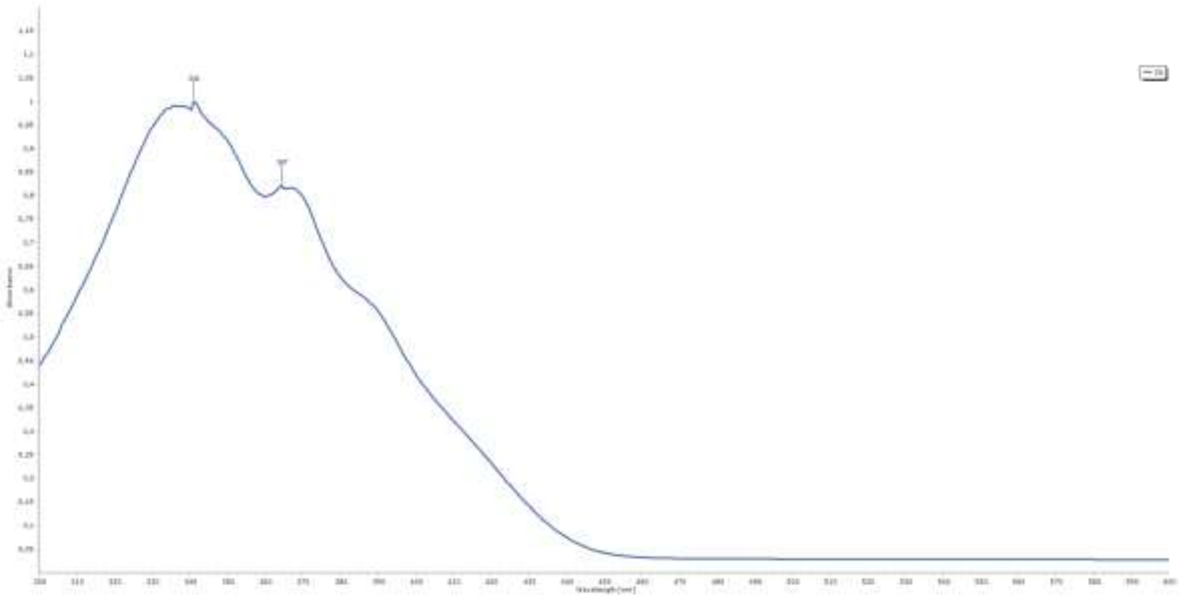

Fig. S3. The UV-Vis spectrum of compound **2a** in toluene.

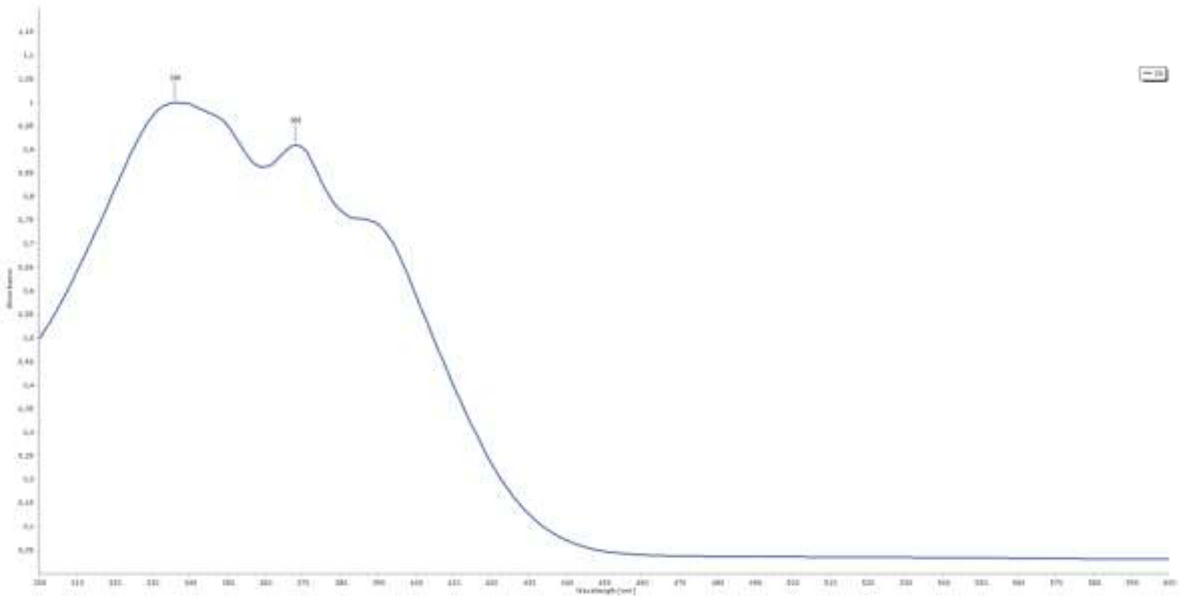

Fig. S4. The UV-Vis spectrum of compound **2a** in chloroform.

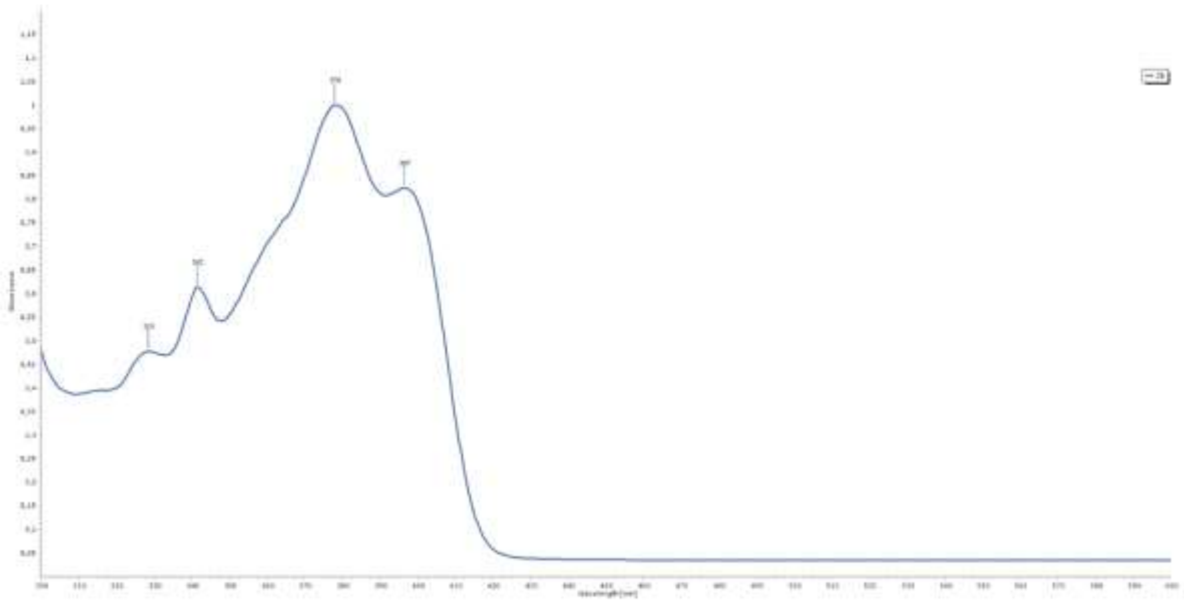

Fig. S5. The UV-Vis spectrum of compound **2b** in toluene.

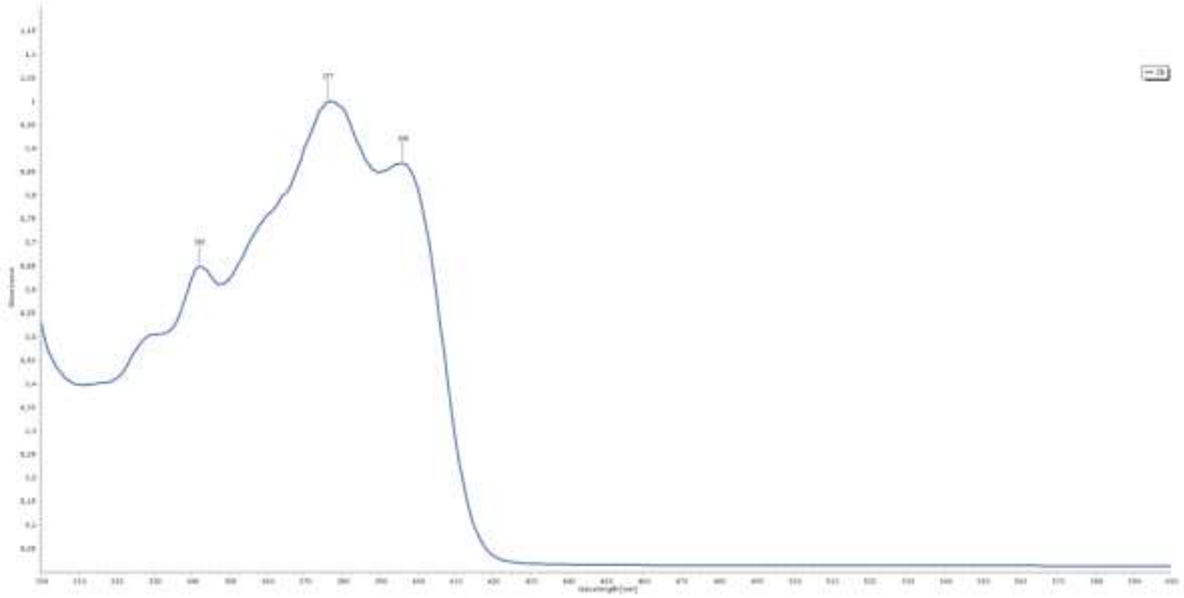

Fig. S6. The UV-Vis spectrum of compound **2b** in chloroform.

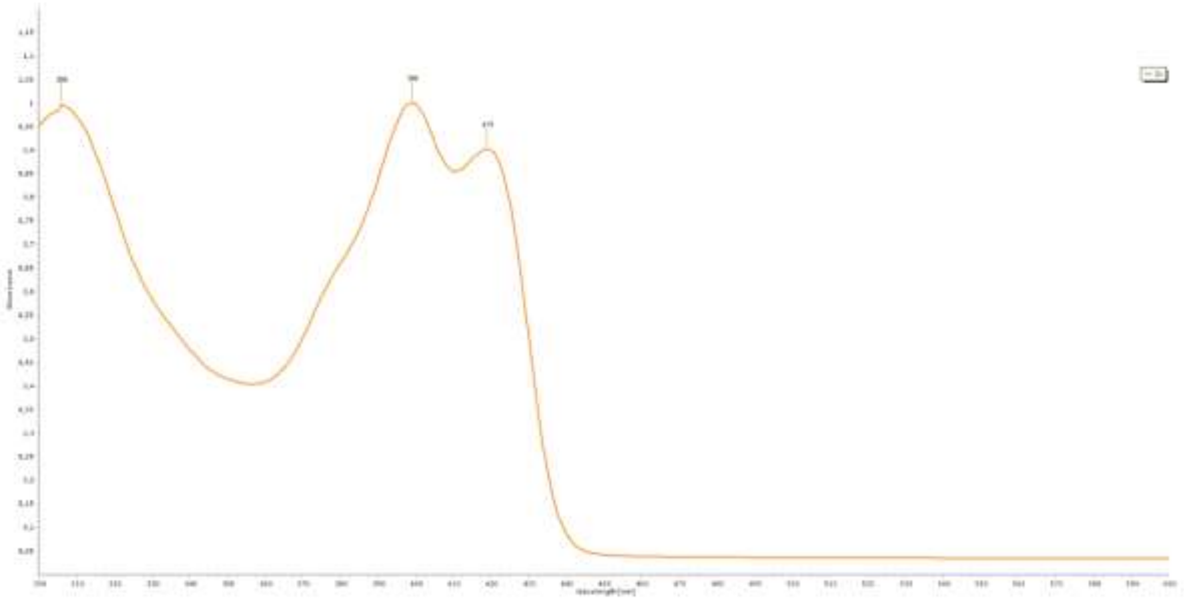

Fig. S7. The UV-Vis spectrum of compound **2c** in toluene.

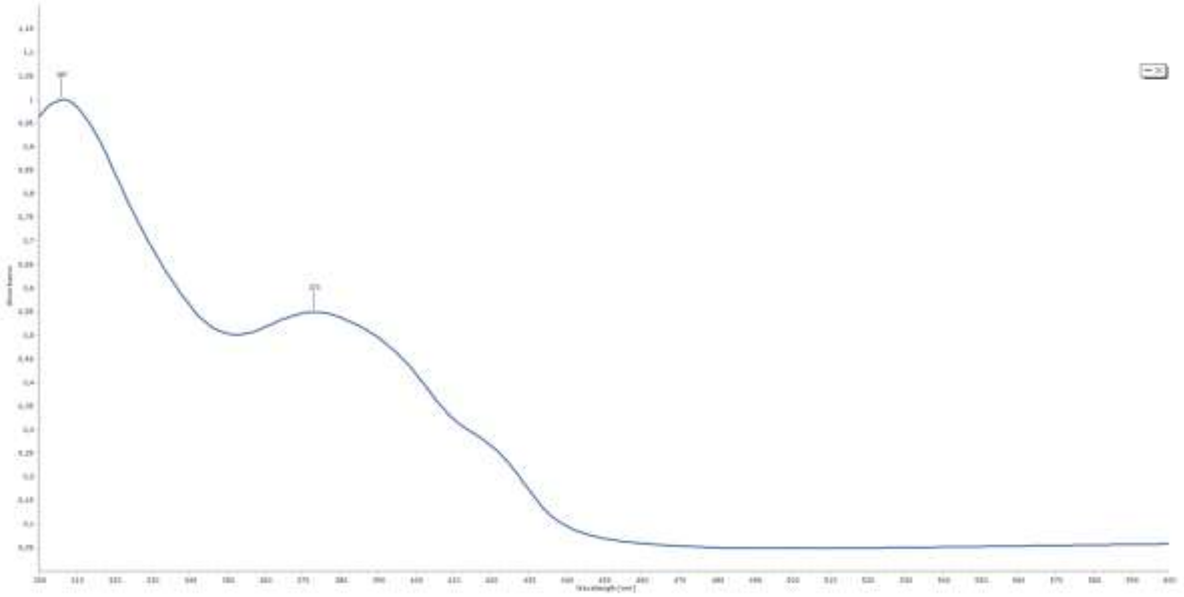

Fig. S8. The UV-Vis spectrum of compound **2c** in chloroform.

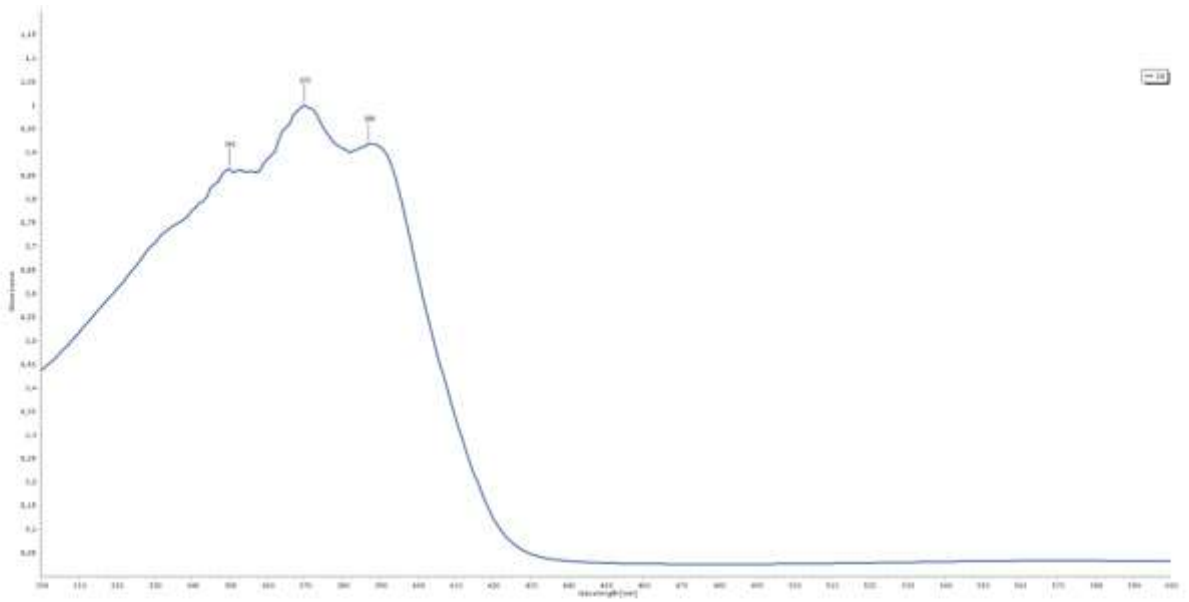

Fig. S9. The UV-Vis spectrum of compound **2d** in toluene.

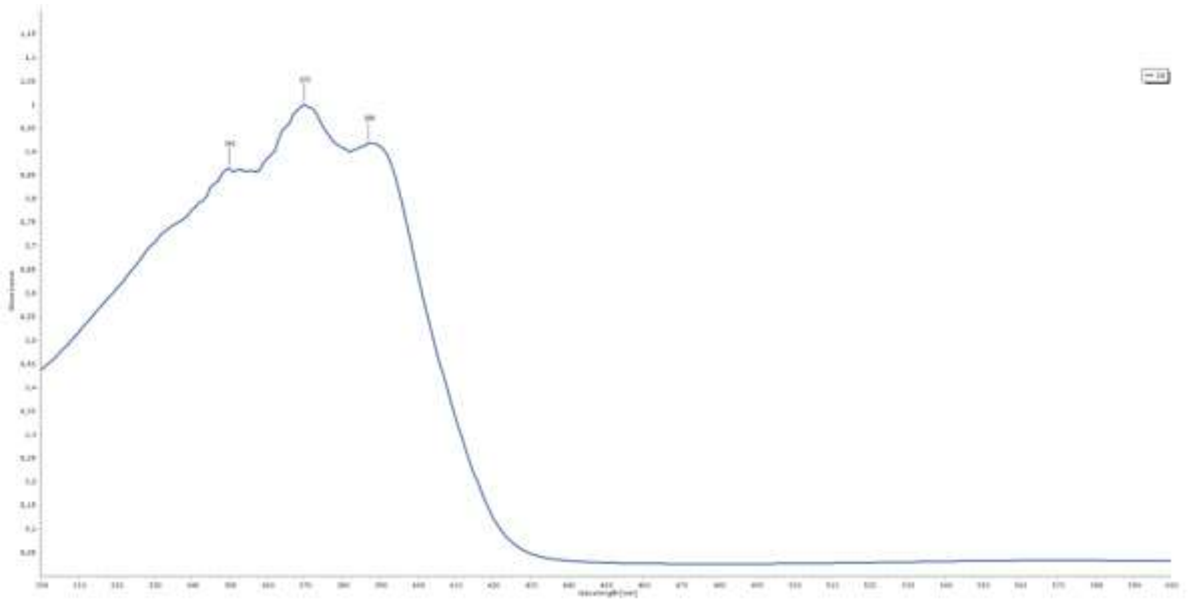

Fig. S10. The UV-Vis spectrum of compound **2d** in chloroform.

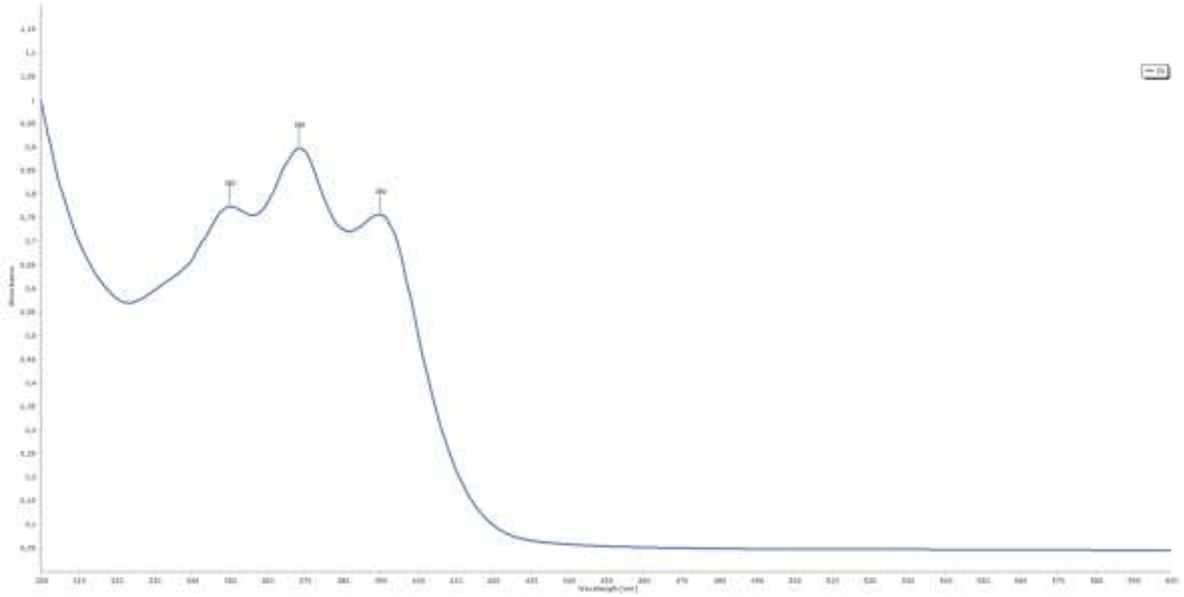

Fig. S11. The UV-Vis spectrum of compound **2e** in toluene.

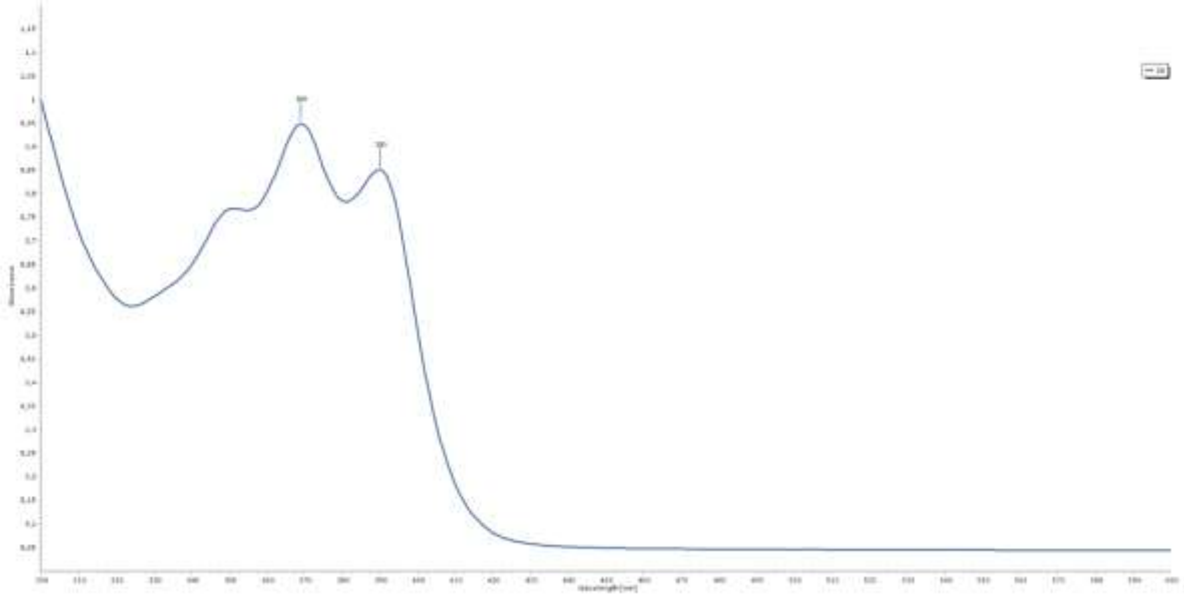

Fig. S12. The UV-Vis spectrum of compound **2e** in chloroform.

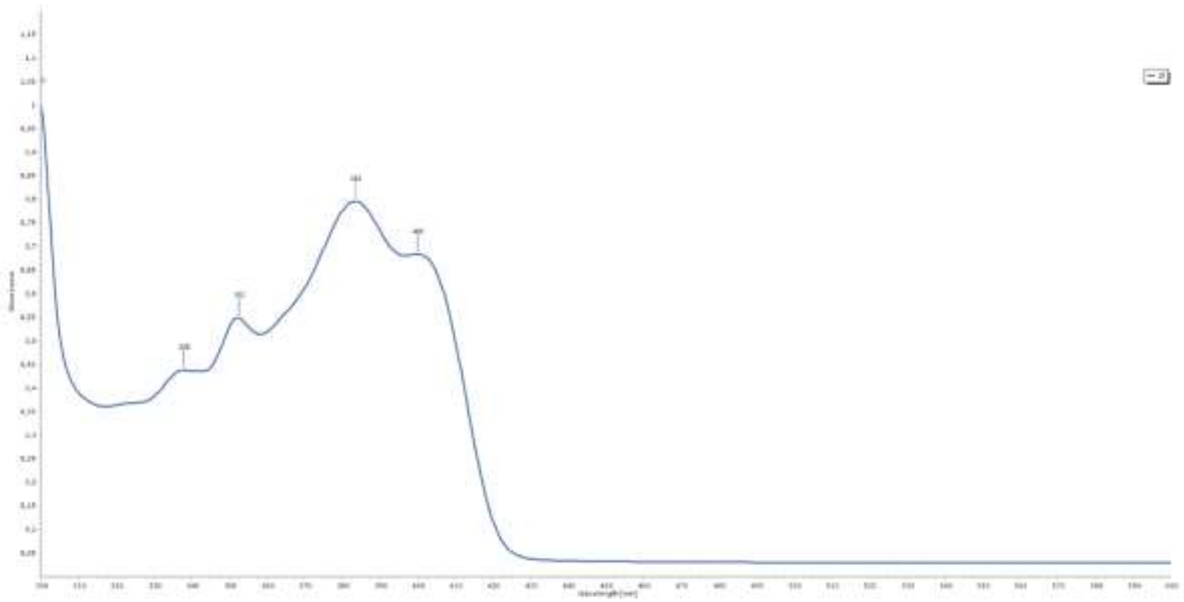

Fig. S13. The UV-Vis spectrum of compound **2f** in toluene.

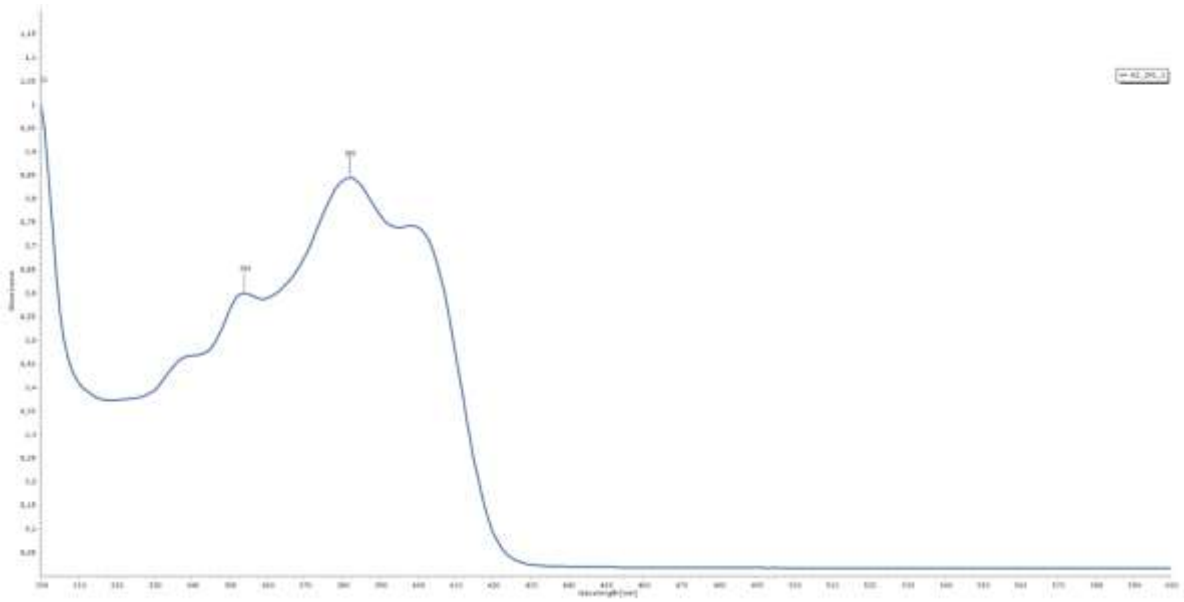

Fig. S14. The UV-Vis spectrum of compound **2f** in chloroform.

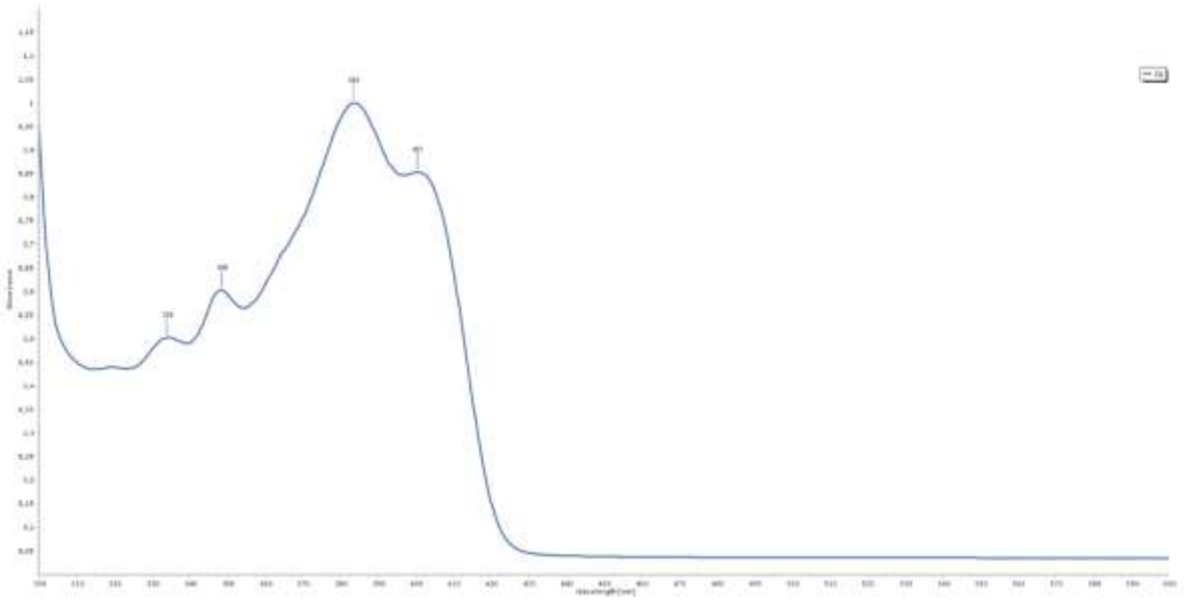

Fig. S15. The UV-Vis spectrum of compound **2g** in toluene.

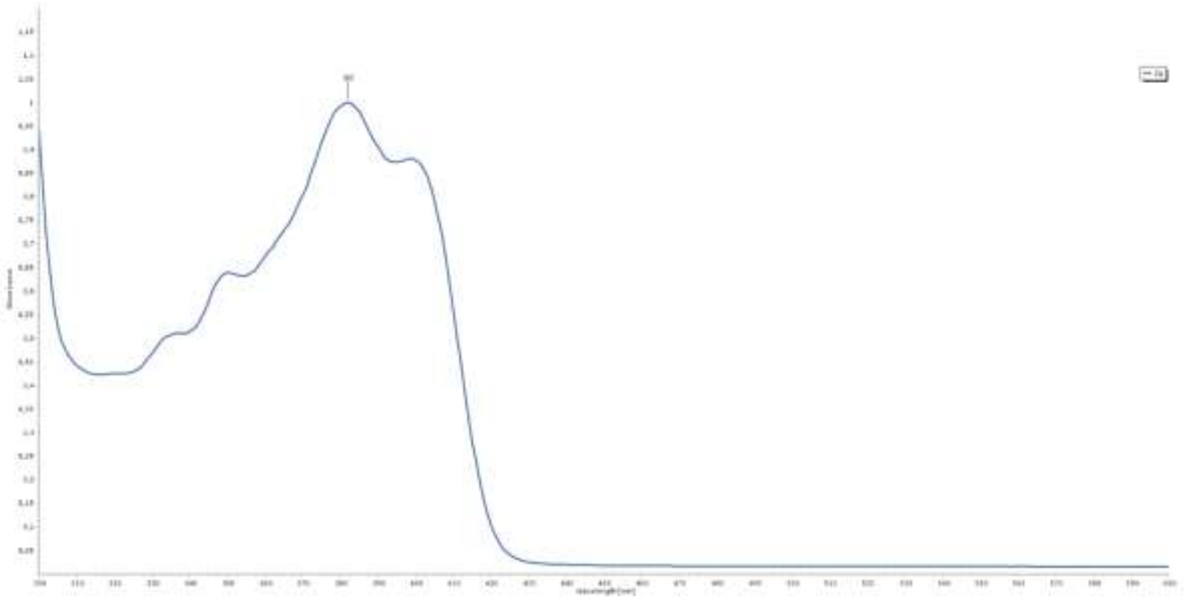

Fig. S16. The UV-Vis spectrum of compound **2g** in chloroform.

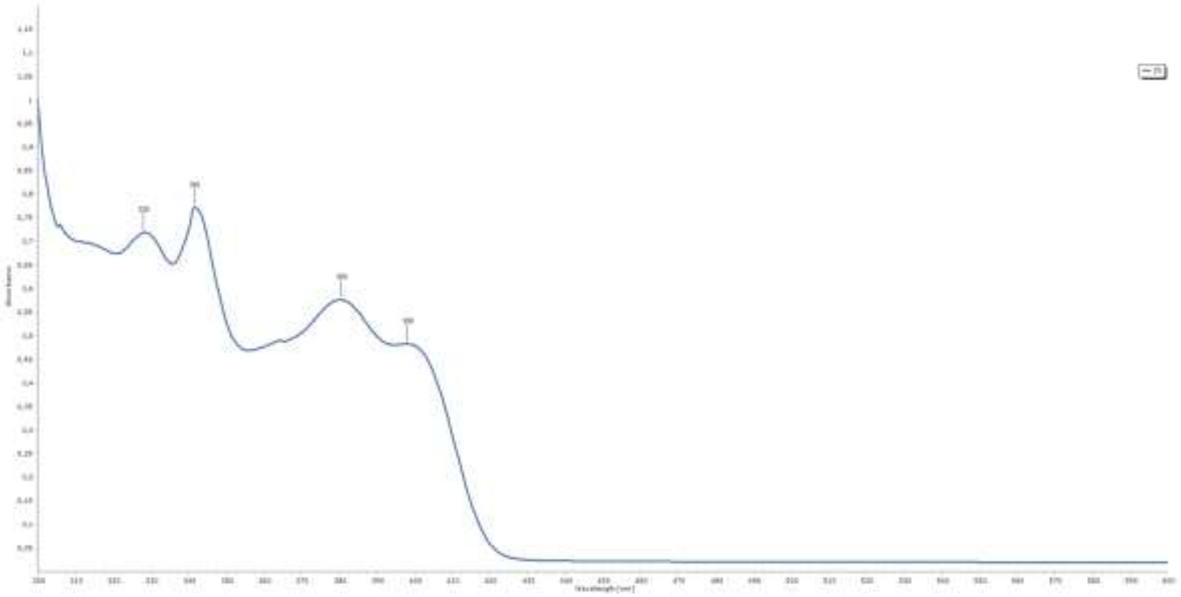

Fig. S17. The UV-Vis spectrum of compound **2h** in toluene.

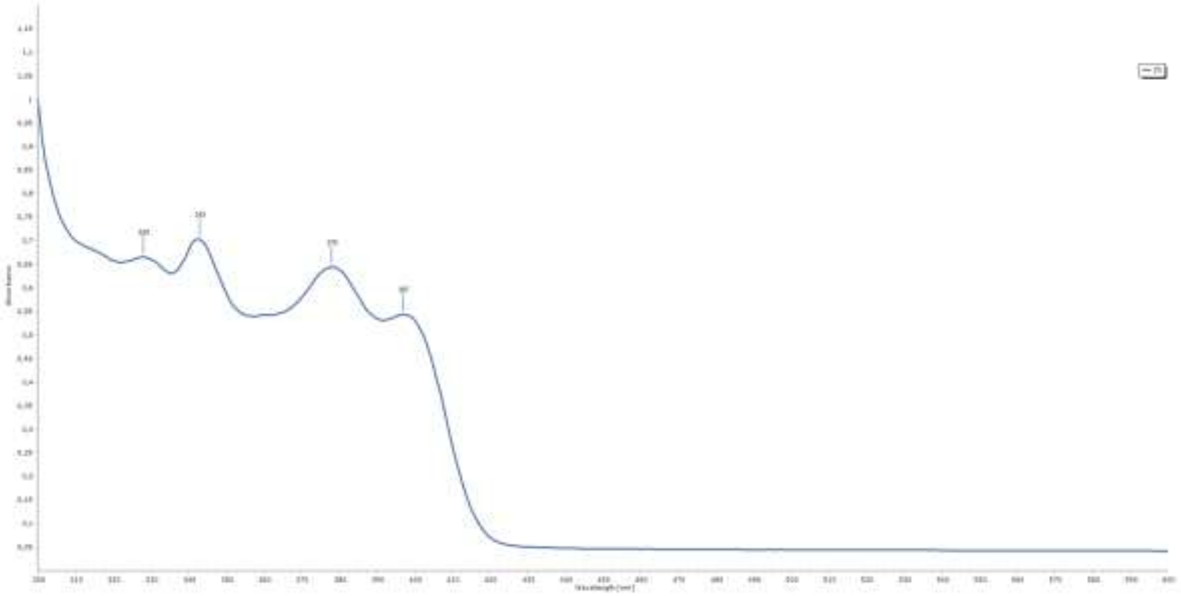

Fig. S18. The UV-Vis spectrum of compound **2h** in chloroform.

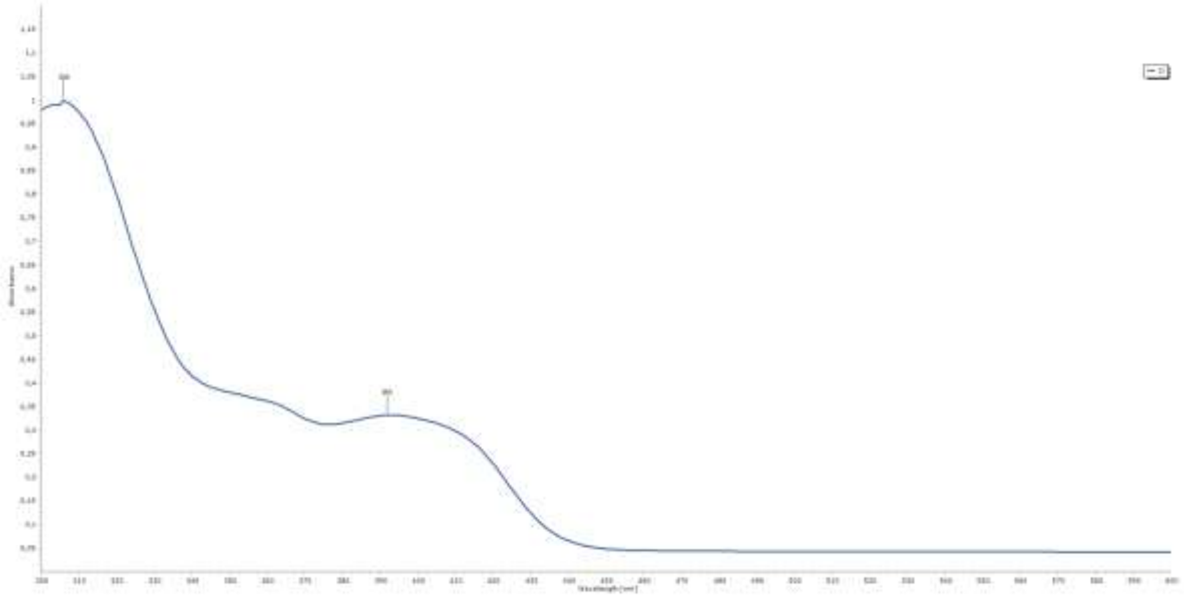

Fig. S19. The UV-Vis spectrum of compound **2i** in toluene.

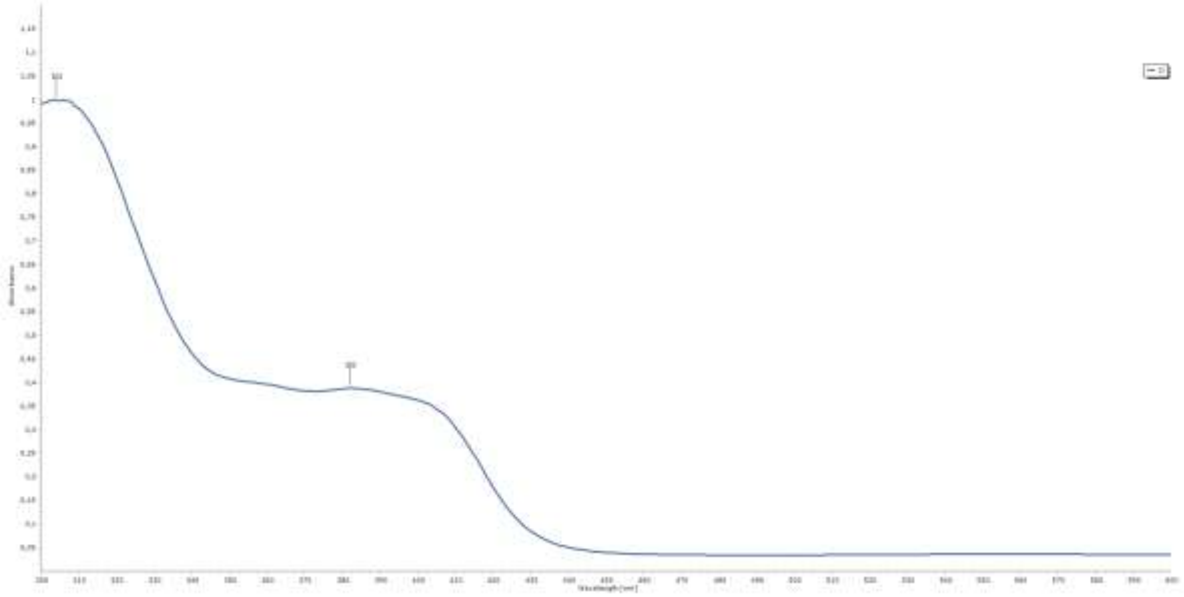

Fig. S20. The UV-Vis spectrum of compound **2i** in chloroform.

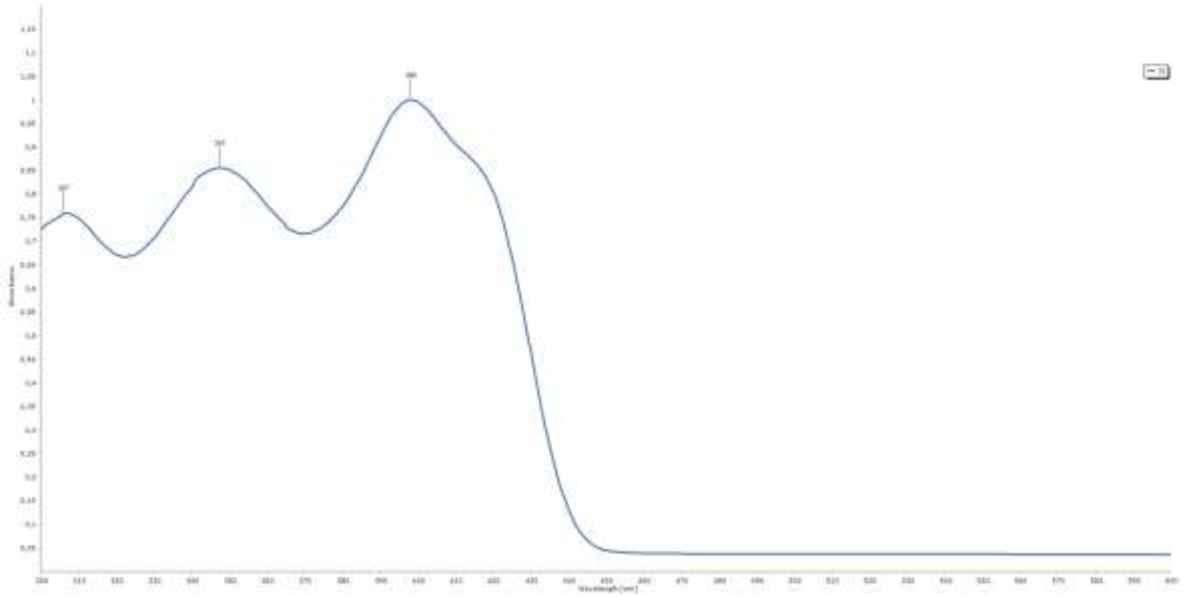

Fig. S21. The UV-Vis spectrum of compound **2j** in toluene.

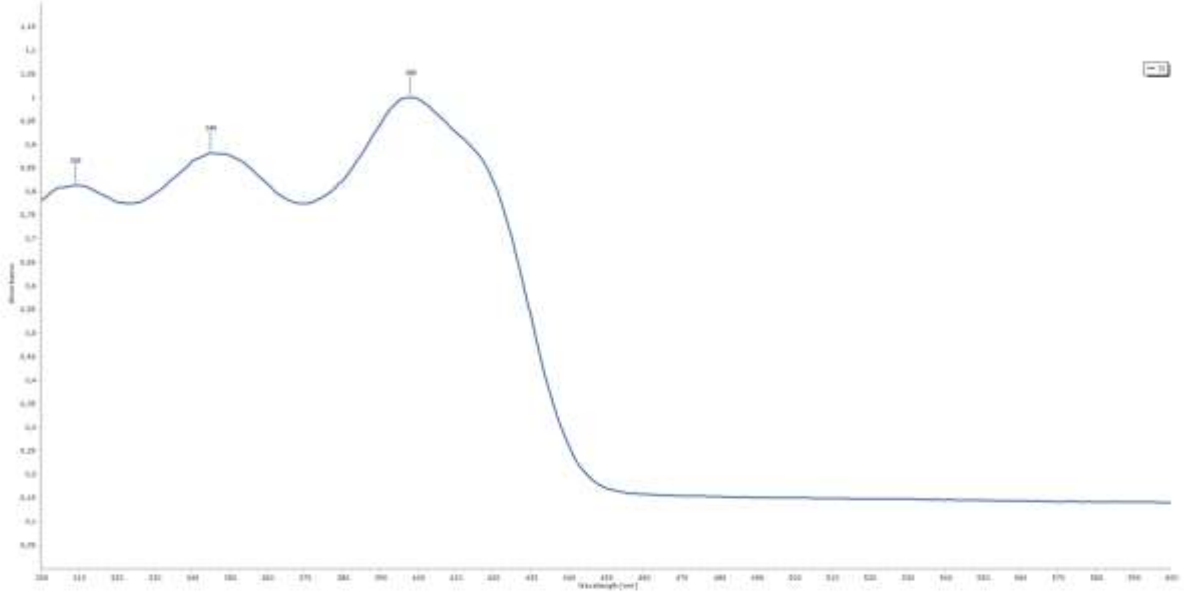

Fig. S22. The UV-Vis spectrum of compound **2j** in chloroform.

3. Computation data

Table S1. Summary of the geometry optimization step. PBE0/6-31G* level of theory with GD3 empirical dispersion, gas phase.

Structure	Total energy [Hartree]	NImag
**2a**	-4305.86110980	0
**2b**	-4310.52939936	0
**2c**	-4606.33885589	0
**2d**	-5897.91171530	0
**2e**	-4777.00116994	0
**2f**	-7447.26748358	0
**2g**	-5562.44061998	0
**2h**	-8431.48119631	0
**2i**	-8440.83747225	0
**2j**	-5233.70246645	0

Table S2. HOMO-LUMO energies for different gas phase, toluene and chloroform. calculated at the PBE0/6-31G* level of theory with GD3 empirical dispersion, at the geometry for gas phase. Energies in Hartree.

Comp.	Gas phase		Toluene		Chloroform	
	HOMO	LUMO	HOMO	LUMO	HOMO	LUMO
**2a**	-0.19723	-0.06199	-0.1973	-0.06155	-0.19762	-0.06162
**2b**	-0.17610	-0.04719	-0.17939	-0.05093	-0.18110	-0.05292
**2c**	-0.18371	-0.06302	-0.18529	-0.0619	-0.18622	-0.06145
**2d**	-0.19057	-0.05559	-0.19179	-0.05614	-0.19260	-0.05685
**2e**	-0.19045	-0.05854	-0.19236	-0.05884	-0.19353	-0.05911
**2f**	-0.19244	-0.05857	-0.19369	-0.05924	-0.19457	-0.05982
**2g**	-0.19131	-0.05782	-0.19272	-0.05874	-0.19382	-0.05957
**2h**	-0.19763	-0.07479	-0.19839	-0.06943	-0.19883	-0.06718
**2i**	-0.17552	-0.05822	-0.17844	-0.05839	-0.18008	-0.05846
**2j**	-0.18048	-0.05293	-0.18476	-0.05779	-0.18679	-0.06015

Table S3. The most intense absorption lines calculated for different solvents at the PBE0/6-31G* level of theory with GD3 empirical dispersion, at the geometry for gas phase. Oscillator strengths, denoted as *f*.

Comp.	Gas phase		Toluene		Chloroform		DMSO		THF	
	λ [nm]	*f*	λ [nm]	*f*	λ [nm]	*f*	λ [nm]	*f*	λ [nm]	*f*
**2a**	394.8	1.3393	399.89	1.6108	398.46	1.5984	397.03	1.5924	397.62	1.5854
	311.9	0.5357	310.33	0.8917	309.26	0.6729	307.74	0.6537	308.08	0.6161
	340.6	0.1343	308.27	0.0654	337.93	0.2341	309.82	0.1332	337.39	0.2395
	309.0	0.1631	339.15	0.2138	308.30	0.2924	336.55	0.2514	309.24	0.3188
**2b**	417.6	1.5916	427.56	1.8728	427.93	1.8450	428.95	1.8181	427.91	1.8234
	325.7	0.3316	325.25	0.4242	324.60	0.4201	324.14	0.3900	324.34	0.4105
	352.5	0.3183	358.08	0.3456	360.99	0.3264	365.02	0.3125	362.39	0.3184
**2c**	363.4	0.8082	368.85	0.9737	367.67	0.9680	366.48	0.9275	367.00	0.9496
	455.3	0.2934	445.10	0.4174	438.87	0.4374	431.46	0.4718	436.05	0.4444
	372.2	0.1578	364.92	0.1484	361.06	0.122	356.05	0.1238	359.22	0.1201
**2d**	399.4	0.1363	387.10	0.0912	323.15	0.0996	322.15	0.0924	322.73	0.0966
	388.3	1.3088	397.75	1.6228	396.68	1.6663	395.39	1.6927	395.93	1.6658
**2e**	365.9	1.2016	371.85	1.4274	371.15	1.4091	370.65	1.3836	370.67	1.3938
	347.6	0.0704	341.45	0.0853	337.98	0.0843	333.68	0.0831	336.40	0.0832
	405.2	0.0109	399.20	0.0239	396.00	0.0284	391.49	0.0379	394.20	0.0305
**2f**	400.9	1.3244	404.45	1.5611	402.93	1.5565	401.30	1.5581	402.08	1.5482
	316.1	0.6568	315.14	0.6240	313.88	0.6295	314.09	0.4564	313.85	0.5662
	353.0	0.1428	352.03	0.1921	351.12	0.2027	349.90	0.2170	350.68	0.2066
	341.9	0.0899	341.59	0.0580	315.04	0.1877	315.57	0.0794	315.15	0.1218
**2g**	401.6	1.5659	406.22	1.8375	404.78	1.8293	403.21	1.8283	403.93	1.8187
	314.6	0.5932	313.62	0.4681	313.37	0.3939	313.41	0.2807	313.31	0.3383
	312.3	0.0762	352.52	0.1139	351.51	0.1206	311.28	0.1282	350.95	0.1260
	353.5	0.0864	314.24	0.2038	310.94	0.2460	349.80	0.1457	310.96	0.1818
**2h**	419.1	0.8899	409.47	1.6025	405.16	1.7191	401.12	1.8074	403.36	1.7478
	372.9	0.5166	366.70	0.2438	359.78	0.1414	344.75	0.0574	356.66	0.1062
**2i**	447.0	0.7399	438.79	0.9983	433.26	1.0569	425.70	1.1655	430.58	1.0833
	387.1	0.0360	386.78	0.0464	374.23	0.0407	367.17	0.0371	371.81	0.0407
**2j**	418.9	2.5421	427.91	2.7614	428.14	2.7356	428.77	2.7038	428.05	2.7153
	346.5	0.6004	335.06	0.3460	333.23	0.3633	332.51	0.5444	332.64	0.4396
	335.0	0.8435	345.93	1.3944	345.74	1.5198	345.79	1.5648	345.63	1.5384
	362.3	0.3527	371.28	0.3136	375.40	0.2548	379.94	0.2115	377.16	0.2334

4. **Table S4.** $S_0 \rightarrow S_1$ excitation analysis in terms of HOMO-LUMO orbitals.

Comp.	Toluene		Transition	Percent
	Wavelength λ [nm]	Oscillator *f*		
**2a**	399.89	1.6108	HOMO → LUMO	95.8
**2b**	427.56	1.8728	HOMO-1 → LUMO+1	2.4
			HOMO → LUMO	94.8
**2c**	368.85	0.9737	HOMO-3 → LUMO+1	2.7
			HOMO-2 → LUMO	9.8
			HOMO-1 → LUMO+1	6.4
			HOMO → LUMO	78.0
**2d**	397.75	1.6228	HOMO → LUMO	93.8
**2e**	371.85	1.4274	HOMO-3 → LUMO+1	6.7
			HOMO-2 → LUMO	55.4
			HOMO-1 → LUMO+1	2.4
			HOMO → LUMO	30.6
**2f**	404.45	1.5611	HOMO-1 → LUMO+1	2.5
			HOMO → LUMO	94.4
**2g**	406.22	1.8375	HOMO-1 → LUMO+1	2.3
			HOMO → LUMO	94.8
**2h**	409.47	1.6025	HOMO-8 → LUMO	12.3
			HOMO-4 → LUMO	3.0
			HOMO-2 → LUMO	20.5
			HOMO → LUMO	58.4
**2i**	438.79	0.9983	HOMO-8 →LUMO	3.5
			HOMO-1 → LUMO+3	3.7
			HOMO → LUMO	88.7
**2j**	427.91	2.7614	HOMO-1 → LUMO+1	3.2
			HOMO → LUMO	91.7

5. **Table S5.** $S_0 \rightarrow S_1$ excitation analysis in terms of Natural Transition Orbitals.

Comp.	λ [nm]	particle/hole occupations	NTO transition
**2a**	399.89	0.9654	HONTO→LUNTO
**2b**	427.56	0.9556	HONTO→LUNTO
**2c**	445.10	0.1057	HONTO-1→LUNTO+1
		0.8920	HONTO→LUNTO
**2d**	397.75	0.9704	HONTO→LUNTO
**2e**	399.20	0.8910	HONTO-1→LUNTO+1
		0.1071	HONTO→LUNTO
**2f**	404.45	0.9566	HONTO→LUNTO
**2g**	406.22	0.9593	HONTO→LUNTO
**2h**	409.47	0.9527	HONTO→LUNTO
**2i**	438.79	0.9416	HONTO→LUNTO
**2j**	427.91	0.9409	HONTO→LUNTO

6. HOMO and LUMO orbitals calculated within PBE0/6-31G* level of theory with GD3 empirical dispersion

HOMO LUMO

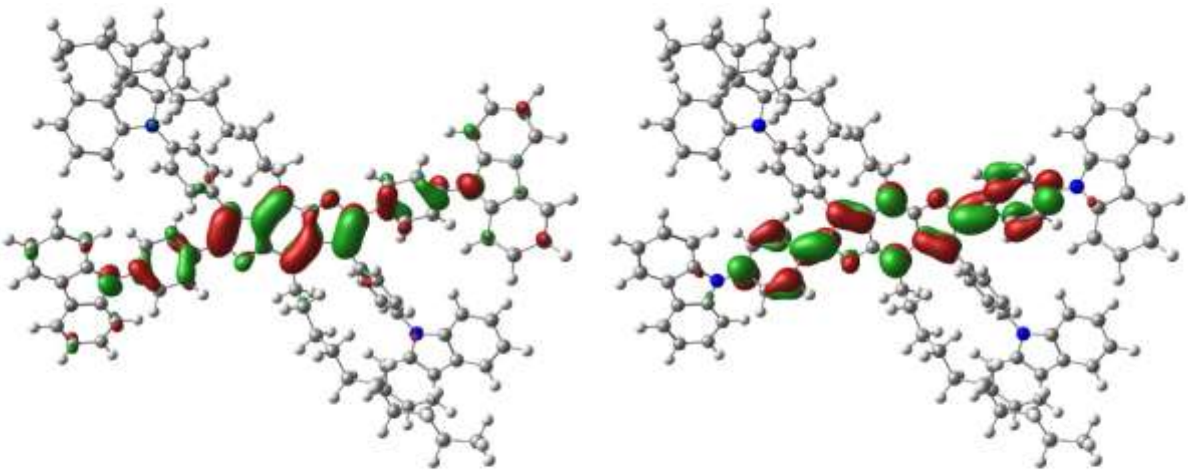

Fig. S23. Graphical representation of the HOMO and LUMO orbitals of **2a** in ground state.

HOMO LUMO

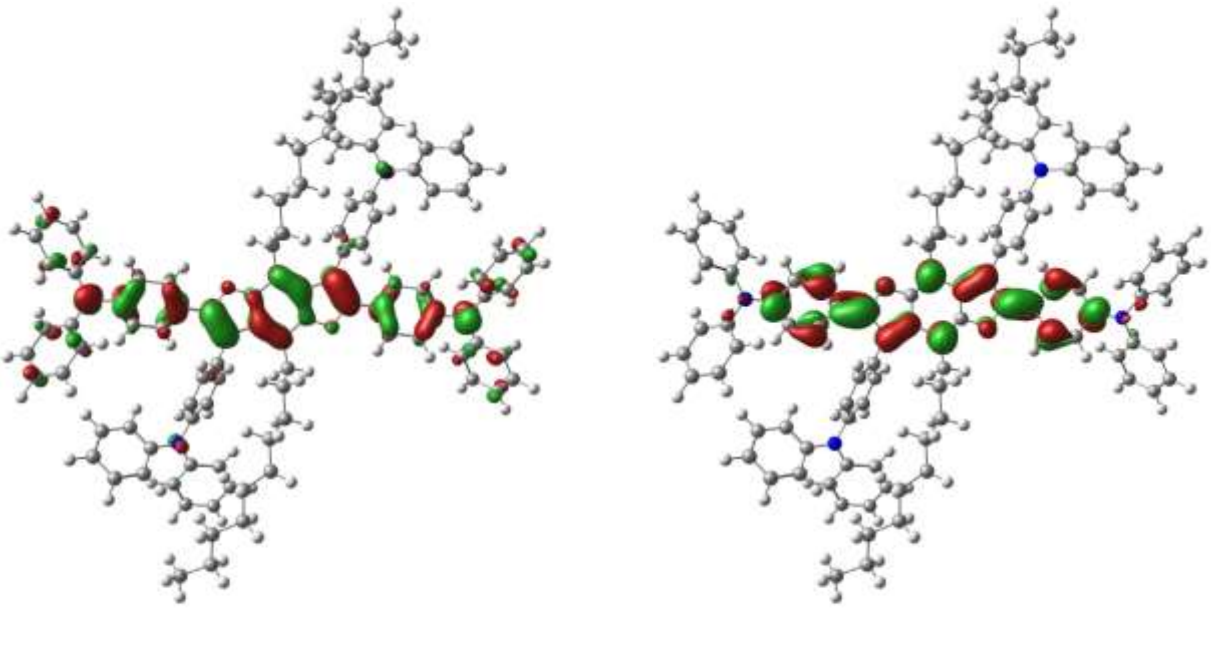

Fig. S24. Graphical representation of HOMO and LUMO orbitals of **2b** in ground state.

HOMO LUMO

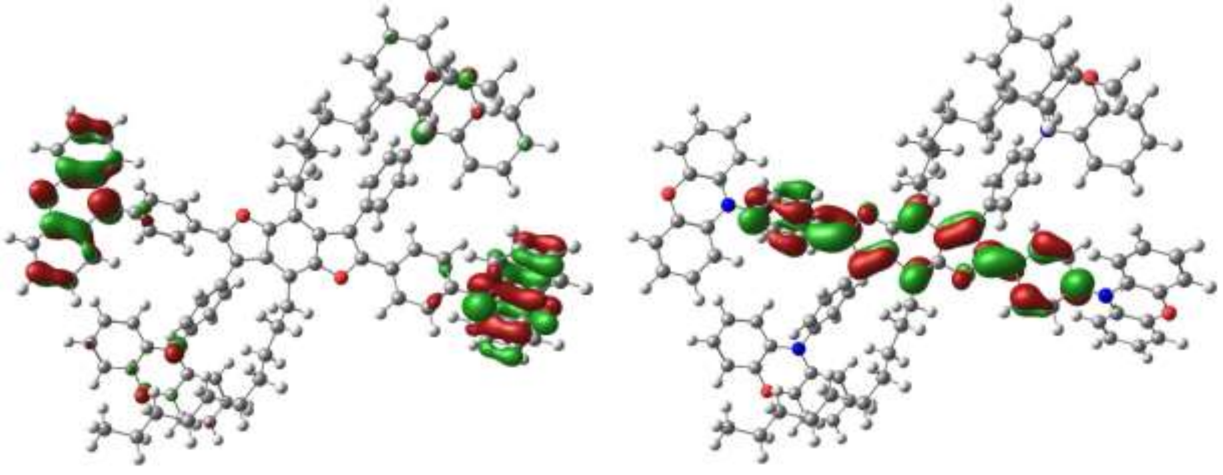

Fig. S25. Graphical representation of HOMO and LUMO orbitals of **2c** in ground state.

HOMO LUMO

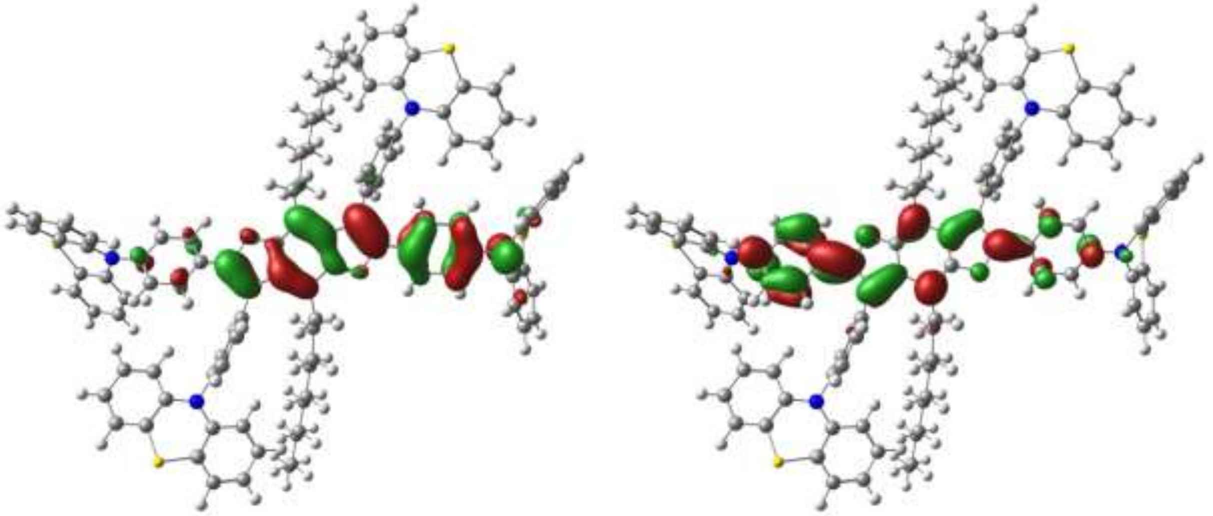

Fig. S26. Graphical representation of HOMO and LUMO orbitals of **2d** in ground state.

HOMO LUMO

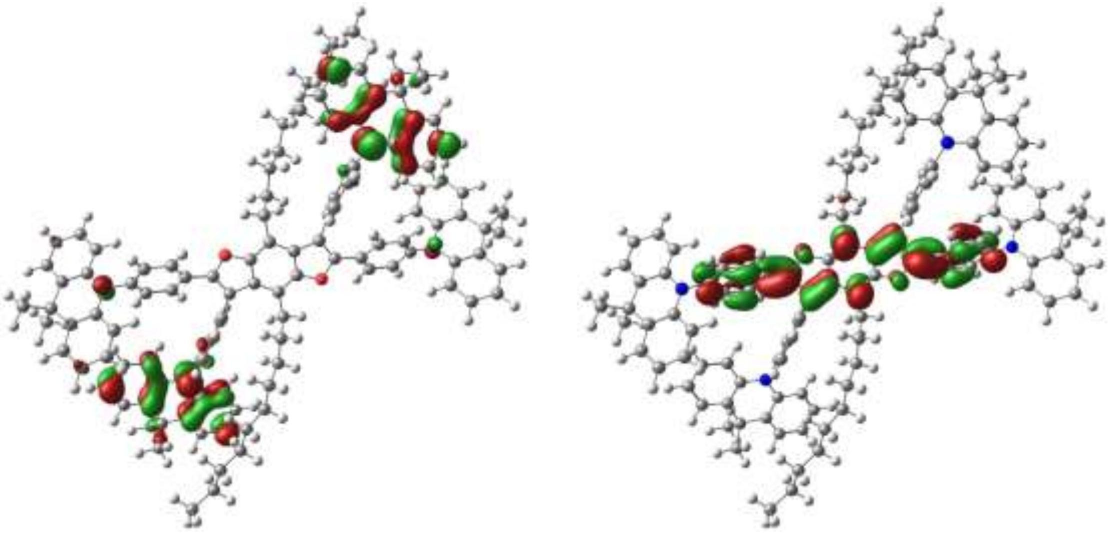

Fig. S27. Graphical representation of HOMO and LUMO orbitals of **2e** in ground state.

HOMO LUMO

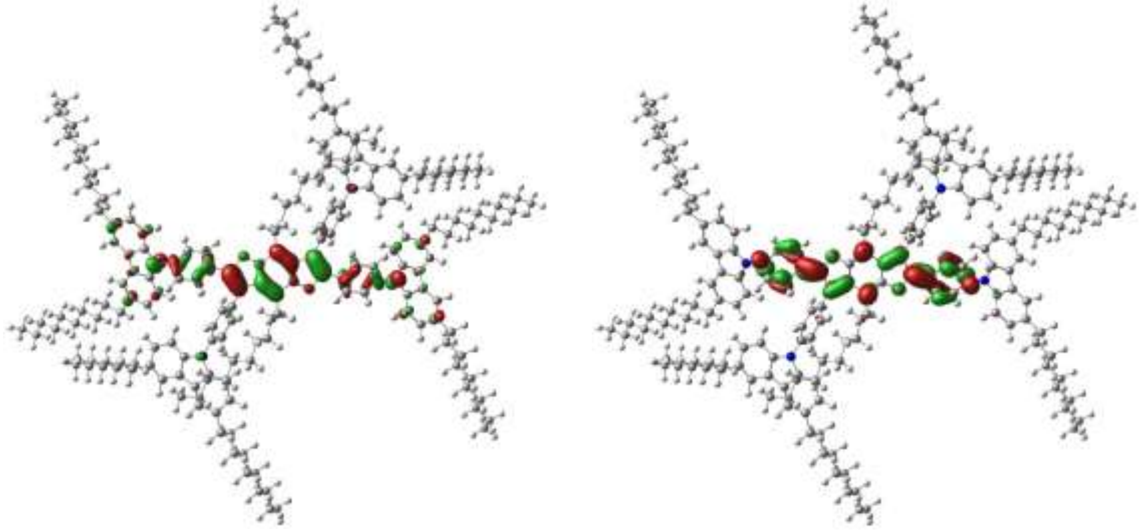

Fig. S28. Graphical representation of HOMO and LUMO orbitals of **2f** in ground state.

HOMO LUMO

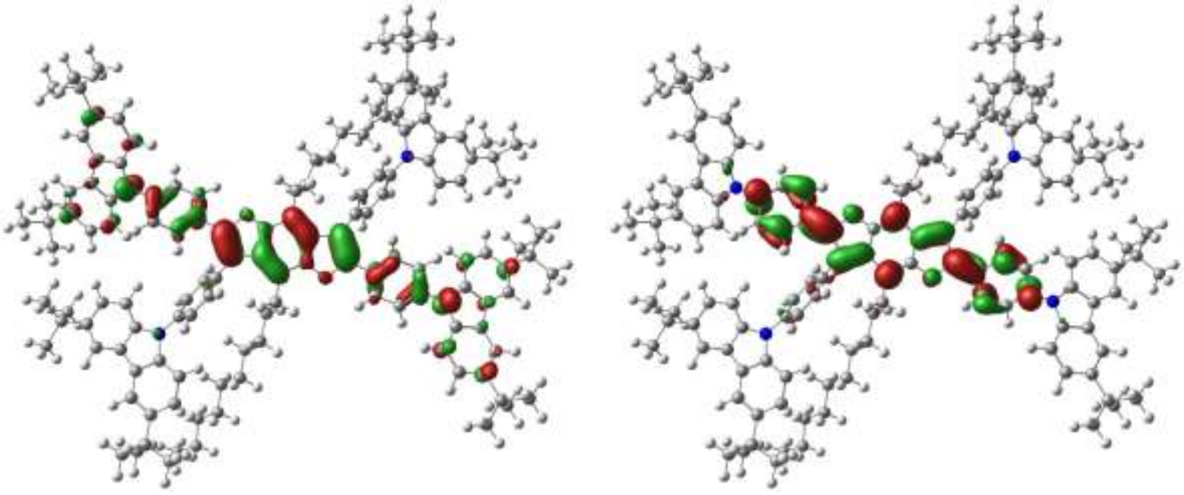

Fig. S29. Graphical representation of HOMO and LUMO orbitals of **2g** in ground state.

HOMO LUMO

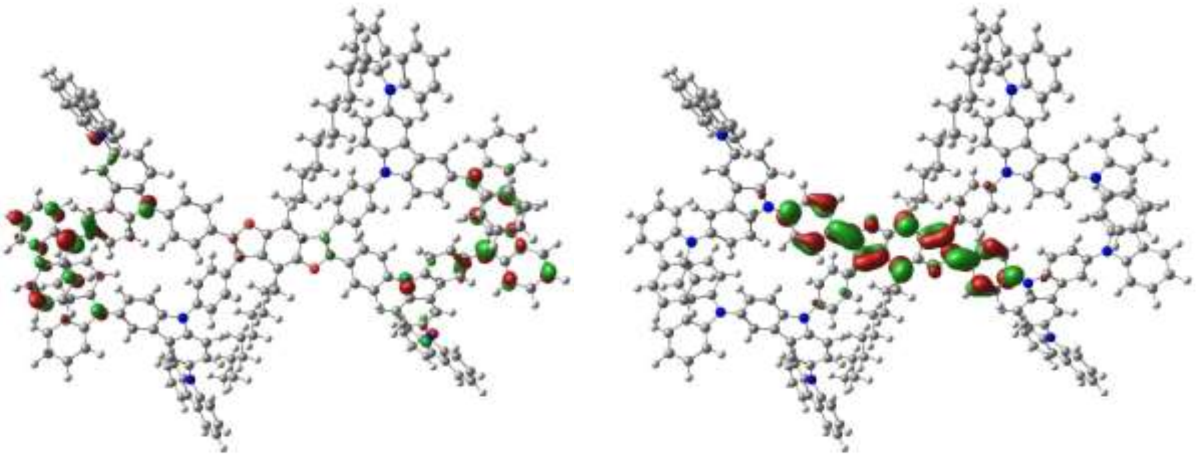

Fig. S30. Graphical representation of HOMO and LUMO orbitals of **2h** in ground state.

HOMO LUMO

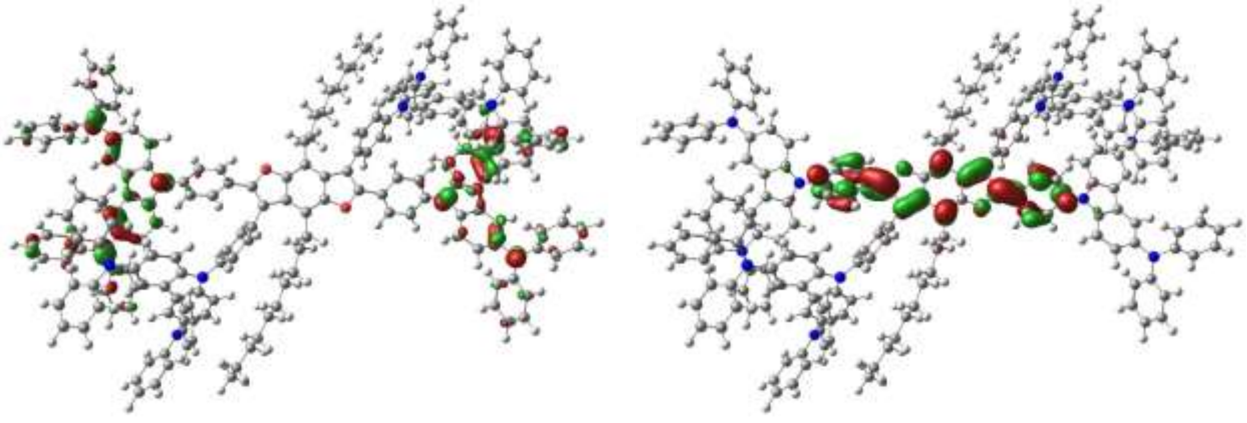

Fig. S31. Graphical representation of HOMO and LUMO orbitals of **2i** in ground state.

HOMO LUMO

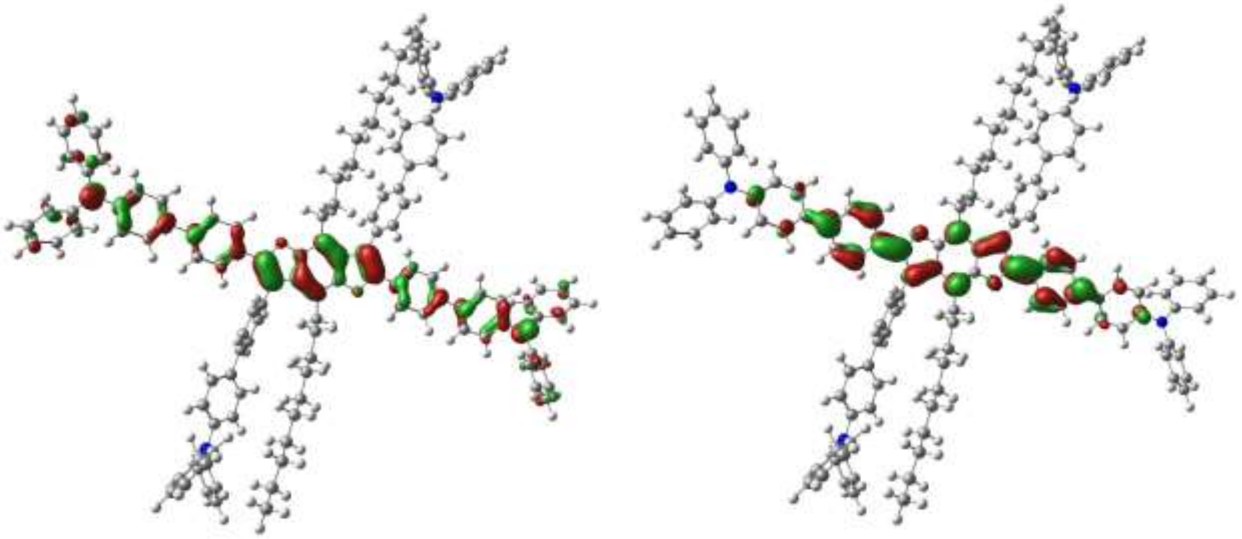

Fig. S32. Graphical representation of HOMO and LUMO orbitals of **2j** in ground state.

7. Natural Transition Orbitals for 2a-2j derived for $S_0 \rightarrow S_1$ excitation.

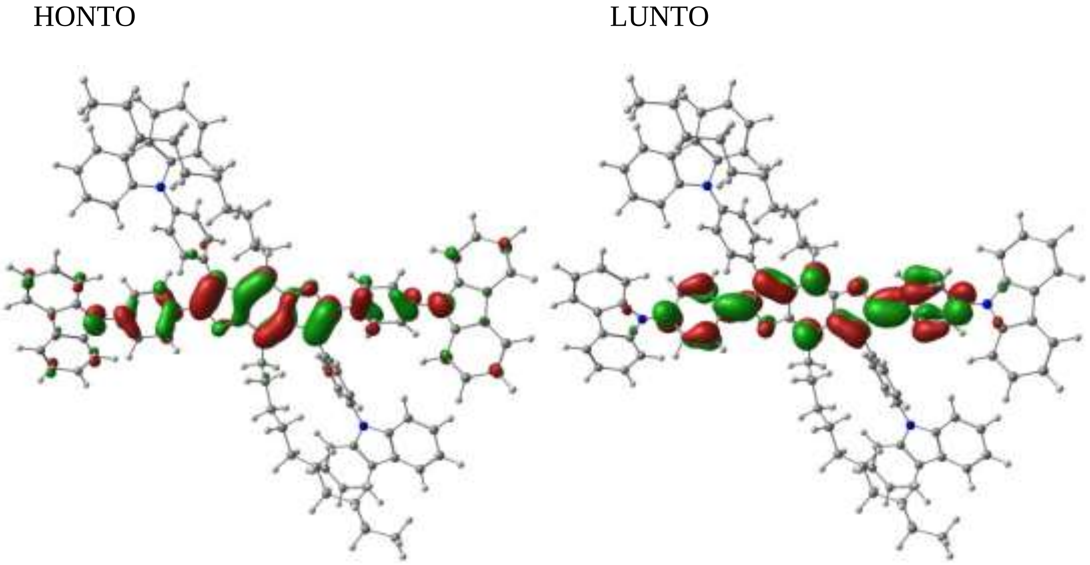

Fig. S33. Graphical representation of HONTO and LUNTO orbitals of **2a** for $S_0 \rightarrow S_1$ excitation.

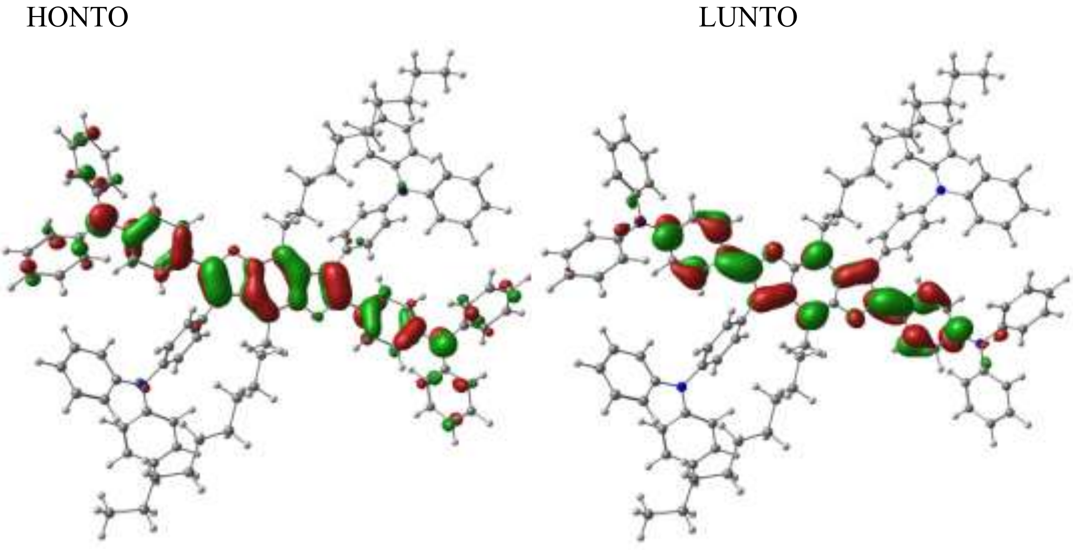

Fig. S34. Graphical representation of HONTO and LUNTO orbitals of **2b** for $S_0 \rightarrow S_1$ excitation.

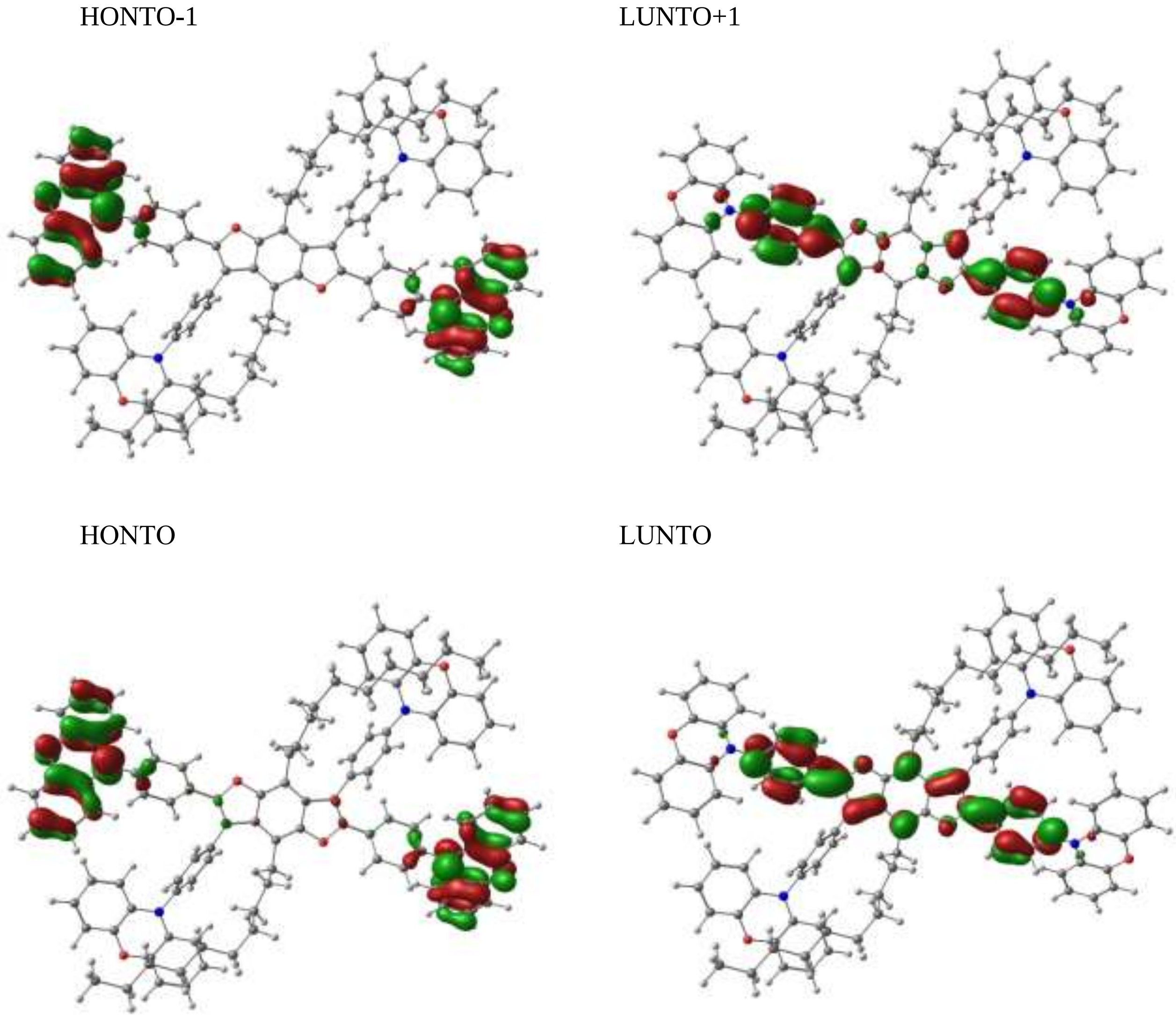

Fig. S35. Graphical representations of HONTO-1, HONTO and LUNTO+1, LUNTO orbitals of **2c** for $S_0 \rightarrow S_1$ excitation.

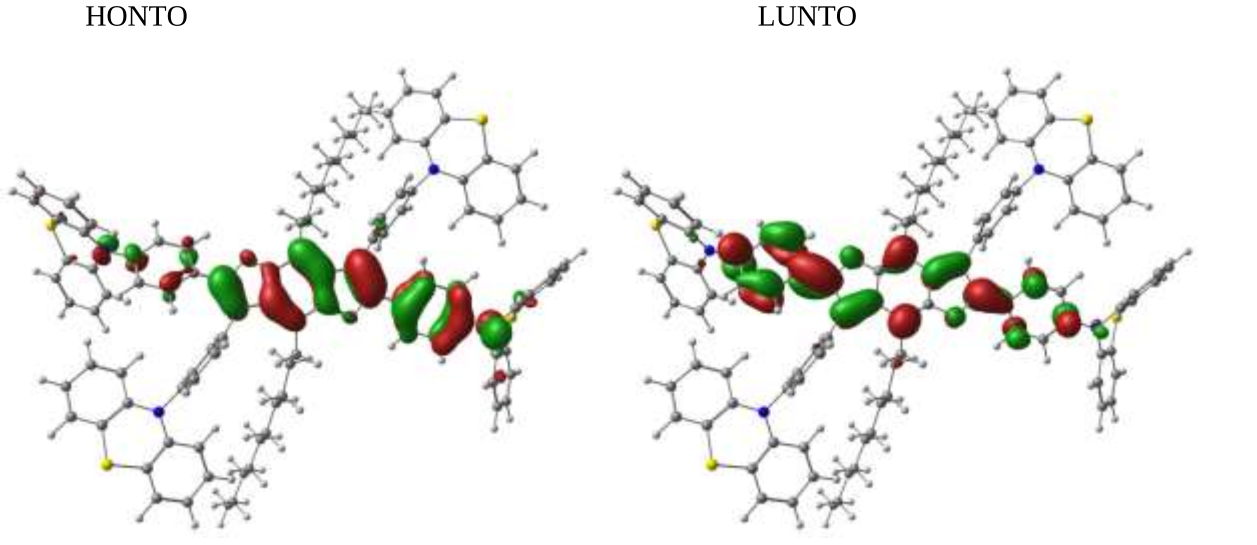

Fig. S36. Graphical representation of HONTO and LUNTO orbitals of **2d** for $S_0 \rightarrow S_1$ excitation.

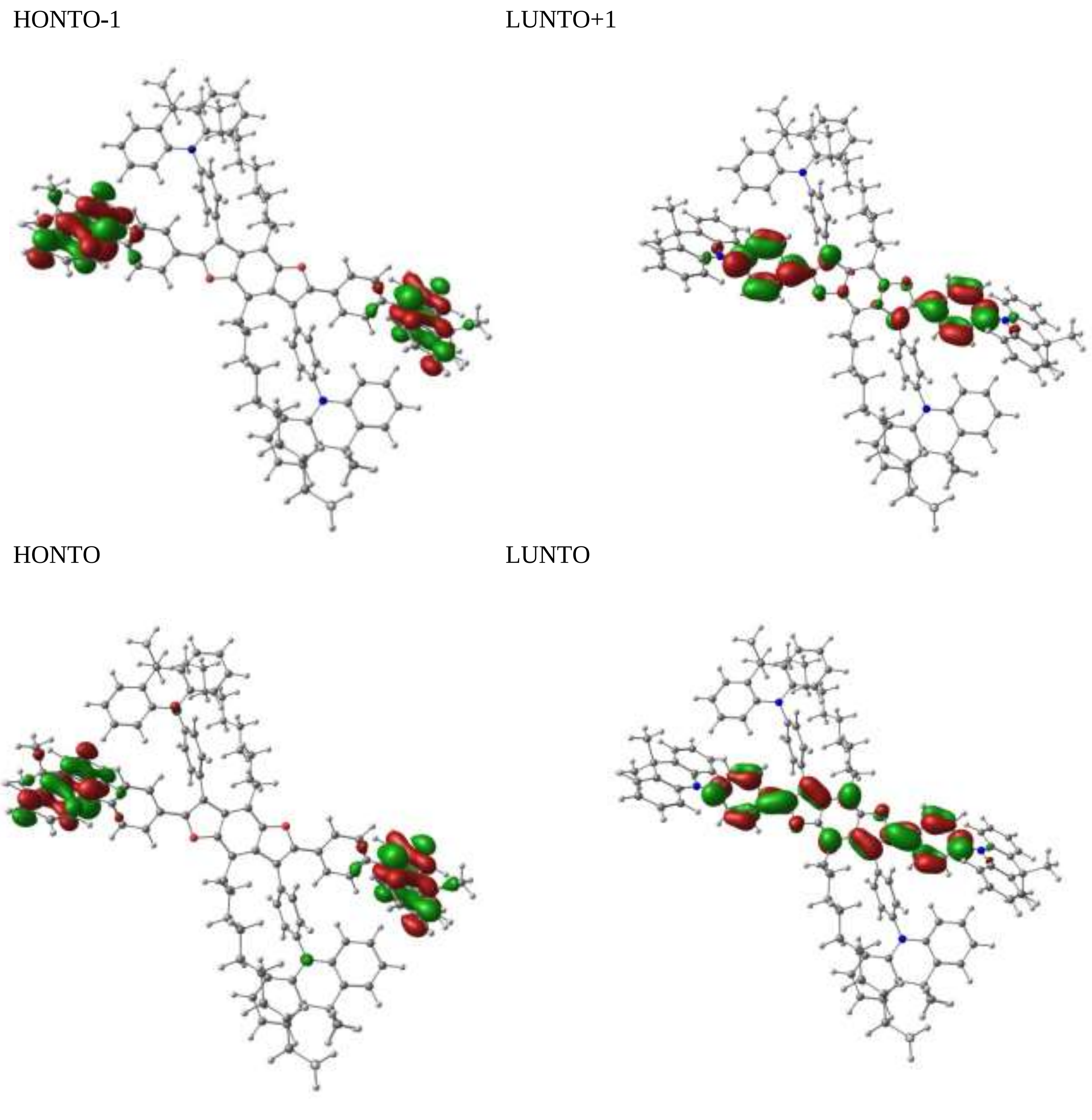

Fig. S37. Graphical representations of HONTO and LUNTO orbitals of **2e** for $S_0 \rightarrow S_1$ excitation.

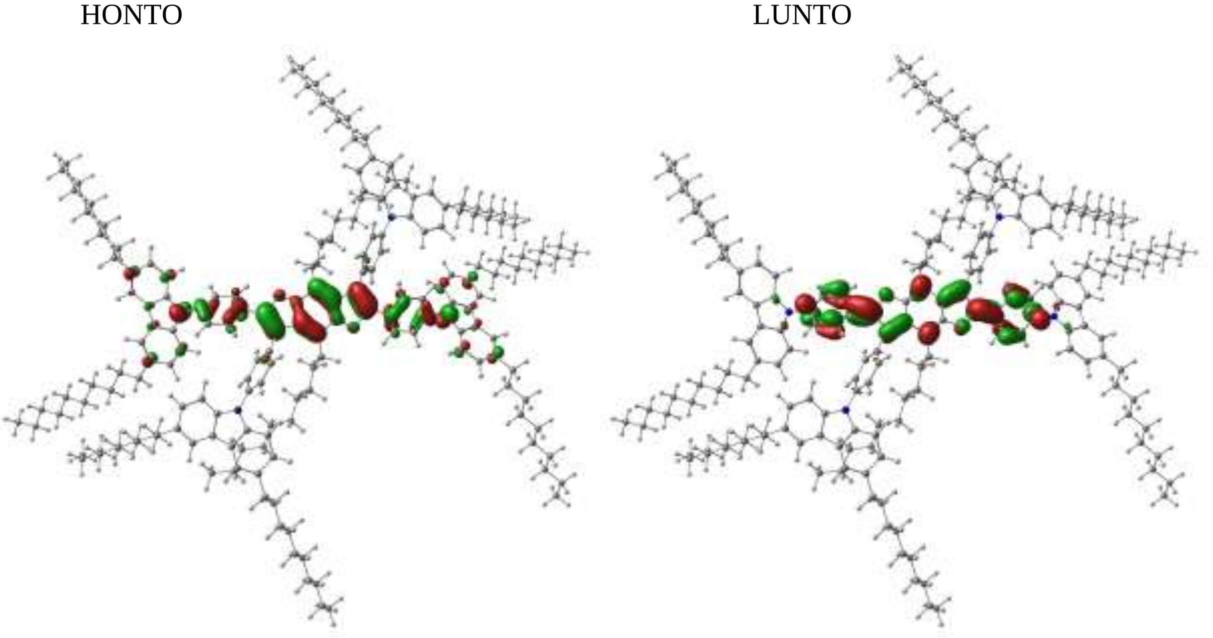

Fig. S38. Graphical representation of HONTO and LUNTO orbitals of **2f** for $S_0 \rightarrow S_1$ excitation.

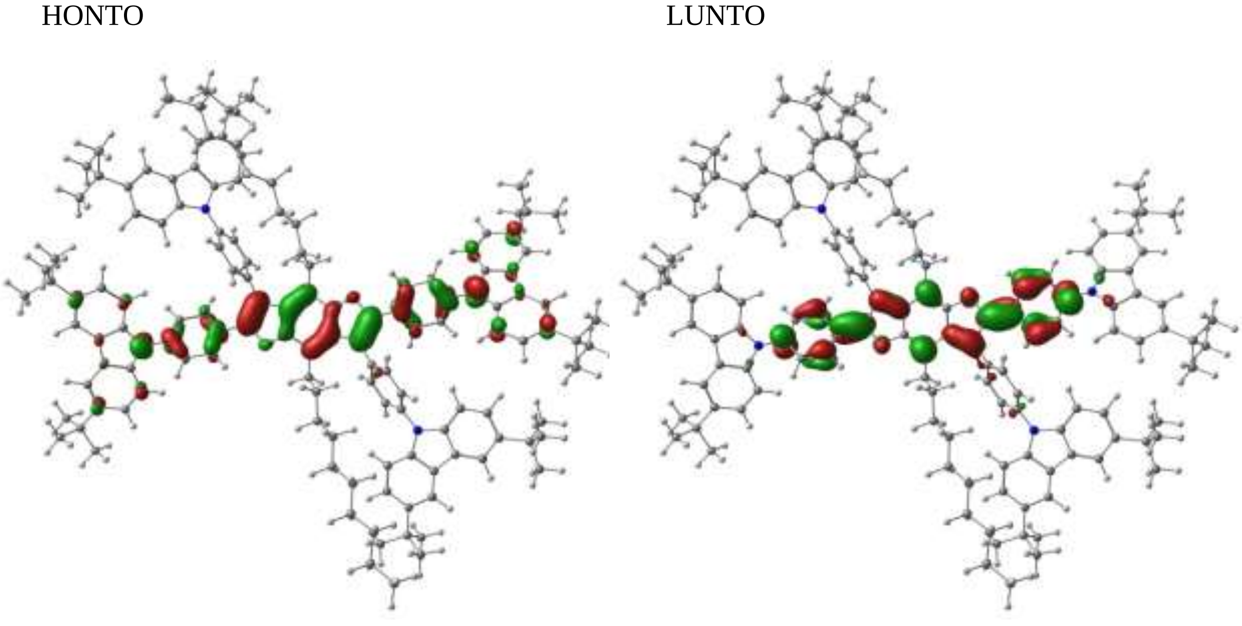

Fig. S39. Graphical representation of HONTO and LUNTO orbitals of **2g** for $S_0 \rightarrow S_1$ excitation.

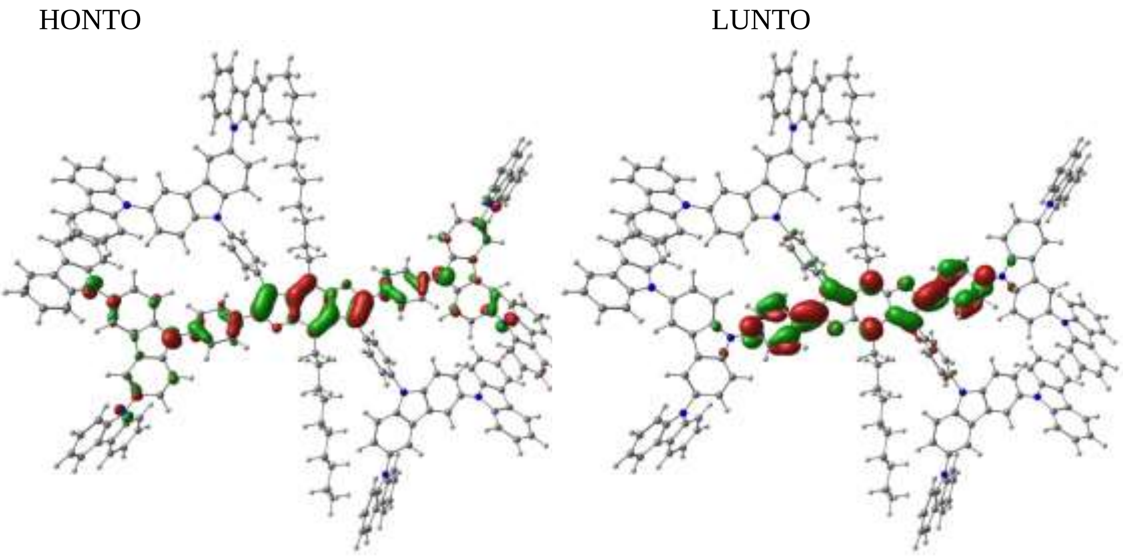

Fig. S40. Graphical representation of HONTO and LUNTO orbitals of **2h** for $S_0 \rightarrow S_1$ excitation.

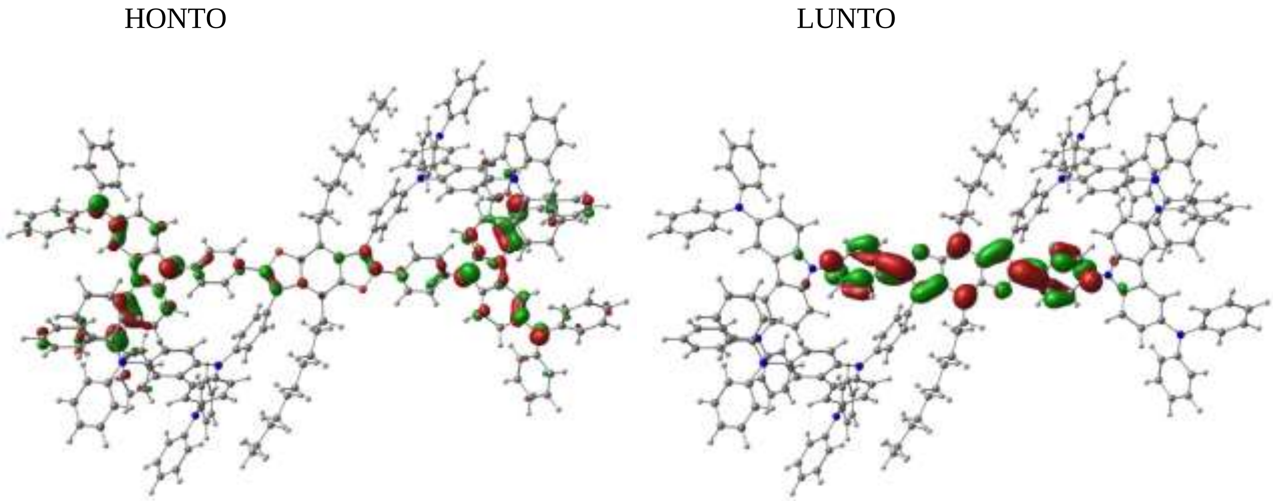

Fig. S41. Graphical representation of HONTO and LUNTO orbitals of **2i** for $S_0 \rightarrow S_1$ excitation.

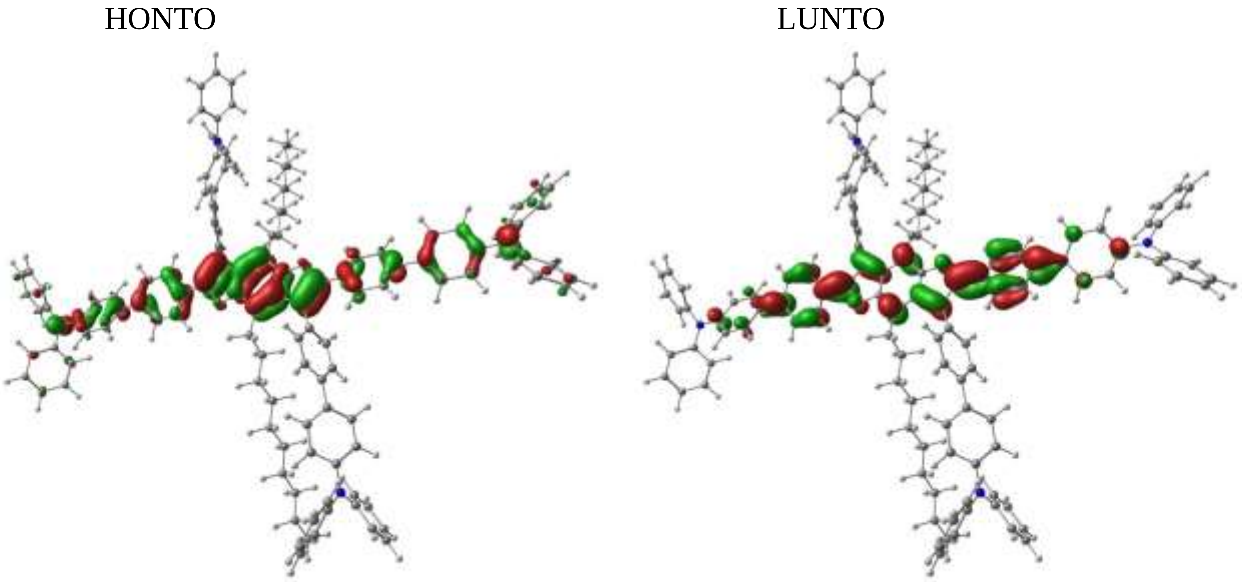

Fig. S42. Graphical representation of HONTO and LUNTO orbitals of **2j** for $S_0 \rightarrow S_1$ excitation.

8. 1H NMR and 13C{1H} NMR spectra

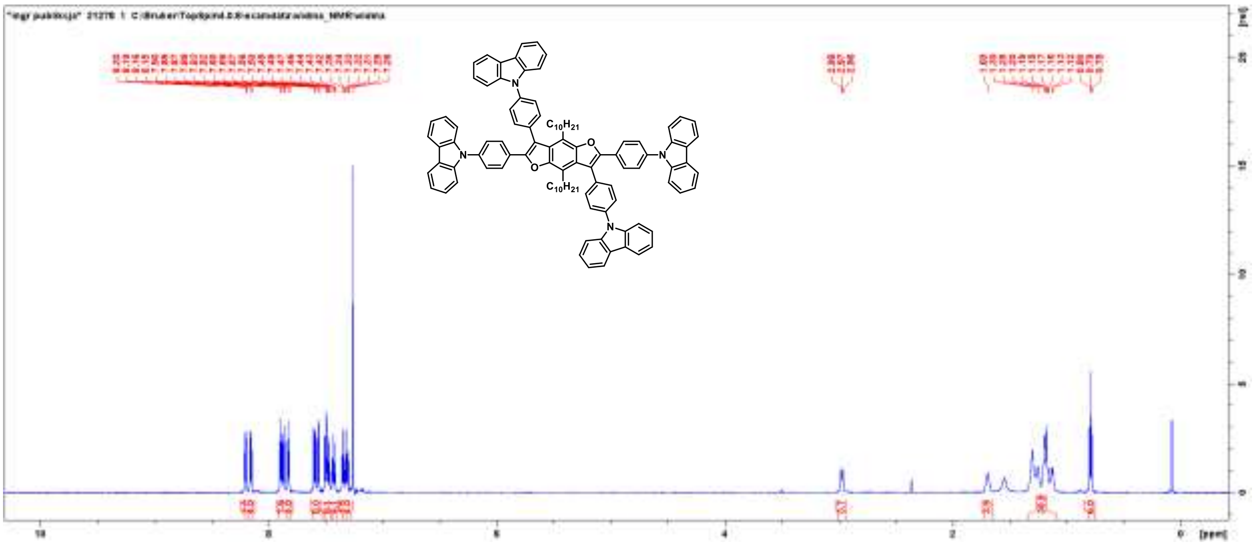

Fig. S43. ^{1}H NMR ($CDCl_3$, 700 MHz) spectrum of **2a**.

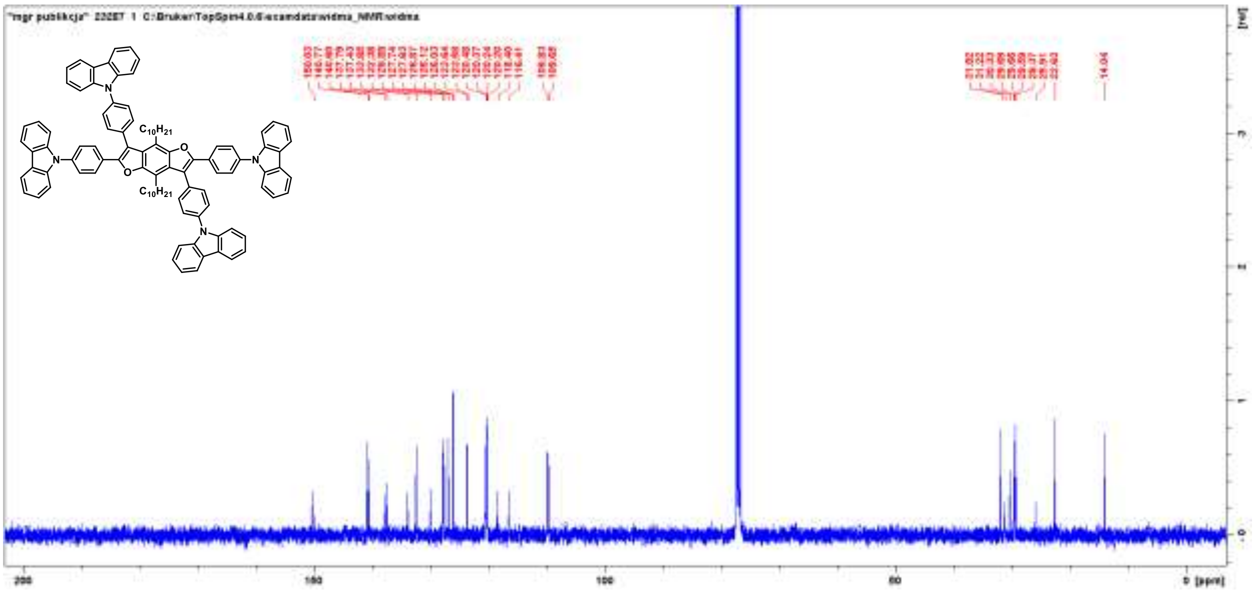

Fig. S44. $^{13}C\{^{1}H\}$ NMR ($CDCl_3$, 100 MHz) spectrum of **2a**.

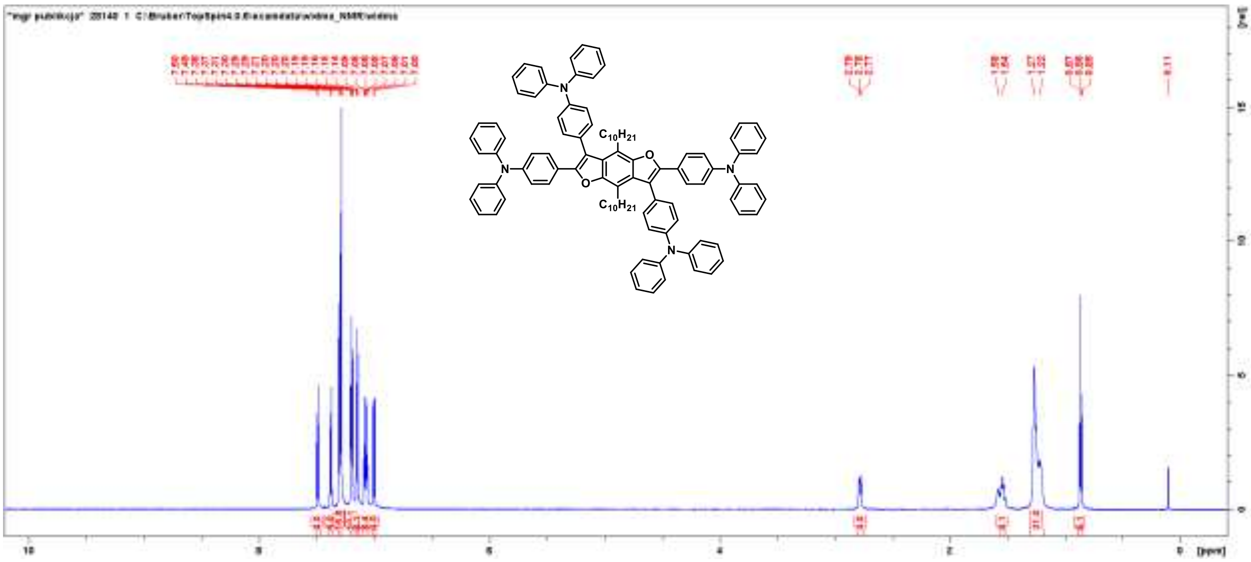

Fig. S45. ^{1}H NMR ($CDCl_3$, 700 MHz) spectrum of **2b**.

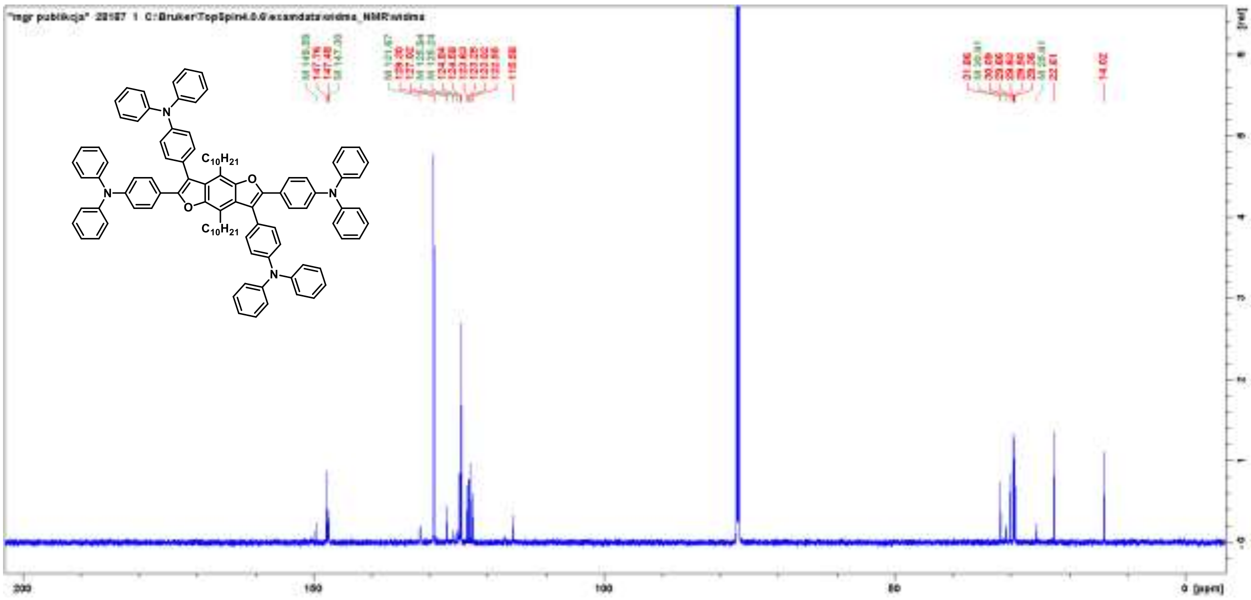

Fig. S46. $^{13}C\{^{1}H\}$ NMR ($CDCl_3$, 100 MHz) spectrum of **2b**.

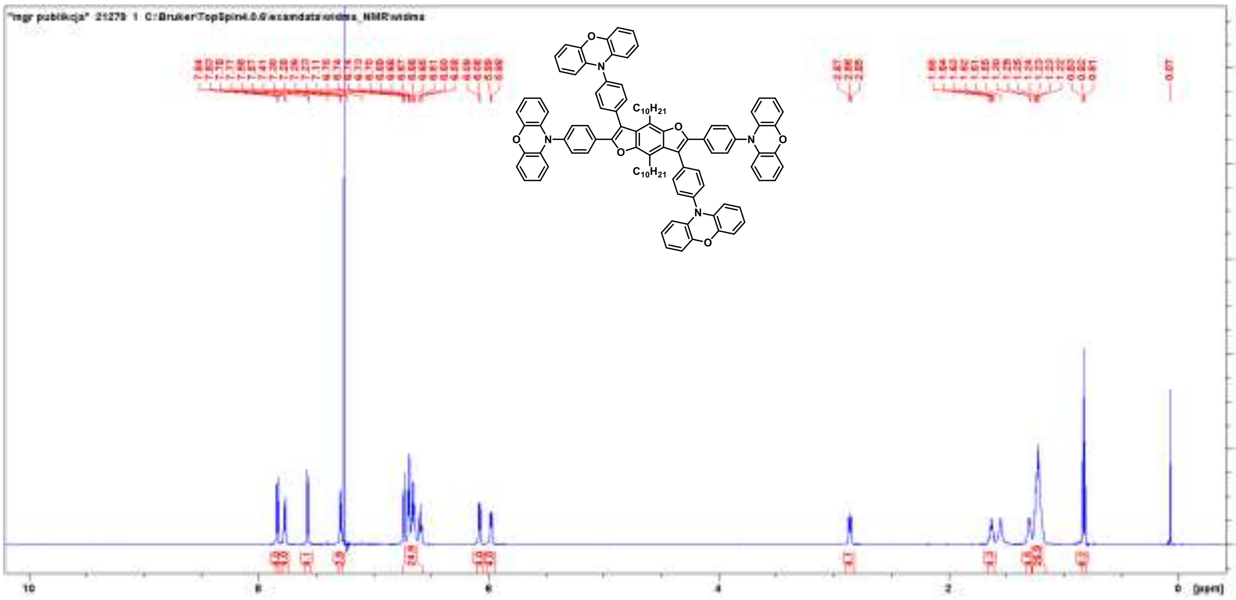

Fig. S47. ^{1}H NMR ($CDCl_3$, 700 MHz) spectrum of **2c**.

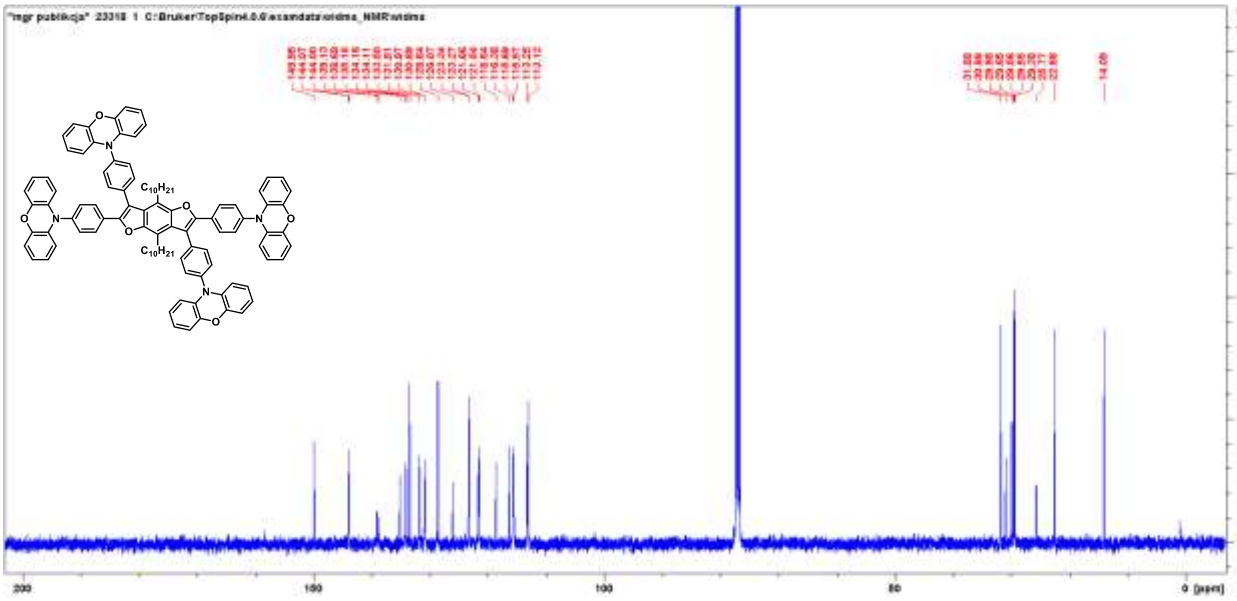

Fig. S48. $^{13}C\{^{1}H\}$ NMR ($CDCl_3$, 100 MHz) spectrum of **2c**.

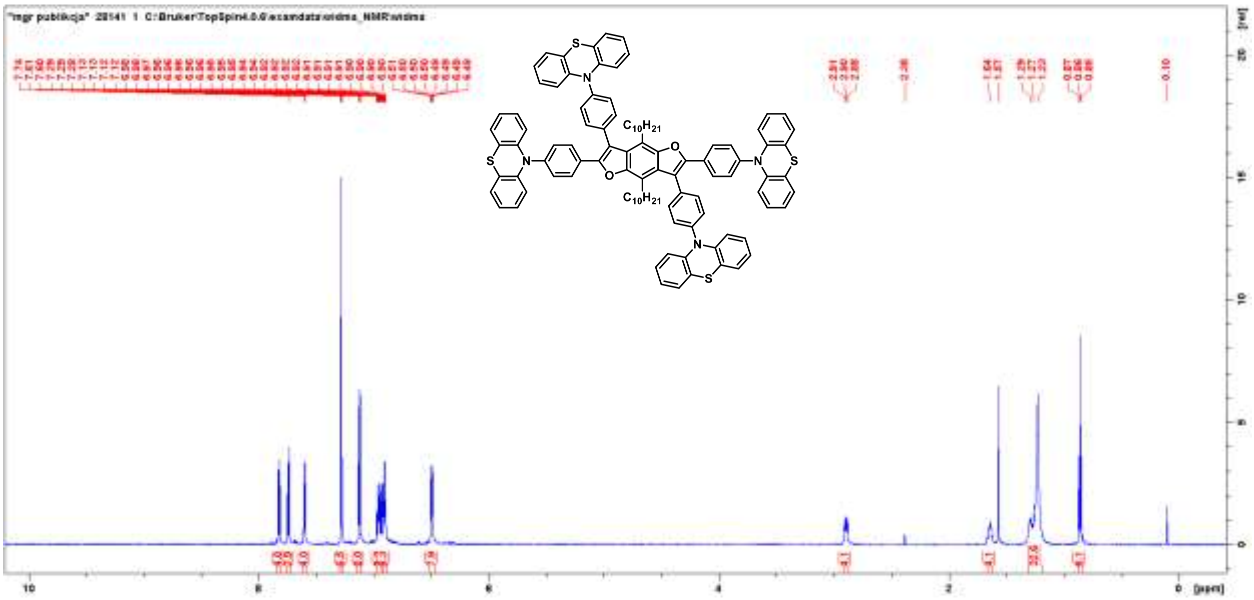

Fig. S49. ^{1}H NMR ($CDCl_3$, 700 MHz) spectrum of **2d**.

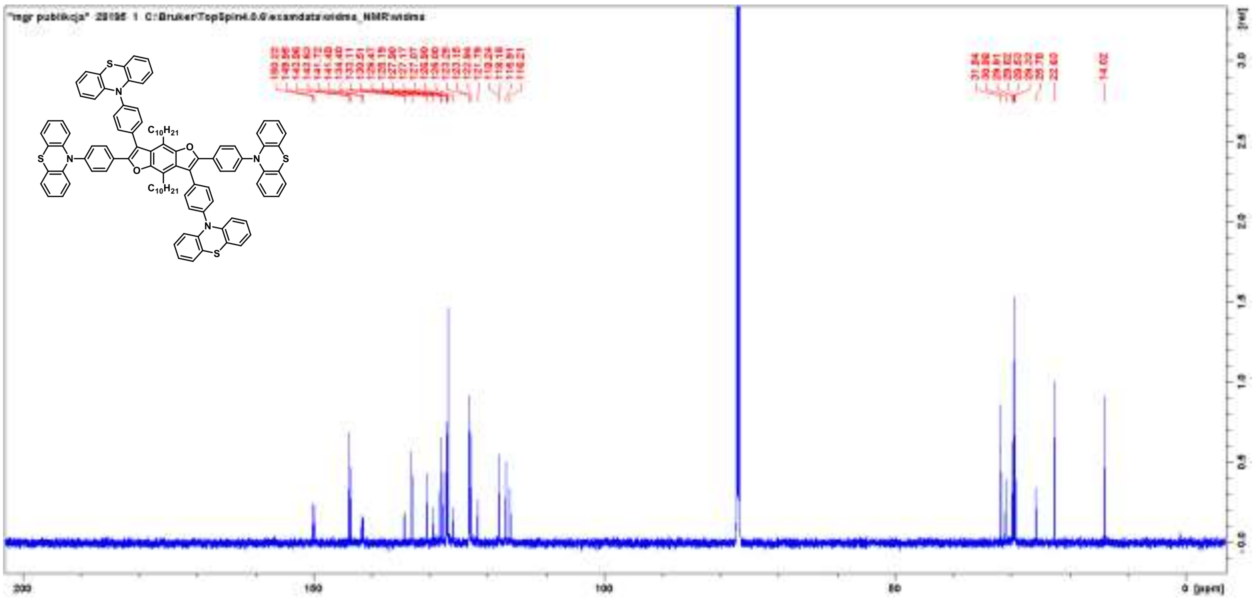

Fig. S50. $^{13}C\{^{1}H\}$ NMR ($CDCl_3$, 175 MHz) spectrum of **2d**.

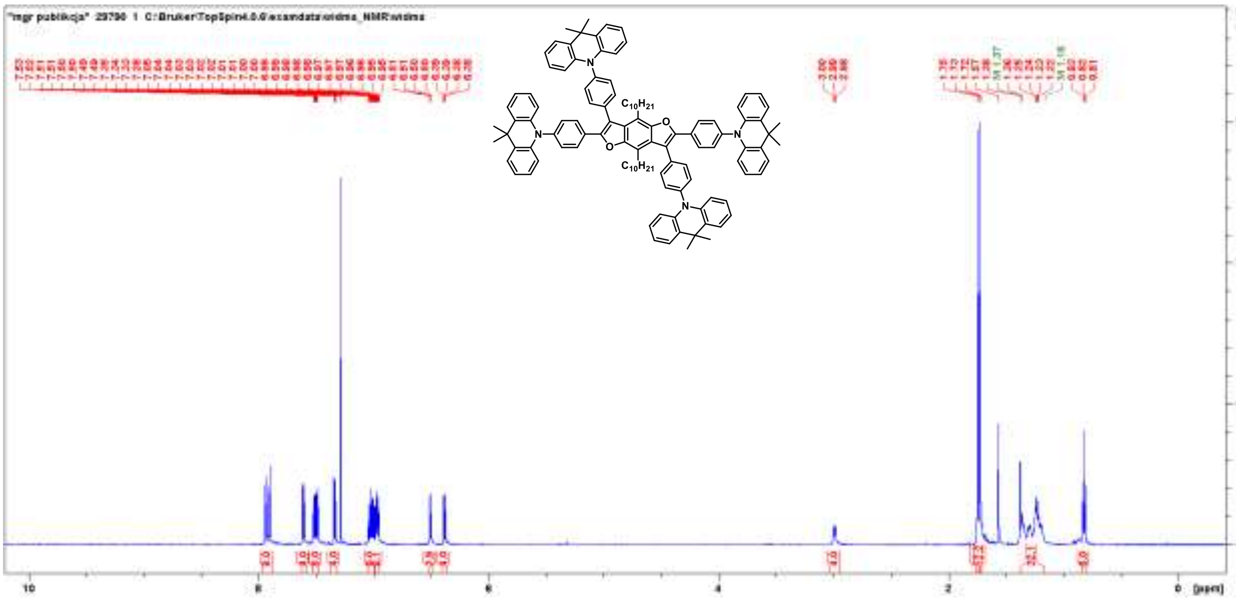

Fig. S51. ^{1}H NMR ($CDCl_3$, 700 MHz) spectrum of **2e**.

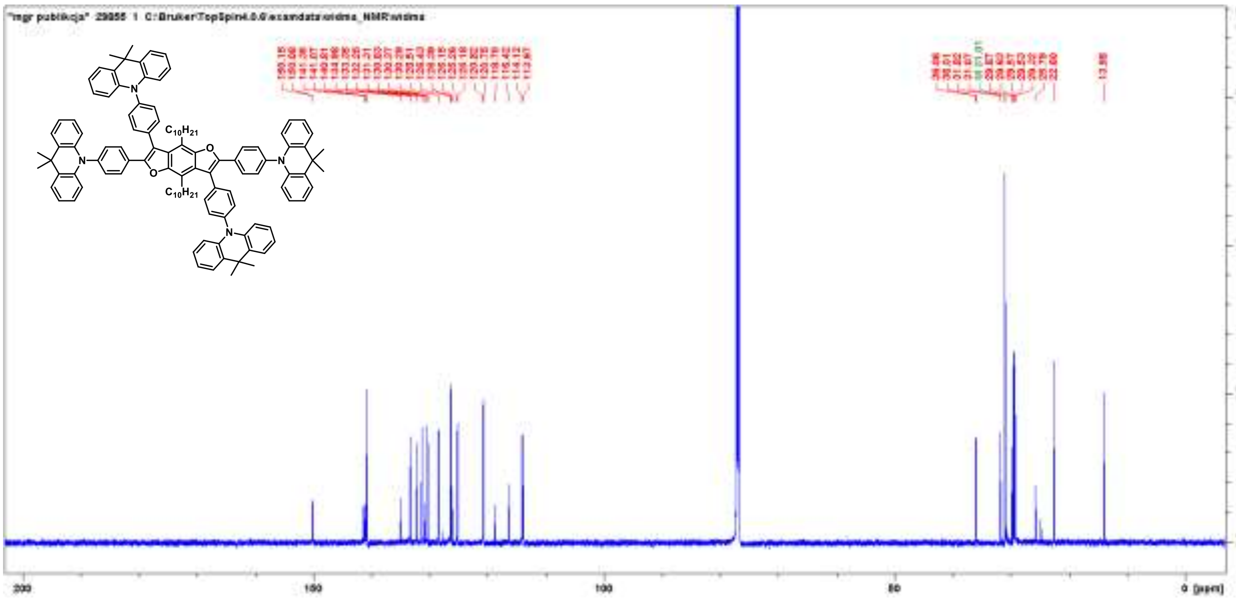

Fig. S52. $^{13}C\{^{1}H\}$ NMR ($CDCl_3$, 175 MHz) spectrum of **2e**.

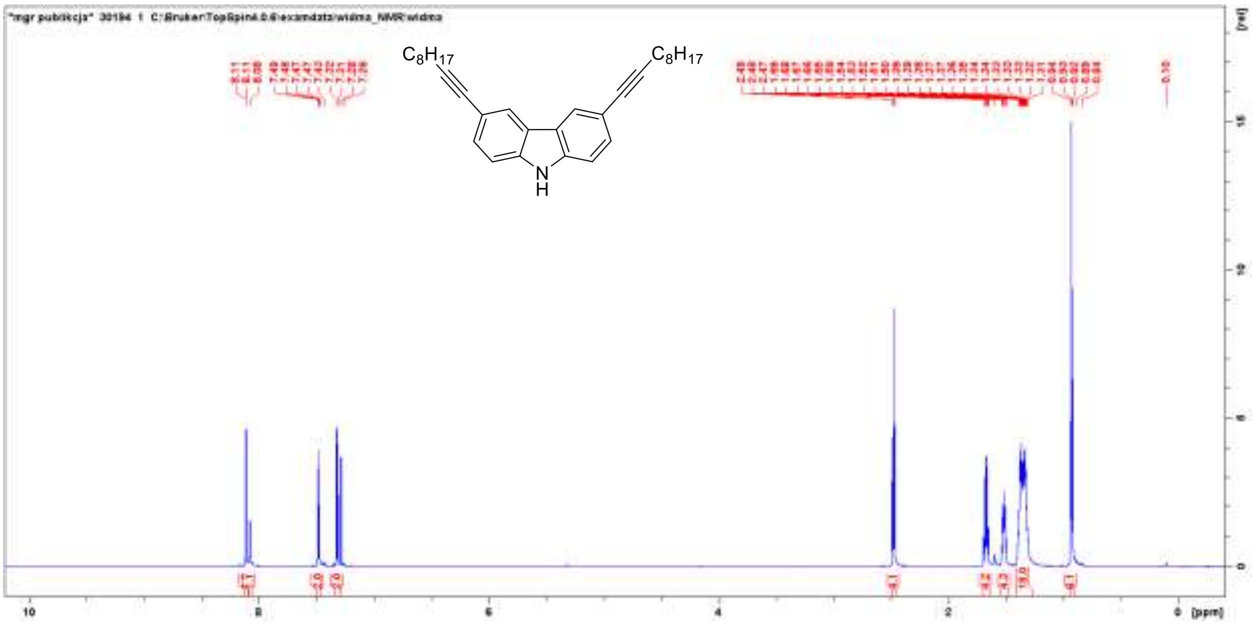

Fig. S53. ^{1}H NMR ($CDCl_3$, 700 MHz) spectrum of 3,6-di(dec-1-yn-1-yl)-9*H*-carbazole.

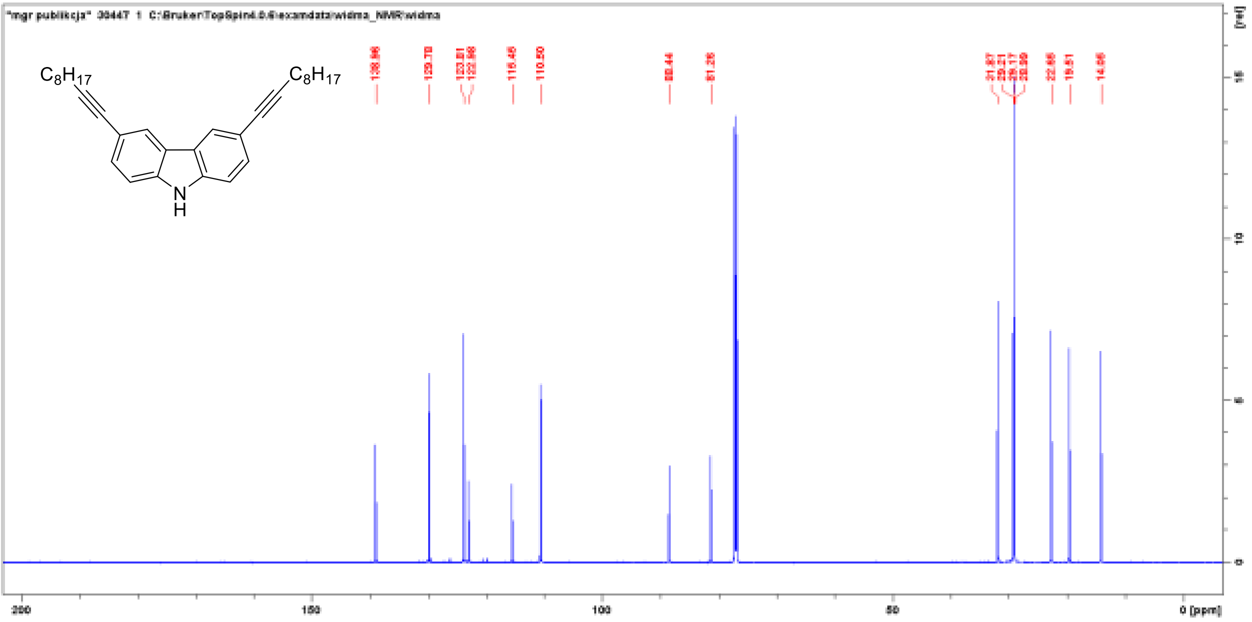

Fig. S54. $^{13}C\{^1H\}$ NMR ($CDCl_3$, 175 MHz) spectrum of 3,6-di(dec-1-yn-1-yl)-9*H*-carbazole.

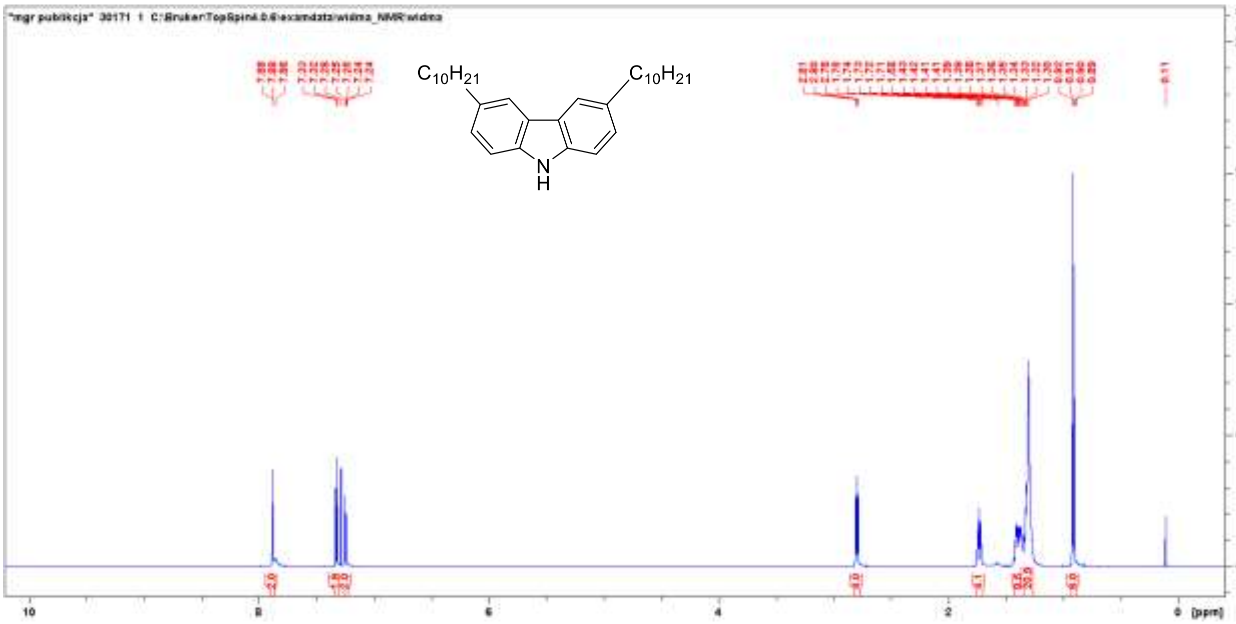

Fig. S55. ^{1}H NMR ($CDCl_3$, 700 MHz) spectrum of **1a**.

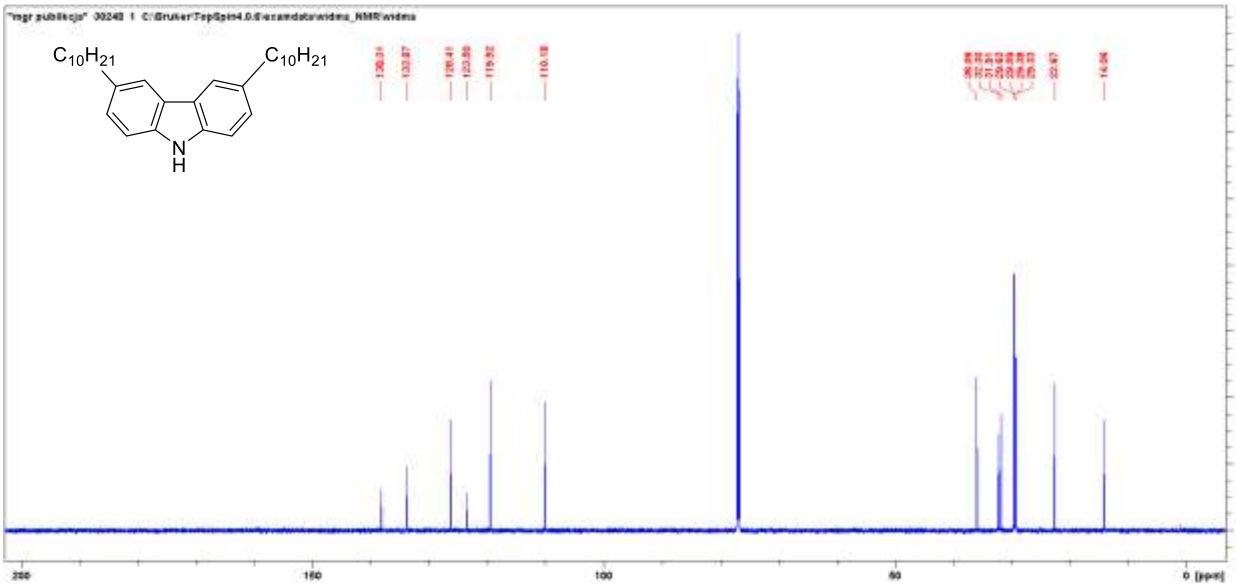

Fig. S56. $^{13}C\{^{1}H\}$ NMR ($CDCl_3$, 175 MHz) spectrum of **1a**.

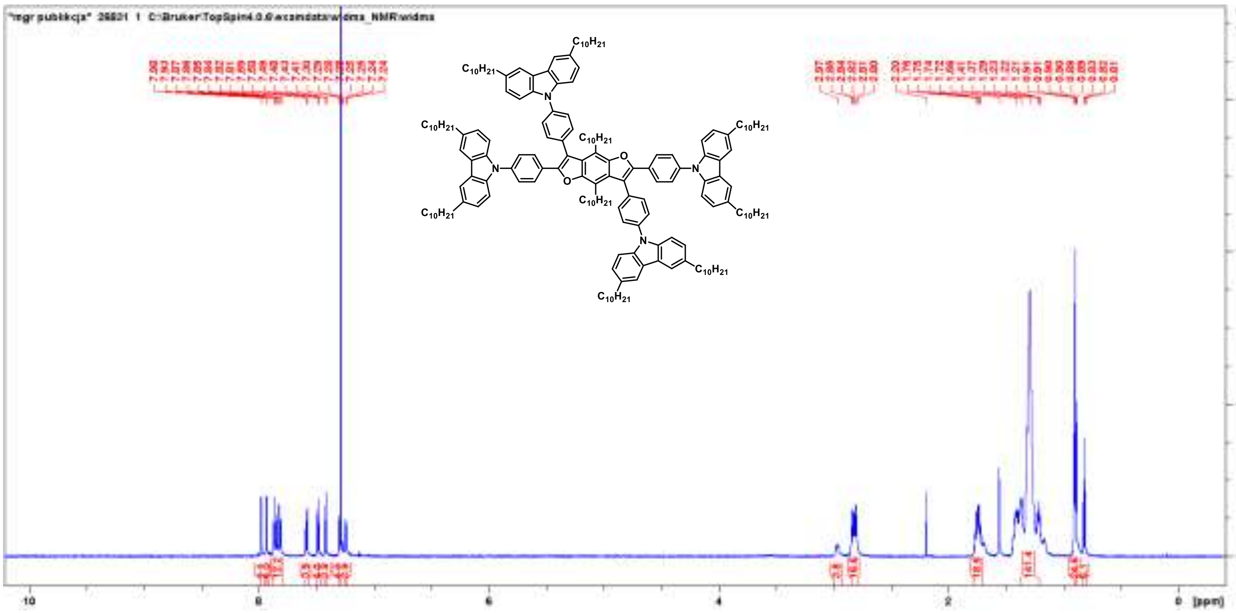

Fig. S57. ^{1}H NMR ($CDCl_3$, 700 MHz) spectrum of **2f**.

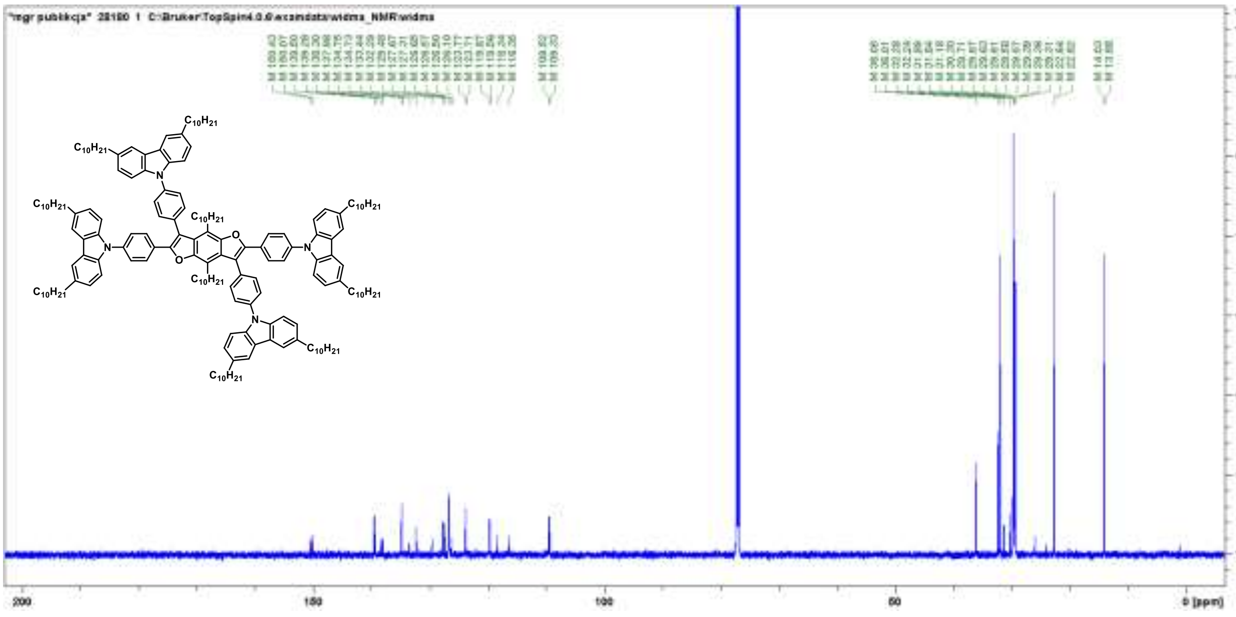

Fig. S58. $^{13}C\{^{1}H\}$ NMR ($CDCl_3$, 175 MHz) spectrum of **2f**.

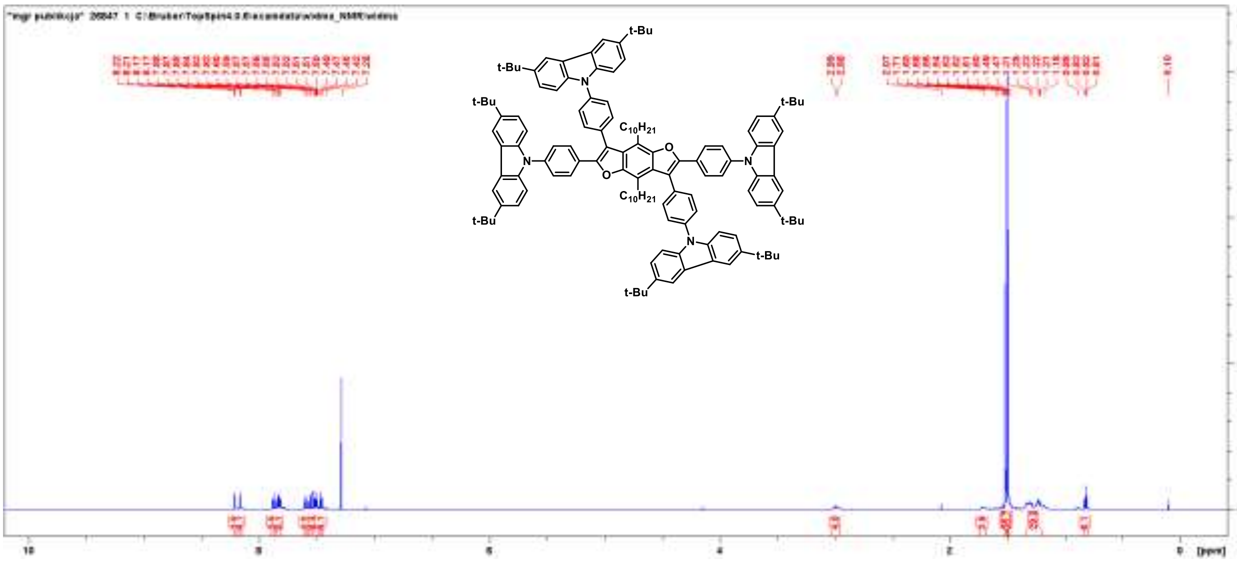

Fig. S59. ^{1}H NMR ($CDCl_3$, 700 MHz) spectrum of **2g**.

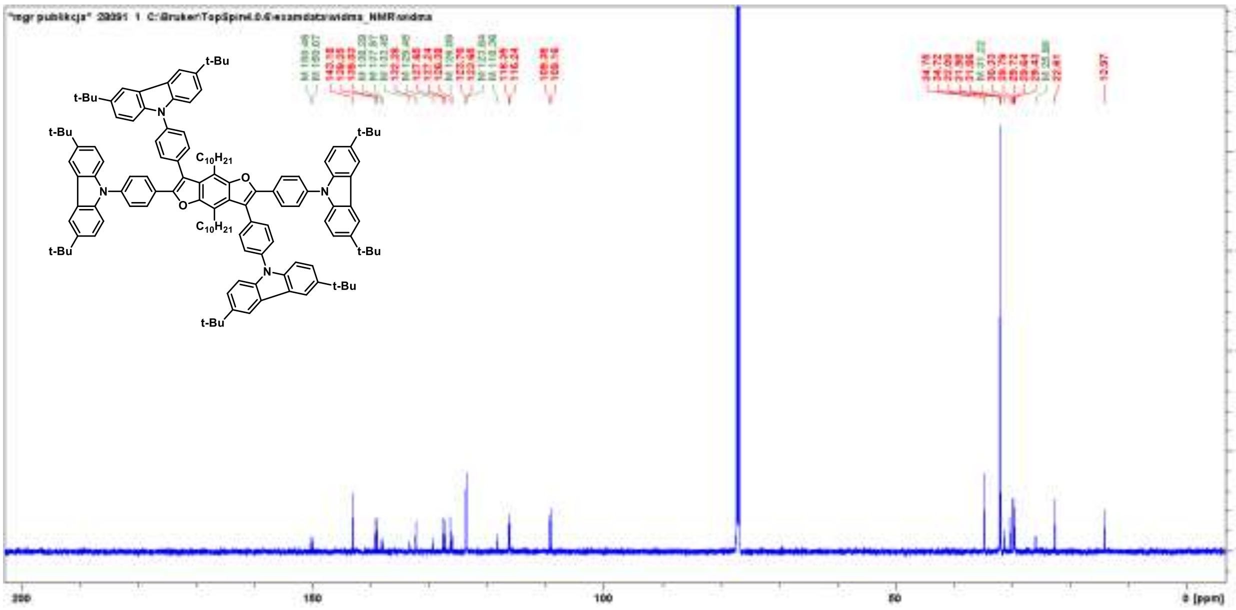

Fig. S60. $^{13}C\{^{1}H\}$ NMR ($CDCl_3$, 175 MHz) spectrum of **2g**.

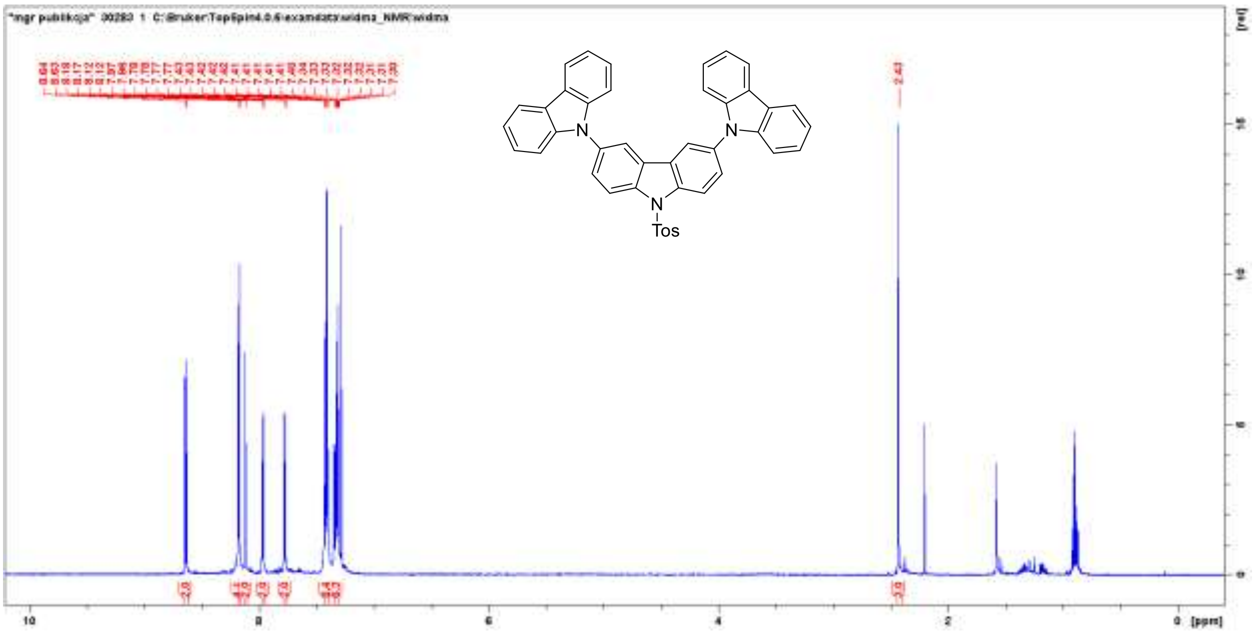

Fig. S61. ^{1}H NMR ($CDCl_3$, 700 MHz) spectrum of 9'-tosyl-9'*H*-9,3':6',9''-tercarbazole.

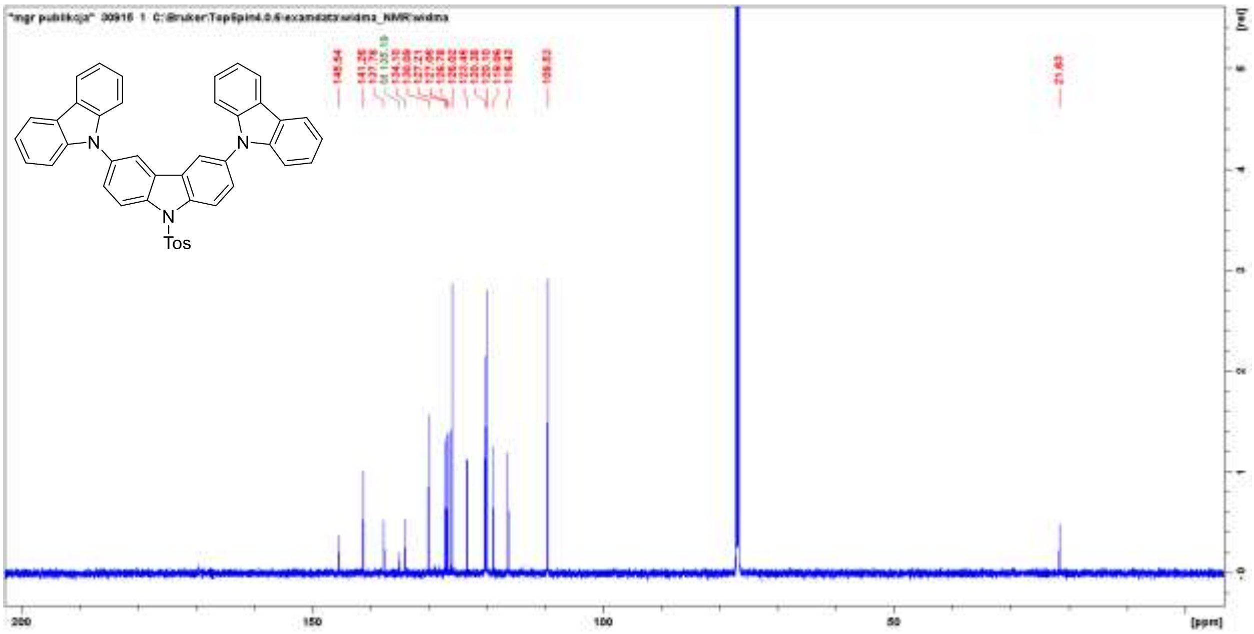

Fig. S62. $^{13}C\{^{1}H\}$ NMR ($CDCl_3$, 175 MHz) spectrum of 9'-tosyl-9'*H*-9,3':6',9''-tercarbazole.

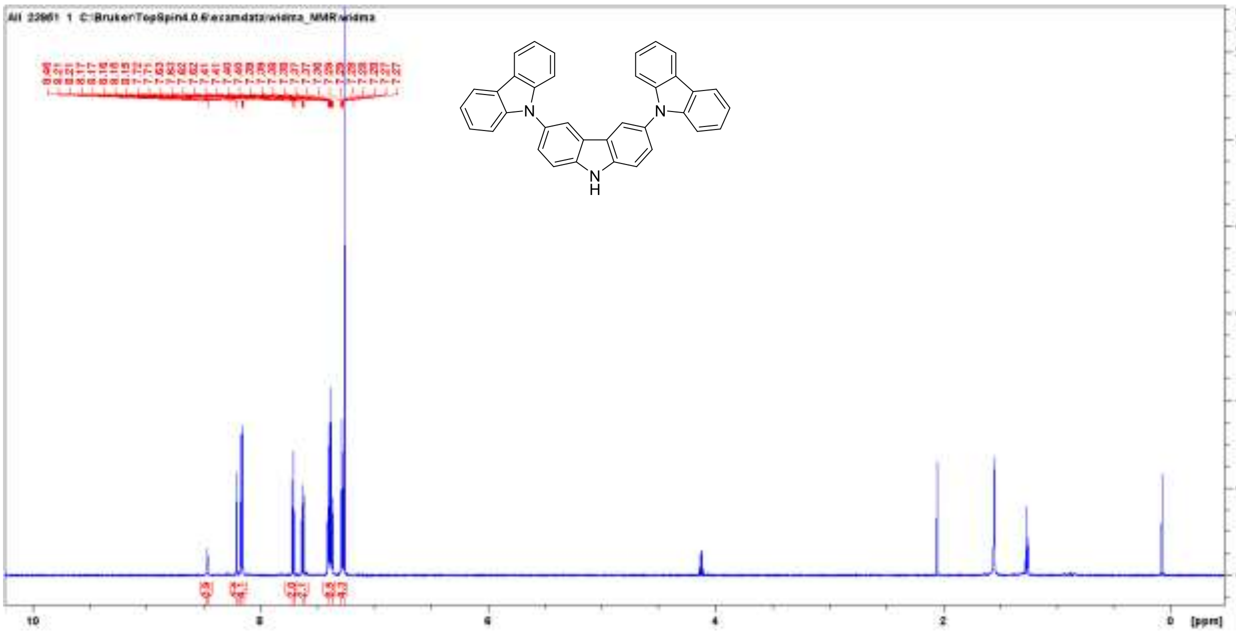

Fig. S63. ^{1}H NMR ($CDCl_3$, 700 MHz) spectrum of **1c**.

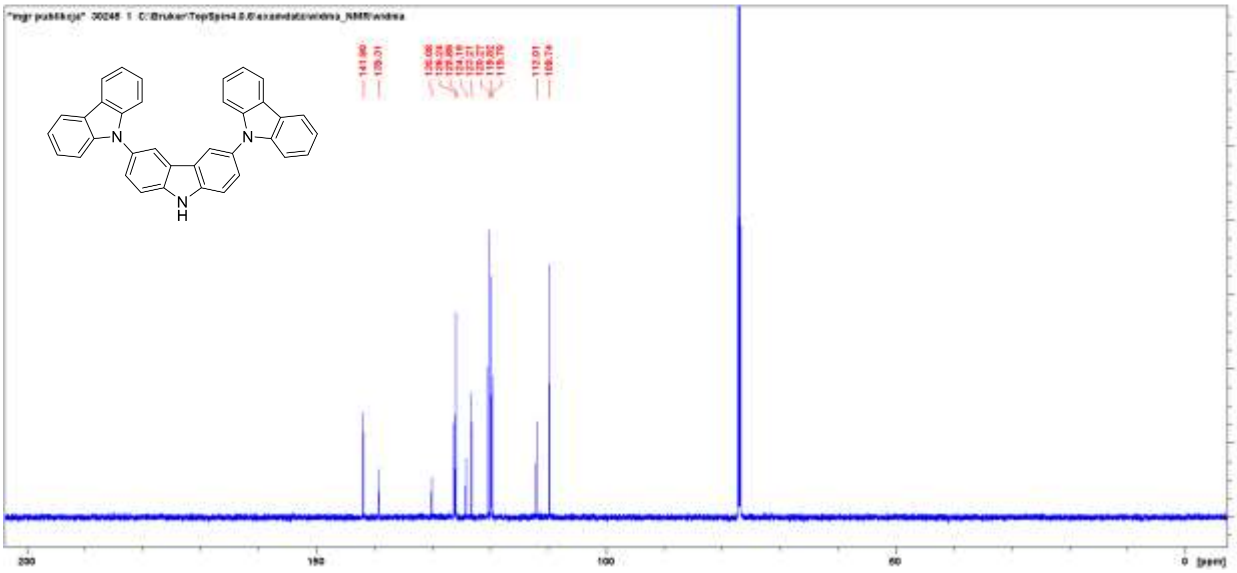

Fig. S64. $^{13}C\{^{1}H\}$ NMR ($CDCl_3$, 175 MHz) spectrum of **2c**.

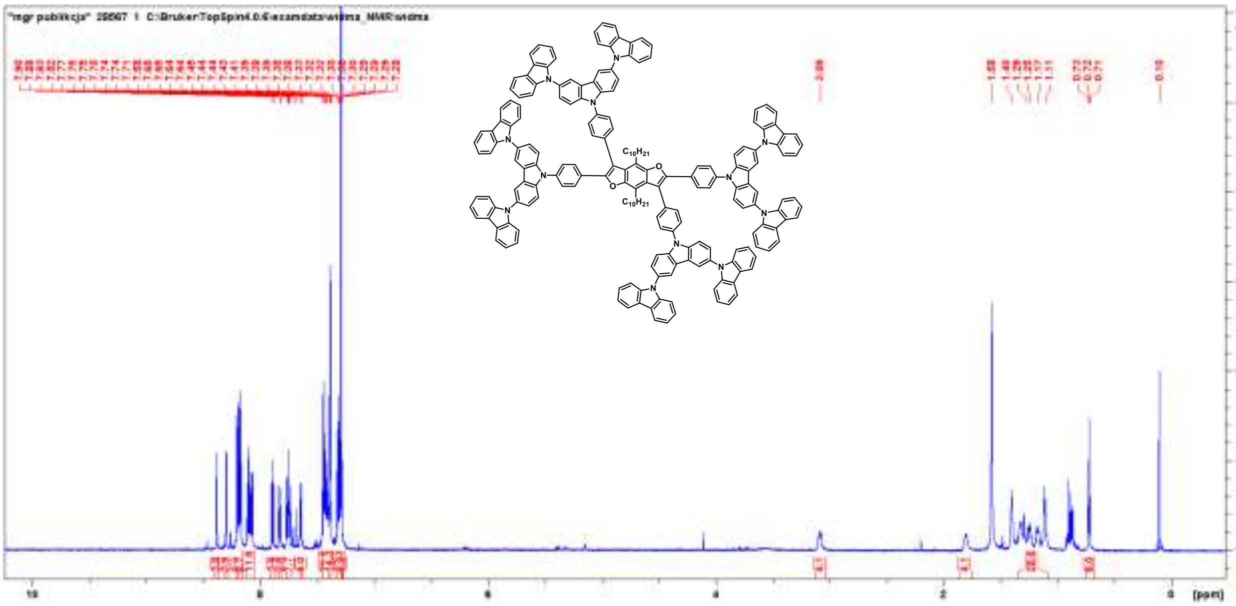

Fig. S65. ^{1}H NMR ($CDCl_3$, 700 MHz) spectrum of **2h**.

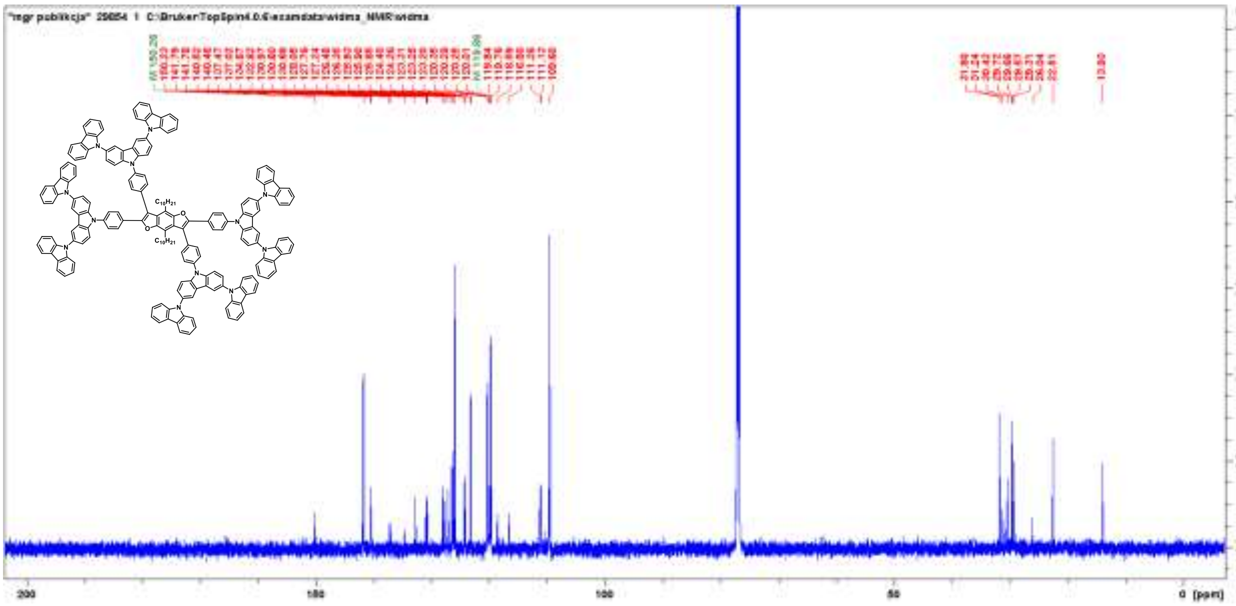

Fig. S66. $^{13}C\{^{1}H\}$ NMR ($CDCl_3$, 175 MHz) spectrum of **2h**.

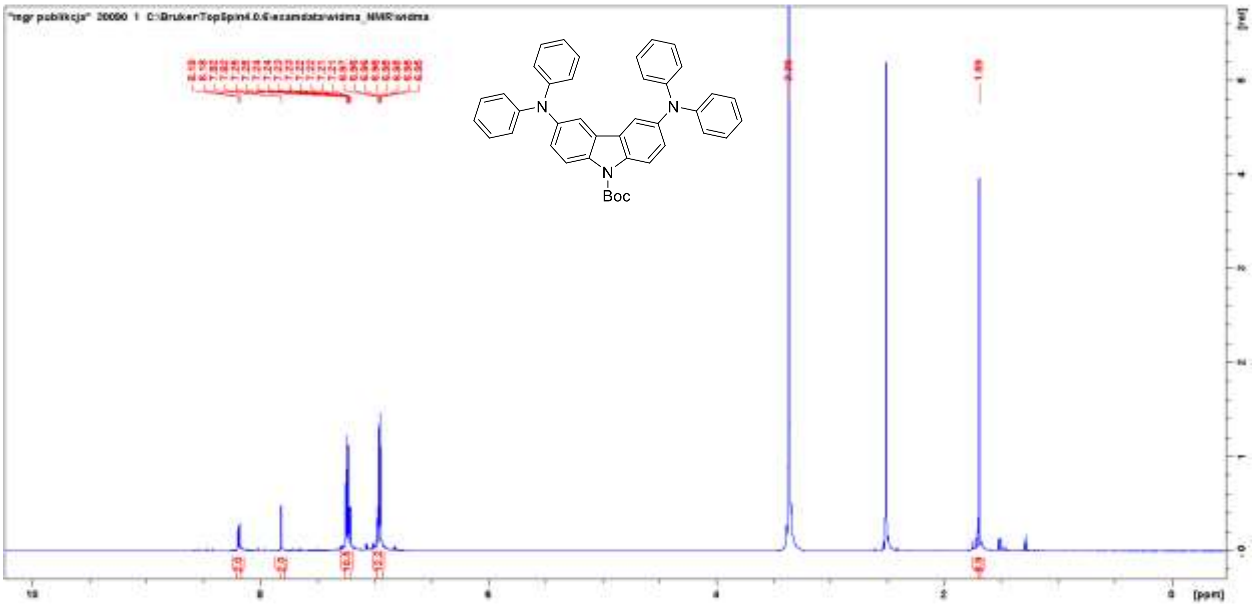

Fig. S67. ^{1}H NMR (DMSO-d_6, 700 MHz) spectrum of *tert*-butyl 3,6-bis(diphenylamino)-9*H*-carbazole-9-carboxylate.

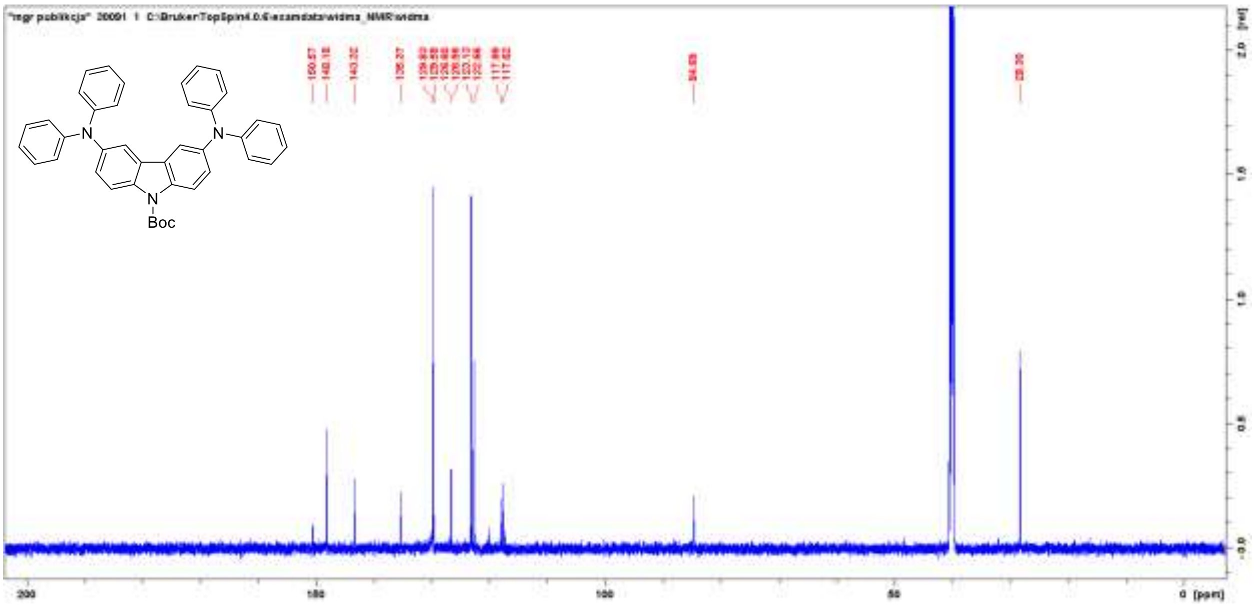

Fig. S68. $^{13}C\{^{1}H\}$ NMR (DMSO-d_6, 175 MHz) spectrum of *tert*-butyl 3,6-bis(diphenylamino)-9*H*-carbazole-9-carboxylate.

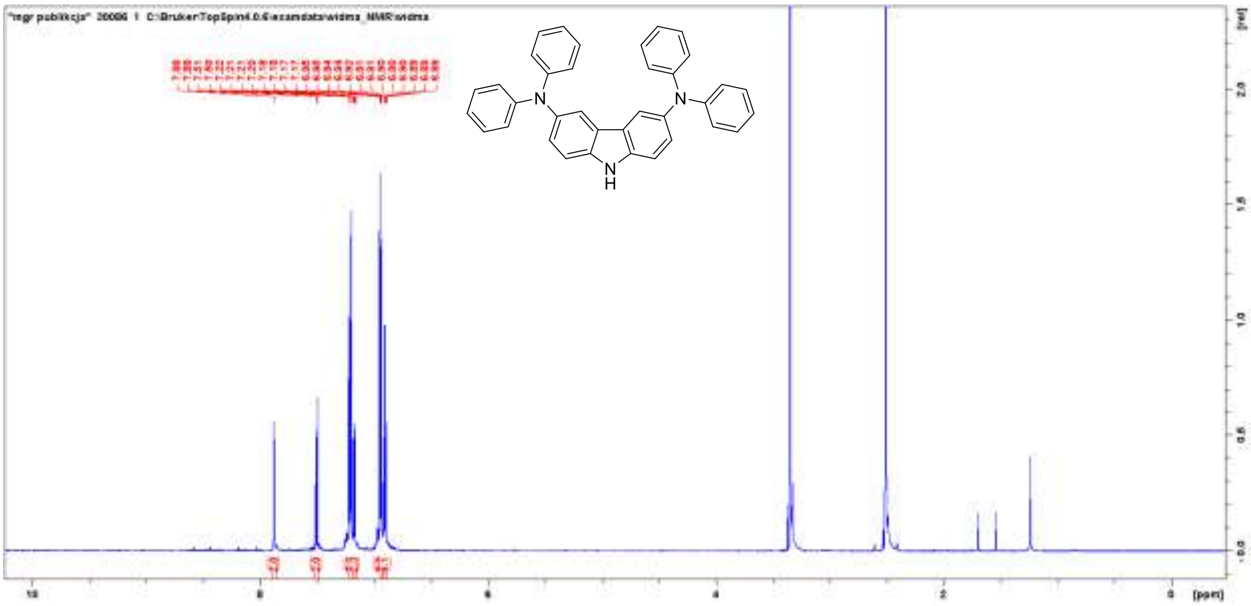

Fig. S69. ^{1}H NMR (DMSO-d$_6$, 700 MHz) spectrum of **1d**.

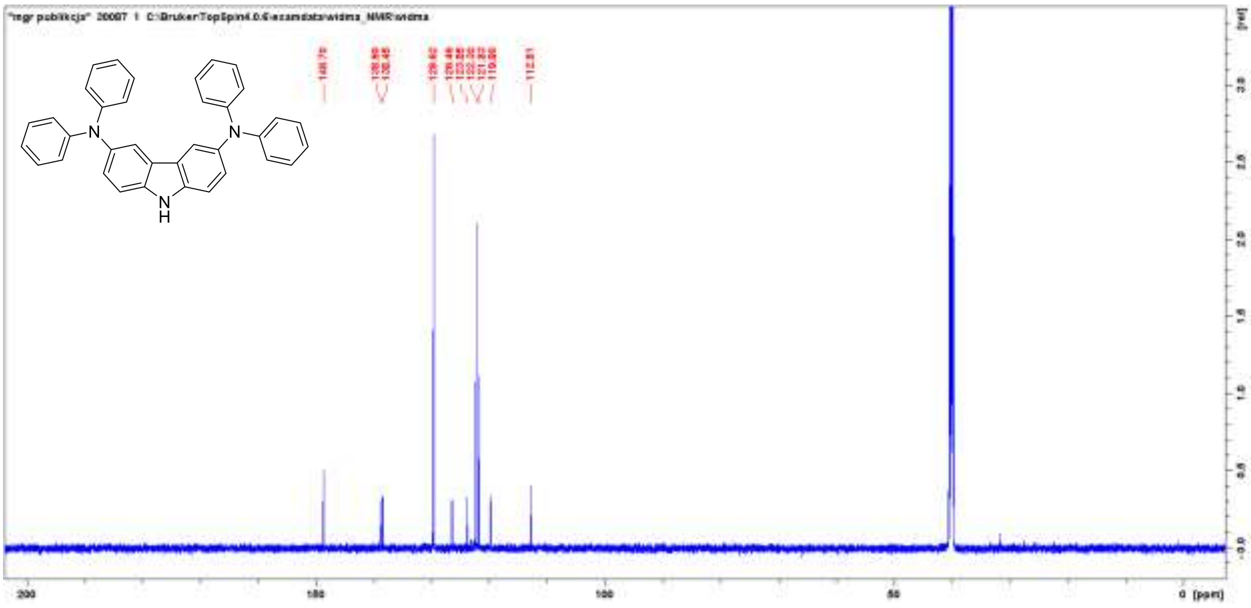

Fig. S70. ^{13}C{^{1}H} NMR (DMSO-d$_6$, 175 MHz) spectrum of **1d**.

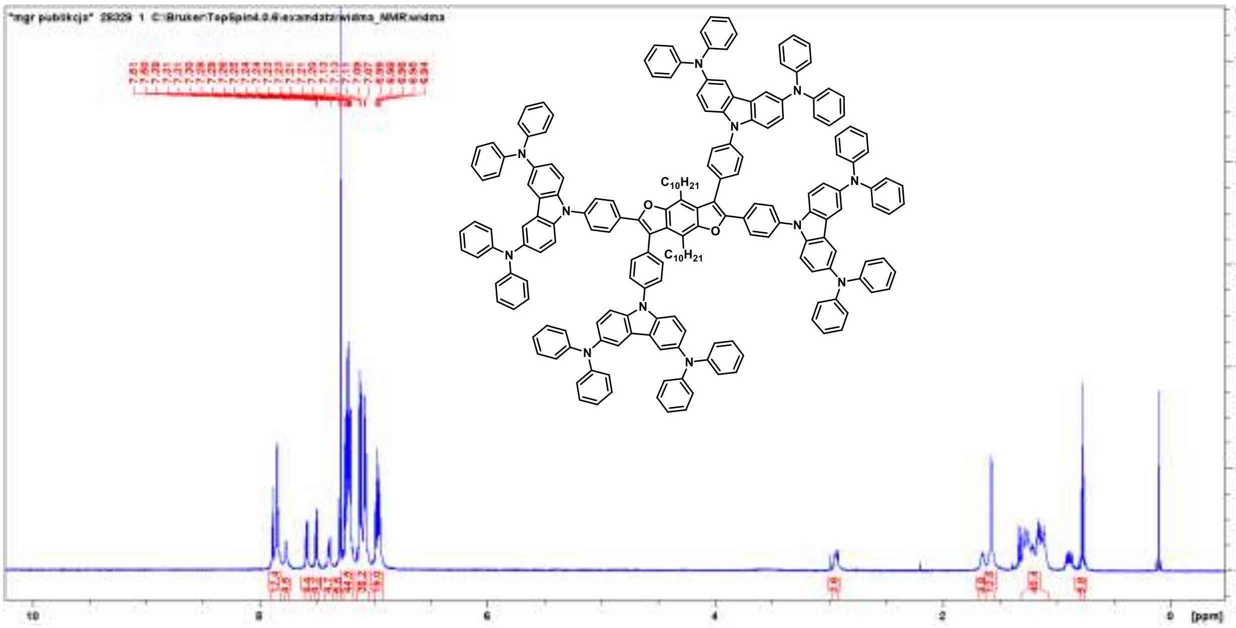

Fig. S71. ^{1}H NMR ($CDCl_3$, 700 MHz) spectrum of **2i**.

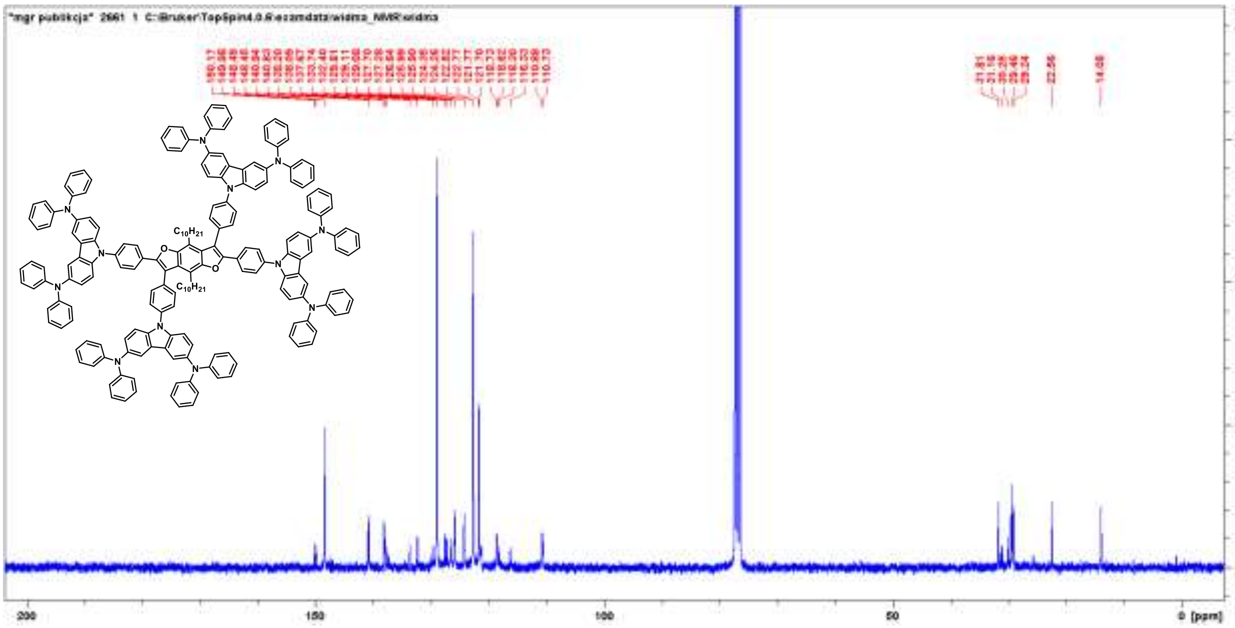

Fig. S72. $^{13}C\{^{1}H\}$ NMR ($CDCl_3$, 175 MHz) spectrum of **2i**.

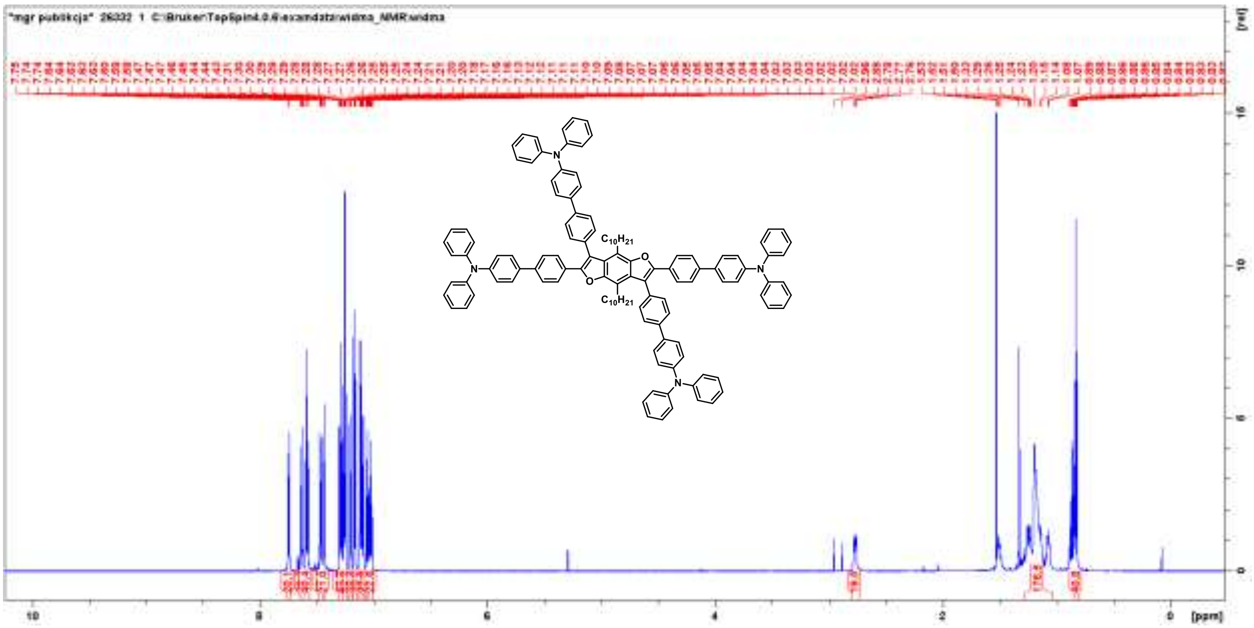

Fig. S73. ^{1}H NMR ($CDCl_3$, 700 MHz) spectrum of **2j**.

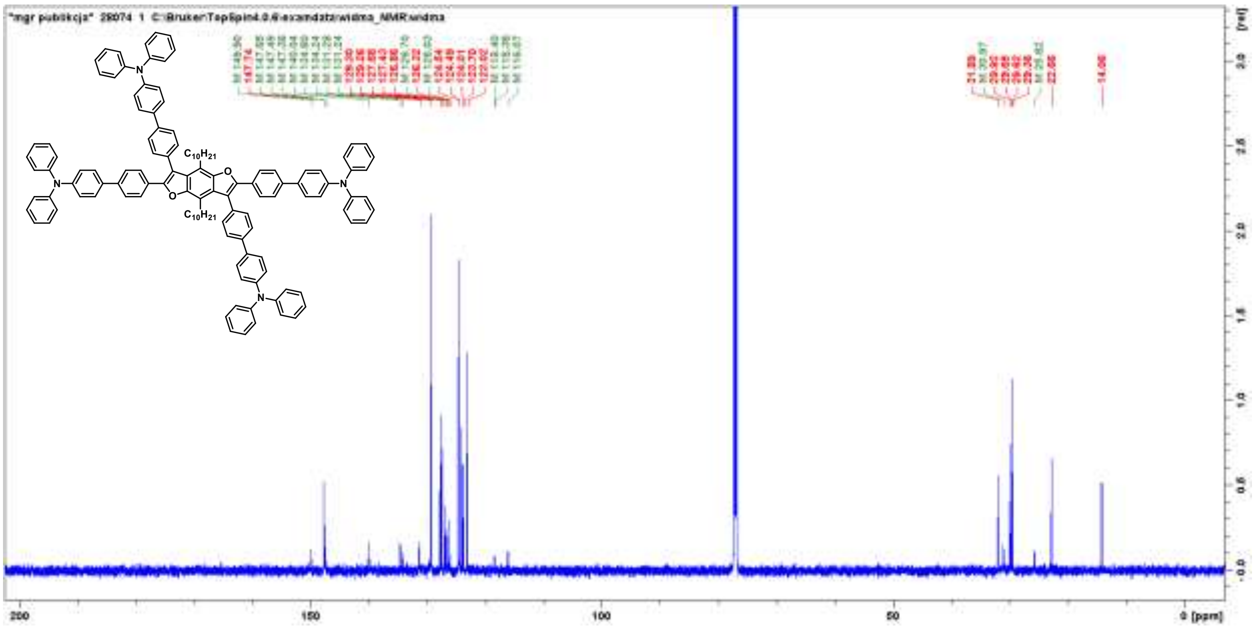

Fig. S74. $^{13}C\{^{1}H\}$ NMR ($CDCl_3$, 175 MHz) spectrum of **2j**.

9. Geometries of the optimized structures: PBE0/6-31G* level of theory with GD3 empirical dispersion, gas phase

1. **2a**

C	-7.2865940	-6.5319420	-0.2339930
N	-7.1387560	-5.1681300	-0.0091800
C	-8.2729450	-4.6995640	0.6470080
C	-9.1679850	-5.7761680	0.8394930
C	-8.5385140	-6.9465210	0.2750970
C	-10.3787200	-5.5539530	1.4983480
C	-10.6701480	-4.2791030	1.9642550
C	-9.7587800	-3.2277780	1.7859180
C	-8.5509320	-3.4196410	1.1258370
C	-6.4246840	-7.4184590	-0.8770490
C	-6.8303320	-8.7427820	-0.9856980
C	-8.0627920	-9.1755310	-0.4750270
C	-8.9217790	-8.2830160	0.1515510
C	-6.0293580	-4.3752210	-0.3597180
C	-4.7420650	-4.7157730	0.0622430
C	-3.6745030	-3.8733890	-0.2165720
C	-3.8738110	-2.6687430	-0.9059770
C	-5.1596130	-2.3732350	-1.3849830
C	-6.2240550	-3.2161790	-1.1150860
C	-2.7833040	-1.7153660	-1.0500860
O	-1.5155580	-2.2215350	-1.0577110
C	-0.6591080	-1.1591030	-1.1151770
C	-1.3900990	0.0523410	-1.1408190
C	-2.7783640	-0.3448990	-1.1030450
C	0.7199710	-1.2894360	-1.1160960
C	1.3900720	-0.0524020	-1.1408670
C	0.6590800	1.1590390	-1.1151540
C	-0.7200000	1.2893740	-1.1160150
C	2.7783370	0.3448340	-1.1030920
C	2.7832790	1.7153010	-1.0500880
O	1.5155320	2.2214680	-1.0576690
C	3.9955700	-0.4853030	-1.0361570
C	4.7961730	-0.4426940	0.1120860
C	5.9993830	-1.1286040	0.1712900
C	6.4396700	-1.8637930	-0.9326940
C	5.6454570	-1.9272680	-2.0794830
C	4.4302930	-1.2513070	-2.1223340
N	7.6836260	-2.5212670	-0.8819460
C	8.8811010	-1.9420940	-0.4689220
C	9.8909310	-2.9303710	-0.4675660
C	9.2721660	-4.1590860	-0.9038710
C	7.9122430	-3.8666150	-1.1551240
C	11.1937480	-2.5848170	-0.1025900
C	11.4717440	-1.2675700	0.2363170
C	10.4638510	-0.2926740	0.2010910

C	9.1580370	-0.6129130	-0.1491820
C	7.0193100	-4.8509330	-1.5765420
C	7.5127390	-6.1388430	-1.7485730
C	8.8603320	-6.4444420	-1.5113070
C	9.7436910	-5.4596370	-1.0893770
C	3.8737770	2.6686860	-0.9059540
C	3.6744400	3.8733180	-0.2165320
C	4.7419790	4.7157290	0.0622890
C	6.0292780	4.3752200	-0.3596850
C	6.2240060	3.2161890	-1.1150590
C	5.1595870	2.3732160	-1.3849610
N	7.1386530	5.1681680	-0.0091560
C	7.2864380	6.5319850	-0.2339760
C	8.5383420	6.9466140	0.2751130
C	9.1678580	5.7762870	0.8395150
C	8.2728590	4.6996490	0.6470340
C	8.9215560	8.2831230	0.1515600
C	8.0625350	9.1756030	-0.4750210
C	6.8300900	8.7428040	-0.9856890
C	6.4244920	7.4184660	-0.8770340
C	8.5508930	3.4197370	1.1258660
C	9.7587460	3.2279250	1.7859520
C	10.6700720	4.2792860	1.9642920
C	10.3785970	5.5541220	1.4983770
C	-3.9955930	0.4852400	-1.0360880
C	-4.7962130	0.4425950	0.1121410
C	-5.9994050	1.1285390	0.1713570
C	-6.4396500	1.8637930	-0.9325970
C	-5.6454260	1.9272920	-2.0793780
C	-4.4302850	1.2512980	-2.1222420
N	-7.6835730	2.5213300	-0.8818260
C	-7.9121140	3.8667000	-1.1549510
C	-9.2720530	4.1592040	-0.9038290
C	-9.8909100	2.9304820	-0.4676720
C	-8.8811120	1.9421680	-0.4689830
C	-9.7434990	5.4597930	-1.0892840
C	-8.8600490	6.4445880	-1.5110490
C	-7.5124440	6.1389480	-1.7482020
C	-7.0190900	4.8510040	-1.5762080
C	-9.1581160	0.6129790	-0.1493390
C	-10.4639780	0.2927580	0.2007720
C	-11.4718460	1.2676810	0.2359380
C	-11.1937760	2.5849410	-0.1028620
H	5.9996320	-2.4799740	-2.9443960
H	3.8315190	-1.2779500	-3.0288220
H	5.3255610	1.4782430	-1.9738690
H	7.2179440	2.9854140	-1.4869590
H	7.8417870	2.6065370	1.0038880
H	9.9925440	2.2428220	2.1811510
H	11.6068650	4.0940510	2.4818250

H	11.0774280	6.3723790	1.6513220
H	9.8810190	8.6165720	0.5383110
H	8.3473690	10.2188570	-0.5762560
H	6.1771600	9.4562080	-1.4810740
H	5.4754460	7.0841150	-1.2845740
H	4.5933690	5.6200270	0.6449560
H	2.6801840	4.1206240	0.1424840
C	1.4090420	-2.6185090	-1.0077520
H	-3.8314950	1.2779620	-3.0287180
H	-5.9995720	2.4800680	-2.9442590
H	-8.3869410	-0.1504930	-0.1890620
H	-10.7051770	-0.7395570	0.4391470
H	-12.4814320	0.9842960	0.5187860
H	-11.9781040	3.3373280	-0.0946390
H	-10.7873680	5.6956560	-0.8997000
H	-9.2131720	7.4611760	-1.6567650
H	-6.8346140	6.9242120	-2.0710190
H	-5.9723480	4.6253970	-1.7530260
H	-6.6065040	1.1033970	1.0712350
H	-4.4648250	-0.1439100	0.9642810
H	-5.3255620	-1.4782510	-1.9738820
H	-7.2179860	-2.9853740	-1.4869830
H	-5.4756250	-7.0841460	-1.2845940
H	-6.1774300	-9.4562130	-1.4810820
H	-8.3476670	-10.2187750	-0.5762560
H	-9.8812530	-8.6164260	0.5383050
H	-11.0775820	-6.3721830	1.6512950
H	-11.6069370	-4.0938300	2.4817820
H	-9.9925420	-2.2426630	2.1811100
H	-7.8417930	-2.6064690	1.0038660
H	-4.5934780	-5.6200800	0.6449020
H	-2.6802520	-4.1207240	0.1424390
C	-1.4090650	2.6184410	-1.0075570
H	4.4647580	0.1437660	0.9642480
H	6.6064630	-1.1034870	1.0711790
H	5.9725750	-4.6253690	-1.7534500
H	6.8349740	-6.9241120	-2.0715130
H	9.2135160	-7.4610030	-1.6570650
H	10.7875520	-5.6954640	-0.8997020
H	8.3868500	0.1505450	-0.1889550
H	10.7049950	0.7396360	0.4395410
H	12.4812900	-0.9841700	0.5192950
H	11.9780960	-3.3371830	-0.0943260
H	0.7028280	-3.4197040	-1.2506300
H	2.2108230	-2.6702920	-1.7540800
C	2.0191330	-2.8498670	0.3827100
H	-0.7028630	3.4196490	-1.2504210
H	-2.2108840	2.6702760	-1.7538420
C	-2.0190820	2.8497270	0.3829490
C	-3.0625490	3.9623640	0.4012080

H	-2.4987270	1.9202530	0.7124460
H	-1.2184870	3.0505580	1.1071210
C	-3.9320210	3.9435110	1.6551280
H	-2.5846720	4.9444080	0.2742580
H	-3.7246200	3.8280400	-0.4674960
C	-5.0812980	4.9564990	1.6191800
H	-4.3551630	2.9332780	1.7455990
H	-3.3143230	4.0926870	2.5514890
C	-6.3306350	4.4608460	2.3403140
H	-4.7593120	5.9211580	2.0351120
H	-5.3552570	5.1579020	0.5747560
C	-7.5168580	5.4147080	2.2580950
H	-6.6237500	3.5009660	1.8887160
H	-6.0979250	4.2406530	3.3928100
C	-8.8087400	4.7887040	2.7720290
H	-7.2959860	6.3343750	2.8198840
H	-7.6620090	5.7197940	1.2120490
C	-10.0159090	5.7148460	2.6784510
H	-9.0157140	3.8774440	2.1915290
H	-8.6763880	4.4630930	3.8150410
C	-11.3125580	5.0342990	3.0998790
H	-9.8431940	6.6066850	3.2974370
H	-10.1057910	6.0737870	1.6441500
H	-12.1720530	5.7091610	3.0160290
H	-11.5105070	4.1550190	2.4744680
H	-11.2591060	4.6910340	4.1402610
C	3.0626210	-3.9624900	0.4008740
H	2.4987850	-1.9204010	0.7122240
H	1.2185680	-3.0507280	1.1069090
C	3.9322830	-3.9435040	1.6546640
H	2.5847600	-4.9445580	0.2740630
H	3.7245610	-3.8282040	-0.4679360
C	5.0816190	-4.9563900	1.6186080
H	4.3553760	-2.9332390	1.7450000
H	3.3147400	-4.0926670	2.5511360
C	6.3307370	-4.4610140	2.3403090
H	4.7595540	-5.9212550	2.0340080
H	5.3558870	-5.1573010	0.5741690
C	7.5170120	-5.4148080	2.2578820
H	6.6239120	-3.5008880	1.8892710
H	6.0977780	-4.2413760	3.3928620
C	8.8088290	-4.7889600	2.7721650
H	7.2960980	-6.3346740	2.8193260
H	7.6622900	-5.7195370	1.2117480
C	10.0159850	-5.7151170	2.6785320
H	9.0159070	-3.8775650	2.1919130
H	8.6763420	-4.4636020	3.8152370
C	11.3125970	-5.0347100	3.1002940
H	9.8431540	-6.6070960	3.2972850
H	10.1059970	-6.0738200	1.6441610

H	12.1720820	-5.7095820	3.0164140
H	11.5106610	-4.1552980	2.4751040
H	11.2590140	-4.6916730	4.1407450

2. **2b**

C	-9.5451710	-2.0733830	1.2093090
C	-8.7951330	-1.5124720	0.1713350
C	-9.0377380	-0.1921420	-0.2190340
C	-10.0150330	0.5592550	0.4229790
C	-10.7691390	-0.0033790	1.4509950
C	-10.5323520	-1.3213750	1.8370640
N	-7.7758280	-2.2597310	-0.4755490
C	-8.0510630	-3.5414280	-0.9820770
C	-7.0365300	-4.5009420	-1.1095140
C	-7.3274340	-5.7758230	-1.5782730
C	-8.6303110	-6.1326370	-1.9173310
C	-9.6392540	-5.1794610	-1.7992370
C	-9.3579090	-3.8944710	-1.3504470
C	-6.5002190	-1.6667130	-0.5740700
C	-5.7275100	-1.7628760	-1.7376750
C	-4.4983950	-1.1175830	-1.8200940
C	-4.0121670	-0.3460620	-0.7595400
C	-4.7864400	-0.2689690	0.4052010
C	-6.0032220	-0.9232710	0.5047150
C	-2.7699740	0.4425550	-0.8574650
C	-1.3962590	-0.0060010	-0.8902740
C	-0.6203490	1.1769840	-0.8748400
O	-1.4364560	2.2699410	-0.8379040
C	-2.7250800	1.8141940	-0.8258080
C	-0.7630390	-1.2625720	-0.8788100
C	0.6201380	-1.1763310	-0.8747620
C	1.3960560	0.0066570	-0.8902590
C	0.7628330	1.2632250	-0.8788730
O	1.4362370	-2.2692880	-0.8377240
C	2.7248640	-1.8135540	-0.8256450
C	2.7697750	-0.4419140	-0.8574270
C	3.7609750	-2.8297380	-0.7360330
C	5.0778830	-2.5810750	-1.1557480
C	6.0677200	-3.5335760	-0.9931110
C	5.7802620	-4.7708760	-0.4013060
C	4.4600960	-5.0430050	-0.0202250
C	3.4688210	-4.0896950	-0.1921000
C	4.0119790	0.3466820	-0.7595280
C	4.7862970	0.2694700	0.4051810
C	6.0032010	0.9235470	0.5046390
C	6.5002770	1.6669100	-0.5741650
C	5.7274670	1.7633080	-1.7376880
C	4.4982530	1.1181960	-1.8200640
N	7.7760710	2.2595240	-0.4757670
C	8.7951420	1.5118650	0.1710920

C	9.0376920	0.1916980	-0.2198440
C	10.0146890	-0.5601300	0.4221110
C	10.7685250	0.0019430	1.4506380
C	10.5318070	1.3197870	1.8372580
C	9.5449160	2.0722210	1.2095390
N	6.8070250	-5.7113340	-0.1912850
C	6.5627510	-7.0873190	-0.3913140
C	7.0864130	-8.0389940	0.4919720
C	6.8481090	-9.3920550	0.2823820
C	6.0721870	-9.8174390	-0.7934980
C	5.5426300	-8.8704390	-1.6673230
C	5.7910010	-7.5163970	-1.4779200
C	-3.7612070	2.8303540	-0.7361680
C	-3.4690850	4.0902970	-0.1921800
C	-4.4603930	5.0435550	-0.0202310
C	-5.7805630	4.7713930	-0.4012910
C	-6.0679810	3.5341190	-0.9931680
C	-5.0781080	2.5816660	-1.1558740
N	-6.8073580	5.7117810	-0.1911360
C	-8.0708000	5.2732840	0.2620280
C	-8.1658540	4.2575090	1.2215420
C	-9.4108730	3.8211360	1.6580480
C	-10.5783410	4.3965820	1.1594590
C	-10.4849790	5.4128180	0.2104770
C	-9.2447000	5.8454710	-0.2434700
C	8.0517740	3.5411320	-0.9822020
C	7.0375530	4.5009470	-1.1098560
C	7.3289470	5.7757710	-1.5784700
C	8.6320070	6.1322260	-1.9171900
C	9.6406390	5.1787440	-1.7988970
C	9.3588190	3.8938180	-1.3502320
C	8.0704450	-5.2730050	0.2620570
C	8.1655290	-4.2571230	1.2214590
C	9.4105540	-3.8209420	1.6581410
C	10.5780070	-4.3966580	1.1598320
C	10.4846170	-5.4129800	0.2109440
C	9.2443390	-5.8454580	-0.2431690
C	-6.5632460	7.0877980	-0.3910720
C	-7.0872730	8.0393550	0.4921270
C	-6.8491600	9.3924640	0.2826380
C	-6.0730730	9.8180250	-0.7930530
C	-5.5431570	8.8711470	-1.6667900
C	-5.7913300	7.5170540	-1.4774930
H	3.9263650	1.1746190	-2.7427140
H	6.1040250	2.3295180	-2.5842790
H	10.1539230	3.1594290	-1.2726720
H	10.6627260	5.4303330	-2.0699970
H	8.8570000	7.1336980	-2.2715600
H	6.5243680	6.5021830	-1.6606120
H	6.0216170	4.2486750	-0.8249820

H	9.3475060	3.0950880	1.5171380
H	11.1092770	1.7646980	2.6433000
H	11.5364900	-0.5846690	1.9477110
H	10.1891050	-1.5874830	0.1153080
H	8.4472790	-0.2390520	-1.0236630
H	6.5901120	0.8519220	1.4152030
H	4.4210060	-0.3206910	1.2410260
H	2.4542940	-4.3053610	0.1284900
H	4.2213620	-6.0023640	0.4287820
H	9.1736100	-6.6297250	-0.9909420
H	11.3860960	-5.8672080	-0.1917960
H	11.5497480	-4.0586550	1.5090530
H	9.4655860	-3.0265440	2.3976780
H	7.2579070	-3.8113870	1.6169020
H	5.3884690	-6.7802890	-2.1674650
H	4.9409460	-9.1869020	-2.5152560
H	5.8820940	-10.8754370	-0.9492480
H	7.2619340	-10.1183320	0.9770070
H	7.6800920	-7.7089510	1.3391950
H	7.0811450	-3.3254290	-1.3231310
H	5.3254450	-1.6370220	-1.6280250
C	-1.4751580	-2.5823410	-0.8214770
H	-5.3256480	1.6376200	-1.6281770
H	-7.0813990	3.3259460	-1.3231870
H	-5.3885180	6.7810510	-2.1669860
H	-4.9413420	9.1877430	-2.5145810
H	-5.8831300	10.8760630	-0.9487230
H	-7.2632710	10.1186400	0.9771980
H	-7.6810800	7.7091820	1.3392100
H	-9.1739700	6.6296780	-0.9913060
H	-11.3864760	5.8668310	-0.1924650
H	-11.5500810	4.0584370	1.5085460
H	-9.4658950	3.0268340	2.3976910
H	-7.2582060	3.8120070	1.6171910
H	-4.2216950	6.0028920	0.4288430
H	-2.4545610	4.3059770	0.1284120
H	-4.4211950	0.3212320	1.2410370
H	-6.5900930	-0.8517880	1.4153170
H	-10.1532390	-3.1603080	-1.2730580
H	-10.6612020	-5.4313370	-2.0705890
H	-8.8549180	-7.1341570	-2.2718120
H	-6.5226250	-6.5019960	-1.6602800
H	-6.0207390	-4.2483860	-0.8243760
H	-9.3477150	-3.0963500	1.5165450
H	-11.1099970	-1.7667460	2.6427270
H	-11.5373430	0.5828880	1.9480990
H	-10.1894590	1.5867230	0.1165480
H	-8.4471470	0.2391100	-1.0224510
H	-6.1040370	-2.3291130	-2.5842610
H	-3.9265370	-1.1738830	-2.7427690

C	1.4749040	2.5830230	-0.8215500
C	2.0998780	2.8761110	0.5467460
H	2.2725710	2.5982140	-1.5744060
H	0.7748410	3.3825320	-1.0892860
C	2.9442850	4.1482400	0.5407130
H	1.3108820	2.9370850	1.3077310
H	2.7348540	2.0284570	0.8289370
C	3.9818520	4.1881490	1.6588550
H	3.4822770	4.2083760	-0.4180740
H	2.2988310	5.0374350	0.5801570
C	4.8522630	5.4466560	1.6285670
H	3.4934840	4.0823460	2.6375050
H	4.6252910	3.3035080	1.5454240
C	6.2574050	5.2243530	2.1791550
H	4.9510170	5.7858890	0.5864460
H	4.3565940	6.2710900	2.1597420
C	7.1797310	6.4300190	2.0061320
H	6.2112700	4.9277520	3.2367050
H	6.6961600	4.3680550	1.6464100
C	8.6558950	6.0513780	2.0308660
H	6.9678740	6.9054670	1.0380980
H	6.9657550	7.1878610	2.7733790
C	9.6015410	7.2272030	1.8176390
H	8.8993510	5.5479140	2.9793100
H	8.8319590	5.3173500	1.2311820
C	11.0597510	6.7932170	1.7365910
H	9.3213890	7.7396870	0.8867270
H	9.4712000	7.9604340	2.6260010
H	11.7305050	7.6452940	1.5797230
H	11.3737950	6.2865040	2.6574950
H	11.2064490	6.0916500	0.9061880
C	-2.1000450	-2.8755590	0.5468220
H	-2.2728830	-2.5974530	-1.5742780
H	-0.7751450	-3.3818580	-1.0893320
C	-2.9441660	-4.1478840	0.5407480
H	-1.3110280	-2.9363960	1.3077960
H	-2.7351960	-2.0280480	0.8290410
C	-3.9818610	-4.1879990	1.6587620
H	-3.4820160	-4.2082020	-0.4181090
H	-2.2985150	-5.0369310	0.5803060
C	-4.8518070	-5.4468300	1.6285410
H	-3.4936590	-4.0818650	2.6374580
H	-4.6255940	-3.3035980	1.5451110
C	-6.2570530	-5.2250070	2.1790470
H	-4.9504060	-5.7861670	0.5864350
H	-4.3558220	-6.2710330	2.1597780
C	-7.1790550	-6.4308740	2.0057260
H	-6.2111310	-4.9285740	3.2366530
H	-6.6959860	-4.3687370	1.6464000
C	-8.6553100	-6.0525660	2.0303700

H	-6.9670200	-6.9060760	1.0376080
H	-6.9649560	-7.1888170	2.7728350
C	-9.6006860	-7.2285890	1.8170810
H	-8.8989590	-5.5490970	2.9787610
H	-8.8314510	-5.3186190	1.2306250
C	-11.0590120	-6.7949510	1.7362840
H	-9.3205290	-7.7408320	0.8860360
H	-9.4700550	-7.9619400	2.6252880
H	-11.7295640	-7.6471490	1.5792150
H	-11.3731180	-6.2885790	2.6573530
H	-11.2059560	-6.0931840	0.9060880

3. **2c**

C	-8.6947730	2.3715330	0.5050880
N	-7.4595050	3.0185210	0.6689580
C	-7.4214740	4.4199090	0.7208630
C	-8.6120570	5.1385860	0.5295290
O	-9.8084010	4.5169080	0.2795940
C	-9.8486890	3.1456240	0.3139500
C	-8.6243170	6.5226730	0.5535520
C	-7.4427100	7.2344520	0.7577860
C	-6.2553550	6.5385280	0.9361180
C	-6.2451580	5.1440200	0.9190190
C	-8.8342280	0.9828020	0.5409080
C	-10.0805260	0.3829730	0.3630060
C	-11.2101490	1.1635750	0.1587340
C	-11.0878250	2.5524400	0.1425550
C	-6.2681940	2.2538610	0.7361130
C	-5.8381390	1.5435910	-0.3862780
C	-4.6956160	0.7586460	-0.3162440
C	-3.9510390	0.6837410	0.8659990
C	-4.3757990	1.4186060	1.9780650
C	-5.5330450	2.1870710	1.9203650
C	-2.7848910	-0.2162100	0.9366880
C	-1.3809730	0.1129390	0.9650340
C	-0.7096890	-1.1323160	0.9407500
O	-1.6170300	-2.1529970	0.8912020
C	-2.8583390	-1.5825160	0.8951670
C	-0.6625010	1.3210640	0.9412900
C	0.7096920	1.1322160	0.9407620
C	1.3809810	-0.1130340	0.9649650
C	0.6624990	-1.3211630	0.9412050
O	1.6170250	2.1529080	0.8912120
C	2.8583260	1.5824340	0.8951340
C	2.7849010	0.2161230	0.9365890
C	4.0067600	2.4731940	0.8066100
C	5.2227130	2.1246860	1.4141710
C	6.3431440	2.9236520	1.2553410
C	6.2786510	4.0768870	0.4705900
C	5.0634110	4.4526100	-0.1043390

C	3.9327830	3.6657550	0.0744090
C	3.9510820	-0.6837830	0.8659210
C	4.6958120	-0.7585170	-0.3162340
C	5.8383530	-1.5434520	-0.3862390
C	6.2682460	-2.2538930	0.7360960
C	5.5329630	-2.1872490	1.9202770
C	4.3757130	-1.4187970	1.9779430
N	7.4595620	-3.0185660	0.6690040
C	8.6948440	-2.3715740	0.5053460
C	9.8487410	-3.1456430	0.3139920
O	9.8083450	-4.5169170	0.2789660
C	8.6120690	-5.1386240	0.5291760
C	7.4214960	-4.4199630	0.7206470
C	11.0879160	-2.5524630	0.1429150
C	11.2102930	-1.1636030	0.1595750
C	10.0806810	-0.3830290	0.3640070
C	8.8343450	-0.9828630	0.5416330
C	6.2451850	-5.1440890	0.9187530
C	6.2553900	-6.5386000	0.9357240
C	7.4427480	-7.2345050	0.7573580
C	8.6243490	-6.5227070	0.5531490
N	7.4433920	4.8571650	0.2568730
C	7.6039490	6.0819590	0.9239290
C	8.7875970	6.8108210	0.7328110
O	9.7930610	6.3614790	-0.0855060
C	9.7017510	5.0843380	-0.5768870
C	8.5362090	4.3139170	-0.4369130
C	8.9886220	8.0261080	1.3633730
C	8.0097970	8.5499610	2.2068900
C	6.8405560	7.8329020	2.4182310
C	6.6416510	6.6051450	1.7877650
C	8.5133300	3.0486810	-1.0270700
C	9.6256640	2.5559120	-1.7099710
C	10.7791210	3.3205230	-1.8169940
C	10.8085540	4.5926710	-1.2471390
C	-4.0067680	-2.4732670	0.8066000
C	-5.2228050	-2.1246630	1.4139400
C	-6.3432250	-2.9236350	1.2550500
C	-6.2786300	-4.0769780	0.4704690
C	-5.0633110	-4.4528030	-0.1042210
C	-3.9327020	-3.6659340	0.0745730
N	-7.4433320	-4.8573170	0.2567190
C	-8.5360520	-4.3142280	-0.4373430
C	-9.7014720	-5.0848040	-0.5774960
O	-9.7926560	-6.3619600	-0.0861160
C	-8.7876370	-6.8107990	0.7330300
C	-7.6041190	-6.0817780	0.9243330
C	-10.8082030	-4.5933250	-1.2480010
C	-10.7787900	-3.3212310	-1.8179820
C	-9.6254440	-2.5564770	-1.7107940

C	-8.5131990	-3.0490370	-1.0275930
C	-6.6421860	-6.6044650	1.7888770
C	-6.8413230	-7.8319100	2.4198770
C	-8.0104450	-8.5491220	2.2083830
C	-8.9889140	-8.0257530	1.3641500
H	3.8107180	-1.3563270	2.9043270
H	5.8806610	-2.7349700	2.7914130
H	5.3076310	-4.6146730	1.0447490
H	5.3204920	-7.0703960	1.0861950
H	7.4599630	-8.3197570	0.7693100
H	9.5746590	-7.0243330	0.3983020
H	11.9454050	-3.2014020	-0.0065560
H	12.1835150	-0.7043510	0.0168670
H	10.1532110	0.7002750	0.3817030
H	7.9586690	-0.3634670	0.7055560
H	6.4168030	-1.6052400	-1.3033910
H	4.3701700	-0.1908550	-1.1830990
H	2.9910870	3.9495820	-0.3856420
H	5.0203990	5.3578940	-0.7028650
H	7.6155390	2.4440460	-0.9583500
H	9.5775980	1.5641770	-2.1498770
H	11.6501790	2.9421520	-2.3429160
H	11.6839820	5.2308350	-1.3179170
H	9.9229230	8.5470980	1.1782450
H	8.1712470	9.5067890	2.6936740
H	6.0704240	8.2178700	3.0801740
H	5.7296700	6.0460500	1.9674780
H	7.2842680	2.6558470	1.7272950
H	5.2842640	1.2286610	2.0220450
C	-1.3008270	2.6725840	0.8097250
H	-2.9909390	-3.9498330	-0.3852950
H	-5.0202250	-5.3581840	-0.7025950
H	-5.7303090	-6.0452340	1.9686980
H	-6.0714750	-8.2165160	3.0823590
H	-8.1720730	-9.5057040	2.6955920
H	-9.9231000	-8.5468900	1.1788600
H	-11.6835390	-5.2316000	-1.3189080
H	-11.6497780	-2.9430150	-2.3441330
H	-9.5773910	-1.5647930	-2.1508180
H	-7.6154910	-2.4442940	-0.9587440
H	-7.2844120	-2.6557540	1.7268340
H	-5.2844410	-1.2285670	2.0216980
H	-3.8109190	1.3560180	2.9045120
H	-5.8808390	2.7346770	2.7915360
H	-7.9585430	0.3633890	0.7047090
H	-10.1530200	-0.7003390	0.3803700
H	-12.1833430	0.7043350	0.0157910
H	-11.9453330	3.2013960	-0.0067370
H	-9.5746330	7.0243110	0.3987840
H	-7.4599180	8.3197030	0.7697930

H	-5.3204550	7.0703040	1.0866520
H	-5.3076040	4.6145880	1.0449620
H	-6.4164740	1.6055250	-1.3034920
H	-4.3698590	0.1911030	-1.1831430
C	1.3008650	-2.6726550	0.8095280
C	1.6660050	-2.9973800	-0.6473910
H	2.2087360	-2.7100010	1.4206450
H	0.6249610	-3.4414190	1.2001520
C	2.7201130	-4.0919070	-0.7717610
H	0.7534790	-3.2759550	-1.1922460
H	2.0473880	-2.0905230	-1.1351130
C	2.9059260	-4.5724580	-2.2102640
H	3.6676520	-3.6970310	-0.3796320
H	2.4557470	-4.9489050	-0.1345990
C	4.1777780	-5.3925550	-2.4392860
H	2.0223780	-5.1576660	-2.4993650
H	2.9221720	-3.7011190	-2.8824240
C	5.4347740	-4.5327930	-2.5316330
H	4.2969550	-6.1286810	-1.6304620
H	4.0782990	-5.9722750	-3.3672720
C	6.7184810	-5.3272770	-2.7367090
H	5.3129460	-3.8157910	-3.3584090
H	5.5360390	-3.9302790	-1.6200380
C	7.9424420	-4.4353610	-2.9165830
H	6.8748260	-5.9931050	-1.8768070
H	6.6114810	-5.9779320	-3.6177440
C	9.2422880	-5.2110250	-3.0984590
H	7.7886760	-3.7744820	-3.7835230
H	8.0404810	-3.7710050	-2.0448810
C	10.4476320	-4.3045120	-3.3127910
H	9.4071250	-5.8418320	-2.2150000
H	9.1366520	-5.8970880	-3.9509740
H	11.3717380	-4.8825800	-3.4251300
H	10.3255430	-3.6922290	-4.2148110
H	10.5810130	-3.6221960	-2.4654430
C	-1.6659340	2.9974260	-0.6471830
H	-2.2087210	2.7098770	1.4208090
H	-0.6249230	3.4412990	1.2004410
C	-2.7201630	4.0918240	-0.7715700
H	-0.7534090	3.2761210	-1.1919780
H	-2.0472100	2.0905640	-1.1349820
C	-2.9059750	4.5722700	-2.2101160
H	-3.6676650	3.6968180	-0.3794840
H	-2.4559430	4.9488750	-0.1344240
C	-4.1777840	5.3923580	-2.4393080
H	-2.0224090	5.1574230	-2.4992690
H	-2.9222360	3.7008470	-2.8821790
C	-5.4349130	4.5327600	-2.5312490
H	-4.2968280	6.1287970	-1.6307430
H	-4.0782970	5.9717340	-3.3675100

C	-6.7184650	5.3274730	-2.7364720
H	-5.3133160	3.8154480	-3.3577900
H	-5.5362150	3.9306050	-1.6194300
C	-7.9425740	4.4358130	-2.9165680
H	-6.8748020	5.9932730	-1.8765510
H	-6.6112140	5.9781660	-3.6174510
C	-9.2422900	5.2117580	-3.0981830
H	-7.7889410	3.7751880	-3.7837240
H	-8.0406910	3.7711950	-2.0450680
C	-10.4478580	4.3055160	-3.3124220
H	-9.4068600	5.8425580	-2.2146680
H	-9.1366370	5.8978460	-3.9506780
H	-11.3718620	4.8837850	-3.4245470
H	-10.3260330	3.6933230	-4.2145390
H	-10.5812370	3.6231130	-2.4651470

4. **2d**

C	-7.8681010	-4.1466540	0.7405340
N	-7.6877500	-2.7956810	1.0969150
C	-8.7632480	-1.8773130	1.0945700
C	-10.0679340	-2.3167020	1.3770080
S	-10.3514360	-3.9763490	1.9135950
C	-9.0918730	-4.7929380	0.9830200
C	-11.1402290	-1.4290470	1.3413930
C	-10.9352250	-0.0774650	1.0848530
C	-9.6445530	0.3749630	0.8424220
C	-8.5732550	-0.5133260	0.8341010
C	-6.8358540	-4.8869640	0.1547680
C	-7.0206960	-6.2230710	-0.1890410
C	-8.2489010	-6.8410030	0.0111530
C	-9.2846710	-6.1143790	0.5907170
C	-6.3690620	-2.2515940	1.0354410
C	-5.6056330	-2.1929880	2.1986630
C	-4.3849550	-1.5249280	2.1951770
C	-3.9243510	-0.8896360	1.0366790
C	-4.6596780	-1.0288840	-0.1458590
C	-5.8669270	-1.7163950	-0.1521740
C	-2.7766770	0.0380250	1.0875190
C	-1.3628310	-0.2397070	1.0899450
C	-0.7360110	1.0278520	1.1255370
O	-1.6793050	2.0137040	1.1364080
C	-2.9048390	1.4039990	1.1172780
C	-0.6005930	-1.4161550	1.0009740
C	0.7647590	-1.1800000	1.0002940
C	1.3915080	0.0843490	1.0831490
C	0.6277950	1.2639890	1.1248540
O	1.7071950	-2.1618970	0.8939360
C	2.9301900	-1.5510040	0.9218110
C	2.8055120	-0.1904150	1.0345050
C	4.0919890	-2.4193600	0.7852440

C	3.9777120	-3.6437660	0.1118650
C	5.0918660	-4.4515740	-0.0809840
C	6.3357980	-4.0573720	0.4045050
C	6.4521950	-2.8595270	1.1139410
C	5.3446590	-2.0477180	1.3014550
C	3.9517850	0.7406950	1.0595790
C	4.4171840	1.2741360	2.2665800
C	5.6274880	1.9587230	2.3179010
C	6.3762390	2.1375380	1.1569590
C	5.8701920	1.6988490	-0.0681110
C	4.6740360	0.9929280	-0.1120140
N	7.6750370	2.7219490	1.2517380
C	8.7892200	1.8591900	1.1291600
C	10.0778600	2.3232820	1.4442620
S	10.2957450	3.9187440	2.1720420
C	8.9888860	4.7806220	1.3552880
C	7.7917280	4.1120260	1.0495390
C	11.1887690	1.4982310	1.2896480
C	11.0416480	0.1777980	0.8778390
C	9.7679830	-0.3045340	0.6045160
C	8.6567170	0.5268190	0.7160130
C	6.7217040	4.8666160	0.5572930
C	6.8431150	6.2404300	0.3684770
C	8.0445360	6.8864830	0.6320610
C	9.1183050	6.1458320	1.1167450
N	7.4713110	-4.8939530	0.1817890
C	8.4248560	-4.4723010	-0.7691960
C	9.4534890	-5.3431080	-1.1695810
S	9.4373900	-7.0282130	-0.6389950
C	8.7810710	-6.7950310	0.9845060
C	7.8218500	-5.7949070	1.2123600
C	10.4307810	-4.9225380	-2.0670230
C	10.3758500	-3.6530970	-2.6346070
C	9.3343030	-2.8036880	-2.2839510
C	8.3750710	-3.2018850	-1.3563300
C	7.2243500	-5.7276550	2.4759820
C	7.5732970	-6.6282610	3.4777270
C	8.5452640	-7.5954950	3.2534510
C	9.1550390	-7.6647990	2.0047450
C	-4.0608670	2.2855930	1.1017630
C	-5.3261830	1.8684370	1.5397420
C	-6.4257040	2.7068770	1.4842870
C	-6.3118810	4.0176010	0.9914340
C	-5.0449380	4.4417160	0.5549940
C	-3.9503780	3.5952210	0.6161030
N	-7.4288300	4.8637180	0.9204940
C	-8.7399940	4.3909610	1.1872070
C	-9.6818510	4.3877080	0.1504600
S	-9.1889320	4.9731600	-1.4478500
C	-8.1519010	6.3012560	-0.8980240

C	-7.3725280	6.1132520	0.2495640
C	-10.9847320	3.9477480	0.3867530
C	-11.3428930	3.4962560	1.6525090
C	-10.4140340	3.5155330	2.6916560
C	-9.1246120	3.9831010	2.4653950
C	-6.5987270	7.1676830	0.7351310
C	-6.5434250	8.3706790	0.0399690
C	-7.2954790	8.5424070	-1.1207770
C	-8.1172090	7.5179670	-1.5782140
H	4.3104140	0.5933040	-1.0544070
H	6.4485610	1.8686150	-0.9721720
H	5.7806410	4.3755950	0.3353260
H	5.9879140	6.8006230	0.0016640
H	8.1475380	7.9553180	0.4711970
H	10.0659340	6.6288340	1.3395240
H	12.1728390	1.8985630	1.5185090
H	11.9115940	-0.4638480	0.7766040
H	9.6281940	-1.3319020	0.2792810
H	7.6763020	0.1249350	0.4873250
H	6.0250740	2.3242800	3.2600000
H	3.8560510	1.0911390	3.1790520
H	5.4514050	-1.1273040	1.8637880
H	7.4186540	-2.5698380	1.5187240
H	6.4720020	-4.9729360	2.6746850
H	7.0822840	-6.5575260	4.4442150
H	8.8281400	-8.2917730	4.0371350
H	9.9171380	-8.4127350	1.8031540
H	11.2270810	-5.6120730	-2.3340610
H	11.1339550	-3.3400150	-3.3459630
H	9.2635750	-1.8107880	-2.7191100
H	7.5726460	-2.5201360	-1.0989450
H	5.0126900	-5.3918160	-0.6183660
H	3.0104930	-3.9472900	-0.2761050
C	-1.1985170	-2.7759360	0.7954950
H	-2.9871190	3.9502360	0.2623640
H	-4.9152000	5.4334970	0.1384130
H	-6.0321540	7.0235560	1.6504740
H	-5.9235760	9.1801720	0.4142200
H	-7.2621520	9.4853950	-1.6587220
H	-8.7381350	7.6577630	-2.4584870
H	-11.7114060	3.9678010	-0.4203250
H	-12.3558980	3.1480560	1.8328090
H	-10.6974300	3.1771050	3.6836000
H	-8.3958870	4.0229210	3.2695840
H	-7.3822480	2.3261360	1.8229500
H	-5.4629800	0.8720890	1.9444240
H	-4.3018400	-0.5469850	-1.0511420
H	-6.4602540	-1.7891160	-1.0595310
H	-5.8744180	-4.4158750	-0.0178790
H	-6.1937070	-6.7753290	-0.6260160

H	-8.4013660	-7.8784450	-0.2705610
H	-10.2514700	-6.5777520	0.7683320
H	-12.1392050	-1.8084570	1.5396470
H	-11.7726490	0.6131720	1.0774970
H	-9.4617890	1.4271250	0.6417430
H	-7.5803660	-0.1313120	0.6281110
H	-6.0019520	-2.6323420	3.1092790
H	-3.8141070	-1.4292620	3.1148900
C	1.2207960	2.6386330	1.0406410
C	1.6170790	2.9839750	-0.4006700
H	0.5051230	3.3771230	1.4186050
H	2.1095770	2.6981280	1.6798900
C	2.4943720	4.2233270	-0.5032550
H	2.1580240	2.1316460	-0.8319990
H	0.7078820	3.1056560	-1.0050540
C	2.9169810	4.5340550	-1.9354090
H	1.9741270	5.0914760	-0.0729780
H	3.3918930	4.0681480	0.1147440
C	3.9008570	5.6934280	-2.0286050
H	3.3768650	3.6368220	-2.3762000
H	2.0266890	4.7496910	-2.5438570
C	4.3422120	6.0270220	-3.4485660
H	3.4601640	6.5876610	-1.5625320
H	4.7918420	5.4471910	-1.4362260
C	5.3797400	7.1441210	-3.4964570
H	4.7589760	5.1245110	-3.9197390
H	3.4673310	6.3089450	-4.0526310
C	5.8542990	7.4753890	-4.9067170
H	4.9601690	8.0492990	-3.0319490
H	6.2446170	6.8603490	-2.8781740
C	6.8953020	8.5894080	-4.9483830
H	6.2730330	6.5703480	-5.3715050
H	4.9906380	7.7610780	-5.5256880
C	7.3666050	8.9104940	-6.3612630
H	6.4756640	9.4933460	-4.4845960
H	7.7557950	8.3018920	-4.3280150
H	8.1128130	9.7125280	-6.3658780
H	7.8192940	8.0310260	-6.8348210
H	6.5291650	9.2300250	-6.9932230
C	-1.6306570	-2.9810850	-0.6622750
H	-0.4776340	-3.5489120	1.0843330
H	-2.0726010	-2.8951820	1.4466410
C	-2.5258430	-4.1963530	-0.8581360
H	-2.1705790	-2.0868510	-0.9993080
H	-0.7370900	-3.0553050	-1.2970430
C	-2.9933820	-4.3630280	-2.3002310
H	-2.0048100	-5.1070310	-0.5284690
H	-3.4041980	-4.0921760	-0.2028790
C	-3.9870880	-5.5043320	-2.4760050
H	-3.4614280	-3.4251510	-2.6350890

H	-2.1240260	-4.5203800	-2.9550810
C	-4.4905370	-5.6812130	-3.9035450
H	-3.5333070	-6.4443410	-2.1278560
H	-4.8503320	-5.3213550	-1.8227550
C	-5.5444890	-6.7771010	-4.0254640
H	-4.9143370	-4.7300850	-4.2584340
H	-3.6449540	-5.9051810	-4.5703270
C	-6.0769720	-6.9570050	-5.4424120
H	-5.1207210	-7.7295330	-3.6726120
H	-6.3816080	-6.5488610	-3.3487490
C	-7.1368550	-8.0480740	-5.5552130
H	-6.4983420	-6.0042590	-5.7962280
H	-5.2421500	-7.1889340	-6.1205740
C	-7.6670350	-8.2181060	-6.9734560
H	-6.7146020	-8.9995710	-5.2021430
H	-7.9681520	-7.8142900	-4.8752640
H	-8.4260370	-9.0060950	-7.0294580
H	-8.1228290	-7.2894230	-7.3374120
H	-6.8597270	-8.4823570	-7.6671390

5. **2e**

C	11.1901830	1.0033660	0.6494960
C	10.1583460	1.8973620	0.9509550
C	8.8412800	1.4103470	0.9504120
C	8.6100140	0.0481300	0.6875340
C	9.6559010	-0.8114180	0.3898500
C	10.9618640	-0.3349090	0.3624000
N	7.7602250	2.2659940	1.2244340
C	7.9603630	3.6007060	1.5938470
C	9.2548860	4.1497480	1.6246260
C	10.5000880	3.3506630	1.2573550
C	9.3916180	5.4910510	1.9917950
C	8.3091620	6.2921560	2.3317220
C	7.0352020	5.7367660	2.2990030
C	6.8630320	4.4110120	1.9312430
C	6.4429650	1.7696350	1.0173750
C	5.5966720	1.4983210	2.0933690
C	4.3516640	0.9152180	1.8740630
C	3.9544700	0.5460170	0.5844790
C	4.7945790	0.8591630	-0.4897660
C	6.0155570	1.4857380	-0.2798930
C	2.7589570	-0.2923770	0.3476440
C	1.3683010	0.0704540	0.2184210
C	0.6995930	-1.1338780	-0.1048330
O	1.5955600	-2.1626830	-0.1767220
C	2.8260730	-1.6382660	0.0992830
C	-0.6598400	-1.2789800	-0.3264810
C	-1.3681420	-0.0698250	-0.2179260
C	-0.6994430	1.1345000	0.1053360
C	0.6599910	1.2795920	0.3270490
C	-2.7588190	0.2929740	-0.3470210

C	-2.8259290	1.6388570	-0.0986700
O	-1.5954300	2.1633020	0.1773060
C	-3.9543210	-0.5453950	-0.5839380
C	-4.3511680	-0.9149850	-1.8735140
C	-5.5960900	-1.4982130	-2.0929490
C	-6.4426230	-1.7692930	-1.0170890
C	-6.0155790	-1.4849730	0.2802020
C	-4.7946920	-0.8582530	0.4901890
N	-7.7597220	-2.2659860	-1.2244260
C	-7.9593570	-3.6009530	-1.5932060
C	-9.2537420	-4.1502870	-1.6245610
C	-10.4993960	-3.3511660	-1.2589170
C	-10.1580580	-1.8978220	-0.9522770
C	-8.8410800	-1.4105500	-0.9509870
C	-9.3899490	-5.4918160	-1.9910890
C	-8.3071060	-6.2928720	-2.3299080
C	-7.0332870	-5.7371850	-2.2966650
C	-6.8616380	-4.4112090	-1.9294600
C	-11.1902640	-1.0039950	-0.6515950
C	-10.9623920	0.3343460	-0.3644490
C	-9.6564970	0.8110830	-0.3910480
C	-8.6102490	-0.0482840	-0.6880010
C	-11.4882780	-3.3946170	-2.4413920
C	-11.1544250	-3.9851530	-0.0156730
C	-3.9725370	2.5341810	-0.0073540
C	-5.1010550	2.3255950	-0.8154430
C	-6.2434150	3.0863070	-0.6361830
C	-6.2829520	4.0724100	0.3492820
C	-5.1472850	4.3334020	1.1134760
C	-3.9958390	3.5735010	0.9321250
C	1.3184900	2.6018890	0.5850730
C	2.0413840	3.1346970	-0.6608740
C	3.0536990	4.2278330	-0.3412700
C	4.0074690	4.5203290	-1.4953340
C	5.0832320	5.5400200	-1.1293310
C	6.3215360	5.4814410	-2.0172150
C	7.4349010	6.4045010	-1.5348240
C	8.7299090	6.2687180	-2.3261040
C	9.8548620	7.1425400	-1.7813290
C	11.1712410	6.9536550	-2.5240880
C	-1.3184680	-2.6012660	-0.5842520
C	-2.0412770	-3.1338140	0.6618370
C	-3.0534690	-4.2271600	0.3425760
C	-4.0071140	-4.5193900	1.4968110
C	-5.0824840	-5.5398350	1.1315720
C	-6.3216840	-5.4793650	2.0180920
C	-7.4345250	-6.4036690	1.5369040
C	-8.7302890	-6.2659140	2.3266260
C	-9.8550560	-7.1402650	1.7823210
C	-11.1717820	-6.9503950	2.5242120

C	3.9725980	-2.5336700	0.0078070
C	3.9954390	-3.5732270	-0.9314430
C	5.1466860	-4.3333740	-1.1129620
C	6.2826300	-4.0724290	-0.3491500
C	6.2435700	-3.0860680	0.6360660
C	5.1014090	-2.3250800	0.8154770
N	-7.5100720	4.7593610	0.5852680
C	-8.4964760	4.0739010	1.3061470
C	-9.8419630	4.4725690	1.2478700
C	-10.3118680	5.6610650	0.4169070
C	-9.1457100	6.3417060	-0.2922630
C	-7.8256760	5.8705010	-0.2058620
C	-10.7768880	3.7306520	1.9760870
C	-10.4236280	2.6310990	2.7466910
C	-9.0852100	2.2571760	2.8065290
C	-8.1322590	2.9710340	2.0984780
C	-6.8045410	6.5233000	-0.9147620
C	-7.0796090	7.6347420	-1.6953560
C	-8.3806810	8.1191280	-1.7843410
C	-9.3872070	7.4666810	-1.0849780
C	-11.0040570	6.6792310	1.3442750
C	-11.3139450	5.1599880	-0.6430370
N	7.5095170	-4.7597220	-0.5852760
C	7.8249200	-5.8710100	0.2057100
C	9.1447910	-6.3427630	0.2916200
C	10.3109900	-5.6624970	-0.4178470
C	9.8412050	-4.4740920	-1.2490100
C	8.4958590	-4.0748690	-1.3067900
C	9.3861150	-7.4678290	1.0842610
C	8.3795720	-8.1198640	1.7839840
C	7.0786660	-7.6349460	1.6954640
C	6.8037710	-6.5233870	0.9149780
C	8.1317490	-2.9720380	-2.0992170
C	9.0846890	-2.2587450	-2.8078540
C	10.4229740	-2.6332160	-2.7485180
C	10.7761170	-3.7327340	-1.9778040
C	11.3132370	-5.1613280	0.6418970
C	11.0029410	-6.6809780	-1.3450380
C	11.4905180	3.3940120	2.4385270
C	11.1534770	3.9848130	0.0133200
H	-10.3042670	7.0456430	2.1018070
H	-11.3760510	7.5394780	0.7782900
H	-11.8584380	6.2271700	1.8586360
H	-12.1800440	4.6831250	-0.1717060
H	-10.8395550	4.4271690	-1.3039750
H	-11.6821310	5.9869730	-1.2592620
H	-10.4048920	7.8401910	-1.1647370
H	-8.6112150	8.9893420	-2.3916880
H	-6.2704490	8.1210490	-2.2334890
H	-5.7881710	6.1504610	-0.8489770

H	-5.1863220	5.1128020	1.8689460
H	-3.1187400	3.7550440	1.5458480
H	5.0852360	-1.5575120	1.5802230
H	7.1260880	-2.9121330	1.2457680
H	7.0922210	-2.6676470	-2.1453400
H	8.7765950	-1.4018160	-3.4001740
H	11.1817490	-2.0768440	-3.2902980
H	11.8220270	-4.0253300	-1.9332390
H	11.6813300	-5.9882360	1.2582850
H	10.8390190	-4.4282790	1.3027020
H	12.1793590	-4.6847020	0.1703640
H	11.8573410	-6.2291780	-1.8595920
H	11.3748890	-7.5411410	-0.7789010
H	10.3030250	-7.0474750	-2.1024130
H	10.4036820	-7.8417310	1.1636910
H	8.6099680	-8.9901670	2.3912560
H	6.2695040	-8.1209250	2.2338880
H	5.7875320	-6.1501270	0.8495640
H	5.1853470	-5.1129780	-1.8682410
H	3.1181110	-3.7547730	-1.5448320
H	4.4927380	0.5796900	-1.4952560
H	6.6778980	1.7060140	-1.1121100
H	7.5984360	-0.3409810	0.7010270
H	9.4459840	-1.8526650	0.1621200
H	11.7861450	-0.9974350	0.1160590
H	12.2115500	1.3749200	0.6322730
H	11.0420350	2.9475480	3.3314780
H	12.4072770	2.8432930	2.2055260
H	11.7776000	4.4229730	2.6777500
H	10.4618600	3.9650460	-0.8348610
H	12.0606450	3.4421480	-0.2733390
H	11.4309590	5.0276680	0.2004750
H	10.3871790	5.9267450	2.0074230
H	8.4601150	7.3308520	2.6094480
H	6.1628460	6.3346560	2.5485620
H	5.8612420	3.9991710	1.8968520
H	5.9334070	1.7230420	3.1013760
H	3.7092040	0.6762390	2.7176620
H	-4.4930980	-0.5784930	1.4956730
H	-6.6781040	-1.7050770	1.1123200
H	-5.8599590	-3.9991430	-1.8946320
H	-6.1606350	-6.3350040	-2.5453780
H	-8.4576590	-7.3317460	-2.6071820
H	-10.3854190	-5.9277060	-2.0071840
H	-11.7751220	-4.4236050	-2.6808000
H	-12.4052650	-2.8437190	-2.2096870
H	-11.0385590	-2.9483670	-3.3338290
H	-12.0620660	-3.4425770	0.2696390
H	-10.4639840	-3.9651330	0.8334570
H	-11.4315390	-5.0280750	-0.2029760

H	-12.2115800	-1.3757310	-0.6351010
H	-11.7869710	0.9967470	-0.1187690
H	-9.4468970	1.8523730	-0.1632260
H	-7.5987490	0.3410540	-0.7008570
H	-5.9325490	-1.7232970	-3.1009680
H	-3.7084950	-0.6762400	-2.7170170
H	-5.0845000	1.5581900	-1.5803470
H	-7.1257340	2.9123380	-1.2461670
H	-11.8228930	4.0228490	1.9311600
H	-11.1824060	2.0742710	3.2879950
H	-8.7770190	1.4002230	3.3987650
H	-7.0926240	2.6670680	2.1449970
H	-2.0514690	-2.4845640	-1.3921570
H	-0.5752690	-3.3274370	-0.9310700
H	-1.3022880	-3.4852950	1.3937810
H	-2.5692490	-2.2998740	1.1400940
H	-3.6562550	-3.8992820	-0.5181230
H	-2.5403300	-5.1463660	0.0251330
H	-3.4476240	-4.8488920	2.3837330
H	-4.4945370	-3.5743750	1.7769590
H	-5.4039710	-5.3587800	0.0979100
H	-4.6603880	-6.5549650	1.1429030
H	-6.0592920	-5.7099830	3.0612290
H	-6.6988730	-4.4449780	2.0195780
H	-7.6397580	-6.1885420	0.4790980
H	-7.0895680	-7.4477840	1.5749380
H	-8.5542760	-6.5078100	3.3855600
H	-9.0514280	-5.2134020	2.3066480
H	-9.9931760	-6.9133730	0.7158910
H	-9.5497240	-8.1950520	1.8309440
H	-11.9613390	-7.5936610	2.1197400
H	-11.0630150	-7.1860320	3.5898360
H	-11.5172040	-5.9119190	2.4502150
H	0.5752110	3.3279420	0.9319590
H	2.0514430	2.4851060	1.3930140
H	2.5692580	2.3008230	-1.1393460
H	1.3024490	3.4864750	-1.3927270
H	2.5406460	5.1470070	-0.0235900
H	3.6563850	3.8996360	0.5193780
H	4.4945190	3.5752960	-1.7760780
H	3.4481650	4.8506860	-2.3820600
H	4.6610820	6.5551600	-1.1381360
H	5.4058110	5.3568740	-0.0963800
H	6.6986170	4.4470200	-2.0215180
H	6.0582220	5.7145680	-3.0595600
H	7.0899190	7.4487150	-1.5698600
H	7.6411660	6.1867350	-0.4777560
H	9.0514960	5.2163020	-2.3083650
H	8.5527500	6.5124560	-3.3844250
H	9.5493990	8.1973510	-1.8285480

H	9.9935410	6.9144150	-0.7152330
H	11.9609190	7.5965890	-2.1193270
H	11.5168220	5.9151390	-2.4513630
H	11.0619180	7.1904040	-3.5894090

6. **2f**

C	8.3581970	-7.3766800	-3.0160460
C	8.1917540	-5.9942780	-2.9318160
C	6.9328720	-5.4233210	-3.2233460
C	5.8489460	-6.2149020	-3.5963600
C	6.0441040	-7.5886860	-3.6586690
C	7.2834840	-8.1880490	-3.3699640
N	7.0072010	-4.0458130	-3.0354270
C	8.2998110	-3.7264960	-2.6273220
C	9.0660580	-4.9093690	-2.5535980
C	10.4078110	-4.8410440	-2.1720960
C	10.9860100	-3.6081110	-1.8858500
C	10.2050180	-2.4427840	-2.0066620
C	8.8666720	-2.4787920	-2.3704270
C	5.9242210	-3.1475500	-3.0634220
C	5.6778580	-2.3277270	-1.9581670
C	4.6358970	-1.4149630	-1.9903770
C	3.8085700	-1.3022320	-3.1150390
C	4.0442480	-2.1496790	-4.2020280
C	5.0963740	-3.0601960	-4.1840870
C	2.7888060	-0.2382900	-3.1616170
C	3.0783160	1.1008890	-3.0975410
O	1.9444070	1.8602640	-3.0959130
C	0.8853830	0.9988920	-3.1605030
C	1.3485460	-0.3381140	-3.1938040
C	0.4378040	-1.4104720	-3.1598130
C	-0.8845420	-0.9978440	-3.1602410
C	-1.3477100	0.3391560	-3.1934940
C	-0.4369620	1.4115200	-3.1598010
C	-2.7879560	0.2393390	-3.1609360
C	-3.0774660	-1.0998380	-3.0967440
O	-1.9435540	-1.8592100	-3.0953660
C	4.3492590	1.7906600	-2.9288770
C	4.4281890	2.9619160	-2.1622410
C	5.6606570	3.5051080	-1.8234360
C	6.8371530	2.8908490	-2.2593380
C	6.7637540	1.7817380	-3.1061100
C	5.5348890	1.2389860	-3.4382390
N	8.0974820	3.3390540	-1.8228420
C	9.0402470	2.5356170	-1.1882190
C	10.1814620	3.3088720	-0.8919680
C	9.9091990	4.6464750	-1.3637540
C	8.6124580	4.6259110	-1.9248700
C	8.9512380	1.1995920	-0.7984260
C	10.0368330	0.6515120	-0.1317430
C	11.2079710	1.3850650	0.1475380

C	11.2654170	2.7222590	-0.2325090
C	10.6411420	5.8344090	-1.3691000
C	10.0946260	6.9866020	-1.9268880
C	8.8046780	6.9284850	-2.4875670
C	8.0508840	5.7628840	-2.5012890
C	12.3502980	0.6813860	0.8447270
C	13.4479200	1.5749420	1.4095530
C	14.4921010	0.7902850	2.1950320
C	15.6121330	1.6608170	2.7537660
C	16.6247070	0.8710690	3.5744070
C	17.7552890	1.7186010	4.1465130
C	18.7266560	0.9079730	4.9970120
C	19.8716060	1.7262070	5.5818980
C	20.8206170	0.8984840	6.4430860
C	21.9660130	1.7172980	7.0249720
C	10.8433330	8.2929420	-1.8881500
C	10.4674930	9.1512220	-0.6764110
C	11.2180010	10.4768580	-0.6268950
C	10.8511170	11.3323780	0.5808610
C	11.6038770	12.6574080	0.6343270
C	11.2402600	13.5120400	1.8436980
C	11.9949270	14.8359420	1.8981650
C	11.6334570	15.6902100	3.1083440
C	12.3888320	17.0141420	3.1639220
C	12.0200980	17.8586910	4.3773390
C	0.8398660	-2.8516600	-3.0337250
C	1.4098210	-3.1789540	-1.6447240
C	2.2185980	-4.4725170	-1.6146260
C	3.0838810	-4.6030220	-0.3638980
C	4.0202870	-5.8157250	-0.3864370
C	5.3325020	-5.5617000	0.3488680
C	6.3250370	-6.7172170	0.2796890
C	7.7042500	-6.3297610	0.8031570
C	8.7276560	-7.4574300	0.7367370
C	10.1179200	-7.0167530	1.1788540
C	-0.8390180	2.8527210	-3.0338440
C	-1.4086670	3.1802770	-1.6447770
C	-2.2176160	4.4737370	-1.6148180
C	-3.0825240	4.6045210	-0.3638590
C	-4.0192210	5.8169970	-0.3865610
C	-5.3310210	5.5630240	0.3495010
C	-6.3238900	6.7182510	0.2802420
C	-7.7027680	6.3306400	0.8044790
C	-8.7265200	7.4579940	0.7380540
C	-10.1164660	7.0170980	1.1809610
C	-3.8077170	1.3032730	-3.1141320
C	-4.6345670	1.4161760	-1.9891430
C	-5.6766480	2.3287970	-1.9567270
C	-5.9236140	3.1482790	-3.0620960
C	-5.0961880	3.0608130	-4.1830680

C	-4.0439270	2.1504580	-4.2012130
C	-4.3483530	-1.7896480	-2.9277930
C	-4.4269960	-2.9611840	-2.1615520
C	-5.6593320	-3.5046070	-1.8226260
C	-6.8359950	-2.8902930	-2.2579990
C	-6.7629060	-1.7808330	-3.1043370
C	-5.5341740	-1.2378660	-3.4366030
N	-7.0068390	4.0462390	-3.0339620
C	-8.2991610	3.7266340	-2.6251820
C	-9.0657510	4.9092800	-2.5514140
C	-8.1919540	5.9943590	-2.9303250
C	-6.9330180	5.4237210	-3.2222590
C	-10.4073020	4.8406360	-2.1692300
C	-10.9849500	3.6075940	-1.8823530
C	-10.2036370	2.4424840	-2.0032440
C	-8.8654930	2.4788140	-2.3676830
C	-5.8494880	6.2155440	-3.5959070
C	-6.0450900	7.5892540	-3.6584520
C	-7.2845330	8.1883030	-3.3693650
C	-8.3588500	7.3766900	-3.0148060
C	-12.4065330	3.5034600	-1.3976220
C	-12.4960650	3.3493990	0.1239590
C	-13.9290790	3.2311050	0.6274370
C	-14.0287460	3.0828460	2.1414740
C	-15.4668770	3.0285970	2.6443770
C	-15.5793800	2.8169100	4.1497420
C	-17.0194060	2.8201340	4.6508270
C	-17.1483960	2.4993990	6.1353730
C	-18.5864460	2.5458320	6.6412950
C	-18.7163410	2.1368190	8.1028200
C	-7.4147230	9.6880910	-3.3492250
C	-7.0362040	10.2731370	-1.9844380
C	-7.1615490	11.7901580	-1.9204870
C	-6.8027710	12.3605290	-0.5525420
C	-6.9343600	13.8775910	-0.4748350
C	-6.5815230	14.4459310	0.8954210
C	-6.7153820	15.9628500	0.9744670
C	-6.3661290	16.5313520	2.3454920
C	-6.5008090	18.0485800	2.4255460
C	-6.1506610	18.6049660	3.8001040
N	-8.0961880	-3.3388410	-1.8214490
C	-8.6111050	-4.6256680	-1.9241090
C	-9.9077750	-4.6466050	-1.3628360
C	-10.1800500	-3.3092740	-0.8902910
C	-9.0389150	-2.5358050	-1.1862690
C	-10.6396430	-5.8345820	-1.3687150
C	-10.0931300	-6.9864430	-1.9271930
C	-8.8032600	-6.9279540	-2.4880100
C	-8.0495390	-5.7622990	-2.5012100
C	-8.9499260	-1.1999690	-0.7958060

C	-10.0354630	-0.6523040	-0.1287100
C	-11.2065280	-1.3860750	0.1503500
C	-11.2639690	-2.7230600	-0.2303890
C	-10.8417540	-8.2928470	-1.8889290
C	-10.4665360	-9.1510980	-0.6769810
C	-11.2170480	-10.4767440	-0.6278090
C	-10.8506990	-11.3322340	0.5801290
C	-11.6034800	-12.6572640	0.6333130
C	-11.2402270	-13.5119360	1.8427650
C	-11.9948940	-14.8358500	1.8969630
C	-11.6336960	-15.6901910	3.1071710
C	-12.3890890	-17.0141240	3.1625090
C	-12.0205920	-17.8587580	4.3759390
C	-12.3486420	-0.6827320	0.8482280
C	-13.4480590	-1.5761640	1.4096960
C	-14.4924970	-0.7917520	2.1950820
C	-15.6143670	-1.6619120	2.7506950
C	-16.6270750	-0.8722820	3.5712880
C	-17.7596330	-1.7192350	4.1403290
C	-18.7309700	-0.9086860	4.9909420
C	-19.8779890	-1.7262050	5.5727590
C	-20.8268530	-0.8985550	6.4341820
C	-21.9743730	-1.7165930	7.0129640
C	12.4078760	-3.5044400	-1.4018240
C	12.4978020	-3.3505940	0.1197650
C	13.9308040	-3.2324240	0.6232980
C	14.0301070	-3.0850080	2.1374470
C	15.4680050	-3.0303610	2.6409510
C	15.5795720	-2.8202900	4.1466200
C	17.0192870	-2.8228470	4.6485790
C	17.1469480	-2.5043340	6.1337240
C	18.5846950	-2.5499900	6.6405670
C	18.7130700	-2.1435240	8.1029360
C	7.4132200	-9.6878720	-3.3495260
C	7.0351750	-10.2724770	-1.9844130
C	7.1600630	-11.7895240	-1.9201770
C	6.8018480	-12.3594610	-0.5519050
C	6.9329790	-13.8765490	-0.4739170
C	6.5806820	-14.4444540	0.8966570
C	6.7141000	-15.9613970	0.9759940
C	6.3653950	-16.5294600	2.3473390
C	6.4996080	-18.0467140	2.4276790
C	6.1500330	-18.6026550	3.8025630
H	5.3026590	-3.6827220	-5.0495630
H	3.4253770	-2.0601180	-5.0908470
H	5.4921130	0.3727730	-4.0889710
H	7.6817510	1.3465390	-3.4900760
H	8.0565640	0.6135760	-0.9869800
H	9.9791840	-0.3852280	0.1937770
H	12.1430130	3.3229290	-0.0112530

H	11.6416910	5.8608860	-0.9417760
H	8.3877580	7.8297940	-2.9322090
H	7.0646900	5.7357160	-2.9549360
H	5.7213320	4.3744070	-1.1752920
H	3.5147890	3.4100190	-1.7829400
H	-3.4254000	2.0607730	-5.0902590
H	-5.3029340	3.6830830	-5.0486180
H	-8.2894630	1.5631690	-2.4640770
H	-10.6652880	1.4770450	-1.8098780
H	-11.0020180	5.7493330	-2.1007950
H	-9.3244790	7.8237680	-2.7869130
H	-5.2077290	8.2265720	-3.9342350
H	-4.8797030	5.7802430	-3.8155700
H	-6.3070280	2.4177410	-1.0770500
H	-4.4554360	0.7680040	-1.1360460
H	-5.4916610	-0.3714350	-4.0870590
H	-7.6810380	-1.3455650	-3.4879030
H	-7.0634040	-5.7348230	-2.9549680
H	-8.3863480	-7.8290080	-2.9331710
H	-11.6401450	-5.8613370	-0.9412910
H	-12.1415210	-3.3238800	-0.0093750
H	-9.9778540	0.3842760	0.1973340
H	-8.0553230	-0.6137990	-0.9842170
H	-5.7197670	-4.3741720	-1.1748170
H	-3.5134590	-3.4093730	-1.7826820
H	4.4571970	-0.7665730	-1.1373540
H	6.3085750	-2.4165720	-1.0787200
H	4.8792040	-5.7793550	-3.8157270
H	5.2064340	-8.2258120	-3.9339590
H	9.3237930	-7.8239920	-2.7884750
H	8.2908870	-1.5629890	-2.4667800
H	10.6670700	-1.4774420	-1.8137670
H	11.0022710	-5.7499140	-2.1037070
H	-0.0198430	-3.4937400	-3.2531880
H	1.6028320	-3.0807880	-3.7872310
H	0.0206250	3.4947810	-3.2536270
H	-1.6021630	3.0816900	-3.7872200
H	-2.0632240	2.3573420	-1.3335880
H	-0.5924180	3.2111700	-0.9109110
H	-1.5581940	5.3466670	-1.7248570
H	-2.8874230	4.4817160	-2.4877780
H	-3.6907760	3.6921430	-0.2896770
H	-2.4530420	4.6208550	0.5366960
H	-3.5156970	6.7022830	0.0260940
H	-4.2624990	6.0685510	-1.4273710
H	-5.8018920	4.6744590	-0.0975780
H	-5.1317620	5.3021870	1.3996310
H	-5.9362570	7.5805340	0.8429790
H	-6.4200120	7.0493260	-0.7631950
H	-8.0774720	5.4795270	0.2167120

H	-7.6195100	5.9732120	1.8422120
H	-8.3860120	8.3019450	1.3550990
H	-8.7715470	7.8314430	-0.2937450
H	-10.8421080	7.8363380	1.1221670
H	-10.4803210	6.1979920	0.5482230
H	-10.1060770	6.6541270	2.2160080
H	2.0645700	-2.3560310	-1.3339080
H	0.5937490	-3.2095440	-0.9106480
H	1.5590220	-5.3453920	-1.7241780
H	2.8881280	-4.4808660	-2.4877960
H	3.6923600	-3.6907500	-0.2902670
H	2.4546800	-4.6188610	0.5368630
H	3.5167560	-6.7006860	0.0269040
H	4.2630110	-6.0678650	-1.4272340
H	5.8033770	-4.6734700	-0.0988720
H	5.1338100	-5.3003070	1.3989670
H	5.9374170	-7.5791500	0.8429710
H	6.4206180	-7.0487690	-0.7636450
H	8.0789250	-5.4789920	0.2148740
H	7.6215520	-5.9718870	1.8407810
H	8.3871780	-8.3010390	1.3542660
H	8.7721290	-7.8313050	-0.2949310
H	10.8433020	-7.8362260	1.1200880
H	10.4817320	-6.1980190	0.5456110
H	10.1080880	-6.6533390	2.2137520
H	11.9243730	8.1005870	-1.8702650
H	10.6463810	8.8635170	-2.8061580
H	9.3842520	9.3376620	-0.6907860
H	10.6628230	8.5780520	0.2407930
H	12.3008860	10.2822390	-0.6195640
H	11.0193800	11.0434270	-1.5489080
H	9.7686970	11.5283270	0.5727680
H	11.0482540	10.7642100	1.5019370
H	12.6862770	12.4606260	0.6403010
H	11.4054930	13.2262510	-0.2862120
H	10.1581000	13.7099330	1.8374850
H	11.4377510	12.9425370	2.7639940
H	13.0770670	14.6375580	1.9031290
H	11.7966740	15.4058430	0.9782140
H	10.5514050	15.8895860	3.1037820
H	11.8312670	15.1205230	4.0286390
H	13.4694860	16.8133900	3.1682180
H	12.1900370	17.5823030	2.2442400
H	12.5746040	18.8033130	4.3959410
H	10.9502120	18.0996590	4.3793770
H	12.2387420	17.3249150	5.3100730
H	11.9373850	0.0697800	1.6602980
H	12.8063740	-0.0395930	0.1481280
H	13.9454250	2.1168610	0.5929870
H	12.9996570	2.3394810	2.0597590

H	13.9995890	0.2595460	3.0234200
H	14.9234620	0.0087010	1.5518590
H	16.1271300	2.1707330	1.9263030
H	15.1782640	2.4561440	3.3776390
H	16.1032890	0.3632720	4.3993350
H	17.0506160	0.0703710	2.9516620
H	18.3019870	2.2049290	3.3252450
H	17.3310030	2.5323890	4.7530050
H	18.1719750	0.4266130	5.8159390
H	19.1397230	0.0871790	4.3914980
H	20.4381240	2.1995450	4.7661860
H	19.4610360	2.5508780	6.1834320
H	20.2515840	0.4281140	7.2573900
H	21.2254890	0.0719070	5.8418040
H	22.6315430	1.1008820	7.6391160
H	22.5701460	2.1710520	6.2301100
H	21.5878690	2.5306220	7.6560070
H	12.9014450	-2.6450630	-1.8769070
H	12.9726510	-4.3948270	-1.7078590
H	11.9234480	-2.4645520	0.4260710
H	12.0055940	-4.2096100	0.5969510
H	14.5020610	-4.1170890	0.3046570
H	14.4168280	-2.3697310	0.1422120
H	13.4987010	-2.1736490	2.4483560
H	13.5063340	-3.9224780	2.6212220
H	15.9856410	-3.9599120	2.3611100
H	16.0038790	-2.2194480	2.1251840
H	15.1016160	-1.8658840	4.4124690
H	15.0087530	-3.6009370	4.6707910
H	17.4742490	-3.8029680	4.4422890
H	17.6053350	-2.0901080	4.0747490
H	16.7262340	-1.5051830	6.3240240
H	16.5318260	-3.2057500	6.7169290
H	18.9855830	-3.5635140	6.4998650
H	19.2065810	-1.8884350	6.0221270
H	19.7535810	-2.1840790	8.4435660
H	18.3520730	-1.1190560	8.2579020
H	18.1221880	-2.8015350	8.7514850
H	8.4439030	-9.9780490	-3.5931960
H	6.7734190	-10.1329650	-4.1234740
H	7.6710890	-9.8157330	-1.2133080
H	6.0066910	-9.9717050	-1.7389570
H	6.5147780	-12.2441790	-2.6865830
H	8.1888360	-12.0820360	-2.1783510
H	7.4450920	-11.8981760	0.2120920
H	5.7725670	-12.0685680	-0.2949720
H	6.2874050	-14.3377040	-1.2360910
H	7.9618900	-14.1664370	-0.7340660
H	7.2259080	-13.9821300	1.6583680
H	5.5516280	-14.1553700	1.1569310

H	6.0675020	-16.4237990	0.2153970
H	7.7428320	-16.2500410	0.7136500
H	7.0117750	-16.0671180	3.1082820
H	5.3365300	-16.2415930	2.6103700
H	5.8528290	-18.5072870	1.6676140
H	7.5276450	-18.3327340	2.1640870
H	6.2537730	-19.6927270	3.8361480
H	6.8036290	-18.1812140	4.5758120
H	5.1166940	-18.3571830	4.0758360
H	-11.9228250	-8.1005950	-1.8716860
H	-10.6441910	-8.8633860	-2.8068220
H	-9.3832840	-9.3375210	-0.6908240
H	-10.6623270	-8.5779130	0.2401160
H	-12.2999410	-10.2821510	-0.6209910
H	-11.0179770	-11.0433200	-1.5497200
H	-9.7682750	-11.5281830	0.5725120
H	-11.0482350	-10.7640420	1.5011050
H	-12.6858810	-12.4604810	0.6389670
H	-11.4048180	-13.2260750	-0.2871860
H	-10.1580630	-13.7098150	1.8368720
H	-11.4380020	-12.9424680	2.7630210
H	-13.0770370	-14.6374790	1.9016870
H	-11.7964170	-15.4056870	0.9770210
H	-10.5516430	-15.8895720	3.1028300
H	-11.8317010	-15.1205590	4.0274570
H	-13.4697420	-16.8133670	3.1666120
H	-12.1901190	-17.5822200	2.2428260
H	-12.5751020	-18.8033820	4.3943660
H	-10.9507070	-18.0997260	4.3781670
H	-12.2394170	-17.3250480	5.3086680
H	-12.8030710	0.0405510	0.1529520
H	-11.9357620	-0.0736680	1.6657240
H	-13.9449090	-2.1159570	0.5913220
H	-13.0016770	-2.3424350	2.0591600
H	-14.0005670	-0.2630280	3.0251060
H	-14.9220360	-0.0085400	1.5526720
H	-16.1288380	-2.1697290	1.9216150
H	-15.1823700	-2.4588690	3.3737870
H	-16.1061430	-0.3666230	4.3978360
H	-17.0510230	-0.0699240	2.9493440
H	-18.3059090	-2.2033120	3.3174530
H	-17.3373470	-2.5347070	4.7459550
H	-18.1767040	-0.4296440	5.8115070
H	-19.1419450	-0.0861710	4.3863380
H	-20.4441450	-2.1971450	4.7554090
H	-19.4695370	-2.5526270	6.1733310
H	-20.2581800	-0.4306230	7.2501410
H	-21.2295550	-0.0702040	5.8338830
H	-22.6397610	-1.1002420	7.6273270
H	-22.5781320	-2.1678980	6.2164250

H	-21.5984170	-2.5316950	7.6430120
H	-12.9716840	4.3937220	-1.7033410
H	-12.9000650	2.6440230	-1.8726410
H	-11.9216390	2.4633370	0.4300690
H	-12.0038680	4.2083840	0.6012100
H	-14.5002000	4.1160010	0.3091920
H	-14.4151680	2.3687400	0.1458200
H	-13.4978780	2.1710390	2.4519790
H	-13.5047330	3.9197800	2.6259100
H	-15.9837700	3.9587940	2.3653100
H	-16.0030660	2.2186190	2.1274650
H	-15.1024060	1.8617810	4.4147480
H	-15.0081840	3.5964590	4.6751330
H	-17.4732490	3.8010310	4.4457730
H	-17.6058240	2.0888890	4.0754680
H	-16.7289160	1.4994790	6.3243470
H	-16.5328950	3.1991960	6.7201220
H	-18.9860310	3.5600890	6.5021770
H	-19.2086850	1.8861600	6.0211960
H	-19.7570370	2.1780450	8.4428050
H	-18.3567320	1.1116150	8.2561350
H	-18.1250900	2.7928890	8.7529970
H	-6.7754260	10.1331900	-4.1235820
H	-8.4456100	9.9778870	-3.5924740
H	-7.6715840	9.8163640	-1.2129090
H	-6.0075000	9.9727580	-1.7394200
H	-6.5168300	12.2448460	-2.6873490
H	-8.1905570	12.0822790	-2.1781640
H	-7.4454430	11.8992080	0.2119140
H	-5.7732530	12.0700340	-0.2961090
H	-6.2893400	14.3387820	-1.2374580
H	-7.9635030	14.1670810	-0.7345060
H	-7.2262020	13.9835820	1.6575790
H	-5.5522400	14.1572350	1.1552220
H	-6.0693350	16.4252760	0.2134180
H	-7.7443450	16.2511040	0.7126020
H	-7.0119430	16.0689720	3.1068920
H	-5.3370260	16.2438930	2.6080350
H	-5.8546070	18.5091900	1.6650140
H	-7.5290890	18.3341890	2.1624560
H	-6.2547560	19.6950090	3.8334890
H	-6.8036840	18.1834840	4.5738150
H	-5.1170870	18.3599120	4.0728620

7. **2g**

C	-11.0550130	3.1834500	0.2635200
C	-9.7412620	3.5487260	-0.0407290
C	-8.7269560	2.5713270	-0.0490270
C	-9.0143650	1.2334240	0.2145340

C	-10.3266650	0.9043060	0.5140420
C	-11.3669540	1.8574940	0.5527770
N	-7.5187660	3.1694780	-0.3944250
C	-7.7443170	4.5225070	-0.6148130
C	-9.1128670	4.8003670	-0.3987760
C	-9.5796390	6.1044270	-0.5396910
C	-8.7075160	7.1398250	-0.8848280
C	-7.3507390	6.8244520	-1.0843500
C	-6.8520730	5.5318640	-0.9553350
C	-6.2942500	2.4866280	-0.5007200
C	-5.8572190	1.6969670	0.5658660
C	-4.7137440	0.9268300	0.4315590
C	-3.9719240	0.9382740	-0.7568740
C	-4.3855170	1.7782990	-1.7952910
C	-5.5401110	2.5446910	-1.6743380
C	-2.8432190	0.0015090	-0.8916680
C	-2.9804400	-1.3626440	-0.8359600
O	-1.7646500	-1.9847970	-0.8553180
C	-0.8126380	-1.0094090	-0.9248030
C	-1.4249150	0.2663970	-0.9606510
C	-0.6358950	1.4308960	-1.0101590
C	0.7243600	1.1636920	-1.0178930
C	1.3350430	-0.1125210	-1.0009660
C	0.5465960	-1.2754270	-0.9450860
O	1.6806990	2.1386780	-1.0399520
C	2.8934970	1.5114970	-1.0348450
C	2.7562610	0.1482440	-1.0106250
C	-4.1460220	-2.2301260	-0.7390410
C	-4.0197270	-3.5209410	-0.2050380
C	-5.1278960	-4.3432310	-0.0612060
C	-6.3902650	-3.8994580	-0.4641940
C	-6.5227000	-2.6261790	-1.0252310
C	-5.4161610	-1.8043830	-1.1620130
N	-7.5171850	-4.7298470	-0.3245910
C	-7.6231080	-6.0379150	-0.7909310
C	-8.9067800	-6.5335180	-0.4905910
C	-9.6115940	-5.4750580	0.1929790
C	-8.7248030	-4.3786110	0.2745310
C	-9.2642780	-7.8282620	-0.8721480
C	-8.3592480	-8.6336780	-1.5576630
C	-7.0890650	-8.0965090	-1.8577180
C	-6.7035230	-6.8156590	-1.4920360
C	-10.8837740	-5.3804150	0.7489480
C	-11.2909040	-4.2159620	1.4026840
C	-10.3770420	-3.1483540	1.4782150
C	-9.1001890	-3.2077070	0.9255810
C	1.0995420	-2.6675390	-0.8587430
C	1.6725780	-2.9955430	0.5264200
C	2.5335580	-4.2549580	0.5252040
C	3.4255750	-4.3750470	1.7568840

C	4.3692840	-5.5763980	1.7031160
C	5.6313730	-5.3915060	2.5386690
C	6.6018880	-6.5621470	2.4332590
C	7.9479540	-6.2867590	3.0929670
C	8.9249160	-7.4509920	2.9730620
C	10.3101010	-7.1203470	3.5135610
C	-1.1671670	2.8348160	-1.0504800
C	-1.7898600	3.3163070	0.2634910
C	-2.4350460	4.6924230	0.1219640
C	-3.4033870	5.0251870	1.2518240
C	-3.9584470	6.4436610	1.1701880
C	-5.1354280	6.6881680	2.1070370
C	-5.6357440	8.1274380	2.0804920
C	-6.8776940	8.3511180	2.9352450
C	-7.3146580	9.8111640	2.9983070
C	-8.5625820	10.0236610	3.8462010
C	3.8873190	-0.7911740	-0.8738890
C	4.6086280	-0.8184570	0.3254630
C	5.7109680	-1.6448220	0.4828190
C	6.1472650	-2.4415040	-0.5798690
C	5.4410510	-2.4183290	-1.7861190
C	4.3130420	-1.6152360	-1.9203030
C	4.0736810	2.3619400	-1.0086620
C	4.0362540	3.6192220	-0.3900380
C	5.1848010	4.3901090	-0.2785680
C	6.3930430	3.9317440	-0.8129600
C	6.4241000	2.7055910	-1.4842330
C	5.2830050	1.9272730	-1.5707820
N	7.2882140	-3.2498590	-0.4285550
C	8.4803250	-2.8642480	0.1861770
C	9.3547470	-3.9719850	0.2283730
C	8.6583480	-5.0769910	-0.3860650
C	7.3919060	-4.5969820	-0.7742490
C	10.6189910	-3.8411530	0.7969370
C	11.0363880	-2.6190020	1.3275640
C	10.1500400	-1.5300890	1.2464670
C	8.8834150	-1.6268260	0.6788600
C	6.4593840	-5.4531700	-1.3581510
C	6.8248680	-6.7781530	-1.5477060
C	8.0880370	-7.2886610	-1.1816940
C	9.0006490	-6.4132400	-0.5994230
N	7.5750760	4.6781100	-0.6720000
C	7.7326930	6.0377390	-0.9322910
C	9.0750380	6.4000440	-0.6841940
C	9.7592730	5.2020420	-0.2565670
C	8.8050840	4.1658290	-0.2618250
C	9.4871990	7.7135670	-0.8899280
C	8.5868460	8.6778500	-1.3477560
C	7.2637460	8.2761870	-1.6047360
C	6.8202400	6.9720840	-1.4095050

C	9.1507190	2.8776250	0.1445120
C	10.4657820	2.6492070	0.5182400
C	11.4531130	3.6568110	0.5149390
C	11.0758310	4.9399820	0.1288430
H	5.7951730	-3.0095590	-2.6248170
H	3.7791620	-1.5931600	-2.8667640
H	5.3240890	0.9795350	-2.0959790
H	7.3481030	2.3750200	-1.9481670
H	8.4198610	2.0757460	0.1746070
H	10.7381910	1.6412600	0.8198590
C	12.8794350	3.3032810	0.9396850
H	11.7948850	5.7528660	0.1229260
H	10.5235270	7.9785020	-0.6944340
C	9.0659690	10.1166450	-1.5596440
H	6.5459790	9.0000920	-1.9750320
H	5.7950740	6.6982960	-1.6376610
H	5.1606080	5.3364250	0.2529740
H	3.1035910	3.9714260	0.0400690
H	-3.8302270	1.7861150	-2.7291360
H	-5.8901000	3.1532240	-2.5029280
H	-8.2427630	0.4698470	0.1792910
H	-10.5516680	-0.1391440	0.7183040
C	-12.7756740	1.4042240	0.9396450
H	-11.8249880	3.9482980	0.2660880
H	-10.6352060	6.3077900	-0.3772380
C	-9.2547550	8.5596490	-1.0560650
H	-6.6501980	7.6090610	-1.3491930
H	-5.7972950	5.3245890	-1.1082150
H	-6.4313230	1.6845740	1.4874270
H	-4.3876480	0.2901730	1.2488420
H	-5.5357040	-0.8316050	-1.6251220
H	-7.4963230	-2.2981950	-1.3775330
H	-5.7218150	-6.4337060	-1.7540110
H	-6.3730080	-8.7052900	-2.4034410
C	-8.6911760	-10.0608920	-1.9979040
H	-10.2581600	-8.1900660	-0.6291930
H	-11.5558220	-6.2319030	0.6753270
C	-12.6969220	-4.1488340	2.0059310
H	-10.6579910	-2.2383650	1.9989340
H	-8.4169560	-2.3687130	1.0193840
H	-5.0275210	-5.3282170	0.3847430
H	-3.0437630	-3.8669050	0.1205280
H	4.2847210	-0.1881070	1.1487420
H	6.2335030	-1.6875760	1.4332410
H	5.4692370	-5.1099390	-1.6394480
H	6.0918890	-7.4463740	-1.9914420
C	8.3910140	-8.7711450	-1.4045010
H	9.9821090	-6.7570480	-0.2894060
H	8.2390190	-0.7551160	0.6236580
H	10.4468250	-0.5666740	1.6469740

C	12.4263820	-2.5046680	1.9589450
H	11.2782010	-4.7055210	0.8211550
H	0.3189780	-3.3891200	-1.1239410
H	1.8985310	-2.7811860	-1.6015200
H	-0.3569760	3.5128230	-1.3416810
H	-1.9274110	2.9062960	-1.8383790
H	-2.5492200	2.5956090	0.5862160
H	-1.0247310	3.3314770	1.0508870
H	-1.6563840	5.4657130	0.0495420
H	-2.9890950	4.7310480	-0.8284480
H	-4.2340920	4.3045990	1.2213250
H	-2.9114240	4.8798750	2.2245900
H	-3.1547680	7.1639140	1.3830300
H	-4.2801130	6.6513400	0.1387080
H	-5.9578650	6.0141550	1.8276310
H	-4.8509490	6.4195560	3.1353080
H	-4.8317630	8.7998890	2.4153620
H	-5.8591320	8.4114750	1.0414800
H	-7.7000630	7.7364070	2.5395490
H	-6.6905230	7.9870310	3.9565910
H	-6.4896400	10.4173960	3.3980670
H	-7.4932080	10.1820920	1.9787570
H	-8.8560820	11.0787210	3.8768540
H	-9.4116450	9.4529280	3.4509680
H	-8.3971260	9.6920470	4.8783230
H	2.2879670	-2.1510680	0.8583590
H	0.8539650	-3.0811910	1.2532190
H	1.9057190	-5.1507340	0.4132320
H	3.1872270	-4.2265840	-0.3596840
H	4.0249950	-3.4563780	1.8237940
H	2.8161100	-4.4072270	2.6709950
H	3.8411840	-6.4900040	2.0112130
H	4.6783040	-5.7448830	0.6624500
H	6.1425650	-4.4791100	2.1951050
H	5.3679090	-5.2143770	3.5920110
H	6.1467300	-7.4620690	2.8737010
H	6.7703620	-6.7900690	1.3712110
H	8.3951530	-5.3963880	2.6274500
H	7.8023720	-6.0374510	4.1549080
H	8.5183000	-8.3285370	3.4955650
H	9.0025890	-7.7344540	1.9144470
H	11.0016330	-7.9640520	3.4086380
H	10.7383890	-6.2634610	2.9791800
H	10.2681180	-6.8526040	4.5762230
C	-10.1067620	-10.4771940	-1.5908260
C	-8.5839960	-10.1643830	-3.5277330
C	-7.6999770	-11.0403410	-1.3491900
C	-12.9527880	-2.8371550	2.7500290
C	-13.7352620	-4.2682400	0.8786940
C	-12.8874940	-5.3037460	3.0019530

C	-13.8065030	2.5268770	0.7983460
C	-13.2206000	0.2383500	0.0417390
C	-12.7575710	0.9500440	2.4082600
C	-8.1476960	9.5833770	-1.3204500
C	-10.2273480	8.5819240	-2.2461660
C	-10.0012180	8.9867200	0.2172130
C	9.8169210	-9.1397300	-0.9868060
C	7.4129100	-9.6144000	-0.5700470
C	8.2261580	-9.1159870	-2.8928740
C	12.6961200	-1.1091610	2.5259460
C	12.5545850	-3.5188930	3.1063980
C	13.4937450	-2.8008000	0.8933710
C	13.8236190	4.5050290	0.8568300
C	12.8690330	2.8034100	2.3933340
C	13.4295250	2.2015850	0.0193180
C	7.9493830	11.0362710	-2.0607520
C	9.5883850	10.6813560	-0.2290220
C	10.1983300	10.1305630	-2.5988600
H	8.3400980	12.0515540	-2.1923000
H	7.5543820	10.7064230	-3.0284520
H	7.1180220	11.0914220	-1.3486250
H	9.9418290	11.7112360	-0.3625390
H	8.7973900	10.6857920	0.5291890
H	10.4228540	10.0902380	0.1627210
H	10.5573420	11.1545250	-2.7601090
H	11.0518910	9.5246350	-2.2772010
H	9.8500950	9.7338880	-3.5590120
H	14.8320560	4.2038290	1.1619200
H	13.8892220	4.8989670	-0.1638790
H	13.5059930	5.3169410	1.5210610
H	14.4435890	1.9174060	0.3261210
H	12.8090950	1.2994910	0.0428690
H	13.4694620	2.5478550	-1.0194820
H	13.8812370	2.5227820	2.7097310
H	12.5021250	3.5842390	3.0688170
H	12.2264880	1.9261070	2.5189060
H	13.5415390	-3.4387850	3.5781200
H	11.7921460	-3.3404750	3.8727380
H	12.4389190	-4.5492740	2.7546390
H	14.4983530	-2.7326040	1.3285110
H	13.3783270	-3.8056330	0.4733940
H	13.4285070	-2.0833280	0.0676330
H	13.6929730	-1.0803510	2.9797640
H	12.6731370	-0.3425270	1.7439560
H	11.9712130	-0.8354990	3.3013890
H	9.9926850	-10.2042140	-1.1778900
H	10.5645450	-8.5724730	-1.5531350
H	9.9872310	-8.9657050	0.0819120
H	8.4354400	-10.1790780	-3.0629330
H	7.2097520	-8.9172510	-3.2484410

H	8.9170520	-8.5283050	-3.5076480
H	7.6105230	-10.6837440	-0.7138300
H	7.5152140	-9.3861100	0.4968820
H	6.3717840	-9.4254280	-0.8524650
H	-8.8165190	-11.1839700	-3.8589490
H	-9.2855230	-9.4773760	-4.0139090
H	-7.5772650	-9.9204930	-3.8829120
H	-7.9220910	-12.0693400	-1.6576740
H	-6.6658880	-10.8224390	-1.6362700
H	-7.7615820	-10.9890440	-0.2564200
H	-10.2993290	-11.5035550	-1.9228020
H	-10.2404470	-10.4500890	-0.5032830
H	-10.8663920	-9.8332880	-2.0487240
H	-14.7523710	-4.2336230	1.2884410
H	-13.6267400	-3.4461110	0.1620700
H	-13.6268650	-5.2085020	0.3275790
H	-13.8921510	-5.2658640	3.4405060
H	-12.7715490	-6.2815370	2.5232510
H	-12.1559130	-5.2406370	3.8151510
H	-13.9598390	-2.8481430	3.1821210
H	-12.2417180	-2.6871730	3.5707320
H	-12.8953550	-1.9736280	2.0803330
H	-14.2233490	-0.1010520	0.3290070
H	-13.2519180	0.5474390	-1.0090000
H	-12.5481490	-0.6228950	0.1133400
H	-13.7442150	0.5787670	2.7121980
H	-12.0306680	0.1479010	2.5727750
H	-12.4853960	1.7827640	3.0664780
H	-14.8006450	2.1508390	1.0648440
H	-13.5852780	3.3691850	1.4636180
H	-13.8556660	2.9023690	-0.2302880
H	-10.6424220	9.5879400	-2.3849580
H	-9.7175760	8.2903320	-3.1712610
H	-11.0632740	7.8907380	-2.0936410
H	-10.3829860	10.0094300	0.1112240
H	-10.8555400	8.3358290	0.4297980
H	-9.3350190	8.9587260	1.0854340
H	-8.5854920	10.5835690	-1.4130690
H	-7.4199990	9.6137790	-0.5009870
H	-7.6100170	9.3729430	-2.2519180

8. **2h**

C	14.1523730	-2.9272860	-1.1998620
N	13.1384340	-2.4766070	-0.3667380
C	13.6827280	-1.5910860	0.5573130
C	15.0716510	-1.4817910	0.3252480
C	15.3716390	-2.3310450	-0.8018490
C	15.8390130	-0.6434060	1.1356310
C	15.2179880	0.0696120	2.1512500
C	13.8391460	-0.0567380	2.3713340

C	13.0544320	-0.8940590	1.5879850
C	14.0759470	-3.7839240	-2.2961160
C	15.2503850	-4.0433130	-2.9923430
C	16.4699730	-3.4650600	-2.6103960
C	16.5360940	-2.6085980	-1.5188510
C	11.7881130	-2.8857380	-0.3937700
C	10.7849550	-1.9122590	-0.5417240
C	9.4403290	-2.2440760	-0.4807710
C	9.1117630	-3.5865180	-0.2913650
C	10.1084770	-4.5850160	-0.2160100
C	11.4561730	-4.2283360	-0.2481170
C	9.4217440	-5.8460680	-0.0761960
C	8.0378590	-5.5588350	-0.0573780
N	7.8598180	-4.1853640	-0.1934780
C	7.0899530	-6.5650020	0.1218970
C	7.5450530	-7.8697490	0.2402640
C	8.9183210	-8.1712720	0.2153400
C	9.8622020	-7.1598340	0.0704880
C	6.6366480	-3.4936260	-0.0970940
C	6.5134790	-2.4331710	0.8058750
C	5.3285970	-1.7230300	0.8893150
C	4.2321730	-2.0615920	0.0818070
C	4.3493140	-3.1560920	-0.7856740
C	5.5431720	-3.8577640	-0.8861540
C	3.0058580	-1.2786230	0.1246650
O	1.8494310	-1.9394850	-0.1665270
C	0.8360000	-1.0268540	-0.0978410
C	1.3511750	0.2446290	0.2520840
C	2.7773280	0.0485960	0.3832600
C	-0.4868390	-1.3481830	-0.3613430
C	-1.3513300	-0.2444750	-0.2520320
C	-0.8361520	1.0270090	0.0978940
C	0.4866840	1.3483350	0.3614080
C	-2.7774850	-0.0484360	-0.3832020
C	-3.0060080	1.2787850	-0.1246050
O	-1.8495800	1.9396430	0.1665850
C	-3.8524070	-1.0384080	-0.5747970
C	-4.7947980	-1.2015560	0.4504700
C	-5.8673380	-2.0648490	0.3019200
C	-6.0206920	-2.7849360	-0.8844490
C	-5.0845110	-2.6489440	-1.9080280
C	-4.0083110	-1.7791220	-1.7507990
C	-4.2323170	2.0617640	-0.0817640
C	-5.3287360	1.7232080	-0.8892800
C	-6.5136200	2.4333460	-0.8058420
C	-6.6367970	3.4937950	0.0971330
C	-5.5433200	3.8579370	0.8861910
C	-4.3494610	3.1562680	0.7857120
N	-7.8599740	4.1855170	0.1935350
C	-8.0380510	5.5589760	0.0573280

C	-9.4219490	5.8461590	0.0759640
C	-10.1086540	4.5850860	0.2157520
C	-9.1119140	3.5866330	0.2912800
C	-9.8624420	7.1598990	-0.0708400
C	-8.9185830	8.1713670	-0.2156310
C	-7.5453020	7.8698950	-0.2403760
C	-7.0901650	6.5651710	-0.1218850
C	-9.4404500	2.2441870	0.4807250
C	-10.7850670	1.9123200	0.5415240
C	-11.7882460	2.8857550	0.3933860
C	-11.4563430	4.2283570	0.2477120
N	-9.3376770	9.5138430	-0.3399830
C	-9.0071280	10.3740930	-1.3803410
C	-9.6082700	11.6318300	-1.1447850
C	-10.3363850	11.5187260	0.0960290
C	-10.1451120	10.1976310	0.5617020
C	-9.4253850	12.6647090	-2.0658230
C	-8.6589010	12.4301770	-3.1991790
C	-8.0817740	11.1716870	-3.4232820
C	-8.2478310	10.1266310	-2.5230310
C	-10.6966080	9.7568600	1.7637230
C	-11.4625470	10.6606570	2.4893600
C	-11.6735850	11.9712770	2.0367020
C	-11.1111180	12.4064650	0.8446020
N	-13.1385410	2.4765520	0.3661840
C	-13.6826390	1.5909340	-0.5578900
C	-15.0715770	1.4815040	-0.3259750
C	-15.3717780	2.3307770	0.8010520
C	-14.1526170	2.9271540	1.1991800
C	-15.8387550	0.6429820	-1.1363880
C	-15.2175370	-0.0700400	-2.1518890
C	-13.8386870	0.0564520	-2.3718300
C	-13.0541530	0.8939170	-1.5884510
C	-14.0763940	3.7838180	2.2954270
C	-15.2509300	4.0430900	2.9915320
C	-16.4704170	3.4646980	2.6094720
C	-16.5363350	2.6082120	1.5179330
N	-7.1376050	-3.6412490	-1.0268440
C	-7.0942130	-5.0290460	-0.9956170
C	-8.4057380	-5.5288680	-0.8246820
C	-9.2782960	-4.3821250	-0.7322810
C	-8.4557070	-3.2398930	-0.8538310
C	-8.6187990	-6.9047830	-0.7566440
C	-7.5198610	-7.7565330	-0.8179190
C	-6.2216680	-7.2400820	-0.9747990
C	-5.9927020	-5.8766040	-1.0762290
C	-8.9827060	-1.9499590	-0.8215670
C	-10.3503970	-1.8190790	-0.6397110
C	-11.1764740	-2.9434290	-0.4559830
C	-10.6471060	-4.2290360	-0.5120590

N	-7.6992540	-9.1518600	-0.6976280
C	-8.3837030	-9.7923920	0.3296690
C	-8.3481370	-11.1878920	0.1051100
C	-7.5891790	-11.3950470	-1.1044980
C	-7.2081710	-10.1156290	-1.5709890
C	-8.9765180	-12.0477760	1.0070040
C	-9.6114500	-11.5121220	2.1195780
C	-9.6110910	-10.1275680	2.3429890
C	-8.9995210	-9.2491020	1.4568810
C	-6.4781690	-9.9546450	-2.7480660
C	-6.1109830	-11.1021190	-3.4400960
C	-6.4637780	-12.3794250	-2.9812850
C	-7.2065130	-12.5318460	-1.8185340
N	-12.5480610	-2.7477800	-0.1917590
C	-13.0601940	-1.9505610	0.8265650
C	-14.4711130	-1.9953030	0.7796090
C	-14.8223320	-2.8642560	-0.3169780
C	-13.6095650	-3.3012660	-0.8972660
C	-15.2133490	-1.2495840	1.6959620
C	-14.5487150	-0.4785790	2.6395560
C	-13.1480680	-0.4683140	2.6899680
C	-12.3846000	-1.2109970	1.7963900
C	-13.5889580	-4.1170480	-2.0270330
C	-14.8112000	-4.5069670	-2.5615310
C	-16.0243420	-4.0956960	-1.9900870
C	-16.0355190	-3.2728090	-0.8714550
C	3.8522460	1.0385730	0.5748770
C	4.0081710	1.7792350	1.7509050
C	5.0843840	2.6490420	1.9081510
C	6.0205420	2.7850680	0.8845600
C	5.8671590	2.0650430	-0.3018440
C	4.7946130	1.2017610	-0.4504090
N	7.1374930	3.6413340	1.0269900
C	7.0941760	5.0291330	0.9958420
C	8.4057270	5.5288930	0.8249040
C	9.2782200	4.3821050	0.7324140
C	8.4555680	3.2399120	0.8539230
C	8.6188760	6.9047960	0.7570130
C	7.5199980	7.7566250	0.8184150
C	6.2217820	7.2402340	0.9752830
C	5.9927300	5.8767560	1.0765840
C	8.9824910	1.9499510	0.8215810
C	10.3501730	1.8190040	0.6396930
C	11.1763070	2.9433130	0.4560030
C	10.6470130	4.2289510	0.5121520
N	7.6994920	9.1519480	0.6982990
C	7.2084970	10.1156420	1.5718020
C	7.5898180	11.3950760	1.1056200
C	8.3489130	11.1880150	-0.1039200
C	8.3842270	9.7925590	-0.3287630

C	7.2073210	12.5318110	1.8198480
C	6.4644400	12.3793080	2.9824950
C	6.1113410	11.1019790	3.4410090
C	6.4783550	9.9545700	2.7487800
C	9.0001030	9.2493850	-1.4560010
C	9.6119920	10.1279170	-2.3418250
C	9.6126060	11.5124240	-2.1181180
C	8.9776080	12.0479680	-1.0055300
N	12.5478780	2.7476000	0.1917380
C	13.0599390	1.9504980	-0.8267120
C	14.4708620	1.9952180	-0.7798360
C	14.8221570	2.8640600	0.3168140
C	13.6094280	3.3010220	0.8972220
C	15.2130300	1.2495720	-1.6963030
C	14.5483270	0.4786710	-2.6399340
C	13.1476760	0.4684350	-2.6902660
C	12.3842740	1.2110400	-1.7965680
C	13.5888940	4.1167120	2.0270570
C	14.8111700	4.5065890	2.5615050
C	16.0242740	4.0953650	1.9899480
C	16.0353790	3.2725690	0.8712470
N	9.3373770	-9.5137720	0.3395680
C	10.1446580	-10.1975480	-0.5622610
C	10.3359350	-11.5186770	-0.0966830
C	9.6079820	-11.6318130	1.1442230
C	9.0069280	-10.3740620	1.3799260
C	11.1105300	-12.4064090	-0.8454070
C	11.6728580	-11.9711830	-2.0375580
C	11.4618190	-10.6605290	-2.4901180
C	10.6960160	-9.7567370	-1.7643300
C	8.2477940	-10.1266250	2.5227290
C	8.0818090	-11.1717210	3.4229470
C	8.6588500	-12.4302260	3.1987000
C	9.4251720	-12.6647320	2.0652310
H	-3.5043980	3.4333690	1.4082840
H	-5.6436010	4.6733450	1.5961950
H	-8.6740380	1.4838570	0.5949020
H	-11.0935650	0.8877650	0.7211880
H	-13.1305350	4.2231060	2.5981520
H	-15.2208020	4.7066240	3.8516220
H	-17.3701710	3.6896340	3.1750710
H	-17.4801050	2.1537640	1.2271090
H	-16.9077410	0.5433910	-0.9654520
H	-15.7959600	-0.7502570	-2.7692800
H	-13.3717720	-0.5165840	-3.1679790
H	-11.9907150	1.0074820	-1.7761520
H	-12.2410470	4.9717820	0.1408510
H	-10.9210980	7.4020860	-0.0851830
H	-10.5281470	8.7458110	2.1214350
H	-11.9053730	10.3425620	3.4293390

H	-12.2805480	12.6510010	2.6278260
H	-11.2672850	13.4246550	0.4976670
H	-9.8815850	13.6367680	-1.8971620
H	-8.5075260	13.2255320	-3.9233090
H	-7.4942470	11.0061240	-4.3223040
H	-7.8098320	9.1502450	-2.7057690
H	-6.8361230	8.6845220	-0.3512490
H	-6.0290290	6.3428280	-0.1633860
H	-7.3493280	2.1850970	-1.4531560
H	-5.2420500	0.9103620	-1.6022340
H	-3.3041150	-1.6423970	-2.5665250
H	-5.2217730	-3.2017130	-2.8328230
H	-8.3470660	-1.0785170	-0.9486740
H	-10.8123130	-0.8373640	-0.6471840
H	-12.6516340	-4.4278910	-2.4783750
H	-14.8239630	-5.1435340	-3.4421450
H	-16.9619790	-4.4224250	-2.4307280
H	-16.9748600	-2.9402190	-0.4372140
H	-16.2993770	-1.2586810	1.6572540
H	-15.1150420	0.1343090	3.3340990
H	-12.6458210	0.1355960	3.4407540
H	-11.3001810	-1.2201440	1.8554050
H	-11.2953710	-5.0876960	-0.3630970
H	-9.6180680	-7.3160190	-0.6480330
H	-6.2126200	-8.9681520	-3.1146520
H	-5.5391400	-11.0039690	-4.3588530
H	-6.1560600	-13.2553840	-3.5449370
H	-7.4891810	-13.5205990	-1.4667040
H	-8.9577170	-13.1221010	0.8438100
H	-10.1044690	-12.1698100	2.8295390
H	-10.0990870	-9.7291750	3.2282360
H	-8.9956420	-8.1791710	1.6393120
H	-5.3870370	-7.9321840	-0.9974970
H	-4.9852790	-5.4864610	-1.1850050
H	-6.5945700	-2.1924460	1.0984800
H	-4.6749030	-0.6363260	1.3699400
C	-0.8912370	-2.7493770	-0.7171930
H	4.6746940	0.6365640	-1.3698950
H	6.5943840	2.1926710	-1.0984050
H	8.3468040	1.0785360	0.9486490
H	10.8120280	0.8372580	0.6470960
H	12.6515960	4.4275230	2.4784770
H	14.8239930	5.1430870	3.4421690
H	16.9619400	4.4220570	2.4305530
H	16.9746920	2.9400170	0.4369180
H	16.2990600	1.2586440	-1.6576560
H	15.1146010	-0.1341560	-3.3345740
H	12.6453770	-0.1353920	-3.4410840
H	11.2998510	1.2202130	-1.8555140
H	11.2953300	5.0875770	0.3632190

H	9.6181710	7.3159740	0.6484170
H	8.9960370	8.1794960	-1.6386700
H	10.1000420	9.7296120	-3.2270810
H	10.1058660	12.1701580	-2.8278680
H	8.9589970	13.1222600	-0.8421010
H	7.4902350	13.5205740	1.4682440
H	6.1568480	13.2552120	3.5463000
H	5.5393960	11.0037570	4.3596940
H	6.2125720	8.9680620	3.1151560
H	5.3871840	7.9323720	0.9980820
H	4.9852820	5.4866750	1.1853490
H	5.2216760	3.2017650	2.8329690
H	3.3039900	1.6424770	2.5666390
H	3.5042480	-3.4331930	-1.4082420
H	5.6434480	-4.6731800	-1.5961490
H	6.0288320	-6.3426160	0.1635310
H	6.8358590	-8.6843560	0.3511800
H	7.8098630	-9.1502290	2.7055750
H	7.4944090	-11.0061790	4.3220560
H	8.5075350	-13.2256110	3.9228090
H	9.8813060	-13.6368030	1.8964580
H	11.2666980	-13.4246250	-0.4985470
H	12.2797120	-12.6509010	-2.6287990
H	11.9045360	-10.3424030	-3.4301370
H	10.5275540	-8.7456600	-2.1219640
H	10.9208500	-7.4020650	0.0846900
H	12.2408630	-4.9717950	-0.1413940
H	13.1300110	-4.2231030	-2.5987550
H	15.2200990	-4.7068290	-3.8524400
H	17.3696470	-3.6900870	-3.1760870
H	17.4799420	-2.1542560	-1.2281150
H	16.9079920	-0.5439270	0.9645840
H	15.7965530	0.7497160	2.7686330
H	13.3723860	0.5163040	3.1675690
H	11.9910010	-1.0075110	1.7757880
H	11.0934670	-0.8877050	-0.7213740
H	8.6739360	-1.4837070	-0.5948110
H	7.3491930	-2.1849270	1.4531840
H	5.2419100	-0.9101800	1.6022650
C	0.8910870	2.7495210	0.7172750
C	-1.6054020	-3.5084140	0.4055720
H	-1.5421610	-2.7327260	-1.5990770
H	0.0060680	-3.3079180	-1.0071170
C	-1.9400700	-4.9359940	-0.0110290
H	-0.9682200	-3.5193720	1.2998230
H	-2.5242410	-2.9773190	0.6832720
C	-2.7744800	-5.7013100	1.0102610
H	-2.4802360	-4.9134170	-0.9705920
H	-1.0061000	-5.4838050	-0.2058380
C	-2.9429930	-7.1735000	0.6474640

H	-2.3077160	-5.6237240	2.0028470
H	-3.7625730	-5.2261120	1.1004300
C	-3.8815820	-7.9345690	1.5767760
H	-3.3088030	-7.2521400	-0.3877580
H	-1.9560360	-7.6590570	0.6467590
C	-3.9997190	-9.4168480	1.2371750
H	-3.5282410	-7.8290610	2.6131460
H	-4.8782030	-7.4711600	1.5462100
C	-4.9230790	-10.1825540	2.1777920
H	-4.3586550	-9.5377060	0.2036690
H	-2.9989070	-9.8735290	1.2592030
C	-5.0216230	-11.6682520	1.8476320
H	-4.5650620	-10.0620160	3.2115970
H	-5.9294380	-9.7395730	2.1490930
C	-5.8643200	-12.4396210	2.8551470
H	-5.4475120	-11.7857510	0.8419570
H	-4.0101010	-12.0975990	1.8059290
H	-5.9460010	-13.4984370	2.5840110
H	-5.4252470	-12.3854280	3.8591190
H	-6.8792140	-12.0309480	2.9097500
C	1.6052480	3.5085640	-0.4054840
H	-0.0062120	3.3080610	1.0072200
H	1.5420240	2.7328470	1.5991500
C	1.9401080	4.9360750	0.0111950
H	2.5240130	2.9773920	-0.6832840
H	0.9680060	3.5196530	-1.2996900
C	2.7744910	5.7013670	-1.0101360
H	1.0062200	5.4839750	0.2061500
H	2.4803840	4.9133620	0.9706920
C	2.9431920	7.1735230	-0.6472920
H	3.7625270	5.2260710	-1.1004300
H	2.3076130	5.6238760	-2.0026750
C	3.8819970	7.9344540	-1.5764990
H	1.9563130	7.6592380	-0.6466690
H	3.3089230	7.2520890	0.3879650
C	4.0002260	9.4167390	-1.2369580
H	4.8785670	7.4709550	-1.5457300
H	3.5288270	7.8289140	-2.6129240
C	4.9238680	10.1822900	-2.1774180
H	2.9994710	9.8735340	-1.2592060
H	4.3589660	9.5376050	-0.2033840
C	5.0227120	11.6679440	-1.8471590
H	5.9301250	9.7390810	-2.1486280
H	4.5659480	10.0618930	-3.2112730
C	5.8657570	12.4391630	-2.8544950
H	4.0112850	12.0975290	-1.8055980
H	5.4484640	11.7852880	-0.8414070
H	5.9477410	13.4979230	-2.5832300
H	6.8805320	12.0301870	-2.9090320
H	5.4267790	12.3852170	-3.8585210

9. **2i**

C	12.6430690	-6.7583590	3.4327460
C	11.4581160	-6.6244750	2.6950270
C	11.3046320	-7.3814080	1.5251320
C	12.3158070	-8.2348350	1.1019830
C	13.4916680	-8.3660560	1.8370880
C	13.6424170	-7.6252340	3.0067000
N	10.4445150	-5.7346910	3.0976970
C	10.1647050	-5.4864240	4.4535980
C	9.7335490	-4.2153790	4.8589330
C	9.4271010	-3.9698570	6.1920800
C	9.5547520	-4.9746150	7.1483950
C	9.9864730	-6.2373720	6.7484720
C	10.2816900	-6.4990380	5.4156920
C	9.6745860	-5.0712950	2.1061430
C	8.2701820	-5.1242220	2.1709340
C	7.4758920	-4.5014940	1.2196970
C	8.1143870	-3.8285580	0.1808190
C	9.5226160	-3.7596210	0.1047980
C	10.3050430	-4.3798870	1.0753680
C	9.8363710	-2.9532160	-1.0519810
C	8.6068310	-2.5930610	-1.6414990
N	7.5602360	-3.1559250	-0.9033460
C	8.5627830	-1.7550590	-2.7537770
C	9.7641310	-1.2877720	-3.2671210
C	10.9990640	-1.6051640	-2.6674050
C	11.0317990	-2.4493920	-1.5583630
C	6.2455210	-2.6422210	-0.9485070
C	5.4873870	-2.7435570	-2.1156600
C	4.2937610	-2.0353540	-2.2316850
C	3.8419350	-1.2299560	-1.1830290
C	4.5535890	-1.2394240	0.0236980
C	5.7468930	-1.9312440	0.1448690
C	2.7818210	-0.2159000	-1.3459600
C	1.3456610	-0.3227790	-1.3748590
C	0.8769640	1.0131030	-1.3660250
O	1.9327390	1.8831150	-1.3445830
C	3.0678400	1.1252540	-1.3371170
C	0.4505640	-1.4062420	-1.3708380
C	-0.8769000	-1.0111920	-1.3657130
C	-1.3456000	0.3246880	-1.3746250
C	-0.4505020	1.4081550	-1.3709120
O	-1.9326600	-1.8812080	-1.3439470
C	-3.0677740	-1.1233550	-1.3363580
C	-2.7817530	0.2178030	-1.3454330
C	-4.3605170	-1.7914960	-1.2428090
C	-5.4805220	-1.2255410	-1.8710300
C	-6.7521310	-1.7186750	-1.6419730

C	-6.9372340	-2.7955890	-0.7713490
C	-5.8229580	-3.4198230	-0.2013240
C	-4.5463220	-2.9273690	-0.4440410
C	-3.8418930	1.2318380	-1.1825820
C	-4.5535410	1.2413840	0.0241560
C	-5.7469800	1.9329750	0.1452100
C	-6.2457510	2.6436740	-0.9482860
C	-5.4875940	2.7450440	-2.1154170
C	-4.2938290	2.0370410	-2.2313340
N	-7.5606340	3.1569780	-0.9032760
C	-8.6069700	2.5936470	-1.6414460
C	-9.8366900	2.9531770	-1.0519130
C	-9.5233160	3.7596770	0.1049200
C	-8.1151140	3.8292960	0.1809120
C	-11.0318690	2.4487850	-1.5583260
C	-10.9987070	1.6046150	-2.6674090
C	-9.7636130	1.2878390	-3.2671100
C	-8.5625030	1.7556960	-2.7537390
C	-7.4768820	4.5024560	1.2198030
C	-8.2714300	5.1246670	2.1711560
C	-9.6758010	5.0710210	2.1064320
C	-10.3060070	4.3794550	1.0756070
N	-12.1986410	1.0650850	-3.1854660
C	-12.2913240	-0.2982090	-3.5269580
C	-11.4422960	-1.2571170	-2.9538820
C	-11.5678020	-2.6008480	-3.2907050
C	-12.5462120	-3.0224100	-4.1872250
C	-13.3883550	-2.0719190	-4.7617210
C	-13.2579850	-0.7242370	-4.4497580
N	-10.4459660	5.7338110	3.0982400
C	-11.4590390	6.6243270	2.6959770
C	-11.3054420	7.3812840	1.5261090
C	-12.3161750	8.2354630	1.1034080
C	-13.4916990	8.3674270	1.8389110
C	-13.6425650	7.6265580	3.0084800
C	-12.6436770	6.7589480	3.4340920
N	-8.2426770	-3.2184140	-0.4638890
C	-9.2928890	-2.3760290	-0.0932790
C	-10.4650490	-3.1450830	0.0631220
C	-10.1145640	-4.5075930	-0.2563630
C	-8.7383330	-4.5133300	-0.5770280
C	-11.6636300	-2.5436800	0.4380130
C	-11.7129670	-1.1589670	0.5950290
C	-10.5261350	-0.4065140	0.4529450
C	-9.3134340	-0.9993530	0.1328160
C	-8.1033480	-5.6793190	-1.0034860
C	-8.8661320	-6.8324020	-1.1132050
C	-10.2369870	-6.8455100	-0.7891470
C	-10.8588630	-5.6810510	-0.3455660
N	-12.9304430	-0.5004950	0.8728940

C	-14.1682190	-0.9960490	0.4108930
C	-14.2745540	-1.6828460	-0.8076800
C	-15.5097040	-2.1380530	-1.2540790
C	-16.6656290	-1.9121330	-0.5104960
C	-16.5628390	-1.2339640	0.7017770
C	-15.3306170	-0.7901160	1.1672990
N	-10.9901200	-8.0336530	-0.9551350
C	-12.2396380	-7.9653390	-1.6053750
C	-12.4170460	-7.1162120	-2.7061890
C	-13.6552840	-7.0261540	-3.3293890
C	-14.7324870	-7.7889950	-2.8837740
C	-14.5555850	-8.6408890	-1.7961740
C	-13.3261640	-8.7248080	-1.1532080
C	4.3606460	1.7933130	-1.2438530
C	5.4804230	1.2272390	-1.8723730
C	6.7521620	1.7201170	-1.6434960
C	6.9376190	2.7968890	-0.7727740
C	5.8235670	3.4212810	-0.2024890
C	4.5467950	2.9290760	-0.4450140
N	8.2432220	3.2193060	-0.4654170
C	9.2930990	2.3765710	-0.0946850
C	10.4654990	3.1452230	0.0619000
C	10.1155010	4.5078640	-0.2576200
C	8.7392730	4.5140690	-0.5783530
C	11.6637290	2.5434200	0.4373230
C	11.7124200	1.1587340	0.5947420
C	10.5253970	0.4066770	0.4521870
C	9.3130460	0.9999030	0.1315070
C	8.1045640	5.6803100	-1.0045330
C	8.8676220	6.8332440	-1.1138740
C	10.2384740	6.8458790	-0.7898310
C	10.8601080	5.6811530	-0.3466300
N	12.9293810	0.4997510	0.8737360
C	14.1677610	0.9944310	0.4124920
C	15.3295150	0.7881380	1.1698020
C	16.5623110	1.2311350	0.7049890
C	16.6663490	1.9087790	-0.5074700
C	15.5110790	2.1350490	-1.2519580
C	14.2753510	1.6807210	-0.8062620
N	10.9919000	8.0339170	-0.9554110
C	12.2405890	7.9656590	-1.6072400
C	13.3281710	8.7237930	-1.1553800
C	14.5567260	8.6399690	-1.8000110
C	14.7316700	7.7895150	-2.8890570
C	13.6534060	7.0280040	-3.3343840
C	12.4160360	7.1179260	-2.7094340
N	12.1993140	-1.0663230	-3.1854440
C	12.2931790	0.2969990	-3.5264050
C	11.4440910	1.2563280	-2.9541160
C	11.5710370	2.6000910	-3.2902900

C	12.5509240	3.0212940	-4.1853540
C	13.3930470	2.0703740	-4.7591690
C	13.2612720	0.7226780	-4.4478860
C	-10.1660610	5.4848240	4.4539970
C	-9.7351300	4.2134520	4.8585520
C	-9.4285320	3.9671020	6.1915090
C	-9.5557900	4.9713600	7.1484060
C	-9.9872510	6.2344470	6.7492550
C	-10.2826260	6.4969330	5.4166670
C	-13.3648970	1.8687950	-3.1251620
C	-13.3314810	3.1756640	-3.6233360
C	-14.4516000	3.9922450	-3.5204180
C	-15.6240780	3.5147250	-2.9374790
C	-15.6626740	2.2087990	-2.4540880
C	-14.5411320	1.3903070	-2.5385530
C	-10.4972170	-9.2498390	-0.4460790
C	-10.7128380	-10.4532320	-1.1316720
C	-10.2173180	-11.6473610	-0.6231970
C	-9.4838410	-11.6667340	0.5613190
C	-9.2589780	-10.4704080	1.2381190
C	-9.7653740	-9.2723840	0.7485670
C	-12.9129250	0.6841070	1.6499730
C	-13.5884950	1.8290050	1.2098490
C	-13.5772640	2.9848250	1.9841470
C	-12.8777670	3.0207300	3.1894080
C	-12.1964580	1.8856940	3.6213440
C	-12.2191500	0.7202380	2.8634320
C	10.4999410	9.2497850	-0.4447650
C	9.7684830	9.2713520	0.7501450
C	9.2629750	10.4690920	1.2412960
C	9.4883280	11.6661020	0.5658720
C	10.2214040	11.6476930	-0.6189060
C	10.7160490	10.4538860	-1.1289790
C	12.9105730	-0.6840910	1.6519510
C	12.2157930	-0.7185870	2.8648800
C	12.1916100	-1.8833270	3.6238330
C	12.8724500	-3.0192520	3.1935120
C	13.5730380	-2.9849600	1.9888430
C	13.5857240	-1.8298670	1.2134700
C	13.3650810	-1.8707810	-3.1245490
C	13.3313720	-3.1771450	-3.6239750
C	14.4508420	-3.9945570	-3.5205130
C	15.6229260	-3.5183170	-2.9357380
C	15.6618070	-2.2128810	-2.4510420
C	14.5408870	-1.3935870	-2.5360540
H	-3.7453610	2.0529060	-3.1695110
H	-5.8654340	3.3372450	-2.9437090
H	-6.3943920	4.5514390	1.2800660
H	-7.8093090	5.6640440	2.9917870
H	-10.6013210	7.4889390	5.1122610

H	-10.0824520	7.0343480	7.4791170
H	-9.3199580	4.7736020	8.1899200
H	-9.0951000	2.9745290	6.4839480
H	-9.6453600	3.4252790	4.1175570
H	-12.7740090	6.1878490	4.3482800
H	-14.5532530	7.7128770	3.5955750
H	-14.2778020	9.0391260	1.5067230
H	-12.1753070	8.8117050	0.1926660
H	-10.3903340	7.2882000	0.9494420
H	-11.3888780	4.3216050	1.0440220
H	-11.9761040	2.6804080	-1.0756860
H	-14.5726290	0.3796640	-2.1447880
H	-16.5646610	1.8186870	-1.9895480
H	-16.4985490	4.1548150	-2.8616410
H	-14.4093280	5.0068180	-3.9078260
H	-12.4164370	3.5451730	-4.0772150
H	-13.9154950	0.0071930	-4.9092020
H	-14.1520790	-2.3776710	-5.4724400
H	-12.6501520	-4.0752540	-4.4336870
H	-10.9113210	-3.3249270	-2.8151330
H	-10.7075600	-0.9562460	-2.2148840
H	-9.7584410	0.6417610	-4.1395090
H	-7.6199200	1.4785140	-3.2153020
H	-6.3331730	1.8731690	1.0575900
H	-4.1999600	0.6259550	0.8463240
H	-3.6874040	-3.3882020	0.0350880
H	-5.9684290	-4.2594280	0.4719300
H	-7.0542200	-5.6819220	-1.2815750
H	-8.4076070	-7.7508140	-1.4671560
H	-9.5991480	-8.3441410	1.2869240
H	-8.6944120	-10.4674250	2.1670040
H	-9.0922510	-12.6019140	0.9509810
H	-10.3949750	-12.5702800	-1.1692780
H	-11.2704170	-10.4422770	-2.0633540
H	-13.1982360	-9.3774840	-0.2948630
H	-15.3875780	-9.2365100	-1.4295210
H	-15.6977250	-7.7191680	-3.3768420
H	-13.7751730	-6.3607380	-4.1807550
H	-11.5777180	-6.5258240	-3.0618650
H	-11.9194710	-5.6881030	-0.1117550
H	-12.5590830	-3.1428270	0.5671360
H	-11.6986450	-0.1718360	3.2000820
H	-11.6552670	1.9068470	4.5633360
H	-12.8545960	3.9291530	3.7821080
H	-14.1025550	3.8707590	1.6354800
H	-14.1232920	1.8006330	0.2638760
H	-15.2636980	-0.2728380	2.1192450
H	-17.4497610	-1.0570120	1.3048960
H	-17.6297600	-2.2633240	-0.8667360
H	-15.5570600	-2.6590010	-2.2067200

H	-13.3929100	-1.8472240	-1.4170720
H	-10.5639660	0.6696480	0.5800950
H	-8.4219820	-0.3889050	0.0279030
H	-7.6122220	-1.2671590	-2.1278680
H	-5.3513020	-0.3823450	-2.5398090
C	0.8905680	-2.8405330	-1.3410760
H	3.6880540	3.3900060	0.0343380
H	5.9693140	4.2607880	0.4708290
H	7.0554360	5.6832240	-1.2826190
H	8.4093530	7.7519090	-1.4675040
H	11.2732890	10.4437480	-2.0608720
H	10.3994350	12.5711550	-1.1639490
H	9.0974290	12.6010490	0.9567850
H	8.6987060	10.4653210	2.1703610
H	9.6018680	8.3425780	1.2874620
H	11.5758450	6.5286150	-3.0648640
H	13.7717490	6.3638700	-4.1869690
H	15.6962170	7.7197960	-3.3834910
H	15.3895770	9.2345260	-1.4335820
H	13.2017220	9.3753760	-0.2959840
H	11.9207240	5.6879450	-0.1128380
H	12.5593680	3.1422320	0.5667150
H	14.1213210	-1.8027370	0.2679090
H	14.0979700	-3.8716220	1.6414770
H	12.8480200	-3.9270850	3.7870650
H	11.6496240	-1.9032200	4.5653970
H	11.6956340	0.1741670	3.2002540
H	13.3942440	1.8453660	-1.4163550
H	15.5593890	2.6555820	-2.2047740
H	17.6309310	2.2592800	-0.8631650
H	17.4487000	1.0539190	1.3088140
H	15.2616530	0.2712730	2.1219040
H	10.5628150	-0.6694770	0.5795410
H	8.4213950	0.3897890	0.0263500
H	7.6121000	1.2684910	-2.1295600
H	5.3509030	0.3841400	-2.5412160
H	4.2001210	-0.6238140	0.8457780
H	6.3330860	-1.8714400	1.0572510
H	7.6203340	-1.4774190	-3.2153410
H	9.7593010	-0.6416890	-4.1395200
H	14.5725560	-0.3833000	-2.1413780
H	16.5635160	-1.8238010	-1.9851000
H	16.4969010	-4.1590360	-2.8594830
H	14.4083950	-5.0087400	-3.9089190
H	12.4165950	-3.5455880	-4.0792610
H	13.9188440	-0.0090420	-4.9067750
H	14.1579280	2.3758100	-5.4687810
H	12.6561050	4.0741830	-4.4310700
H	10.9145080	3.3244820	-2.8152640
H	10.7082270	0.9557970	-2.2161060

H	11.9759160	-2.6815160	-1.0757370
H	11.3879330	-4.3225550	1.0436920
H	10.6005900	-7.4907900	5.1106700
H	10.0820000	-7.0376540	7.4778740
H	9.3190300	-4.7774960	8.1900550
H	9.0934820	-2.9775270	6.4851310
H	9.6435170	-3.4267920	4.1184140
H	12.7732960	-6.1872880	4.3469640
H	14.5533620	-7.7110050	3.5934760
H	14.2781310	-9.0371640	1.5045560
H	12.1750420	-8.8110810	0.1912290
H	10.3892520	-7.2888690	0.9488060
H	7.8078120	-5.6634700	2.9915100
H	6.3933820	-4.5499580	1.2800240
H	5.8651390	-3.3359410	-2.9438630
H	3.7452790	-2.0512540	-3.1698550
C	-0.8905300	2.8424400	-1.3411660
C	-1.4344530	3.2832760	0.0258760
H	-0.0578330	3.4890950	-1.6383490
H	-1.6785220	2.9835270	-2.0916370
C	-2.2817430	4.5469860	-0.0669340
H	-2.0497810	2.4776790	0.4443180
H	-0.5990090	3.4251200	0.7245360
C	-2.9245130	4.9461860	1.2566800
H	-1.6757230	5.3795380	-0.4534230
H	-3.0775950	4.3765790	-0.8070950
C	-3.8836960	6.1247880	1.1234010
H	-3.4742960	4.0814610	1.6583690
H	-2.1453560	5.1826830	1.9959330
C	-4.5468110	6.5230850	2.4382400
H	-3.3504380	6.9914760	0.7064870
H	-4.6601220	5.8697200	0.3860430
C	-5.6246730	7.5899240	2.2759220
H	-4.9940950	5.6316860	2.9056270
H	-3.7797980	6.8732070	3.1444010
C	-6.2632940	8.0122070	3.5957160
H	-5.1984510	8.4735460	1.7790690
H	-6.4036490	7.2101130	1.5993430
C	-7.4947670	8.8948060	3.4185300
H	-6.5460830	7.1194330	4.1748270
H	-5.5175260	8.5373650	4.2106190
C	-8.0580340	9.3941750	4.7428260
H	-7.2412420	9.7501700	2.7764660
H	-8.2675890	8.3280790	2.8793290
H	-8.9670220	9.9881050	4.5949090
H	-8.3095410	8.5539790	5.4004350
H	-7.3295320	10.0222740	5.2697590
C	1.4345690	-3.2813730	0.0259290
H	0.0578370	-3.4871780	-1.6381880
H	1.6785120	-2.9816560	-2.0915910

C	2.2815920	-4.5452570	-0.0669240
H	2.0501050	-2.4758660	0.4442400
H	0.5991820	-3.4230150	0.7247010
C	2.9243360	-4.9446170	1.2566550
H	1.6753730	-5.3776750	-0.4533880
H	3.0774450	-4.3750180	-0.8071240
C	3.8831000	-6.1235560	1.1233410
H	3.4744420	-4.0800600	1.6582680
H	2.1451570	-5.1808170	1.9959790
C	4.5461250	-6.5221040	2.4381480
H	3.3495140	-6.9900510	0.7064470
H	4.6595910	-5.8687710	0.3859540
C	5.6235990	-7.5893210	2.2757540
H	4.9937440	-5.6308750	2.9055380
H	3.7790160	-6.8719670	3.1443330
C	6.2620110	-8.0120040	3.5955190
H	5.1970650	-8.4727310	1.7787910
H	6.4027350	-7.2097160	1.5992430
C	7.4930710	-8.8951690	3.4182620
H	6.5452200	-7.1194230	4.1747180
H	5.5159860	-8.5368750	4.2103570
C	8.0559180	-9.3951560	4.7425030
H	7.2391990	-9.7502470	2.7759540
H	8.2662310	-8.3286760	2.8792980
H	8.9646100	-9.9895340	4.5945560
H	8.3077890	-8.5552570	5.4003490
H	7.3270200	-10.0230060	5.2691860

10. **2j**

C	-11.7489490	7.2058600	-2.1979300
C	-11.5351710	7.9997010	-1.0646500
C	-12.6372230	8.4375080	-0.3205410
C	-13.9256560	8.0927200	-0.7110210
C	-14.1367140	7.2942300	-1.8328950
C	-13.0402670	6.8507750	-2.5690680
N	-10.2245450	8.3568380	-0.6778790
C	-9.1851660	7.4097730	-0.7481390
C	-7.8978340	7.7879420	-1.1510150
C	-6.8764020	6.8527150	-1.2098620
C	-7.0960940	5.5059030	-0.8887090
C	-8.3906940	5.1385870	-0.4957530
C	-9.4157470	6.0683260	-0.4168530
C	-6.0089210	4.5137430	-0.9600090
C	-4.6906690	4.8541430	-0.6251770
C	-3.6684020	3.9207890	-0.6869890
C	-3.9251670	2.5993020	-1.0834880
C	-5.2399170	2.2554560	-1.4338590
C	-6.2558790	3.1950910	-1.3693770
C	-2.8386010	1.6340910	-1.1307310

C	-2.7943220	0.2630260	-1.1508460
C	-1.3935660	-0.0979420	-1.1836580
C	-0.6952360	1.1319840	-1.1752790
O	-1.5801290	2.1684320	-1.1500240
C	0.6792690	1.3118230	-1.1832690
C	1.3930350	0.0983800	-1.1836240
C	0.6947080	-1.1315460	-1.1753490
C	-0.6797980	-1.3113870	-1.1833710
C	2.7937920	-0.2625790	-1.1508350
C	2.8380770	-1.6336470	-1.1308400
O	1.5796080	-2.1679930	-1.1501720
C	-3.9459240	-0.6504710	-1.0293730
C	-4.6554010	-0.7203560	0.1755970
C	-5.6848880	-1.6347830	0.3445360
C	-6.0528760	-2.5093010	-0.6877640
C	-5.3738000	-2.4027590	-1.9088260
C	-4.3382330	-1.4909200	-2.0762230
C	3.9454230	0.6508840	-1.0293020
C	4.6548350	0.7207400	0.1757070
C	5.6844320	1.6350400	0.3446620
C	6.0526070	2.5094470	-0.6876650
C	5.3735540	2.4029750	-1.9087440
C	4.3378660	1.4912690	-2.0761540
C	3.9246500	-2.5988510	-1.0836270
C	5.2394130	-2.2549640	-1.4339080
C	6.2553830	-3.1945920	-1.3694390
C	6.0084190	-4.5132750	-0.9601740
C	4.6901550	-4.8537150	-0.6254330
C	3.6678810	-3.9203690	-0.6872320
C	7.0956020	-5.5054250	-0.8888820
C	6.8759520	-6.8522150	-1.2101560
C	7.8973940	-7.7874300	-1.1513160
C	9.1846930	-7.4092720	-0.7483250
C	9.4152320	-6.0678480	-0.4169170
C	8.3901700	-5.1381180	-0.4958130
N	10.2240850	-8.3563250	-0.6780700
C	11.5347270	-7.9991310	-1.0647390
C	11.7485530	-7.2052090	-2.1979530
C	13.0398850	-6.8500660	-2.5689870
C	14.1362990	-7.2935430	-1.8327760
C	13.9251920	-8.0921140	-0.7109680
C	12.6367440	-8.4369610	-0.3205910
C	7.0867890	3.5365840	-0.4655540
C	8.2003590	3.2903600	0.3489090
C	9.1326740	4.2793860	0.6186920
C	8.9697290	5.5718640	0.1018130
C	7.8746540	5.8230140	-0.7350380
C	6.9621510	4.8177450	-1.0184020
N	9.8788630	6.5954870	0.4262210
C	10.1858010	7.5905580	-0.5312610

C	10.2023020	8.9428850	-0.1723010
C	10.5087720	9.9124040	-1.1199350
C	10.7843780	9.5528340	-2.4372970
C	10.7598700	8.2071680	-2.7972730
C	10.4730530	7.2293100	-1.8522080
C	-7.0868460	-3.5366480	-0.4656410
C	-8.2004640	-3.2906770	0.3488310
C	-9.1325380	-4.2799260	0.6186390
C	-8.9692840	-5.5723700	0.1017790
C	-7.8741660	-5.8232690	-0.7350890
C	-6.9619110	-4.8177860	-1.0184790
N	-9.8781540	-6.5962250	0.4262190
C	-10.4192010	-6.6873810	1.7270260
C	-11.7329600	-7.1340780	1.9151740
C	-12.2573910	-7.2466270	3.1972550
C	-11.4955430	-6.8979040	4.3100690
C	-10.1929540	-6.4405460	4.1235900
C	-9.6520420	-6.3437450	2.8471570
C	-9.9607110	9.6664810	-0.2198090
C	-10.5554410	10.7685930	-0.8456470
C	-10.3020250	12.0549390	-0.3847320
C	-9.4413400	12.2662880	0.6900950
C	-8.8421660	11.1708950	1.3080610
C	-9.1040730	9.8793040	0.8669980
C	9.9602470	-9.6660090	-0.2201240
C	9.1035210	-9.8789380	0.8665930
C	8.8416100	-11.1705700	1.3075350
C	9.4408660	-12.2659010	0.6895380
C	10.3016370	-12.0544460	-0.3852000
C	10.5550590	-10.7680580	-0.8459950
C	10.4200780	6.6864060	1.7269840
C	9.6528560	6.3431310	2.8471800
C	10.1939230	6.4396850	4.1235660
C	11.4967440	6.8964390	4.3099230
C	12.2586560	7.2448040	3.1970430
C	11.7340580	7.1324960	1.9150080
C	-10.1848870	-7.5913230	-0.5313050
C	-10.4724610	-7.2300470	-1.8521740
C	-10.7590610	-8.2079150	-2.7972940
C	-10.7830250	-9.5536260	-2.4374440
C	-10.5070980	-9.9132270	-1.1201580
C	-10.2008450	-8.9436880	-0.1724720
H	-10.4044700	5.7635930	-0.0873370
H	-8.5884890	4.1105050	-0.2037030
H	-7.2583150	2.9113360	-1.6787050
H	-5.4622020	1.2520520	-1.7801710
C	1.2903940	2.6831980	-1.1683030
H	2.6571270	-4.2038710	-0.4117710
H	4.4697550	-5.8602440	-0.2797050
H	8.5879290	-4.1100560	-0.2036690

H	10.4039290	-5.7631260	-0.0873110
H	11.2167490	-10.6054490	-1.6915620
H	10.7715080	-12.8993090	-0.8818650
H	9.2394330	-13.2734100	1.0419080
H	8.1738780	-11.3188580	2.1521630
H	8.6460890	-9.0252640	1.3578110
H	12.4727770	-9.0495380	0.5609590
H	14.7701700	-8.4403160	-0.1227350
H	15.1445000	-7.0200290	-2.1303430
H	13.1889250	-6.2329380	-3.4511360
H	10.8955030	-6.8698200	-2.7803100
H	7.7078520	-8.8202880	-1.4275250
H	5.8943300	-7.1657730	-1.5557430
H	7.2578330	-2.9108050	-1.6786920
H	5.4617050	-1.2515330	-1.7801350
H	3.8234430	1.4245470	-3.0313960
H	5.6677630	3.0368310	-2.7412610
H	6.0987100	5.0513590	-1.6360290
H	7.7295940	6.8190070	-1.1418870
H	12.3356870	7.3950160	1.0500750
H	13.2794010	7.5957930	3.3236410
H	11.9129610	6.9791010	5.3096520
H	9.5814590	6.1694200	4.9799980
H	8.6314780	6.0022140	2.7101880
H	10.4645170	6.1788230	-2.1280250
H	10.9800690	7.9118870	-3.8197250
H	11.0160500	10.3141540	-3.1765980
H	10.5171290	10.9589060	-0.8269970
H	9.9767030	9.2249250	0.8519090
H	9.9933110	4.0603670	1.2432350
H	8.3548980	2.2947510	0.7567930
H	6.1796750	1.7087540	1.3092410
H	4.3640040	0.0699120	0.9957730
C	-1.2908770	-2.6827870	-1.1685000
H	-3.8237890	-1.4241500	-3.0314500
H	-5.6678960	-3.0366720	-2.7413390
H	-6.0984210	-5.0512010	-1.6361120
H	-7.7288810	-6.8192340	-1.1419280
H	-9.9749900	-9.2257470	0.8516770
H	-10.5150310	-10.9597610	-0.8273220
H	-11.0145270	-10.3149580	-3.1767860
H	-10.9795140	-7.9126150	-3.8196850
H	-10.4643420	-6.1795290	-2.1278850
H	-8.6308310	-6.0023660	2.7100840
H	-9.5805370	-6.1699950	4.9799650
H	-11.9116310	-6.9807600	5.3098360
H	-13.2779620	-7.5980900	3.3239430
H	-12.3345520	-7.3968740	1.0502990
H	-9.9932160	-4.0611130	1.2431990
H	-8.3552410	-2.2951000	0.7567020

H	-6.1801600	-1.7085240	1.3090980
H	-4.3646900	-0.0694660	0.9956560
H	-2.6576590	4.2042630	-0.4114600
H	-4.4702740	5.8606450	-0.2793680
H	-5.8947540	7.1662870	-1.5553600
H	-7.7082570	8.8208180	-1.4271310
H	-10.8958710	6.8704890	-2.7802580
H	-13.1892680	6.2337100	-3.4512680
H	-15.1449040	7.0207620	-2.1305430
H	-14.7706610	8.4409050	-0.1228160
H	-12.4732960	9.0500220	0.5610600
H	-8.6467050	9.0255800	1.3581890
H	-8.1745030	11.3191020	2.1527580
H	-9.2399120	13.2737650	1.0425600
H	-10.7718320	12.8998520	-0.8813730
H	-11.2170630	10.6060640	-1.6912830
C	-1.8499080	-3.1037710	0.1955560
H	-2.1021990	-2.7249360	-1.9036570
H	-0.5381170	-3.4103970	-1.4926970
C	-2.6503150	-4.3982840	0.1038750
H	-1.0243130	-3.2096120	0.9124350
H	-2.4977220	-2.3083110	0.5815530
C	-3.3595600	-4.7811870	1.3982570
H	-3.4062940	-4.2836790	-0.6867470
H	-1.9927990	-5.2204490	-0.2163710
C	-4.2033440	-6.0426720	1.2504700
H	-2.6230020	-4.9158440	2.2041480
H	-4.0073940	-3.9479230	1.7072630
C	-4.9750610	-6.4313910	2.5064840
H	-4.9187460	-5.8954940	0.4297980
H	-3.5587160	-6.8813200	0.9473630
C	-5.8914040	-7.6310650	2.2857790
H	-4.2695610	-6.6475960	3.3225300
H	-5.5772640	-5.5737840	2.8426390
C	-6.5865180	-8.1213150	3.5509040
H	-6.6485160	-7.3664320	1.5323040
H	-5.3110350	-8.4587730	1.8513480
C	-7.5717510	-9.2555290	3.2895260
H	-5.8316770	-8.4502570	4.2805030
H	-7.1194420	-7.2851950	4.0273480
C	-8.2874280	-9.7310090	4.5467340
H	-8.3145490	-8.9140000	2.5554040
H	-7.0394010	-10.0953670	2.8208380
H	-8.9716890	-10.5593190	4.3316920
H	-7.5724970	-10.0752420	5.3040260
H	-8.8792220	-8.9198670	4.9865180
C	1.8493250	3.1041230	0.1958110
H	2.1017790	2.7253210	-1.9033950
H	0.5376980	3.4108540	-1.4925460
C	2.6500700	4.3984250	0.1041350

H	1.0236540	3.2102130	0.9125660
H	2.4968930	2.3085320	0.5819510
C	3.3591920	4.7813380	1.3985810
H	3.4061560	4.2835310	-0.6863420
H	1.9928170	5.2207150	-0.2163280
C	4.2034630	6.0424780	1.2506410
H	2.6225250	4.9164480	2.2042950
H	4.0066670	3.9479110	1.7079010
C	4.9751060	6.4312600	2.5066820
H	4.9189530	5.8948290	0.4301310
H	3.5591950	6.8812820	0.9472010
C	5.8918610	7.6305800	2.2857820
H	4.2695490	6.6479020	3.3225620
H	5.5769860	5.5735480	2.8431490
C	6.5871540	8.1208290	3.5508080
H	6.6488790	7.3655520	1.5323510
H	5.3117780	8.4584110	1.8512040
C	7.5725850	9.2548230	3.2892140
H	5.8324310	8.4500060	4.2804230
H	7.1199440	7.2846580	4.0273150
C	8.2885580	9.7302360	4.5462780
H	8.3151900	8.9131060	2.5549830
H	7.0403330	10.0947420	2.8205590
H	8.9729180	10.5584260	4.3310800
H	7.5738120	10.0746260	5.3036730
H	8.8802930	8.9190200	4.9860030
